# The genera in the second catalogue (1833–1836) of Dejean’s Coleoptera collection

**DOI:** 10.3897/zookeys.282.4401

**Published:** 2013-04-02

**Authors:** Yves Bousquet, Patrice Bouchard

**Affiliations:** 1 Canadian National Collection of Insects, Arachnids and Nematodes, Agriculture and Agri-Food Canada, 960 Carling Avenue, Ottawa, Ontario, K1A 0C6, Canada

**Keywords:** Beetles, nomenclature, Dejean, genus-group names, type species

## Abstract

All genus-group names listed in the second edition of the catalogue (1833-1836) of Dejean’s beetle collection are recorded. For each new genus-group name the originally included available species are listed and for generic names with at least one available species, the type species and the current status are given. Names available prior to the publication of Dejean’s second catalogue (1833-1836) are listed in an appendix.

The following new synonymies are proposed: *Cyclonotum* Dejean, 1833 (= *Dactylosternum* Wollaston, 1854) [Hydrophilidae], *Hyporhiza* Dejean, 1833 (= *Rhinaspis* Perty, 1830) [Scarabaeidae], *Aethales* Dejean, 1834 (= *Epitragus* Latreille, 1802) [Tenebrionidae], *Arctylus* Dejean, 1834 (= *Praocis* Eschscholtz, 1829) [Tenebrionidae], *Euphron* Dejean, 1834 (= *Derosphaerus* Thomson, 1858) [Tenebrionidae], *Hipomelus* Dejean, 1834 (= *Trachynotus* Latreille, 1828) [Tenebrionidae], *Pezodontus* Dejean, 1834 (= *Odontopezus* Alluaud, 1889) [Tenebrionidae], *Zygocera* Dejean, 1835 (= *Disternopsis* Breuning, 1939) [Cerambycidae], and *Physonota* Chevrolat, 1836 (= *Anacassis* Spaeth, 1913) [Chrysomelidae]. *Heterogaster pilicornis* Dejean, 1835 [Cerambycidae] and *Labidomera trimaculata* Chevrolat, 1836 [Chrysomelidae] are placed for the first time in synonymy with *Anisogaster flavicans* Deyrolle, 1862 and *Chrysomela clivicollis* Kirby, 1837 respectively. Type species of the following genus-group taxa are proposed: *Sphaeromorphus* Dejean, 1833 (*Sphaeromorphus humeralis* Erichson, 1843) [Scarabaeidae], *Adelphus* Dejean, 1834 (*Helops marginatus* Fabricius, 1792) [Tenebrionidae], *Cyrtoderes* Dejean, 1834 (*Tenebrio cristatus* DeGeer, 1778) [Tenebrionidae], *Selenepistoma* Dejean, 1834 (*Opatrum acutum* Wiedemann, 1823) [Tenebrionidae], *Charactus* Dejean, 1833 (*Lycus limbatus* Fabricius, 1801) [Lycidae], *Corynomalus* Chevrolat, 1836 (*Eumorphus limbatus* Olivier, 1808) [Endomychidae], *Hebecerus* Dejean, 1835 (*Acanthocinus marginicollis* Boisduval, 1835) [Cerambycidae], *Pterostenus* Dejean, 1835 (*Cerambyx abbreviatus* Fabricius, 1801) [Cerambycidae], *Psalicerus* Dejean, 1833 (*Lucanus femoratus* Fabricius, 1775) [Lucanidae], and *Pygolampis* Dejean, 1833 (*Lampyris glauca* Olivier, 1790) [Lampyridae]. A new name, *Neoeutrapela* Bousquet and Bouchard [Tenebrionidae], is proposed for *Eutrapela* Dejean, 1834 (junior homonym of *Eutrapela* Hübner, 1809).

The following generic names, made available in Dejean’s catalogue, were found to be older than currently accepted valid names: *Catoxantha* Dejean, 1833 over *Catoxantha* Solier, 1833 [Buprestidae], *Pristiptera* Dejean, 1833 over *Pelecopselaphus* Solier, 1833 [Buprestidae], *Charactus* Dejean, 1833 over *Calopteron* Laporte, 1836 [Lycidae], *Cyclonotum* Dejean, 1833 over *Dactylosternum* Wollaston, 1854 [Hydrophilidae], *Ancylonycha* Dejean, 1833 over *Holotrichia* Hope, 1837 [Scarabaeidae], *Aulacium* Dejean, 1833 over *Mentophilus* Laporte, 1840 [Scarabaeidae], *Sciuropus* Dejean, 1833 over *Ancistrosoma* Curtis, 1835 [Scarabaeidae], *Sphaeromorphus* Dejean, 1833 over *Ceratocanthus* White, 1842 [Scarabaeidae], *Psalicerus* Dejean, 1833 over *Leptinopterus* Hope, 1838 [Lucanidae], *Adelphus* Dejean, 1834 over *Praeugena* Laporte, 1840 [Tenebrionidae], *Amatodes* Dejean, 1834 over *Oncosoma* Westwood, 1843 [Tenebrionidae], *Cyrtoderes* Dejean, 1834 over *Phligra* Laporte, 1840 [Tenebrionidae], *Euphron* Dejean, 1834 over *Derosphaerus* Thomson, 1858 [Tenebrionidae], *Pezodontus* Dejean, 1834 over *Odontopezus* Alluaud, 1889 [Tenebrionidae], *Anoplosthaeta* Dejean, 1835 over *Prosopocera* Blanchard, 1845 [Cerambycidae], *Closteromerus* Dejean, 1835 over *Hylomela* Gahan, 1904 [Cerambycidae], *Hebecerus* Dejean, 1835 over *Ancita* Thomson, 1864 [Cerambycidae], *Mastigocera* Dejean, 1835over *Mallonia* Thomson, 1857 [Cerambycidae], *Zygocera* Dejean, 1835 over *Disternopsis* Breuning, 1939 [Cerambycidae], *Australica* Chevrolat, 1836 over *Calomela* Hope, 1840 [Chrysomelidae], *Edusa* Chevrolat, 1836 over *Edusella* Chapuis, 1874 [Chrysomelidae], *Litosonycha* Chevrolat, 1836 over *Asphaera* Duponchel and Chevrolat, 1842 [Chrysomelidae], and *Pleuraulaca* Chevrolat, 1836 over *Iphimeis* Baly, 1864 [Chrysomelidae]. In each of these cases, Reversal of Precedence (ICZN 1999: 23.9) or an applicationto the International Commission on Zoological Nomenclature will be necessary to retain usage of the younger synonyms.

## Introduction

Dejean published four catalogues of the beetles in his collection. These are straightforward lists of species in his collection, with indication of the place of collection, arranged under generic names in five major groups (Pentamères, Hétéromères, Tétramères, Trimères and Dimères). The first catalogue, published in 1802, was not for sale (Boisduval 1846: 501) and if not for the fact that Dejean distributed many copies at a meeting of the *Société entomologique de France* in 1837, it would probably have gone unnoticed. This catalogue is not important nomenclaturally as it contains no new available names. The other catalogues were published in 1821, 1833–1836, and 1836–1837 and were referred to by Dejean as the first, second and third editions. These are important nomenclaturally as many new genus-group names were made available for the first time by the inclusion of one or more available specific names (see ICZN 1999: Article 12.2.5).

Silfverberg (1983, 1984a, 1984b) commented on the genera introduced by Dejean in the first edition of his catalogue published in 1821. The objective of this paper is to summarize, for the first time, the nomenclatural status of all genus-group names listed in the second catalogue of Dejean’s Coleoptera collection published between 1833 and 1836.

## Biographical notice of Dejean

**Figure 1.**
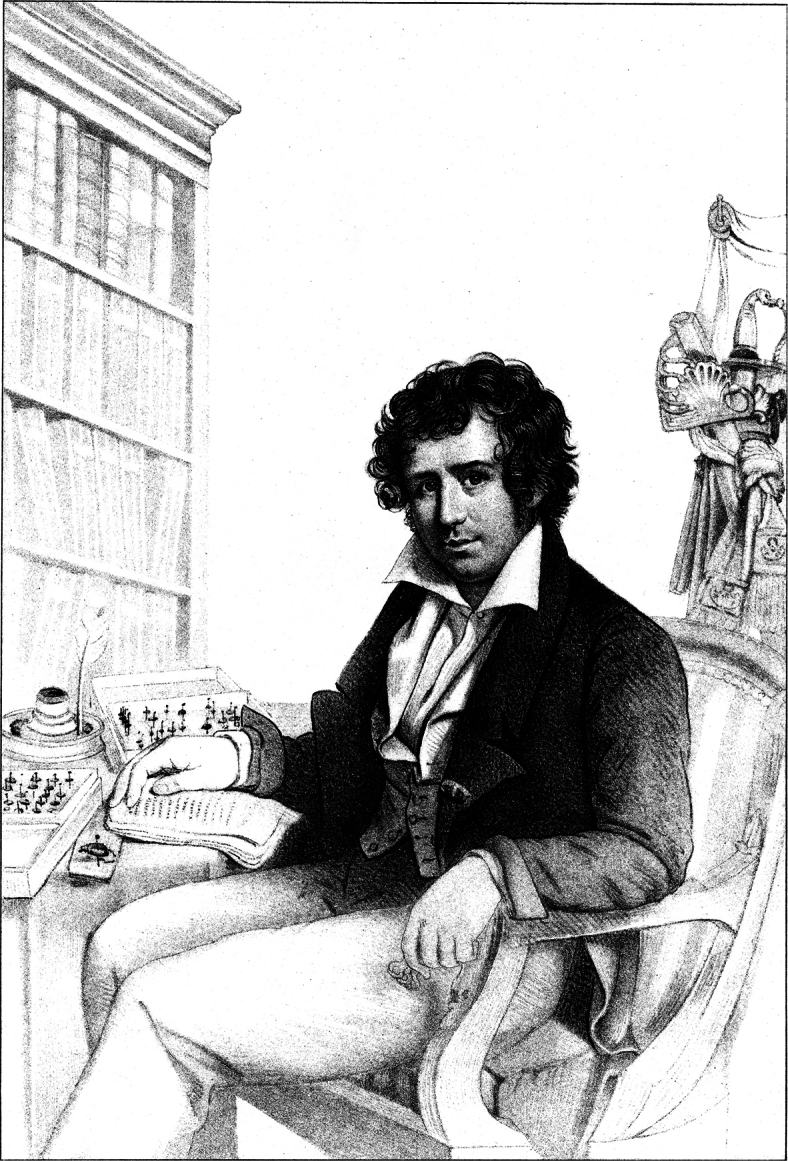
Portrait of Count Pierre François Marie Auguste Dejean [1780-1845].

Pierre François Marie Auguste Dejean (Fig. 1) was born on the 10^th^ of August 1780 at Amiens, a manufacturing city in the department of Somme, about 115 kilometers north of Paris. His father, Jean-François Aimé, Comte Dejean [1749–1824], became a military officer and played an important role in the political arena of France; he became minister of administration of war under Napoleon Bonaparte (Tranié 2001: 280). Dejean was interested in entomology by the age of 13 and at that time collected mainly Lepidoptera together with André Marie Constant Duméril [1774–1860] who was six years his senior. However, shortly after these first entomological steps, Dejean decided to devote himself to the study of Coleoptera. By the age of 15, *Citoyen Dejean* enrolled in the army and until 1815 participated in a series of campaigns that brought him to several countries including Spain, Portugal, Italy, Austria, Poland, Germany and Russia. At Waterloo, in June 1815, he stood as general of division and aide-de-camp to Napoleon Bonaparte.

Dejean was a beetle collector and, despite his military obligations, continued to build his collection through his own collecting in countries where his military activity took him and through exchanges or gifts he received. Even on the battlefield he kept his eyes open for interesting specimens. As his youngest daughter wrote in the preface of one of her poetry books (Mahul 1869) “Lui même il racontait que pendant la bataille [battle of Alcanizas, Spain, in 1809] arrêtant son cheval au fort de la mitraille il fixait à son casque un insecte léger [it was a specimen of *Cebrio*] puis de nouveau courait au devant du danger”[He recounted himself that during the battle he stopped his horse to attach a small insect to his helmet and then carried on forward to combat]. This anecdote is reported in biographies on Dejean although the details differ slightly from one account to another.

After the fall of Napoleon in June 1815, Dejean was one of 38 persons condemned to exile. He left France and for the next three years he traveled mostly on foot with a servant, collecting beetles in the eastern parts of the Austrian empire, visiting successively Carinthia, Carniola, Croatia and Dalmatia. He was about to leave for Hungary when his father obtained a pardon on his behalf from Louis XVIII. Dejean returned to Paris by the end of 1818 and remained lieutenant general on reserve until 1830. During that period he probably spent most of his time working on his collection and publications although he also participated in political activities in France as he became a member of the *Chambre des Pairs* in 1824 following the death of his father. He returned to duty briefly in 1831-1832 and was in charge of the cavalry associated with the Anvers Expedition to support the Belgians fighting for their independence against the Dutch. In January 1833 he was named Grand Officer and in April 1844 Grand Cross of the Legion of Honour.

After his return to Paris in 1818, Dejean was rich and respected as were all the generals that served under Napoleon. He financed several collecting expeditions, and also bought a number of collections including that of Pierre André Latreille, around 1826, which added 1700 species to his collection (Dejean 1828: vi). At the second meeting of the *Société entomologique de France*, on 7 February 1832, the honorary members of the *Société* (which were limited to 12, including one-third from outside of France) were announced. Dejean was not selected. This probably upset the General and may be the reason why he distanced himself from the *Société* in its early years (Bousquet 2004: 36). However, he eventually became a member of the *Société* in 1837 and was elected President for the year 1840.

Around the time of the last livraison of his third catalogue, Dejean in 1837, his sight weakening, talked about selling his collection which at the time was certainly the largest beetle collection ever assembled by one person. Negotiations then started with the French government to place the collection at the *Jardin des Plantes* in Paris. Dejean asked for 50,000 francs for the beetles and 10,000 francs for the Lepidoptera and miscellaneous orders (Anonymous 1840a: 373). However, negotiations with the government failed. The King of Prussia tried to acquire the collection but Dejean refused his offer. Since nobody in France was able to raise the money, Dejean’s collection was finally sold in parts, during 1840, as advertised in a prospectus published in the *Bulletin de la Société Impériale des Naturalistes de Moscou* (Anonymous 1840a). At the time, his collection contained 24,643 species and more than 118,000 specimens (Anonymous 1840a: 371; Mannerheim 1842: 869). In November 1840, Dejean also offered his library for sale (Anonymous 1840b).

Dejean died on the 17^th^ of March 1845, aged 64, after a lengthy illness, at his residence on 17 rue de l’Université, Paris. He was survived by his wife Adèle Barthélemy, whom he married in 1802, and their five children.

## Dates of publication

Dejean’s second catalogue was published in five livraisons (i.e., fascicles). There are no dates inside the publication, except for the year on the cover of each part. The earliest dates on which each part of the work was demonstrated to be in existence were discussed by Madge (1988: 318). These dates are to be adopted as the date of publication for each part (ICZN 1999: Article 21.7). They are as follows: livraison 1 [pp. 1–96], 19 January 1833; livraison 2 [pp. 97–176], 27 July 1833; livraison 3 [pp. 177–256], April-June 1834 (for nomenclatural purposes, 30 June 1834); livraison 4 [pp. 257–360], 22 August 1835; livraison 5 [pp. 361–443], end of 1836 (for nomenclatural purposes, 31 December 1836). The dates for the first, second and fourth livraisons are taken from the *Bibliographie de la France*, a weekly recording journal for publications issued in France. The date for the third livraison came from the notice of new literature of the second quarter (April–June) in the *Annales de la Société Entomologique de France* in 1834 (p. xxxv). The date for the last livraison is more problematic. The wrapper of the last livraison on the copy we have seen, and those on two copies seen by Madge (1988: 320), are dated 1834 and can be explained by the fact that the publisher used old wrappers from the third livraison. Madge (1988: 320) noted that the last livraison was published in 1836, probably toward the end of the year, because it was noted by Erichson (1837b: 285) in his notice of the entomological literature of 1836 published in *Archiv für Naturgeschichte*.

As pointed out by Madge (1988), the fifth livraison of the second and third editions of Dejean’s catalogues, where the *chrysomélines* and *trimères* sections appeared, were printed from the same type but published at different times. Madge (1988) commented that the most logical explanation is that both livraisons were printed in 1836 (possibly at about the same time) but the release of the last livraison of the third edition was delayed possibly to give Dejean time to complete a preface. Since the fifth livraison of the second and third editions are identical (except for the page number), a number of genera at the end of the fourth livraison of the second edition were duplicated at the beginning of the fifth livraison. The duplicated genera are *Megalopus* (part, pp. 358 and 361), *Megascelis* (pp. 358 and 361), *Orsodacna* (pp. 359 and 361), *Syneta* (pp. 359 and 361), *Auchenia* (pp. 359 and 361), *Lema* (pp. 359 and 362), and *Alurnus* (part, pp. 360 and 363).

## Authorship

As frequently done in the first half of the 19th Century, taxonomists and collectors proposed scientific names for new species in their collections. However, specimens were often exchanged, sold or given away to collaborators before they were formally described in scientific literature. As a result, many of these species were eventually described by authors other than the original ones, although they usually retained the original scientific names and credited the person who proposed the name. Dejean received specimens from many correspondents over time and retained the original names and authors in his publications. It should be noted that since Dejean is the author of the second catalogue of his beetle collection, he is to be credited with all new available genus-group names even though they are credited to other authors in his publication (see ICZN 1999: Article 50.1). There is one exception to this and it concerns the new generic names attributed to Chevrolat in the *chrysomélines* [pp. 356-431] and *trimères* [pp. 432–440] sections. In the *avertissement* (i.e., preface) of the third edition of his catalogue, Dejean (1837: xiii) wrote “Quant aux *chrysomélines* et à la section des *trimères*, M. Chevrolat s’en étant particulièrement occupé, je l’ai prié de vouloir bien m’aider de ses conseils, et j’ai adopté tous les genres qu’il a créés au dépens des anciens grands genres… Je le prie de recevoir mes remercîments pour la part qu’il a bien voulu prendre à la rédaction de ce Catalogue” [As for the sections relating to the *chrysomélines* and the *trimères*, which M. Chevrolat dedicated himself to, I asked him to help me with his advice, and I have used all the genera that he created at the expense of the older large genera… I ask him to accept my thanks for the part he took in the compilation of this Catalogue]. The preface was certainly intended to be distributed with the last livraison of Dejean’s second catalogue; however all the undistributed copies of the first four livraisons were destroyed in a fire in Paris on the 12^th^ of December 1835. Because of this, Dejean decided to release the preface only with the third edition of his catalogue which was published soon after in 1836 and 1837.

In the past decades, Chevrolat’s names in Dejean’s second edition for the *chrysomélines* and *trimères* sections have been attributed to Chevrolat by almost all authors we have seen. However, Konstantinov et al. (2011: 17) argued, in reference to the name *Plectroscelis*, that it should be credited to Dejean since it is not explicitly demonstrated in the work itself that Chevrolat alone was responsible for the availability of the name (see ICZN 1999: Article 50.1.1). Strictly speaking this is true because Dejean’s comments about the involvement of Chevrolat appeared only in the third edition of his catalogue, not in his second catalogue as originally intended. However, we believe that using Dejean as the author of *Plectroscelis* and several other genera in the *chrysomélines* and *trimères* goes against the current trend in recent works on Coleoptera. An application to the Commission may be useful to settle this issue. In this work, the generic names attributed to Chevrolat in the *chrysomélines* and *trimères* sections are credited to Chevrolat.

## Precedence

A number of works published between 1833 and 1836 have important nomenclatural significance for the names that appear in Dejean’s second catalogue. These works either contain genus-group names that take precedence over names included in Dejean’s catalogue or include new species-group names listed in Dejean’s catalogue. In order to establish precedence, we have ascertained the dates of publication of these works.

[1] Solier, A.J.J. 1833. Essai sur les buprestides. *Annales de la Société Entomologique de France* 2: 261–316. This paper was published in the second issue of the second volume of the *Annales* which was recorded on 19 August 1833 by the *Académie des Sciences* (France) (published in *L’Institut, Journal des Académie et Sociétés Scientifiques* 1: 121). Therefore Solier’s publication was published after livraison 1 of Dejean’s catalogue recorded on 19 January 1833.

[2] Laporte, F.L.N. 1833. Essai d’une révision du genre Lampyre. *Annales de la Société Entomologique de France* 2: 122–153. This paper was published in the first issue of the second volume of the *Annales* which was recorded on 17 June 1833 by the *Académie des Sciences* (France) (published in *L’Institut, Journal des Académie et Sociétés Scientifiques* 1: 41). Therefore Laporte’s publication was published before livraison 2 of Dejean’s catalogue recorded on 27 July 1833 and his names are considered older than Dejean’s names.

[3] Gory, H.L. and Percheron, A. 1833. *Monographie des cétoines et genres voisins, formant, dans les familles naturelles de Latreille, la division des scarabées mélitophiles*. J.B. Ballière, Paris [&] Londres. 410 pp. + 77 pls. This book was issued in 15 livraisons and the first one (pp. 1–73) was recorded on 25 May 1833 by the *Bibliographie de la France*. Therefore this livraison was issued before the second livraison of Dejean’s catalogue recorded on 27 July 1833, also by the *Bibliographie de la France*.

[4] Schönherr, C.J. 1834. *Genera et species curculionidum, cum synonymia hujus familiae; species novae aut hactenus minus cognitae, descriptionibus A Dom. Leonardo Gyllenhal, C. H. Boheman, et entomologis aliis illustratae. Tomus secundus. Pars prima*. Roret, Parisiis. 326 + [1] pp. This book was recorded on 7 May 1834 by the *Société Entomologique de France* and so is considered to precede Dejean’s third livraison, dated 30 June 1834.

[5] Boisduval, J.B.A. 1835. *Voyage de découvertes de l’Astrolabe exécuté par ordre du Roi, pendant les années 1826-1827-1828-1829, sous le commandement de M. J. Dumont d’Urville. Faune entomologique de l’Océan Pacifique, avec l’illustration des insectes nouveaux receuillis pendant le voyage. Deuxième partie. Coléoptères et autres ordres*. J. Tatsu, Paris. vii + 716 pp. This book was recorded on 27 March 1835 by the *Société de Géographie de France* (Evenhuis 1997: 104) and so precedes Dejean’s fourth livraison recorded on 22 August 1835. Thus, Boisduval’s species-group names were made available before Dejean (1835).

[6] Schönherr, C.J. 1835. *Genera et species curculionidum, cum synonymia hujus familiae; species novae aut hactenus minus cognitae, descriptionibus A Dom. Leonardo Gyllenhal, C. H. Boheman, et entomologis aliis illustratae. Tomus tertius. Pars prima*. Roret, Parisiis. [3] + 505 pp. Although dated 1836 on the title page, this book was published in 1835. It was presented to the *Société Entomologique de France* on 2 December 1835 *(Ann. Soc. Ent. Fr*. 4: lxxvi). Dejean’s fourth livraison, recorded on 22 August 1835, precedes Schönherr’s publication and thus Dejean’s names have precedence over those of Schönherr (1835).

[7] Audinet-Serville, J.G. 1835. Nouvelle classification de la famille des longicornes (suite). *Annales de la Société Entomologique de France* 4: 5–100. Audinet-Serville’s paper appeared in the first issue of the fourth volume of the *Annales*. This issue was recorded on 6 July 1835 by the *Académie des Sciences* (France) (published in *L’Institut, Journal des Académie et Sociétés Scientifiques* 3: 217) and so is considered to precede the fourth livraison of Dejean’s catalogue recorded on 22 August 1835.

[8] Audinet-Serville, J.G. 1835. Nouvelle classification de la famille des longicornes (suite). *Annales de la Société Entomologique de France* 4: 197–228. This article was published in the second issue of the fourth volume of the *Annales* which was recorded on the 28 September 1835 by the *Académie des Sciences* (France). Therefore, the second part of Audinet-Serville’s work (1835) appeared after Dejean’s fourth livraison of his catalogue recorded on 22 August 1835 and Dejean’s names have precedence.

[9] Chevrolat, A. 1835. Mémoire sur un coléoptère tétramère de la famille des xylophages, et observations sur plusieurs espèces de cet ordre, rencontrées dans diverses fourmillières. *Revue Entomologique* 3: 263–269. This paper was published in livraison 17 of the *Revue Entomologique* which was presented to the *Société Entomologique de France* on 5 August 1835 (*Ann. Soc. Ent. Fr*. 4: liv). Dejean’s fourth livraison, recorded on 22 August 1835, was probably published later and so Chevrolat’s new genus-group name (*Myrmechixenus*) has precedence over the same name in Dejean (1835).

[10] Faldermann, F. 1835.Coleopterorum ab illustrissimo Bungio in China boreali, Mongolia, et montibus Altaicus collectorum, nec non ab ill. Turczaninoffio et Stchukino e provincia Irkutzk missorum illustrationes. *Mémoires présentés à l’Académie Impériale des Sciences de Saint-Pétersbourg par divers savans et lus dans ses assemblées* (série 6) 2: 337–464. This volume was published in August 1835 (for nomenclatural purposes, 31 August 1835) as indicated on the recto of the title page of the volume. Consequently the names in Dejean’s fourth livraison, recorded on 22 August 1835, have precedence.

## Methods

All genus-group names listed in Dejean’s second catalogue are treated. Those available prior to the publication of Dejean’s catalogue are listed with their currently accepted authorship and year in the Appendix. All new genus-group names are listed, whether they are available or not. For each new genus-group name, we have determined first the originally included available species and, for available names (e.g., those that include at least one available species-group name), the type species and the current status.

Originally included available species are those that were cited by name and available at the time of publication of the livraisons of Dejean’s catalogue, whether they were listed as valid or invalid. Any one of them can be selected as type species with the exception that when only one species is listed as valid, that species is the type species by monotypy regardless of any cited synonyms or varieties (ICZN 1999: Article 68.3). A species name followed by a question mark in Dejean’s catalogue indicates that Dejean was uncertain if his identification was correct. Such species are considered as “*species inquirenda*” and are deemed not to be originally included (ICZN 1999: Article 67.2.5); they cannot be selected as type species. An author’s name followed by a question mark indicates that Dejean was uncertain about the author’s name; these species are, nevertheless, originally included species.

Dejean regularly misattributed the authorship of the species-group names listed in his catalogue. For example, almost all species first described by Linnaeus were attributed to Fabricius. Those described by Dalman, Gyllenhal and others in Schönherr’s *Synonymia insectorum* were credited to Schönherr. Species described by Boisduval in the *Voyage de découvertes de l’Astrolabe* were often credited to d’Urville who was in charge of the expedition. We determined the correct authorship of species-group names listed in Dejean’s catalogue by checking the primary literature and the *Index Animalium* of Sherborn for similar scientific names with identical provenance and placed in the same taxonomic group.

We have considered all species-group names attributed by Dejean to himself as unavailable except in the following two cases. First, when an author, prior to the publication of Dejean’s catalogue, proposed an identical specific name that he attributed to Dejean. This is the case, for example, with several species described by Klug (1829). Second, when an author, prior to the publication of Dejean’s catalogue, proposed an identical specific name and stipulated that the specimen(s) was in the collection of Dejean. These are available species-group names in Dejean’s catalogue but are attributed to the authors that made them available earlier.

Unless indicated otherwise, we have assumed correct identity for all available species-group names listed in Dejean’s catalogue.

Dejean divided the Coleoptera into five major groups: Pentamères, Hétéromères, Tétramères, Trimères and Dimères. The Pentamères were further divided into the Carabiques (carabids), Hydrocanthares (dytiscids, noterids, haliplids, gyrinids), Brachélytres (staphylinids in part), Sternoxes (buprestids, elaterids, eucnemids), Malacodermes (rhipicerids, ptilodactylids, scirtids, lycids, lampyrids, cantharids, melyrids, etc.), Terediles (clerids, lymexylids, ptinids, etc.), Clavicornes (silphids, nitidulids, cryptophagids, dermestids, histerids, byrrhids, heterocerids, etc.), Palpicornes (hydraenids, hydrophilids) and Lamellicornes (scarabaeids, hybosorids, trogids, geotrupids, glaphyrids, lucanids, passalids). The Hétéromères were subdivided into the Mélasomes (tenebrionids in part), Taxicornes (tenebrionids in part, zopherids, leiodids in part, tetratomids), Ténébrionites (tetratomids, melandryids, pythids, tenebrionids in part, borids), Hélopiens (tenebrionids in part), Trachélides (tenebrionids in part, pyrochroids, anthicids, aderids, scraptiids, ripiphorids, mordellids, etc.), Vésicants (meloids) and Sténélytres (oedemerids, mycterids, salpingids). Finally, the Tétramères were divided into the Curculionites (Curculionoidea except scolytines and platypodines), Xylophages (scolytines, platypodines, bostrichids, sphindids, latridiids, mycetophagids, cerylonids, monotomids, cucujids, etc.), Longicornes (cerambycids), and Chrysomélines (chrysomelids, orsodacnids, megalopodids, erotylids, phalacrids, leiodids in part, corylophids). The Trimères (coccinellids, endomychids, dasycerines) and Dimères (pselaphines) were not divided any further. The same divisions are retained in this publication.

## List of genus-group names in Dejean’s second catalogue (1833–1836)

Below is a list of all new genus-group names proposed in Dejean’s second catalogue following the taxonomic arrangement used in his catalogue. No new genera were proposed in the Pentamères: Carabiques and the Dimères. The Appendix gives all generic names listed in Dejean (1833-1836a) which were available prior to the publication of his catalogue.

Many generic names were proposed for the first time in Dejean’s second catalogue as invalid synonyms. According to the ICZN (1999: Article 11.6.1), a name originally published as junior synonym of an available name can be available from its first publication as synonym if it had been treated before 1961 as an available name and either adopted as the name of a taxon or treated as a senior homonym. The originally included species are the species (cited by available names) first directly associated with the synonym (ICZN 1999: Article 67.12). Except for the name *Sphaeromorphus* Dejean, the species first directly associated with the synonym are the species listed by Dejean following the synonym. Three names first published as junior synonyms in Dejean’s second catalogue are available: *Adoretus* Dejean, 1833, *Sphaeromorphus* Dejean, 1833, and *Pterostenus* Dejean, 1835.

In several instances, Dejean proposed a new generic name as valid while he listed a genus name in synonymy that was already available. It is obvious that Dejean proposed replacement names or emendations in most cases, sometimes with reason because of homonymy, but on other occasions without apparent reason. In five of those cases (*Ampedus*, *Saerangodes*, *Eutrapela*, *Plocaederus*, and *Amphionycha*), the names were not considered as replacement names because it was self-evident that it was not Dejean’s intention; these names are interpreted as new names. Two names are interpreted as unjustified emendations, *Pandarus* Dejean, 1834 for *Dendarus* Dejean, 1821 and *Monohammus* Dejean, 1835 for *Monochamus* Dejean, 1821. All other names are interpreted as replacement names.

### Pentamères: Hydrocanthares

***Cybister* Dejean, 1833: 59** (as “Cybister. *Eschscholtz*.”)

Originally included available species: *Gyrinus cinctus* Germar, 1824.

Type species: *Gyrinus cinctus* Germar, 1824 by monotypy.

Current status: junior homonym of *Cybister* Curtis, 1827 [Dytiscidae]; senior synonym of *Gyretes* Brullé, 1835 in Gyrinidae (*fide* Hope 1838: 145).

***Cyclous* Dejean, 1833: 58** (as “Cyclous. *Eschscholtz*.”)

Originally included available species: *Gyrinus americanus* Linnaeus, 1767 (as “Americanus. *Fabr*.”); *Gyrinus australis* Fabricius, 1775; *Gyrinus longimanus* Olivier, 1791; *Gyrinus micans* Fabricius, 1792; *Gyrinus spinosus* Fabricius, 1781; *Gyrinus vittatus* Germar, 1824.

Type species: *Gyrinus australis* Fabricius, 1775 by subsequent designation (Hope 1838: 145).

Current status: valid subgenus of *Dineutus* Macleay, 1825 in Gyrinidae (*fide* Lawrence et al. 1987: 366).

***Cymatopterus* Dejean, 1833: 54** (as “Cymatopterus. *Eschscholtz*.”)

Originally included available species: *Dytiscus bogemanni* Gyllenhal, 1813; *Dytiscus dolabratus* Paykull, 1798; *Dytiscus fuscus* Linnaeus, 1758 (as “Fuscus. *Fabr*.”); *Dytiscus striatus* Linnaeus, 1758 (as “Striatus. *Fabr*.”).

Type species: *Dytiscus fuscus* Linnaeus, 1758 by subsequent designation (Thomson 1859: 13).

Current status: junior subjective synonym of *Colymbetes* Clairville, 1806 in Dytiscidae (*fide* Nilsson 2003: 45).

***Epinectus* Dejean, 1833: 58** (as “Epinectus. *Eschscholtz*.”)

Originally included available species: none.

***Graphoderus* Dejean, 1833: 54** (as “Graphoderus. *Eschscholtz*.”)

Originally included available species: *Dytiscus bilineatus* DeGeer, 1774 (as “Bilineatus. *Payk*.”); *Dytiscus cinereus* Linnaeus, 1758 (as “Cinereus. *Fabr*.”); *Dytiscus verrucifer* Sahlberg, 1824; *Dytiscus vittatus* Fabricius, 1775; *Dytiscus zonatus* Hoppe, 1795 (as “Zonatus. *Fabr*.”).

Type species: *Dytiscus cinereus* Linnaeus, 1758 by subsequent designation (Westwood 1838: 8).

Current status: valid genus in Dytiscidae (*fide* Nilsson 2003: 49).

***Liopterus* Dejean, 1833: 54** (as “Liopterus. *Eschscholtz*.”)

Originally included available species: *Dytiscus oblongus* Illiger, 1801.

Type species: *Dytiscus oblongus* Illiger, 1801 (= *Dytiscus haemorrhoidalis* Fabricius, 1787) by monotypy.

Current status: junior subjective synonym of *Copelatus* Erichson, 1832 in Dytiscidae (*fide* Nilsson 2003: 47).

***Nogrus* Dejean, 1833: 53** (as “Nogrus. *Eschscholtz*.”)

Originally included available species: *Dytiscus griseus* Fabricius, 1781; *Dytiscus sticticus* Linnaeus, 1767 (as “Var. *Sticticus. Fabr*.”).

Type species: *Dytiscus griseus* Fabricius, 1781 (= *Dytiscus sticticus* Linnaeus, 1767) by monotypy.

Current status: objective synonym of *Eretes* Laporte, 1833 in Dytiscidae (*fide* Nilsson 2003: 53).

Comments. The name *sticticus* is listed as a variety of *griseus* in Dejean’s catalogue; therefore the type species of *Nogrus* is *griseus* by monotypy (ICZN 1999: Article 68.3).

The name *Eretes* Laporte was proposed in the fourth and last issue of the first volume (1832) of the *Annales de la Société Entomologique de France* which was likely published in 1833, probably after the first livraison of Dejean’s catalogue (recorded on 19 January 1833). Therefore *Nogrus* Dejean is probably older than *Eretes* Laporte although we are unable to confirm it.

***Orectochilus* Dejean, 1833: 59** (as “Orectochilus. *Eschscholtz*.”)

Originally included available species: *Gyrinus gangeticus* Wiedemann, 1821 (as “Gangiticus. *Wiedemann*.”); *Gyrinus villosus* Müller, 1776 (as “Villosus. *Fabr*.”).

Type species: *Gyrinus villosus* Müller, 1776 by subsequent designation (Westwood 1838: 8).

Current status: valid genus in Gyrinidae (*fide* Mazzoldi 2003: 28).

***Rhantus* Dejean, 1833: 54** (as “Rantus. *Eschscholtz*.”)

Originally included available species: *Dytiscus adspersus* Panzer, 1797 (as “Adspersus. *Fabr*.”); *Dytiscus agilis* Fabricius, 1792; *Dytiscus collaris* Paykull, 1798 (as “*Collaris. Gyllenhal*.”); *Dytiscus conspersus* Marsham, 1802 (as “*Conspersus. Gyllenhal*.); *Colymbetes insolatus* Gebler, 1830 (as “*Insolatus. Eschsch*.”); *Dytiscus notatus* Fabricius, 1781; *Colymbetes pulverosus* Stephens, 1828 (as “*Pulverosus. Knoch*.”).

Type species: *Colymbetes pulverosus* Stephens, 1828 (= *Colymbetes suturalis* Macleay, 1825) by subsequent designation (Hope 1838: 131).

Current status: valid genus in Dytiscidae (*fide* Nilsson 2003: 45).

Comments. The spelling of Dejean’s name was fixed in Opinion 289 (ICZN 1954).

***Scutopterus* Dejean, 1833: 54** (as “Scutopterus. *Eschscholtz*.”)

Originally included available species: *Dytiscus lanio* Fabricius, 1775; *Dytiscus pustulatus* Rossi, 1792.

Type species: *Dytiscus lanio* Fabricius, 1775 by subsequent designation (Nilsson et al. 1989: 307).

Current status: name suppressed in Dytiscidae.

Comments. The name *Scutopterus* Dejean was suppressed for the purposes of the Principle of Priority in Opinion 1725 (ICZN 1993).

***Thermonetus* Dejean, 1833: 53** (as “Thermonetus. *Eschscholtz*.”)

Originally included available species: *Dytiscus circumscriptus* Latreille, 1809.

Type species: *Dytiscus circumscriptus* Latreille, 1809 by monotypy.

Current status: valid genus in Dytiscidae (*fide* Nilsson et al. 1989: 308).

***Trigonocheilus* Dejean, 1833: 59**

Originally included available species: none.

***Trochalus* Dejean, 1833: 53** (as “Trochalus. *Eschscholtz*.”)

Originally included available species: *Dytiscus aciculatus* Herbst, 1784 (as “*Aciculatus. Oliv*.”); *Dytiscus costalis* Fabricius, 1775 (as “Costalis. *Oliv*.”); *Dytiscus dispar* Rossi, 1790 (as “*Dispar. Sturm*.”); *Dytiscus fimbriolatus* Say, 1823; *Dytiscus immarginatus* Fabricius, 1794; *Dytiscus laevigatus* Olivier, 1795 (as “Laevigatus. *Fabr*.”); *Dytiscus lateralis* Fabricius, 1798; *Dytiscus limbatus* Fabricius, 1775; *Dytiscus roeselii* Füessly, 1775 (as “Roeselii. *Fabr*.”).

Type species: *Dytiscus roeselii* Füessly, 1775 (= *Dytiscus lateralimarginalis* DeGeer, 1774) by subsequent designation (Nilsson et al. 1989: 308).

Current status: junior homonym of *Trochalus* Laporte, 1832 [Scarabaeidae]; senior objective synonym of *Scaphinectes* Ádám, 1993 in Dytiscidae (*fide* Nilsson 2003: 51).

### Pentamères: Brachélytres

***Astenus* Dejean, 1833: 65**

Originally included available species: *Staphylinus angustatus* Paykull, 1789 (as “Angustatus. *Fabr*.”); *Paederus extensus* Mannerheim, 1830 (as “*Extensus. Gyllenhal*.”); *Paederus filiformis* Latreille, 1806 (as “*Filiformis. Dahl*.”); *Paederus procerus* Gravenhorst, 1806 (as “Procerus. *Knoch*.”).

Type species: *Staphylinus angustatus* Paykull, 1789 (= *Staphylinus gracilis* Paykull, 1789) by subsequent designation (Westwood 1838: 17).

Current status: valid genus in Staphylinidae (*fide* Smetana 2004a: 579).

***Callictenus* Dejean, 1833: 59**

Originally included available species: none.

***Corynocerus* Dejean, 1833: 68**

Originally included available species: none.

***Lithocharis* Dejean, 1833: 65**

Originally included available species: *Paederus bicolor* Olivier, 1795 (as “Bicolor. *Grav*.”); *Paederus ochraceus* Gravenhorst, 1802; *Paederus rubricollis* Gravenhorst, 1806 (as “*Rubricollis. Gyllenhal*.”).

Type species: *Paederus ochraceus* Gravenhorst, 1802 by subsequent designation (Thomson 1859: 28).

Current status: valid genus in Staphylinidae (*fide* Smetana 2004a: 605).

***Lyeidius* Dejean, 1833: 64** (as “Lyeidius. *Leach*.”)

Originally included available species: none.

***Macrostenus* Dejean, 1833: 64**

Originally included available species: none.

***Megalops* Dejean, 1833: 66**

Originally included available species: none.

***Microphius* Dejean, 1833: 65**

Originally included available species: none.

***Microsaurus* Dejean, 1833: 61**

Originally included available species: *Staphylinus attenuatus* Gravenhorst, 1802; *Staphylinus boops* Gravenhorst, 1802; *Staphylinus fuliginosus* Gravenhorst, 1802; *Staphylinus impressus* Panzer, 1796 (as “Impressus. *Grav*.”); *Staphylinus laevigatus* Gyllenhal, 1810; *Staphylinus lateralis* Gravenhorst, 1802; *Staphylinus maurorufus* Gravenhorst, 1806 (as “Maurorufus. *Gyllenhal*.”); *Staphylinus molochinus* Gravenhorst, 1806; *Staphylinus nitidus* Fabricius, 1787; *Staphylinus ochripennis* Ménétriés, 1832; *Staphylinus praecox* Gravenhorst, 1802; *Staphylinus scintillans* Gravenhorst, 1806; *Staphylinus scitus* Gravenhorst, 1806; *Staphylinus subuliformis* Gravenhorst, 1802 (as “Subuliformis. *Gyllenhal*.”); *Staphylinus variabilis* Gyllenhal, 1810.

Type species: *Staphylinus ochripennis* Ménétriés, 1832 (Opinion 2115 in ICZN 2005).

Current status: valid subgenus of *Quedius* Stephens, 1829 in Staphylinidae (*fide* Smetana 2004b: 657).

***Olisthaerus* Dejean, 1833: 69**

Originally included available species: *Staphylinus substriatus* Paykull, 1790 (as “Substriatus. *Gyllenhal*.”).

Type species: *Staphylinus substriatus* Paykull, 1790 by monotypy.

Current status: valid genus in Staphylinidae (*fide* Herman 2001b: 659).

***Ophiomorphus* Dejean, 1833: 64**

Originally included available species: none.

***Phloeobium* Dejean, 1833: 69**

Originally included available species: *Staphylinus depressus* Paykull, 1789 (as “Depressum. *Gyllenhal*.).

Type species: *Staphylinus depressus* Paykull, 1789 by monotypy.

Current status: junior objective synonym of *Megarthrus* Stephens, 1829 in Staphylinidae (*fide* Herman 2001a: 605).

***Platytoma* Dejean, 1833: 59**

Originally included available species: none.

***Plochionocerus* Dejean, 1833: 64**

Originally included available species: *Staphylinus violaceus* Olivier, 1795.

Type species: *Staphylinus violaceus* Olivier, 1795 by monotypy.

Current status: valid genus in Staphylinidae (*fide* Herman 2001c: 3743).

***Sauromorphus* Dejean, 1833: 59**

Originally included available species: none.

### Pentamères: Sternoxes

***Abrobapta*** **Dejean, 1833: 80**

Originally included available species: none.

***Actenodes* Dejean, 1833: 80**

Originally included available species: *Buprestis nobilis* Fabricius, 1787.

Type species: *Buprestis nobilis* Linnaeus, 1758 (Opinion 2008 in ICZN 2002: 212).

Current status: valid genus in Buprestidae (*fide* Bellamy 2008c: 1560).

Comments. Linnaeus (1758: 410) described *Buprestis nobilis* from “Indiis.” Fabricius (1787: 180) described a new species under the name *Buprestis nobilis* and gave the provenance as “Cajennae.” It is quite obvious that Dejean (1833: 80) had Fabricius’ species in mind since “Cayennae” is listed as the provenance for his specimens. Fabricius’ species is currently included in the genus *Chrysobothris* Eschscholtz, 1829 (Bellamy 2008c: 1657) while Linnaeus’ species is actually included in the genus *Actenodes* Dejean, 1833 and recorded only from the Neotropical Region, including French Guiana (Bellamy 2008c: 1569). As far as we know, Saunders (1871: 93) is the first author to have listed, without comments, *nobilis* Linnaeus from Cayenne and ever since Linnaeus’ species is recorded from the Neotropical Region only. Nobody seems to have studied the type material of *Buprestis nobilis* Linnaeus. There is no *Actenodes* known from India or from Eurasia which leads to speculate that *nobilis* Linnaeus, unless the provenance given by Linnaeus is incorrect, is probably not an *Actenodes*. Nevertheless, the ICZN (2002: 212) ruled that *Buprestis nobilis* Linnaeus, 1758, a species not Originally included in Dejean (1833: 80), is the type species of *Actenodes* Dejean.

***Ampedus*** **Dejean, 1833: 92** (as “Ampedus. *Megerle*.”)

Originally included available species: *Elater auritus* Herbst, 1806; *Elater balteatus* Linnaeus, 1758 (as “Balteatus. *Fabr*.”); *Elater carbonicolor* Eschscholtz, 1829; *Elater collaris* Say, 1825; *Elater elongatulus* Fabricius, 1787; *Elater ephippium* Olivier, 1790 (as “Ephippium. *Fabr*.”); *Elater erythrogonus* Müller, 1821; *Elater nigricollis* Say, 1823; *Elater nigrinus* Herbst, 1784 (as “Nigrinus. *Gyllenhal*.”); *Elater praeustus* Fabricius, 1792; *Elater sanguineus* Linnaeus, 1758 (as “Sanguineus. *Fabr*.”); *Elater sanguinicollis* Panzer, 1793; *Elater sanguinipennis* Say, 1823; *Elater semiruber* Stephens, 1830 (as “*Semiruber. Leach*.”); *Elater tristis* Linnaeus, 1758 (as “Tristis. *Fabr*.”); *Elater verticinus* Palisot de Beauvois, 1819.

Type species: *Elater sanguineus* Linnaeus, 1758 by subsequent designation (Curtis 1838: Pl. 694).

Current status: valid genus in Elateridae (*fide* Cate 2007: 120).

***Analampis*** **Dejean, 1833: 79**

Originally included available species: none.

***Brachys* Dejean, 1833: 83**

Originally included available species: *Trachys tesselatus* Fabricius, 1801.

Type species: *Trachys tesselatus* Fabricius, 1801 by monotypy.

Current status: valid genus in Buprestidae (*fide* Bellamy 2008d: 2571).

***Callimicra* Dejean, 1833: 83**

Originally included available species: none.

***Cardiotarsus*** **Dejean, 1833: 91** (as “Cardiotarsus. *Eschscholtz*.”)

Originally included available species: none.

***Catoxantha* Dejean, 1833: 75**

Originally included available species: *Buprestis bicolor* Fabricius, 1775; *Buprestis heros* Wiedemann, 1823.

Type species: *Buprestis bicolor* Fabricius, 1775 by monotypy.

Current status: senior synonym of *Megaloxantha* Kerremans, 1902 in Buprestidae (*fide* Bellamy 2008a: 446).

Comments. The name *heros* is listed in synonymy with *bicolor* in Dejean’s catalogue; therefore the type species of *Catoxantha* is *bicolor* by monotypy (ICZN 1999: Article 68.3).

*Catoxantha* Solier, 1833 [type species: *Buprestis opulenta* Gory, 1832] is currently considered a valid genus and is treated as a senior homonym of *Catoxantha* Dejean, 1833 (e.g., Bellamy 2008a: 446). However Dejean’s name is older than Solier’s name (see “Precedence” section[1]). Usage of *Catoxantha* Dejean, 1833 as valid would imply nomenclatural changes: *Megaloxantha* Kerremans, 1902 would become a junior synonym of *Catoxantha* Dejean and *Catoxantha* Solier would be replaced by *Epacmene* Gistel, 1848. A request to the Commission to suppress *Catoxantha* Dejean, 1833 is necessary to promote nomenclatural stability.

***Chalcophora* Dejean, 1833: 77** (as “Chalcophora. *Serville*.”)

Originally included available species: *Buprestis detrita* Klug, 1829; *Buprestis fabricii* Rossi, 1794; *Buprestis mariana* Linnaeus, 1758 (as “Mariana. *Fabr*.”); *Buprestis quadrinotata* Klug, 1829; *Buprestis stigmatica* Dalman, 1817 (as “Stigmatica. *Schönherr*.”); *Buprestis virginiensis* Drury, 1773 (as “Virginiensis. *Herbst*.”).

Type species: *Buprestis mariana* Linnaeus, 1758 by subsequent designation (Duponchel 1843: 372).

Current status: valid genus in Buprestidae (*fide* Bellamy 2008a: 558).

***Chrysesthes* Dejean, 1833: 78** (as “Chrysesthes. *Serville*.”)

Originally included available species: *Buprestis angularis* Dalman, 1817 (as “Angularis. *Schönherr*.”); *Buprestis tripunctata* Fabricius, 1787.

Type species: *Buprestis tripunctata* Fabricius, 1787 by subsequent designation (Drapiez 1837: 412).

Current status: valid genus in Buprestidae (*fide* Bellamy 2008a: 588).

***Chrysochroa* Dejean, 1833: 75** (as “Chrysochroa. *Carcel*.”)

Originally included available species: *Buprestis fulgida* Olivier, 1790 (as “Fulgida. *Fabr*.”); *Buprestis fulminans* Fabricius, 1787; *Buprestis ignita* Linnaeus, 1758 (as “Ignita. *Fabr*.”); *Buprestis mutabilis* Olivier, 1790; *Buprestis ocellata* Fabricius, 1775; *Buprestis vittata* Fabricius, 1775.

Type species: *Buprestis fulminans* Fabricius, 1787 by subsequent designation (Kurosawa 1982: 185).

Current status: valid genus in Buprestidae (*fide* Bellamy 2008a: 429).

***Cratonychus*** **Dejean, 1833: 87**

Comments. This name is treated as an unnecessary replacement name for *Melanotus* Eschscholtz, 1829 [Elateridae].

***Ctenonychus*** **Dejean, 1833: 87**

Originally included available species: none.

Comments. Dejean’s name is considered to be different from *Ctenonychus* Stephens, 1830 also proposed in Elateridae.

***Cylindroderus*** **Dejean, 1833: 94** (as “Cylindroderus. *Eschscholtz*.”)

Originally included available species: none.

***Cyphonota* Dejean, 1833: 79**

Originally included available species: *Buprestis sibirica* Fabricius, 1781; *Buprestis tartarica* Pallas, 1773.

Type species: *Buprestis sibirica* Fabricius, 1781 (= *Buprestis tartarica* Pallas, 1773) by monotypy.

Current status: name suppressed in Buprestidae.

Comments. The name *tartarica* is listed in synonymy with *sibirica* in Dejean’s catalogue; therefore the type species of *Cyphonota* is *sibirica* by monotypy (ICZN 1999: Article 68.3).

*Cyphonota* Dejean was suppressed for the purposes of the Principle of Priority in Opinion 2083 (ICZN 2004).

***Cyria* Dejean, 1833: 75** (as “Cyria. *Serville*.”)

Originally included available species: *Buprestis imperialis* Fabricius, 1801.

Type species: *Buprestis imperialis* Fabricius, 1801 by monotypy.

Current status: junior homonym of *Cyria* Leach, 1818 [Mollusca]; senior subjective synonym of *Cyrioides* Carter, 1920 in Buprestidae (*fide* Bellamy 2008b: 1017).

***Diphucrania* Dejean, 1833: 81**

Originally included available species: *Buprestis leucosticta* Kirby, 1819.

Type species: *Buprestis leucosticta* Kirby, 1819 by monotypy.

Current status: valid genus in Buprestidae (*fide* Bellamy 2008e: 3262).

Comments. The ICZN (2008: 325) voted against the suppression of *Diphucrania* Dejean, 1833 in Opinion 2214 to maintain *Cisseis* Gory and Laporte, 1839. Therefore *Diphucrania* Dejean has precedence over *Cisseis* Gory and Laporte.

***Dirhagus*** **Dejean, 1833: 84** (as “Dirhagus. *Eschscholtz*.”)

Originally included available species: none.

***Euchroma* Dejean, 1833: 76** (as “Euchroma. *Serville*.”)

Originally included available species: *Buprestis gigantea* Linnaeus, 1758 (as “Gigantea. *Fabr*.”).

Type species: *Buprestis gigantea* Linnaeus, 1758 by monotypy.

Current status: valid genus in Buprestidae (*fide* Bellamy 2008a: 572).

***Eurhipis*** **Dejean, 1833: 85**

Originally included available species: none.

***Eurythyrea* Dejean, 1833: 78** (as “Eurythyrea. *Serville*.”)

Originally included available species: *Buprestis austriaca* Linnaeus, 1767 (as “Austriaca. *Fabr*.”); *Buprestis marginata* Olivier, 1790; *Buprestis micans* Fabricius, 1792; *Buprestis quercus* Herbst, 1780; *Buprestis scutellaris* Olivier, 1790; *Buprestis similis* Schönherr, 1817.

Type species: *Buprestis austriaca* Linnaeus, 1767 by subsequent designation (Duponchel 1845: 525).

Current status: valid genus in Buprestidae (*fide* Bellamy 2008b: 1076).

***Evides* Dejean, 1833: 77** (as “Evides. *Serville*.”)

Originally included available species: *Buprestis elegans* Fabricius, 1781; *Buprestis satrapa* Schönherr, 1817; *Buprestis smaragdula* Olivier, 1790 (as “Smaragdula. *Fabr*.”); *Buprestis suturalis* Fabricius, 1801; *Buprestis ventricosa* Olivier, 1790.

Type species: *Buprestis elegans* Fabricius, 1781 by subsequent designation (Bellamy 1997: 370).

Current status: valid genus in Buprestidae (*fide* Bellamy 2008a: 569).

***Geronia*** **Dejean, 1833: 79**

Originally included available species: none.

***Hemiops*** **Dejean, 1833: 95** (as “Hemiops. *Eschscholtz*.”)

Originally included available species: none.

***Hypocaelus*** **Dejean, 1833: 85** (as “Hypocaelus. *Eschscholtz*.”)

Originally included available species: *Elater filum* Fabricius, 1801.

Type species: *Elater filum* Fabricius, 1801 by monotypy.

Current status: junior objective synonym of *Nematodes* Berthold, 1827 in Eucnemidae (*fide* Muona 2007: 87).

Comments. The name “Buprestoides. *Rossi*.” listed from “Italia” by Dejean (1833: 85) cannot be interpreted and is considered a *nomen nudum*.

***Lampetis* Dejean, 1833: 76**

Originally included available species: *Buprestis bioculata* Olivier, 1790; *Buprestis catenulata* Klug, 1829; *Buprestis fastuosa* Fabricius, 1775; *Buprestis mimosae* Klug, 1829.

Type species: *Buprestis bioculata* Olivier, 1790 by subsequent designation (Lacordaire 1857: 30).

Current status: valid genus in Buprestidae (*fide* Bellamy 2008b: 892).

***Lampra* Dejean, 1833: 78** (as “Lampra. *Megerle*.”)

Originally included available species: *Buprestis conspersa* Gyllenhal, 1801; *Buprestis festiva* Linnaeus, 1767 (as “Festiva. *Fabr*.”); *Buprestis plebeja* Herbst, 1801; *Buprestis rutilans* Fabricius, 1777; *Buprestis variolosa* Paykull, 1799.

Type species: *Buprestis rutilans* Fabricius, 1777 (Opinion 1825 in ICZN 1996b).

Current status: junior homonym of *Lampra* Hübner, 1821 [Lepidoptera]; senior objective synonym of *Lamprodila* Motschulsky, 1860 in Buprestidae (*fide* Bellamy 2008a: 600).

Comments. As pointed out by Mühle (1993: 28), the first valid type species designation for *Lampra* Dejean is that of Casey (1909: 52) who designated *Buprestis festiva* Linnaeus, 1767. However, the International Commission on Zoological Nomenclature (ICZN 1996b) ruled in Opinion 1825 that the type species of *Scintillatrix* Obenberger, 1956 is *Buprestis rutilans* Fabricius, 1777. Since *Scintillatrix* Obenberger is a replacement name for *Lampra* Dejean, both must have the same type species (ICZN 1999: Article 67.8).

***Lasionota* Dejean, 1833: 83**

Originally included available species: none.

***Leptia* Dejean, 1833: 78**

Originally included available species: none.

***Lius* Dejean, 1833: 83** (as “Lius. *Eschscholtz*.”)

Originally included available species: none.

***Macrodes*** **Dejean, 1833: 94**

Originally included available species: none.

***Megacnemius*** **Dejean, 1833: 94** (as “Megacnemius. *Eschscholtz*.”)

Originally included available species: none.

***Melanoxanthus*Dejean, 1833: 91** (as “Melanoxanthus. *Eschscholtz*.”)

Originally included available species: *Elater melanocephalus* Fabricius, 1781.

Type species: *Elater melanocephalus* Fabricius, 1781 by monotypy.

Current status: valid genus in Elateridae (*fide* Cate 2007: 137, as “*Melanoxanthus* Eschscholtz, 1838”).

***Microrhagus*** **Dejean, 1833: 85** (as “Microrhagus. *Eschscholtz*.”)

Originally included available species: *Elater pygmaeus* Fabricius, 1792; *Eucnemis sahlbergi* Mannerheim, 1823.

Type species: *Elater pygmaeus* Fabricius, 1792 by subsequent designation (Westwood 1838: 25).

Current status: valid genus in Eucnemidae (*fide* Muona 2007: 82).

***Oomorpha* Dejean, 1833: 83**

Originally included available species: none.

***Oophorus*** **Dejean, 1833: 93** (as “Oophorus. *Eschscholtz*.”)

Originally included available species: *Elater dilectus* Say, 1825; *Elater dorsalis* Say, 1823; *Elater elegans* Fabricius, 1792.

Type species: *Elater elegans* Fabricius, 1792 by subsequent designation (Hyslop 1921: 659).

Current status: junior subjective synonym of *Aeolus* Eschscholtz, 1829 (*fide* Cate 2007: 104).

***Oxycleidius*** **Dejean, 1833: 89** (as “Oxycleidius. *Eschscholtz*.”)

Originally included available species: none.

***Perothops*** **Dejean, 1833: 87** (as “Perothops. *Eschscholtz*.”)

Originally included available species: none.

***Perotis* Dejean, 1833: 77** (as “Perotis. *Megerle*.”)

Originally included available species: *Buprestis lugubris* Fabricius, 1777; *Buprestis unicolor* Olivier, 1790.

Type species: *Buprestis lugubris* Fabricius, 1777 by subsequent designation (Bellamy 1997: 370).

Current status: valid genus in Buprestidae (*fide* Bellamy 2008b: 964).

***Phaenops* Dejean, 1833: 79** (as “Phaenops. *Megerle*.”)

Originally included available species: *Buprestis appendiculata* Fabricius, 1792; *Buprestis clypeata* Paykull, 1799; *Buprestis cyanea* Fabricius, 1775; *Buprestis decostigma* Fabricius, 1787; *Buprestis guttulata* Gebler, 1830; *Buprestis morio* Fabricius *sensu* Paykull, 1799; *Buprestis quatuordecimguttata* Olivier, 1790; *Buprestis tarda* Fabricius, 1792.

Type species: *Buprestis cyanea* Fabricius, 1775 by subsequent designation (Théry 1942: 73) (see Opinion 1826 in ICZN 1996c).

Current status: valid genus in Buprestidae (*fide* Bellamy 2008c: 1535).

***Physorhinus*** **Dejean, 1833: 86** (as “Physorhinus. *Eschscholtz*.”)

Originally included available species: none.

***Polybothris* Dejean, 1833: 78**

Originally included available species: none.

***Polycesta* Dejean, 1833: 78** (as “Polycesta. *Serville*.”)

Originally included available species: *Buprestis depressa* Linnaeus, 1771 (as “*Depressa. Oliv*.”); *Buprestis porcata* Fabricius, 1775.

Type species: *Buprestis porcata* Fabricius, 1775 by monotypy.

Current status: valid genus in Buprestidae (*fide* Bellamy 2008a: 365).

Comments. The name *depressa* is listed in synonymy with *porcata* in Dejean’s catalogue; therefore the type species of *Polycesta* is *porcata* by monotypy (ICZN 1999: Article 68.3).

***Polychroma* Dejean, 1833: 79**

Originally included available species: *Buprestis crenata* Donovan, 1805; *Buprestis decemmaculata* Kirby, 1818; *Buprestis rufipennis* Kirby, 1818; *Buprestis undulata* Donovan, 1805.

Type species: none found.

Current status: name suppressed in Buprestidae.

Comments. Dejean’s name, as *Polychroma* Dejean, 1836, was suppressed for the purposes of the Principle of Priority in Opinion 1628 (ICZN 1991).

***Prionophora* Dejean, 1833: 78**

Originally included available species: none.

***Pristiptera* Dejean, 1833: 78**

Originally included available species: *Buprestis blanda* Fabricius, 1781.

Type species: *Buprestis blanda* Fabricius, 1781 by monotypy.

Current status: senior subjective synonym of *Pelecopselaphus* Solier, 1833 in Buprestidae (*fide* Bellamy 2008a: 586).

Comments. *Pelecopselaphus* Solier, 1833 [type species: *Buprestis angularis* Schönherr, 1817] is currently considered a valid genus and treated as a senior synonym of *Pristiptera* Dejean, 1833 (e.g., Bellamy 2008a: 586). However Dejean’s name is older than Solier’s name (see “Precedence” section[1]). An application to the Commission is needed to conserve *Pelecopselaphus* Solier, 1833 as the valid name.

***Psiloptera* Dejean, 1833: 76** (as “Psiloptera. *Serville*. ”)

Originally included available species: *Buprestis attenuata* Fabricius, 1792; *Buprestis bilineata* Latreille, 1813; *Buprestis collaris* Olivier, 1790 (as “Collaris. *Fabr*.”); *Buprestis cuproaenea* Latreille, 1813 (as “Cupreoaenea. *Latreille*.”); *Buprestis equestris* Olivier, 1790; *Buprestis fulgida* Olivier, 1790; *Buprestis hirtomaculata* Herbst, 1801; *Buprestis viridiaurea* Schönherr, 1817.

Type species: *Buprestis attenuata* Fabricius, 1792 by subsequent designation (Thomson 1878: 29).

Current status: valid genus in Buprestidae (*fide* Bellamy 2008b: 838).

***Pterotarsus* Dejean, 1833: 84** (as “Pterotarsus. *Latreille*.”)

Originally included available species: none.

***Ptosima* Dejean, 1833: 79** (as “Ptosima. *Serville*.”)

Originally included available species: *Buprestis novemmaculata* Fabricius, 1775.

Type species: *Buprestis novemmaculata* Fabricius, 1775 by monotypy.

Current status: valid genus in Buprestidae (*fide* Bellamy 2008a: 326).

***Rhigmaphorus*** **Dejean, 1833: 84**

Originally included available species: none.

***Selagis* Dejean, 1833: 79**

Originally included available species: none.

***Sericosomus*** **Dejean, 1833: 96** (as “Sericosomus. *Serville*.”)

Comments. This name is treated as an unnecessary replacement name for *Sericus* Eschscholtz, 1829 [Elateridae].

***Sphaerocephalus*** **Dejean, 1833: 85** (as “Sphaerocephalus. *Eschscholtz*.”)

Originally included available species: none.

***Sphenoptera*** **Dejean, 1833: 81**

Originally included available species: *Buprestis antiqua* Illiger, 1803; *Buprestis dejeanii* Zoubkoff, 1829; *Buprestis dianthi* Stéven, 1829; *Buprestis fossulata* Gebler, 1824; *Buprestis geminata* Illiger, 1803; *Buprestis glabrata* Ménétriés, 1832; *Buprestis lineata* Fabricius, 1775; *Buprestis metallica* Fabricius, 1792; *Buprestis meyeri* Gebler, 1830; *Buprestis pulverulenta* Herbst, 1801; *Buprestis tricuspidata* Olivier, 1790 (as “Tricuspidata. *Schönherr*.”); *Buprestis trispinosa* Klug, 1829.

Type species: *Buprestis antiqua* Illiger, 1803 by subsequent designation (Volkovitsh and Kalashian 2002: 166).

Current status: valid genus in Buprestidae (*fide* Bellamy 2008b: 635).

***Steatoderus*** **Dejean, 1833: 94** (as “Steatoderus. *Eschscholtz*.”)

Originally included available species: *Elater ferrugineus* Linnaeus, 1758 (as “Ferrugineus. *Fabr*.”).

Type species: *Elater ferrugineus* Linnaeus, 1758 by monotypy.

Current status: junior objective synonym of *Elater* Linnaeus, 1758 in Elateridae (*fide* Cate 2007: 131).

***Steraspis* Dejean, 1833: 75**

Originally included available species: *Buprestis scabra* Fabricius, 1775; *Buprestis speciosa* Klug, 1829; *Buprestis squamosa* Klug, 1829.

Type species: *Buprestis scabra* Fabricius, 1775 by subsequent designation (Bellamy 1997: 369).

Current status: valid genus in Buprestidae (*fide* Bellamy 2008a: 461).

***Strigoptera* Dejean, 1833: 78**

Originally included available species: *Buprestis bimaculata* Linnaeus, 1758 (as “Bimaculata. *Fabr*.”); *Buprestis bivittata* Fabricius, 1801.

Type species: *Buprestis bimaculata* Linnaeus, 1758 by subsequent designation (Cobos 1981: 32).

Current status: valid genus in Buprestidae (*fide* Bellamy 2008a: 379).

***Xyloecus*** **Dejean, 1833: 85** (as “Xyloecus. *Serville*.”)

Comments. This name is treated as an unnecessary replacement name for *Xylophilus* Mannerheim, 1823 [Eucnemidae].

### Pentamères: Malacodermes

***Actenista* Dejean, 1833: 101**

Originally included available species: none.

***Anisocera* Dejean, 1833: 105**

Originally included available species: none.

***Atela* Dejean, 1833: 100**

Originally included available species: none.

***Auge* Dejean, 1833: 100**

Originally included available species: none.

***Calendyma* Dejean, 1833: 111**

Originally included available species: none.

***Callianthia* Dejean, 1833: 104**

Originally included available species: *Cantharis bimaculata* Fabricius, 1781; *Telephorus fallax* Germar, 1824 (as “Fallax. *Illiger*.”); *Telephorus luctuosus* Latreille, 1809; *Cantharis marginata* Fabricius, 1775; *Telephorus pulchellus* MacLeay, 1826; *Cantharis schüppelii* Klug, 1829 (as “Schüppelii. *Dej*.”); *Telephorus scriptus* Germar, 1824; *Cantharis varians* Klug, 1829 (as “Varians. *Dej*.”).

Type species: *Cantharis marginata* Fabricius, 1775 by subsequent designation (Hope 1840: 141).

Current status: junior synonym of *Chauliognathus* Hentz, 1830 in Cantharidae (*fide* Ramsdale 2002: 214).

***Charactus* Dejean, 1833: 98**

Originally included available species: *Lycus atratus* Fabricius, 1801; *Cantharis bicolor* Linnaeus, 1763 (as “Bicolor. *Fabr*.”); *Lycus cinctus* Fabricius, 1801; *Pyrochroa fasciata* Fabricius, 1787; *Lycus flabellatus* Dalman, 1817 (as “Flabellatus. *Schönherr*.”); *Lycus limbatus* Fabricius, 1801; *Lycus nigricornis* Latreille, 1817; *Pyrochroa reticulata* Fabricius, 1775; *Lycus suturalis* Latreille, 1813; *Lycus terminalis* Say, 1823; *Lycus terminatus* Latreille, 1813; *Lycus tricolor* Olivier, 1790 (as “Tricolor. *Fabr*.”).

Type species: *Lycus limbatus* Fabricius, 1801 by **present designation**.

Current status: senior objective synonym of *Calopteron* Laporte, 1836 in Lycidae (*fide* Bocák 1998: 249, as “*Calopteron* Castelnau, 1838”).

Comments. *Charactus* Dejean, 1833 has precedence over *Calopteron* Laporte, 1836 which is currently used as valid (e.g., Bocák 1998: 249, as “*Calopteron* Castelnau, 1838”). Reversal of Precedence (ICZN 1999: Article 23.9) or an application to the Commission is necessary to conserve usage of the name *Calopteron* Laporte. The genus *Calopteron* was first proposed by Laporte (1836: 25) for “*Lycus limbatus*, *fasciatus*, *tricolor*, *bicolor*… de Fabricius.” Its type species is *Lycus limbatus* Fabricius, 1801 by subsequent designation of Bocák (1998: 249).

***Cladon* Dejean, 1833: 97**

Originally included available species: none.

***Colophotia* Dejean, 1833: 103**

Originally included available species: *Lampyris australis* Fabricius, 1775; *Lampyris capensis* Fabricius, 1775; *Cantharis italica* Linnaeus, 1758 (as “Italica. *Fabr*.”); *Lampyris japonica* Thunberg, 1784 (as “Japonica. *Fabr*.”); *Lampyris mingrelica* Ménétriés, 1832 (as “*Mingrelica. Mannerheim*.”); *Lampyris praeusta* Eschscholtz, 1822; *Lampyris vespertina* Fabricius, 1801.

Type species: *Lampyris praeusta* Eschscholtz, 1822 by subsequent designation (Motschulsky 1853: 52).

Current status: valid genus in Lampyridae (*fide* Ballantyne and Lambkin 2000: 21).

***Ctenidion* Dejean, 1833: 104**

Originally included available species: none.

***Dadophora* Dejean, 1833: 100**

Originally included available species: none.

***Ellychnia* Dejean, 1833: 102**

Originally included available species: *Lampyris corrusca* Linnaeus, 1767 (as “Corrusca. *Fabr*.”); *Lampyris guttula* Fabricius, 1801; *Lampyris nigricans* Say, 1823.

Type species: *Lampyris corrusca* Linnaeus, 1767 by subsequent designation (Motschulsky 1853: 28).

Current status: valid genus in Lampyridae (*fide* Lloyd 2002: 193, as “*Ellychnia* Blanchard 1845”).

***Epicyrtus* Dejean, 1833: 97**

Originally included available species: none.

***Epiphyta* Dejean, 1833: 110**

Originally included available species: none.

***Eurycerus* Dejean, 1833: 100**

Originally included available species: *Homalisus platycerus* Wiedemann, 1821.

Type species: *Homalisus platycerus* Wiedemann, 1821 by monotypy.

Current status: unknown.

Comments: We have found no information on the type species. It is not listed as a member of the family Omalisidae based on Bocák and Brlik’s review (2008).

***Geopyris* Dejean, 1833: 103**

Originally included available species: *Lampyris hemiptera* Goeze, 1777 (as “Hemiptera. *Fabr*.”).

Type species: *Lampyris hemiptera* Goeze, 1777 by monotypy.

Current status: junior objective synonym of *Phosphaenus* Laporte, 1833 in Lampyridae (*fide* Geisthardt and Satô 2007: 230).

Comments. *Phosphaenus* Laporte, 1833 is older than *Geopyris* Dejean, 1833 (see “Precedence” section [2]).

***Lychnuris* Dejean, 1833: 101**

Originally included available species: *Lampyris atra* Olivier, 1790; *Lampyris bicolor* Fabricius, 1801; *Lampyris laticornis* Fabricius, 1792; *Lampyris savignii* Kirby, 1818 (as “Savignyi. *Kirby*.”).

Type species: *Lampyris bicolor* Fabricius, 1801 by subsequent designation (McDermot 1966: 14).

Current status: valid genus in Lampyridae (*fide* McDermot 1966: 14).

Comments. *Lychnuris* Dejean, 1833 was incorrectly treated as an invalid synonym of *Pyrocoelia* Gorham, 1880 by Geisthardt and Satô (2007: 228) even though Dejean’s name is older.

***Lygistopterus* Dejean, 1833: 98**

Originally included available species: *Lycus dichrous* Klug, 1829; *Cantharis sanguineus* Linnaeus, 1758 (as “Sanguineus. *Fabr*.”); *Lycus succinctus* Latreille, 1809.

Type species: *Cantharis sanguineus* Linnaeus, 1758 by subsequent designation (Chevrolat 1846: 515).

Current status: valid genus in Lycidae (*fide* Miller 2002: 177).

***Nematophora* Dejean, 1833: 101**

Originally included available species: none.

***Nyctocharis* Dejean, 1833: 100**

Originally included available species: none.

***Nyctophanes* Dejean, 1833: 101**

Originally included available species: *Cantharis ignita* Linnaeus, 1758 (as “Ignita. *Fabr*.”); *Lampyris lineata* Gyllenhal, 1817 (as “Lineata. *Schönherr*.”); *Lampyris maculata* Olivier, 1790 (as “Maculata. *Fabr*.”); *Lampyris pallida* Olivier, 1790; *Lampyris scintillans* Latreille, 1813.

Type species: *Lampyris lineata* Gyllenhal, 1817 by subsequent designation (Motschulsky 1853: 34).

Current status: junior subjective synonym of *Aspisoma* Laporte, 1833 in Lampyridae (*fide* McDermot 1966: 29).

Comments. *Aspisoma* Laporte, 1833 is older than *Nyctophanes* Dejean, 1833 (see “Precedence” section [2]).

***Photuris* Dejean, 1833: 103**

Originally included available species: *Lampyris lunifera* Eschscholtz, 1822; *Lampyris versicolor* Fabricius, 1798.

Type species: *Lampyris versicolor* Fabricius, 1798 by subsequent designation (Barber 1951: 11).

Current status: valid genus in Lampyridae (*fide* Lloyd 2002: 193, as “*Photuris* LeConte 1851”).

***Podabrus* Dejean, 1833: 105** (as “Podabrus. *Fischer*.”)

Originally included available species: *Cantharis alpinus* Paykull, 1798; *Cantharis annulata* Mannerheim, 1824 (as “*Annulatus. Fischer*.”); *Cantharis diadema* Fabricius, 1798.

Type species: *Cantharis alpinus* Paykull, 1798 by subsequent designation (Stephens 1835: 416).

Current status: valid genus in Cantharidae (*fide* Kazantsev and Brancucci 2007: 237, as “*Podabrus* Westwood, 1838”).

***Pygolampis* Dejean, 1833: 102**

Originally included available species: *Lampyris albilatera* Gyllenhal, 1817 (as “Albilatera. *Schönherr*.”); *Lampyris discoidea* Sahlberg, 1823 (as “Discoidea. *Schönherr*.”); *Lampyris glauca* Olivier, 1790; *Lampyris linearis* Latreille, 1809; *Lampyris livida* Olivier, 1790; *Lampyris marginata* Linnaeus, 1767 (as “Marginata. *Fabr*.”); *Cantharis pyralis* Linnaeus, 1758 (as “Pyralis. *Fabr*.”); *Lampyris truncata* Eschscholtz, 1822; *Lampyris vittigera* Gyllenhal, 1817 (as “Vittigera. *Schönherr*.”).

Type species: *Lampyris glauca* Olivier, 1790 by **present designation**.

Current status: junior homonym of *Pygolampis* Germar, 1824 [Hemiptera] and *Pygolampis* Kirby and Spence, 1828 [Lampyridae]; senior subjective synonym of *Robopus* Motschulsky, 1853 in Lampyridae (*fide* McDermot 1966: 50).

Comments. This generic name is not the same as *Pygolampis* Kirby and Spence, 1828, also in the family Lampyridae.

***Pyractomena* Dejean, 1833: 102**

Originally included available species: none.

***Rabdota* Dejean, 1833: 100**

Originally included available species: none.

***Selas* Dejean, 1833: 100**

Originally included available species: *Lampyris latreillei* Kirby, 1818.

Type species: *Lampyris latreillei* Kirby, 1818 by monotypy.

Current status: junior objective synonym of *Lamprocera* Laporte, 1833 in Lampyridae (*fide* McDermot 1966: 23).

Comments. The name *Lamprocera* Laporte, 1833 is older than *Selas* Dejean, 1833 (see “Precedence” section [2]).

***Spenthera* Dejean, 1833: 101**

Originally included available species: none.

***Xanthestha* Dejean, 1833: 105**

Originally included available species: *Cantharis pectoralis* Fabricius, 1801.

Type species: *Cantharis pectoralis* Fabricius, 1801 by monotypy.

Current status: junior subjective synonym of *Cordylocera* Guérin-Méneville, 1830 in Cantharidae (*fide* Delkeskamp 1977: 218).

### Pentamères: Terediles

***Aegialites*** **Dejean, 1833: 117** (as “Aegialites. *Eschscholtz*.”)

Originally included available species: none.

***Callitheres* Dejean, 1833: 112** (as “Callitheres. *Latreille*.”)

Originally included available species: none.

***Epiphloeus*** **Dejean, 1833: 113**

Originally included available species: none.

***Notostenus* Dejean, 1833: 113**

Originally included available species: *Anobium viride* Thunberg, 1781.

Type species: *Anobium viride* Thunberg, 1781 by monotypy.

Current status: valid genus in Cleridae (*fide* Opitz 2011: 52).

***Phyllobaenus* Dejean, 1833: 113**

Originally included available species: *Clerus humeralis* Germar, 1824.

Type species: *Clerus humeralis* Germar, 1824 (= *Clerus humeralis* Say, 1823) by monotypy.

Current status: valid genus in Cleridae (*fide* Opitz 2002: 276).

***Stemmoderus*** **Dejean, 1833: 114**

Originally included available species: none.

***Xystrophorus*** **Dejean, 1833: 115**

Originally included available species: none.

### Pentamères: Clavicornes

***Cylistus*** **Dejean, 1833: 129** (as Cylistus. *Godet*.)

Originally included available species: *Hister cylindricus* Paykull, 1811.

Type species: *Hister cylindricus* Paykull, 1811 by monotypy.

Current status: valid subgenus of *Platysoma* Leach, 1817 in Histeridae (*fide* Mazur 2011: 64).

***Dermophagus* Dejean, 1833: 125**

Originally included available species: none.

***Encaustes* Dejean, 1833: 122**

Originally included available species: none.

***Episcapha* Dejean, 1833: 123**

Originally included available species: *Ips fasciata* Fabricius, 1777; *Engis glabra* Wiedemann, 1823; *Ips grandis* Fabricius, 1792; *Engis heros* Say, 1823; *Erotylus quadripustulatus* Fabricius, 1801.

Type species: none validly designated.

Current status: valid genus in Erotylidae (*fide* Wegrzynowicz 2007b: 539).

Comments. The type species of *Episcapha* cited by Chûjô (1969: 95) and Wegrzynowicz (2007b: 539), *Engis quadrimacula* Wiedemann, 1823, is not an originally included species. No type species is designated here since all available species listed by Dejean are currently included in other genera or in subgenera of *Episcapha* other than the nominotypical one. In order to preserve stability in the nomenclature of the genus, an application to the Commission is needed to retain *Engis quadrimacula* Wiedemann, 1823 as type species even though the species is not an originally included species.

***Haeterius* Dejean, 1833: 128** (as Haeterius. *Godet*.)

Originally included available species: *Hister ferrugineus* Olivier, 1789; *Hister quadratus* Kugelann, 1794 (as “Quadratus. *Paykull*.”).

Type species: *Hister quadratus* Kugelann, 1794 (= *Hister ferrugineus* Olivier, 1789) by monotypy.

Current status: valid genus in Histeridae (*fide* Mazur 2011: 125).

Comments. The name *ferrugineus* is listed in synonymy with *quadratus* in Dejean’s catalogue; therefore the type species of *Haeterius* is *quadratus* by monotypy (ICZN 1999: Article 68.3).

***Hyporhagus* Dejean, 1833: 129**

Originally included available species: *Tritoma marginata* Fabricius, 1792.

Type species: *Tritoma marginata* Fabricius, 1792 by monotypy.

Current status: valid genus in Zopheridae (*fide* Ivie 2002b: 455, as “*Hyporhagus* Thomson, 1860”).

***Lasioderma*** **Dejean, 1833: 119**

Originally included available species: none.

***Leionota* Dejean, 1833: 129**

Originally included available species: *Hololepta lamina* Paykull, 1811; *Hister quadridentatus* Olivier, 1789 (as “Quadridentata. *Fabr*.”).

Type species: *Hister quadridentatus* Olivier, 1789 by subsequent designation (Bickhardt 1916: 29).

Current status: valid subgenus of *Hololepta* Paykull, 1811 in Histeridae (*fide* Mazur 2011: 52, as “*Leionota* Marseul, 1853”).

***Monoplius* Dejean, 1833: 128**

Originally included available species: none.

***Omalodes* Dejean, 1833: 128**

Originally included available species: *Hister angulatus* Fabricius, 1801 (as “Angulatus. *Paykull*.”); *Hister laevigatus* Quensel, 1806 (as “*Laevigatus. Schönherr*.”); *Hister omega* Kirby, 1818 (as “*Omega. Mannerheim*.”).

Type species: *Hister omega* Kirby, 1818 by subsequent designation (Hope 1840: 105).

Current status: valid genus in Histeridae (*fide* Mazur 2011: 72).

***Oxysternus* Dejean, 1833: 129** (as Oxysternus. *Godet*.)

Originally included available species: *Hister maxillosus* Drury, 1782 (as “Maxillosus. *Fabr*.”).

Type species: *Hister maxillosus* Drury, 1782 (= *Hister maximus* Linnaeus, 1767) by monotypy.

Current status: valid genus in Histeridae (*fide* Mazur 2011: 53).

***Platyderus* Dejean, 1833: 125**

Originally included available species: none.

***Selenoderus* Dejean, 1833: 119**

Originally included available species: none.

***Thyreosoma* Dejean, 1833: 119**

Originally included available species: none.

### Pentamères: Palpicornes

***Cyclonotum* Dejean, 1833: 134**

Originally included available species: *Sphaeridium abdominale* Fabricius, 1792.

Type species: *Sphaeridium abdominale* Fabricius, 1792 by monotypy.

Current status: senior synonym of *Dactylosternum* Wollaston, 1854 in Hydrophilidae (**new synonymy**).

Comments. *Cyclonotum* is usually incorrectly credited to Erichson (1837a: 212), with *Hydrophilus orbicularis* Fabricius, 1775 as type species, and listed as a junior synonym of *Coelostoma* Brullé, 1835 (e.g., Hansen 1999: 242; Hansen 2004: 60). To promote nomenclatural stability a request to the Commission is necessary to suppress *Cyclonotum* Dejean, 1833 and conserve *Dactylosternum* Wollaston, 1854 as the valid name. Reversal of Precedence (ICZN 1999, Article 23.9) cannot be used to suppress *Cyclonotum* since the name was used as valid after 1899 (e.g., Régimbart 1906: 269).

### Pentamères: Lamellicornes

***Ablabera* Dejean, 1833: 159**

Originally included available species: *Melolontha lateralis* Wiedemann, 1821; *Melolontha splendida* Fabricius, 1781 (as “Splendida. *Illiger*.”).

Type species: *Melolontha splendida* Fabricius, 1781 by monotypy.

Current status: valid genus in Scarabaeidae (*fide* Ahrens 2006: 141).

Comments: The name *lateralis* is listed in synonymy with *splendida* in Dejean’s catalogue; therefore the type species of *Ablabera* is *splendida* by monotypy (ICZN 1999: Article 68.3).

***Acallus* Dejean, 1833: 149**

Originally included available species: none.

***Acerus* Dejean, 1833: 150**

Originally included available species: none.

***Adelops* Dejean, 1833: 164**

Originally included available species: none.

***Adoretus* Dejean, 1833: 157** (as “Adoretus. *Eschscholtz*.”)

Originally included available species: *Melolontha compressa* Weber, 1801; *Melolontha lanata* Fabricius, 1801; *Melolontha nigrifrons* Steven, 1809; *Melolontha obscura* Fabricius, 1781; *Melolontha senegallia* Dufour, 1821.

Type species: *Melolontha nigrifrons* Steven, 1809 by subsequent designation (Medvedev 1949: 313).

Current status: valid genus (*fide* Král and Smetana 2006: 248, as “*Adoretus* Laporte, 1840”).

Comments. This generic name was first proposed by Dejean as an invalid synonym of *Trigonostoma* Dejean, 1833. It was treated before 1961 as an available name and adopted as the name of a taxon (e.g., Laporte 1840: 142). Krell (2007) commented on the authorship, date and type species of *Adoretus*.

***Aegidium* Dejean, 1833: 150**

Originally included available species: none.

***Aegostheta* Dejean, 1833: 159**

Originally included available species: *Melolontha longicornis* Fabricius, 1787.

Type species: *Melolontha longicornis* Fabricius, 1787 by monotypy.

Current status: valid genus in Scarabaeidae (*fide* Lacroix 2007: 203).

***Amphicrania* Dejean, 1833: 163**

Originally included available species: *Melolontha palpalis* Eschscholtz, 1822.

Type species: *Melolontha palpalis* Eschscholtz, 1822 by monotypy.

Current status: junior objective synonym of *Liogenys* Guérin-Méneville, 1831 in Scarabaeidae (*fide* Evans 2003: 206).

***Ancylonycha* Dejean, 1833: 160**

Originally included available species: *Melolontha fervida* Fabricius, 1775; *Melolontha ilicis* Knoch, 1801; *Melolontha knochii* Schönherr and Gyllenhal, 1817 (as “Knochii. *Schönherr*.”); *Melolontha leucophthalma* Wiedemann, 1819; *Melolontha pilosicollis* Knoch, 1801; *Melolontha quercina* Knoch, 1801; *Melolontha serrata* Fabricius, 1781.

Type species: *Melolontha serrata* Fabricius, 1781 by subsequent designation (Duponchel 1840: 481).

Current status: senior objective synonym of *Holotrichia* Hope, 1837 in Scarabaeidae (*fide* Saylor 1942: 157).

Comments. *Ancylonycha* Dejean, 1833 has precedence over *Holotrichia* Hope, 1837, which is considered as the valid name for the genus (e.g., Smetana and Král 2006: 218). Reversal of Precedence (ICZN 1999: Article 23.9) cannot be used because *Ancylonycha* Dejean was used as valid after 1899 (with *Holotrichia* as synonym) at least once by Saylor (1942: 157). Therefore an application to the Commission is necesssary to conserve usage of the name *Holotrichia* Hope.

***Anisonchus* Dejean, 1833: 157**

Originally included available species: *Melolontha atriplicis* Fabricius, 1787.

Type species: *Melolontha atriplicis* Fabricius, 1787 by monotypy.

Current status: junior objective synonym of *Hoplopus* Laporte, 1832 in Scarabaeidae (*fide* Hope 1837: 70).

***Aplonycha* Dejean, 1833: 162**

Originally included available species: none.

***Arctodium* Dejean, 1833: 167**

Originally included available species: none.

***Aulacium* Dejean, 1833: 137**

Originally included available species: *Scarabaeus novaehollandiae* Fabricius *sensu* Dejean, 1833 (as “Hollandiae. *Fabr*.”).

Type species: *Scarabaeus novaehollandiae* Fabricius *sensu* Dejean 1833 (= *Aulacium carinatum* Reiche, 1841) by monotypy.

Current status: senior objective synonym of *Mentophilus* Laporte, 1840 in Scarabaeidae (*fide* Cassis and Weir 1992: 167, as “*Aulacium* Reiche”).

Comments. *Scarabaeus hollandiae* is treated here as an incorrect subsequent spelling of *Scarabaeus novaehollandiae* Fabricius, 1775 introduced by Fabricius (1781: 32).

According to the ICZN (1999: Article 70.3), an author who discovers that a type species was misidentified, which is the case here, may select, and thereby fix as type species, the nominal species previously cited as type species (e.g., *Scarabaeus novaehollandiae* Fabricius, 1775) or the taxonomic species actually involved in the misidentification (e.g., *Aulacium carinatum* Reiche, 1841). *Scarabaeus novaehollandiae* is currently the type species of the genus *Tesserodon* Hope, 1837 by monotypy and *Aulacium carinatum* is currently placed in the genus *Mentophilus* Laporte, 1840, as a junior synonym of *Mentophilus hollandiae* Laporte, 1840 (Cassis and Weir 1992: 167). However Laporte’s *Mentophilus hollandiae* is not an available species since Laporte (1840: 74) referred to Fabricius’ (1801: 57) *Ateuchus hollandiae* (as “Fabr., 1, 57, 14”) when he described the species. *Aulacium carinatum* Reiche (1841: 211) is the valid name for the species currently know as *Mentophilus hollandiae* Laporte. We are selecting as type species of *Aulacium* Dejean the species actually involved in the misidentification, *Aulacium carinatum* Reiche, 1841. This makes *Aulacium* Dejean, 1833 a senior synonym of *Mentophilus* Laporte, 1840. An application to the Commission is necesssary to conserve usage of the name *Mentophilus* Laporte. We were unable to find 25 references in the last 50 years to qualify *Mentophilus* Laporte as *nomen protectum* (see ICZN 1999: Article 23.9.1).

***Barybas* Dejean, 1833: 164**

Originally included available species: none.

***Brachysternus* Dejean, 1833: 154**

Originally included available species: none.

***Bubas* Dejean, 1833: 143** (as “Bubas. *Megerle*.”)

Originally included available species: *Scarabaeus bison* Linnaeus, 1767 (as “Bison. *Fabr*.”); *Onitis bubalus* Olivier, 1812 (as “Bubalus. *Latreille*.”).

Type species: *Scarabaeus bison* Linnaeus, 1767 by subsequent designation (Janssens 1937: 135).

Current status: valid genus in Scarabaeidae (*fide* Bezděk and Krell 2006: 158, as “*Bubas* Mulsant, 1842”).

***Caelidia* Dejean, 1833: 155**

Originally included available species: none.

***Caelodera* Dejean, 1833: 159**

Comments. This name is treated as a replacement name for *Pachypus* Dejean, 1821 [Scarabaeidae], a junior homonym of *Pachypus* Billberg, 1820 [Scarabaeidae]. However, *Pachypus* Billberg, 1820 is a *nomen oblitum* and *Pachypus* Dejean, 1821a *nomen protectum* following Bouchard et al. (2011: 253).

***Callichloris* Dejean, 1833: 155**

Originally included available species: none.

***Carteronyx* Dejean, 1833: 162**

Originally included available species: none.

***Catalasis* Dejean, 1833: 159**

Originally included available species: *Melolontha anketera* Herbst, 1790; *Melolontha australis* Gyllenhal, 1817 (as “Australis. *Schönherr*.”); *Melolontha occidentalis* Fabricius, 1775; *Melolontha orientalis* Krynicki, 1832 (as “Orientalis. *Ziegler*.”); *Melolontha pilosa* Fabricius, 1792; *Melolontha villosa* Fabricius, 1781.

Type species: *Melolontha villosa* Fabricius, 1781 by subsequent designation (Bezděk 2006b: 191).

Current status: junior objective synonym of *Anoxia* Laporte, 1832 in Scarabaeidae (*fide* Bezděk 2006b: 191).

***Chalconotus* Dejean, 1833: 136**

Originally included available species: *Scarabaeus cupreus* Fabricius, 1775.

Type species: *Scarabaeus cupreus* Fabricius, 1775 by monotypy.

Current status: valid genus in Scarabaeidae (*fide* Branco 2011: 12).

Comments. Until recently the name *Chalconotus* was attributed to Reiche (1841: 212) and considered a junior objective synonym of *Anachalcos* Hope, 1837. Branco (2011: 12) corrected the situation.

***Chloenobia* Dejean, 1833: 161**

Originally included available species: none.

***Chlorota* Dejean, 1833: 154**

Originally included available species: none.

***Coprobas* Dejean, 1833: 137**

Originally included available species: none.

***Coptorhinus* Dejean, 1833: 152**

Originally included available species: *Scarabaeus retusus* Fabricius, 1781.

Type species: *Scarabaeus retusus* Fabricius, 1781 by monotypy.

Current status: name suppressed in Scarabaeidae.

Comments. *Coptorhinus* Dejean, 1833 was placed in Opinion 1838 on the Official Index of Rejected and Invalid Generic Names in Zoology for the purposes of the Principle of Priority but not for the Principle of Homonymy (ICZN 1996d: 134).

***Cryptodon* Dejean, 1833: 150** (as “Cryptodon. *Latreille*. ”)

Originally included available species: none.

***Dasysterna* Dejean, 1833: 159**

Originally included available species: none.

***Dorysthaetus* Dejean, 1833: 154**

Originally included available species: none.

***Encya* Dejean, 1833: 159**

Originally included available species: *Melolontha commersonii* Olivier, 1789.

Type species: *Melolontha commersonii* Olivier, 1789 by monotypy.

Current status: valid genus in Scarabaeidae (*fide* Lacroix 1993: 341).

***Epicaulis* Dejean, 1833: 164**

Originally included available species: none.

***Epichloris* Dejean, 1833: 155**

Originally included available species: *Brachysternus prasinus* Guérin-Méneville, 1831 (as “Prasina. *d’Urville*.”).

Type species: *Brachysternus prasinus* Guérin-Méneville, 1831 by monotypy.

Current status: junior objective synonym of *Brachysternus* Guérin-Méneville, 1831 (*fide* Gemminger and Harold 1869: 1231).

***Epirinus* Dejean, 1833: 137**

Originally included available species: *Canthon aeneus* Wiedemann, 1823; *Scarabaeus granulatus* Olivier, 1789.

Type species: *Scarabaeus granulatus* Olivier, 1789 (=*Scarabaeus flagellatus* Fabricius, 1775) by subsequent designation (Scholtz and Howden 1987: 122).

Current status: valid genus in Scarabaeidae (*fide* Medina and Scholtz 2005: 147, as “*Epirinus* Reiche, 1841”).

Comments. Scholtz and Howden (1987: 122) designated *Scarabaeus flagellatus* Fabricius, 1775 as the type of *Epirinus* (as “*Epirinus* Reiche”), a species not originally included. However since they placed at the same time that species in synonymy with *Scarabaeus granulatus* Olivier, 1789, a species originally included, they are deemed to have designated the latter species as type species (ICZN 1999: Article 69.2.2). Janssens (1938: 12) also designated *Scarabaeus flagellatus* Fabricius, 1775 as type species of *Epirinus* (as “*Epirinus* Reiche”) but he did not list the species in synonymy with *Scarabaeus granulatus* Olivier, 1789.

***Eriesthis* Dejean, 1833: 167**

Originally included available species: none.

***Eucheirus* Dejean, 1833: 140**

Originally included available species: none.

***Eucranium* Dejean, 1833: 135**

Originally included available species: none.

***Geobatus* Dejean, 1833: 164**

Originally included available species: none.

***Gromphas* Dejean, 1833: 143**

Originally included available species: none.

***Gymnogaster* Dejean, 1833: 159**

Originally included available species: none.

***Gymnoloma* Dejean, 1833: 167**

Originally included available species: *Melolontha atomaria* Fabricius, 1781.

Type species: *Melolontha atomaria* Fabricius, 1781 by monotypy.

Current status: valid genus in Scarabaeidae (*fide* Dombrow 2001: 107 as “*Gymnoloma* Burmeister, 1844”).

***Heteronychus* Dejean, 1833: 152**

Originally included available species: *Scarabaeus aries* Fabricius, 1781; *Geotrupes cricetus* Hausmann, 1807; *Scarabaeus morator* Fabricius, 1798; *Scarabaeus piceus* Fabricius, 1775; *Scarabaeus syrichtus* Fabricius, 1775.

Type species: *Geotrupes cricetus* Hausmann, 1807 by subsequent designation (Krell 2002: 40).

Current status: valid genus in Scarabaeidae (*fide* Krell 2006: 280).

Comments: The first valid typification for *Heteronychus* Dejean is that of Duponchel (1845: 601) who selected *Scarabaeus syrichtus* Fabricius, 1775. However, this species is currently placed in the genus *Syrictes* Prell, 1936 (e.g., Endrödi 1985: 685). An application to the Commission to reject the typification of Duponchel is needed in order to keep using the current taxonomic concepts of *Heteronychus* and *Syrictes*. It appears that *Syrictes* Prell is itself a preoccupied genus name (not *Syrictes* Jordan & Evermann, 1927 [Pisces]) and needs a replacement name.

***Hoplites* Dejean, 1833: 150**

Originally included available species: *Scarabaeus enema* Fabricius, 1787; *Scarabaeus pan* Fabricius, 1775.

Type species: none found.

Current status: senior synonym of *Enema* Hope, 1837 (*fide* Ratcliffe 2003: 295).

Comments. *Hoplites* Dejean, 1833 was treated as a *nomen oblitum* and *Enema* Hope, 1837 a *nomen protectum* by Ratcliffe (2003: 295). However, the name was suppressed by the Commission for both the Principle of Priority and Principle of Homonymy in Opinion 353 (ICZN 1955).

***Hybalus* Dejean, 1833: 149**

Originally included available species: *Geobius cornifrons* Brullé, 1832 (as “Cornifrons. *Dej*.”); *Scarabaeus glabratus* Fabricius, 1792 (as “*Glabratus*. *Paykull*.”).

Type species: *Geobius cornifrons* Brullé, 1832 by monotypy.

Current status: valid genus in Scarabaeidae (*fide* López-Colón 2006: 179).

Comments: The name *glabratus* is listed in synonymy with *cornifrons* in Dejean’s catalogue; therefore the type species of *Hybalus* is *cornifrons* by monotypy (ICZN 1999: Article 68.3).

***Hyperis* Dejean, 1833: 167**

Originally included available species: *Hoplia eversmanni* Faldermann, 1833.

Type species: *Hoplia eversmanni* Faldermann, 1833 (= *Hoplia paupera* Krynicki, 1832) by monotypy.

Current status: valid subgenus of *Hoplia* Illiger, 1803 in Scarabaeidae (*fide* Smetana 2006: 188).

Comments. The date of publication of volume 6 of the *Bulletin de la Société Impériale des Naturalistes de Moscou*, where the species *Hoplia eversmanni* Faldermann was published, is unknown besides the year. The permit for publication was delivered on 16 March 1833 [Julian calendar = 28 March 1833 for the Gregorian calendar] and suggests that the actual date of publication preceeds that of the second livraison of Dejean’s catalogue recorded on 27 July 1833. *Hyperis* was considered as available and credited to Dejean in recent publications (e.g., Hardy 1977: 6; Smetana and Smith 2006: 50; Smetana 2006: 188). To promote stability we consider that *Hoplia eversmanni* Faldermann, 1833 was available before the publication of Dejean’s catalogue despite that the date of publication of the *Bulletin* should theoretically be the last day of the year (ICZN 1999: 21.3.2). An application to the Commission may be necessary in this case.

***Hyporhiza* Dejean, 1833: 162**

Originally included available species: *Melolontha hypocrita* Mannerheim, 1829.

Type species: *Melolontha hypocrita* Mannerheim, 1829 by monotypy.

Current status: junior subjective synonym of *Rhinaspis* Perty, 1830 in Scarabaeidae (**new synonymy**).

Comments. *Hyporhiza* Dejean is a senior objective synonym of *Ulomenes* Blanchard, 1850. *Ulomenes* was used as the valid name for this genus (e.g., Evans 2003: 346) until Katovich (2008: 1) synonymized *Ulomenes* Blanchard with *Rhinaspis* Perty.

***Lagosterna* Dejean, 1833: 159**

Originally included available species: none.

***Lasiopus* Dejean, 1833: 164**

Originally included available species: none.

***Leocaeta* Dejean, 1833: 159**

Originally included available species: *Melolontha alopex* Fabricius, 1787.

Type species: *Melolontha alopex* Fabricius, 1787 by monotypy.

Current status: senior synonym of *Sparrmannia* Laporte, 1840 in Scarabaeidae (*fide* Dalla Torre 1913: 291).

Comments. *Leocaeta* Dejean, 1833is a *nomen oblitum* and *Sparrmannia* Laporte, 1840 a *nomen protectum* following Bouchard et al. (2011: 255).

***Leptopus* Dejean, 1833: 159**

Originally included available species: none.

***Leucopholis* Dejean, 1833: 160**

Originally included available species: *Melolontha alba* Olivier, 1789 (as “Alba. *Fabr*.”); *Melolontha hypoleuca* Wiedemann, 1819; *Melolontha rorida* Fabricius, 1801; *Melolontha stigma* Fabricius, 1794.

Type species: *Melolontha rorida* Fabricius, 1801 by subsequent designation (Bezděk 2006a: 33).

Current status: valid genus in Scarabaeidae (*fide* Bezděk 2006b: 190).

***Macrothops*** **Dejean, 1833: 164** (as “Macrothops. *Mac Leay*.”)

Originally included available species: none.

***Mallogaster* Dejean, 1833: 162**

Originally included available species: none.

***Microplus* Dejean, 1833: 166**

Originally included available species: none.

***Myoderma* Dejean, 1833: 168**

Originally included available species: *Stripsypher sordidus* Gory and Percheron, 1833 (as “Sordida. *Dej*.”).

Type species: *Stripsypher sordidus* Gory and Percheron, 1833 (= *Trichius alutaceum* Afzelius, 1817) by monotypy.

Current status: valid genus in Scarabaeidae (*fide* Ricchiardi and Gill 2009: 147 as “*Myodermum* Burmeister and Schaum, 1840”).

Comments. The name *Stripsypher sordidus* Gory and Percheron, 1833 was published and validated as “sordidus. *Dej*.” in the first livraison of Gory and Percheron’s *Monographie des cétoines et genres voisins* which was issued before the second livraison of Dejean’s catalogue (see “Precedence” section [3]). The second species listed by Dejean (1833: 168), “Fuliginosa. *Dej*.,” is not in Gory and Percheron’s publication.

This generic name is usually credited to Burmeister and Schaum (1840: 396) under the spelling *Myodermum* (e.g. Smith 2006: 178; Ricchiardi and Gill 2009: 147). This spelling is in prevailing usage but not attributed to the original author (see ICZN 1999: Article 33.3.1). Therefore the original spelling used by Dejean must be retained.

***Onthocharis* Dejean, 1833: 144**

Originally included available species: none.

***Onthoecus* Dejean, 1833: 140**

Originally included available species: none.

***Ootoma* Dejean, 1833: 163**

Originally included available species: none.

***Orthognatus* Dejean, 1833: 174**

Originally included available species: none.

***Oxyomus* Dejean, 1833: 147** (as “Oxyomus. *Eschscholtz*.”)

Originally included available species: *Scarabaeus asper* Fabricius, 1775; *Aphodius bicolor* Say, 1823; *Scarabaeus caesus* Creutzer, 1796 (as “Caesus. *Fabr*.”); *Scarabaeus porcatus* Fabricius, 1775; *Scarabaeus sabuleti* Panzer, 1797 (as “Sabuleti. *Fabr*.”); *Scarabaeus stercorator* Fabricius, 1775; *Aphodius strigatus* Say, 1823.

Type species: *Scarabaeus porcatus* Fabricius, 1775 (= *Scarabaeus sylvestris* Scopoli, 1763) by subsequent designation (Westwood 1838: 23).

Current status: valid genus in Scarabaeidae (*fide* Dellacasa and Dellacasa 2006: 141).

***Pachylus* Dejean, 1833: 152**

Originally included available species: none.

***Philochloenia*Dejean, 1833: 163**

Originally included available species: *Melolontha elongata* Fabricius, 1792; *Melolontha filitarsis* Germar, 1824.

Type species: *Melolontha filitarsis* Germar, 1824 (= *Melolontha rufipennis* Fabricius, 1801) by subsequent designation (Chevrolat 1847a: 735).

Current status: valid genus in Scarabaeidae (*fide* Bouchard et al. 2011: 251).

Comments. Smith and Evans (2005: 40) designated *Melolontha elongata* Fabricius, 1792 as type species of *Philochloenia* which made the name a junior synonym of *Dichelonyx* Harris, 1827. However there is a valid earlier typification which makes *Philochloenia* a senior synonym of *Anoplosiagum* Blanchard, 1850 in the Melolonthinae (Scarabaeidae). Bouchard et al. (2011: 251) used the second approach and this seems the best avenue (Andrew Smith personal communication 2012).

***Phytolaema* Dejean, 1833: 162**

Originally included available species: none.

***Platycheira* Dejean, 1833: 157**

Originally included available species: none.

***Platycoelia* Dejean, 1833: 154**

Originally included available species: *Melolontha flavostriata* Latreille, 1813.

Type species: *Melolontha flavostriata* Latreille, 1813 by monotypy.

Current status: valid genus in Scarabaeidae (*fide* Smith 2003: 31).

***Podalgus* Dejean, 1833: 152**

Originally included available species: none.

***Psalicerus* Dejean, 1833: 174**

Originally included available species: *Lucanus femoratus* Fabricius, 1775; *Lucanus ibex* Billberg, 1820 (as “*Ibex. Sahlberg*.”).

Type species: *Lucanus femoratus* Fabricius, 1775 by **present designation**.

Current status: senior objective synonym of *Leptinopterus* Hope, 1838 in Lucanidae (*fide* Krajčik 2001: 27).

Comments. *Psalicerus* Dejean, 1833 has precedence over *Leptinopterus* Hope, 1838 which is currently used as valid (e.g., Krajčik 2001: 27). Reversal of Precedence (ICZN 1999: Article 23.9) or an application to the Commission is necessary to conserve usage of the name *Leptinopterus* Hope.

***Pygurus* Dejean, 1833: 137**

Originally included available species: none.

***Rhinyptia* Dejean, 1833: 157**

Originally included available species: none.

***Rhizobia* Dejean, 1833: 157**

Originally included available species: none.

***Ryparus* Dejean, 1833: 144**

Originally included available species: none.

***Schizonycha* Dejean, 1833: 161**

Originally included available species: *Melolontha cylindrica* Gyllenhal, 1817 (as “Cylindrica. *Schönherr*.”); *Scarabaeus globator* Fabricius, 1781; *Melolontha henningii* Fischer von Waldheim, 1823 (as “Henningii. *Gebler*.”); *Melolontha moesta* Say, 1825.

Type species: *Scarabaeus globator* Fabricius, 1781 by subsequent designation (Pope 1960: 68).

Current status: valid genus in Scarabaeidae (*fide* Smetana and Král 2006: 228).

***Sciuropus* Dejean, 1833: 162**

Originally included available species: *Melolontha rufipes* Latreille, 1813.

Type species: *Melolontha rufipes* Latreille, 1813 by monotypy.

Current status: senior subjective synonym of *Ancistrosoma* Curtis, 1835 in Scarabaeidae (*fide* Dalla Torre 1913: 338).

Comments. *Sciuropus* Dejean, 1833 has precedence over *Ancistrosoma* Curtis, 1835 which is currently used as valid (e.g., Evans 2003: 224). Reversal of Precedence (ICZN 1999: Article 23.9) or an application to the Commission is necessary to conserve usage of the name *Ancistrosoma* Curtis, 1835.

***Sericesthis* Dejean, 1833: 164**

Originally included available species: none.

***Sphaeromorphus* Dejean, 1833: 147** (as “Sphaeromorphus. *Germar*.”)

Originally included available species (from Germar 1843): *Sphaeromorphus basilicus* Germar, 1843; *Sphaeromorphus bicinctus* Germar, 1843; *Sphaeromorphus chalceus* Germar, 1843; *Sphaeromorphus ebeninus* Erichson, 1843; *Sphaeromorphus globulus* Erichson, 1843; *Sphaeromorphus humeralis* Erichson, 1843; *Sphaeromorphus nanus* Germar, 1843; *Sphaeromorphus nitidus* Germar, 1843; *Sphaeromorphus politus* Erichson, 1843; *Sphaeromorphus pyritosus* Erichson, 1843; *Sphaeromorphus semipunctatus* Germar, 1843; *Sphaeromorphus semistriatus* Germar, 1843; *Sphaeromorphus seriatus* Erichson, 1843; *Sphaeromorphus sesquistriatus* Germar, 1843; *Sphaeromorphus volvox* Erichson, 1843.

Type species: *Sphaeromorphus humeralis* Erichson, 1843 by **present designation**.

Current status: senior subjective synonym of *Ceratocanthus* White, 1842 in Scarabaeidae (*fide* Howden and Gill 2000: 289, as “*Sphaeromorphus* Germar”).

Comments. This generic name was proposed by Dejean as an invalid synonym of *Acanthocerus* Macleay, 1819. It is available because it was treated before 1961 as an available name and adopted as the name of a taxon (e.g., Germar 1843: 111). All the species listed by Dejean (1833: 147) under this name are unavailable. The first species (cited by available names) directly associated with the name *Sphaeromorphus* are those cited by Germar (1843: 114–124).

*Sphaeromorphus* Dejean, 1833 has precedence over *Ceratocanthus* White, 1842. Reversal of Precedence (ICZN 1999: Article 23.9) or an application to the Commission is necessary to retain *Ceratocanthus* White as a valid taxon.

***Spilota* Dejean, 1833: 155**

Originally included available species: none.

***Streptocerus* Dejean, 1833: 174**

Originally included available species: none.

***Strigidia* Dejean, 1833: 155**

Originally included available species: none.

***Strigoderma* Dejean, 1833: 158**

Originally included available species: none.

***Tarandus*** **Dejean, 1833: 174** (as “Tarandus. *Megerle*.”)

Originally included available species: *Lucanus tenebrioides* Fabricius, 1787.

Type species: *Lucanus tenebrioides* Fabricius, 1787 (= *Lucanus chrysomelinus* Hochenwarth, 1785) by monotypy.

Current status: junior homonym of *Tarandus* Billberg, 1827 [Mammalia]; junior objective synonym of *Ceruchus* MacLeay, 1819 in Lucanidae (*fide* Krajčik 2001: 4).

***Thyridium* Dejean, 1833: 154**

Originally included available species: none.

***Trichops*** **Dejean, 1833: 164** (as “Trichops. *Mannerheim*.”)

Originally included available species: none.

***Trigonostoma* Dejean, 1833: 157**

Originally included available species: *Melolontha compressa* Weber, 1801; *Melolontha lanata* Fabricius, 1801; *Melolontha nigrifrons* Steven, 1809; *Melolontha obscura* Fabricius, 1781; *Melolontha senegallia* Dufour, 1821.

Type species: *Melolontha lanata* Fabricius, 1801 by subsequent designation (Machatschke 1972: 310).

Current status: junior homonym of *Trigonostoma* Blainville, 1825 [Mollusca]; senior objective synonym of *Adoroleptus* Brenske, 1893 in Scarabaeidae (*fide* Machatschke 1972: 310; Willems and Krell 2007: 220).

***Trionychus* Dejean, 1833: 150**

Originally included available species: none.

***Xylonichus* Dejean, 1833: 155** (as “Xylonichus. *Mac Leay*.”)

Originally included available species: none.

### Hétéromères: Mélasomes

***Acisba* Dejean, 1834: 185** (as “Acisba. *Ziegler*.”)

Originally included available species: *Tentyria brevis* Besser, 1832 (as “*Brevis. Solier*”); *Tentyria cribrosa* Besser, 1832 (as “*Cribrosa. Solier*”); *Tentyria subovata* Besser, 1832 (as “Subovata. *Kollar*.).

Type species: *Tentyria cribrosa* Besser, 1832 (= *Pimelia punctata* Fabricius, 1798) by subsequent designation (Löbl et al. 2008a: 40).

Current status: junior objective synonym of *Pachychila* Eschscholtz, 1831 in Tenebrionidae (*fide* Löbl et al. 2008b: 199).

***Aethales* Dejean, 1834: 180**

Originally included available species: *Epitragus brunnicornis* Latreille, 1811.

Type species: *Epitragus brunnicornis* Latreille, 1811 by monotypy.

Current status: junior synonym of *Epitragus* Latreille, 1802 in Tenebrionidae (**new synonymy**).

Comments. *Epitragus brunnicornis* Latreille is listed as a species *incertae sedis* in the genus *Epitragus* Latreille by Freude (1967: 176).

***Amatodes* Dejean, 1834: 189**

Originally included available species: *Pimelia gemmata* Fabricius, 1801.

Type species: *Pimelia gemmata* Fabricius, 1801 by monotypy.

Current status: senior synonym of *Oncosoma* Westwood, 1843 in Tenebrionidae (*fide* Gebien 1943: 905, as “*Amatodes* Solier, 1844”).

Comments. *Amatodes* Dejean, 1834 has precedence over *Oncosoma* Westwood, 1843 which is currently used as valid (e.g., Robiche 2008: 525). Reversal of Precedence (ICZN 1999, Article 23.9) or an application to the Commission is necessary to conserve usage of the name *Oncosoma* Westwood, 1843.

***Amphysus* Dejean, 1834: 189**

Originally included available species: none.

***Arctylus* Dejean, 1834: 180** (as “Arctylus. *Solier*.”)

Originally included available species: *Praocis pentagonus* Lacordaire, 1830.

Type species: *Praocis pentagonus* Lacordaire, 1830 by monotypy.

Current status: junior subjective synonym of *Praocis* Eschscholtz, 1829 in Tenebrionidae (**new synonymy**).

Comments. Two of the species included by Dejean (1834: 180) in this genus were described earlier by Lacordaire (1830: 286) as “*N… ursinus*” and “*N… dasypoides*” where “*N…*” possibly stands for “nouveau” [new] but unnamed genus, not *Nyctelia* as recorded by Sherborn in his *Index Animalium*. According to Article 11.9.3 (ICZN 1999), a species-group name must be published in unambiguous combination with a generic name. This is not the case and so Lacordaire’s species are considered unavailable.

The species *ursinus* was made available by Guérin-Méneville (1834: plate 105) in volume 4 of the *Magasin de Zoologie*. We were unable to find any precise date of publication for that volume. Therefore we consider that the name was not available before the publication of the third livraison of Dejean’s catalogue.

*Praocis pentagonus* Lacordaire is listed as a species *incertae sedis* in the genus *Praocis* Eschscholtz by Vidal and Guerrero (2007: 74).

***Blacodes* Dejean, 1834: 190**

Originally included available species: none.

***Brachygenius* Dejean, 1834: 186** (as “Brachygenius. *Solier*.”)

Originally included available species: none.

***Brachyscelis* Dejean, 1834: 179** (as “Brachyscelis. *Solier*.”)

Originally included available species: *Pimelia clavaria* Faldermann, 1832; *Pimelia metopotapha* Fischer von Waldheim, 1832; *Pimelia musiva* Faldermann, 1832.

Type species: *Pimelia musiva* Faldermann, 1832 by subsequent designation (Bouchard et al. 2007: 388).

Current status: senior synonym of *Pachyscelis* Solier, 1836 in Tenebrionidae (*fide* Löbl et al. 2008b: 154).

Comments. *Brachyscelis* Dejean, 1834is a *nomen oblitum* and *Pachyscelis* Solier, 1836 a *nomen protectum* following Bouchard et al. (2007: 388).

***Bradytes* Dejean, 1834: 182**

Originally included available species: none.

Comments. The sole species included by Dejean (1834: 182) in this genus was described earlier by Lacordaire (1830: 287) as “*N… strangulatus*” where “*N…*” possibly stands for “nouveau” [new] but unnamed genus, not *Nyctelia* as recorded by Sherborn in his *Index Animalium*. According to Article 11.9.3 (ICZN 1999), a species-group name must be published in unambiguous combination with a generic name. This is not the case and so Lacordaire’s species is considered unavailable.

***Bradyus* Dejean, 1834: 190**

Originally included available species: *Erodius pygmaeus* Fischer von Waldheim, 1821.

Type species: *Erodius pygmaeus* Fischer von Waldheim, 1821 by monotypy.

Current status: valid genus in Tenebrionidae (*fide* Löbl et al. 2008b: 240).

***Cacicus* Dejean, 1834: 182**

Originally included available species: *Elenophorus americanus* Lacordaire, 1830.

Type species: *Elenophorus americanus* Lacordaire, 1830 by monotypy.

Current status: junior homonym of *Cacicus* Lacépède, 1799 [Aves]; senior synonym of *Megelenophorus* Gebien, 1910 in Tenebrionidae (*fide* Gebien 1937: 701, as “*Cacicus* Solier, 1836”).

***Caedius* Dejean, 1834: 190**

Originally included available species: none.

***Calymmaphorus* Dejean, 1834: 180** (as “Calymmaphorus. *Solier*.”)

Originally included available species: none.

Comments. The sole species included by Dejean in this genus was described earlier by Lacordaire (1830: 286) as “*N… cucullatus*” where “*N…*” possibly stands for “nouveau” [new] but unnamed genus, not *Nyctelia* as recorded by Sherborn in his *Index Animalium*. According to Article 11.9.3 (ICZN 1999), a species-group name must be published in unambiguous combination with a generic name. This is not the case and so Lacordaire’s species is considered unavailable.

***Cephalostenus* Dejean, 1834: 183** (as “Cephalostenus. *Solier*.”)

Originally included available species: none.

***Cilibe* Dejean, 1834: 187** (as “Cilibe. *Latreille*.”)

Originally included available species: none.

***Colposcelis* Dejean, 1834: 185** (as “Colposcelis. *Solier*.”)

Originally included available species: *Tentyria abbreviata* Gebler, 1830; *Tentyria acutangula* Faldermann, 1833; *Tentyria angulosa* Gebler, 1832 (as “Angulosa. *Fischer*.”); *Tentyria angustata* Steven, 1829 (as “*Angustata. Gebler*.”); *Tentyria angusticollis* Gebler, 1830; *Tentyria depressa* Gebler, 1830 (as “*Depressa. Fischer*.”); *Tentyria elongata* Gebler, 1829 (as “*Elongata*. *Fischer*.”); *Tentyria eremita* Steven, 1829; *Tentyria gibbosa* Steven, 1829 (as “Gibbosa. *Gebler*.”); *Tentyria impressa* Tauscher, 1812; *Tentyria lata* Steven, 1829 (as “Lata. *Gebler*.”); *Tentyria longicollis* Zoubkoff, 1833 (as “*Longicollis. Karelin*.”); *Tentyria macrocephala* Tauscher, 1812; Tentyria pygmaea Gebler, 1832 (as “Pygmaea. *Mannerheim*.”); *Tentyria strigosa* Germar, 1824 (as Strigosa. *Gebler*.”); *Tentyria subquadrata* Tauscher, 1812; *Tentyria undulata* Gebler, 1832 (as “Undulata. *Mannerheim*”).

Type species: *Tentyria longicollis* Zoubkoff, 1833 by subsequent designation (Gebien 1937: 598).

Current status: valid genus in Tenebrionidae (*fide* Löbl et al. 2008b: 186).

***Comphosida* Dejean, 1834: 184** (as “Comphosida. *Solier*.”)

Originally included available species: none.

***Coronus* Dejean, 1834: 192**

Originally included available species: none.

***Cyrtoderes* Dejean, 1834: 181** (as “Cyrtoderes. *Solier*.”)

Originally included available species: *Tenebrio cristatus* DeGeer, 1778 (as “*Cristatus*. *Fab*.”); *Sepidium lacunosum* Thunberg, 1784.

Type species: *Sepidium lacunosum* Thunberg, 1784 (= *Tenebrio cristatus* DeGeer, 1778) by **present designation.**

Current status: senior synonym of *Phligra* Laporte, 1840 in Tenebrionidae (*fide* Gebien 1910: 149, as “*Cyrtoderes* Solier, 1844”).

Comments. *Cyrtoderes* Dejean, 1834 has precedence over *Phligra* Laporte, 1840 which is currently used as valid (e.g., Koch 1955: 46). Reversal of Precedence (ICZN 1999: Article 23.9) or an application to the Commission is necessary to conserve usage of the name *Phligra* Laporte, 1840.

***Dicrossa* Dejean, 1834: 181** (as “Dicrossa. *Klug*.”)

Originally included available species: none.

***Echinotus* Dejean, 1834: 181**

Originally included available species: none.

***Entomoderes* Dejean, 1834: 186** (as “Entomoderes. *Solier*.”)

Originally included available species: none.

Comments. One of the species included by Dejean (1834: 186) in this genus was described earlier by Lacordaire (1830: 281) as “*N… erebi*” where “*N…*” possibly stands for “nouveau” [new] but unnamed genus, not *Nyctelia* as recorded by Sherborn in his *Index Animalium*. According to Article 11.9.3 (ICZN 1999), a species-group name must be published in unambiguous combination with a generic name. This is not the case and so Lacordaire’s species is considered unavailable.

***Epilasium* Dejean, 1834: 192**

Originally included available species: none.

***Epiphysa* Dejean, 1834: 178**

Originally included available species: *Pimelia flavicollis* Fabricius, 1794; *Pimelia inflata* Olivier, 1795.

Type species: *Pimelia flavicollis* Fabricius, 1794 by monotypy.

Current status: valid genus in Tenebrionidae (*fide* Bouchard et al. 2005: 507).

Comments: The name *inflata* is listed in synonymy with *flavicollis* in Dejean’s catalogue; therefore the type species of *Epiphysa* is *flavicollis* by monotypy (ICZN 1999: Article 68.3).

***Hadrus* Dejean, 1834: 192**

Originally included available species: none.

***Heliopates* Dejean, 1834: 191**

Comments. This name is treated as a replacement name for *Heliophilus* Dejean, 1821 [Tenebrionidae], a junior homonym of *Heliophilus* Meigen, 1803 [Diptera]. *Heliophilus* Dejean is currently listed as an invalid synonym of *Phylan* Dejean, 1821 [type species: *Pedinus hybridus* Latreille, 1804] (Löbl et al. 2008: 282) and *Heliopates* Dejean should also be a junior synonym of *Phylan* Dejean. However, *Heliopates* Dejean, 1834 is currently considered a valid genus (Löbl et al. 2008: 279) with *Tenebrio lusitanicus* Herbst, 1797 as type species. To promote stability, we believe the best avenue would be to submit an application to the Commission to retain *Tenebrio lusitanicus* Herbst as type species of *Heliopates* Dejean, 1834.

***Herpiscius* Dejean, 1834: 183**

Originally included available species: none.

***Hipomelus* Dejean, 1834: 181**

Originally included available species: *Sepidium acuminatum* Quensel, 1806 (as “Acuminatus. *Schöenherr*.”); *Sepidium vittatum* Fabricius, 1781.

Type species: *Sepidium vittatum* Fabricius, 1781 by subsequent designation (Hope 1840: 116).

Current status: junior subjective synonym of *Trachynotus* Latreille, 1828 in Tenebrionidae (**new synonymy**).

***Lasiostola* Dejean, 1834: 179**

Originally included available species: *Pimelia hirta* Fischer von Waldheim, 1820; *Tenebrio pubescens* Pallas, 1781.

Type species: *Tenebrio pubescens* Pallas, 1781 by subsequent designation (Hope 1840: 118).

Current status: valid genus in Tenebrionidae (*fide* Löbl et al. 2008b: 152).

***Leichenum* Dejean, 1834: 194**

Originally included available species: *Opatrum pictum* Fabricius, 1801.

Type species: *Opatrum pictum* Fabricius, 1801 by monotypy.

Current status: valid genus in Tenebrionidae (*fide* Löbl et al. 2008b: 283).

***Leptodes* Dejean, 1834: 181**

Originally included available species: *Sepidium boisduvalii* Zoubkoff, 1833.

Type species: *Sepidium boisduvalii* Zoubkoff, 1833 by monotypy.

Current status: valid genus in Tenebrionidae (*fide* Löbl et al. 2008b: 149).

***Macrotis* Dejean, 1834: 186**

Originally included available species: none.

***Melancrus* Dejean, 1834: 185**

Originally included available species: none.

***Melanesthes* Dejean, 1834: 191**

Originally included available species: *Pedinus laticollis* Gebler, 1829 (as “Laticollis. *Fald*.”); *Opatrum sibiricum* Faldermann, 1833.

Type species: *Opatrum sibiricum* Faldermann, 1833 by subsequent designation (Gebien 1939: 462).

Current status: valid genus in Tenebrionidae (*fide* Löbl et al. 2008b: 267).

***Melanostola* Dejean, 1834: 179**

Originally included available species: none.

***Metopocerus* Dejean, 1834: 190**

Originally included available species: none.

***Microzoum* Dejean, 1834: 193**

Originally included available species: *Opatrum tibiale* Fabricius, 1781.

Type species: *Opatrum tibiale* Fabricius, 1781 by monotypy.

Current status: junior objective synonym of *Melanimon* Steven, 1829 in Tenebrionidae (*fide* Löbl et al. 2008b: 258).

***Morica* Dejean, 1834: 182**

Originally included available species: *Tenebrio grossa* Linnaeus, 1767 (as “Grossa. *Olivier*.”); *Akis planata* Fabricius, 1801.

Type species: *Akis planata* Fabricius, 1801 by subsequent designation (Hope 1840: 122).

Current status: valid genus in Tenebrionidae (*fide* Löbl et al. 2008b: 127).

***Nosoderma* Dejean, 1834: 186**

Originally included available species: none.

***Notha* Dejean, 1834: 182** (as “Notha. *Eschscholtz*.”)

Comments. This name was listed by Dejean as an invalid synonym of *Scotera* Dejean, 1834, a *nomen nudum*. Therefore, *Notha* Dejean is not available.

***Notocorax* Dejean, 1834: 191**

Originally included available species: *Opatrum javanum* Wiedemann, 1819.

Type species: *Opatrum javanum* Wiedemann, 1819 by monotypy.

Current status: valid genus in Tenebrionidae (*fide* Löbl et al. 2008b: 290).

***Nyctipates* Dejean, 1834: 188**

Originally included available species: none.

***Oncotus* Dejean, 1834: 190**

Originally included available species: none.

***Pachycoelia* Dejean, 1834: 187** (as “Pachycoelia. *Boisduval*.”)

Originally included available species: none.

***Pachypterus* Dejean, 1834: 192** (as “Pachypterus. *Solier*.”)

Originally included available species: none.

***Pandarus* Dejean, 1834: 191** (as “Pandarus. *Megerle*.”)

Comments. This name is treated as an unjustified emendation of *Dendarus* Dejean, 1821 [Tenebrionidae] (*fide* Löbl et al. 2008b: 277).

***Pelecyphorus* Dejean, 1834: 186** (as “Pelecyphorus. *Solier*.”)

Originally included available species: none.

***Philoscotus* Dejean, 1834: 186**

Originally included available species: none.

***Physosterna* Dejean, 1834: 179** (as “Physosterna. *Solier*.”)

Originally included available species: *Pimelia ovata* Olivier, 1795.

Type species: *Pimelia ovata* Olivier, 1795 (= *Tenebrio torulosus* Pallas, 1781) by monotypy.

Current status: valid genus in Tenebrionidae (*fide* Penrith 1979: 30, as “*Physosterna* Solier, 1837”).

***Pilioloba* Dejean, 1834: 194** (as “Pilioloba. *Solier*.”)

Originally included available species: none.

***Platyholmus* Dejean, 1834: 180** (as “Platyholmus. *Solier*.”)

Originally included available species: *Praocis dilaticollis* Lacordaire, 1830; *Praocis gravidus* Lacordaire, 1830.

Type species: *Praocis dilaticollis* Lacordaire, 1830 by subsequent designation (Desmarest 1860: 142).

Current status: valid genus in Tenebrionidae (*fide* Gebien 1938: 79, as “*Platyholmus* Solier, 1840”).

***Prionotheca* Dejean, 1834: 179** (as “Prionotheca. *Solier*.”)

Originally included available species: *Pimelia coronata* Olivier, 1795.

Type species: *Pimelia coronata* Olivier, 1795 by monotypy.

Current status: valid genus in Tenebrionidae (*fide* Löbl et al. 2008b: 165).

***Psorodes* Dejean, 1834: 189**

Comments. This genus is treated as a replacement name for *Acanthomera* Latreille, 1828 [Tenebrionidae], a junior homonym of *Acanthomera* Wiedemann, 1821 [Diptera].

***Pterocoma* Dejean, 1834:** 178 (as “Pterocoma. *Solier*.”)

Originally included available species: *Pimelia piligera* Gebler, 1830.

Type species: *Pimelia piligera* Gebler, 1830 by monotypy.

Current status: valid genus in Tenebrionidae (*fide* Löbl et al. 2008b: 166).

***Pterolasia* Dejean, 1834: 179** (as “Pterolasia. *Solier*.”)

Originally included available species: none.

***Sciaca* Dejean, 1834: 184**

Originally included available species: none.

Comments. The two species included by Dejean (1834: 184) in this genus were described earlier by Lacordaire (1830: 286) as “*N… tentyrioides*” and “*N… antarctica*” where “*N…*” possibly stands for “nouveau” [new] but unnamed genus, not *Nyctelia* as recorded by Sherborn in his *Index Animalium*. According to Article 11.9.3 (ICZN 1999), a species-group name must be published in unambiguous combination with a generic name. This is not the case and so Lacordaire’s species are considered unavailable.

The species *tentyrioides* was made available by Guérin-Méneville (1834: 12) in volume 4 of the *Magasin de Zoologie*. We were unable to find any precise date of publication for that volume. Therefore we consider that the name was not available before the publication of the third livraison of Dejean’s catalogue.

***Sclerum* Dejean, 1834: 193**

Originally included available species: *Opatrum ferrugineum* Fabricius, 1801 (as “*Ferrugineum. Eschsch. Fabr?*”); *Opatrum foveolatum* Olivier, 1811; *Opatrum orientale* Fabricius, 1775.

Type species: *Opatrum orientale* Fabricius, 1775 by subsequent designation (Hope 1840: 110).

Current status: valid genus in Tenebrionidae (*fide* Löbl et al. 2008b: 274).

***Scotera* Dejean, 1834: 182**

Originally included available species: none.

***Selenomma* Dejean, 1834: 183** (as “Selenomma. *Solier*.”)

Originally included available species: none.

***Selenepistoma* Dejean, 1834: 190** (as “Selenepistoma. *Solier*.”)

Originally included available species: *Opatrum acutum* Wiedemann, 1823; *Opatrum longipalpe* Wiedemann, 1823.

Type species: *Opatrum acutum* Wiedemann, 1823 by **present designation**.

Current status: junior subjective synonym of *Eurynotus* Kirby, 1819 in Tenebrionidae (*fide* Gebien 1938: 295, as “*Selenepistoma* M[u]ls[ant] & R[ey]”).

***Stenholma* Dejean, 1834: 184** (as “Stenholma. *Solier*.”)

Originally included available species: none.

***Tetromma* Dejean, 1834: 183** (as “Tetromma. *Solier*.”)

Originally included available species: *Akis laevigata* Fabricius, 1801; *Tentyria minuta* Tauscher, 1812; *Tagenia striatopuncatata* Wiedemann, 1821; *Upis unicolor* Herbst, 1797.

Type species: *Upis unicolor* Herbst, 1797 by subsequent designation (Löbl et al. 2008a: 43).

Current status: junior subjective synonym of *Hyperops* Eschscholtz, 1831 in Tenebrionidae (*fide* Löbl et al. 2008b: 192).

***Thalpophila* Dejean, 1834: 185** (as “Thalpophila. *Solier*.”)

Originally included available species: none.

***Trigonoscelis* Dejean, 1834: 179** (as “Trigonoscelis. *Solier*.”)

Originally included available species: *Pimelia deplanata* Krynicki, 1832 (as “Deplanata. *Zoubkoff*.”); *Pimelia nodosa* Fischer von Waldheim, 1820.

Type species: *Pimelia nodosa* Fischer von Waldheim, 1820 by subsequent designation (Hope 1840: 118).

Current status: valid genus in Tenebrionidae (*fide* Löbl et al. 2008b: 171).

***Zophius* Dejean, 1834: 189**

Originally included available species: *Helops rufopictus* Wiedemann, 1823.

Type species: *Helops rufopictus* Wiedemann, 1823 by monotypy.

Current status: valid genus in Tenebrionidae (*fide* Gebien 1942b: 757, as “*Zophius* Brême, 1842”).

***Zophobius* Dejean, 1834: 180**

Originally included available species: none.

Comments. The sole species included by Dejean (1834: 180) in this genus was described earlier by Lacordaire (1830: 286) as “*N… erotyloides*” where “*N…*” possibly stands for “nouveau” [new] but unnamed genus, not *Nyctelia* as recorded by Sherborn in his *Index Animalium*. According to Article 11.9.3 (ICZN 1999), a species-group name must be published in unambiguous combination with a generic name. This is not the case and so Lacordaire’s species is considered unavailable.

### Hétéromères: Taxicornes

***Aniara* Dejean, 1834: 199**

Originally included available species: none.

***Anisocheira* Dejean, 1834: 197**

Originally included available species: none.

***Anisocrepis* Dejean, 1834: 198**

Originally included available species: none.

***Apsida* Dejean, 1834: 197**

Originally included available species: none.

***Basanus*** **Dejean, 1834: 197**

Originally included available species: none.

***Calymmus* Dejean, 1834: 195**

Originally included available species: none.

***Cataphronetis* Dejean, 1834: 199**

Originally included available species: none.

***Cerandria* Dejean, 1834: 200**

Originally included available species: *Trogosita cornuta* Fabricius, 1798; *Trogosita maxillosa* Fabricius, 1801.

Type species: *Trogosita cornuta* Fabricius, 1798 by subsequent designation (Duponchel 1842: 285).

Current status: junior synonym of *Gnatocerus* Thunberg, 1814 in Tenebrionidae (*fide* Löbl et al. 2008b: 308).

***Cheirodes*** **Dejean, 1834: 194**

Originally included available species: none.

***Cosmonota* Dejean, 1834: 197**

Originally included available species: none.

***Delognatha* Dejean, 1834: 200**

Originally included available species: none.

***Eleoma* Dejean, 1834: 195** (as “Eleoma. *Ziegler*.”)

Comments. This name was listed by Dejean as an invalid synonym of *Lithophilus* Frölich, 1799. It has not been treated before 1961 as an available name and adopted as the name of a taxon or treated as a senior homonym and therefore *Eleoma* Dejean, 1834 is unavailable. *Eleoma* was first proposed, also as an invalid synonym of *Lithophilus*, by Fischer von Waldheim (1829: 73).

***Endophloeus*** **Dejean, 1834: 195**

Originally included available species: *Eledona spinosula* Latreille, 1807.

Type species: *Eledona spinosula* Latreille, 1807 (= *Sylpha markovichiana* Piller and Mitterpacher, 1783) by monotypy.

Current status: valid genus in Zopheridae (*fide* Ślipiński and Schuh 2008: 82).

***Epicalla* Dejean, 1834: 197**

Originally included available species: none.

***Epicamptus* Dejean, 1834: 198**

Originally included available species: none.

***Epilampus* Dejean, 1834: 198** (as “Epilampus. *Dalman*.”)

Comments. This name is treated as an unnecessary replacement name for *Ceropria* Laporte and Brullé, 1831 [Tenebrionidae].

***Eucyrtus* Dejean, 1834: 198**

Originally included available species: none.

***Eunotus*** **Dejean, 1834: 198**

Originally included available species: none.

***Heterocheira* Dejean, 1834: 199**

Originally included available species: none.

***Heterophaga* Dejean, 1834: 199**

Originally included available species: *Tenebrio chrysomelinus* Herbst, 1799 (as “Chrysomelina. *Fabr*.”); *Tenebrio diaperinus* Panzer, 1796 (as “*Diaperina. Sahlberg*.”); *Tenebrio mauritanicus* Fabricius, 1792.

Type species: *Tenebrio mauritanicus* Fabricius, 1792 (= *Opatrum laevigatum* Fabricius, 1781) by subsequent designation (Duponchel 1845: 601).

Current status: junior synonym of *Alphitobius* Stephens, 1829 in Tenebrionidae (*fide* Löbl et al. 2008b: 214).

***Hylonoma* Dejean, 1834: 199**

Originally included available species: none.

***Hypogena* Dejean, 1834: 199**

Originally included available species: *Tenebrio biimpressus* Latreille, 1833.

Type species: *Tenebrio biimpressus* Latreille, 1833 by monotypy.

Current status: valid genus in Tenebrionidae (*fide* Aalbu et al. 2002a: 497).

Comments. The name “Tricornis. *P*[alisot de].*B*[eauvois].” listed from “Amer. bor.” by Dejean (1834: 199) is not considered the same as *Phaleria tricornis* Dalman, 1823 described from specimens collected in Jamaica.

***Hypsoderes* Dejean, 1834: 195**

Originally included available species: none.

***Margus*** **Dejean, 1834: 200**

Originally included available species: *Colydium castaneum* Herbst, 1797 (as “*Castaneus. Sch*.”); *Tenebrio ferrugineus* Fabricius, 1787.

Type species: *Tenebrio ferrugineus* Fabricius, 1787 (= *Colydium castaneum* Herbst, 1797) by monotypy.

Current status: junior objective synonym of *Tribolium* MacLeay, 1825 in Tenebrionidae (*fide* Löbl et al. 2008b: 301).

Comments: The name *castaneus* is listed in synonymy with *ferrugineus* in Dejean’s catalogue; therefore the type species of *Margus* is *ferrugineus* by monotypy (ICZN 1999: Article 68.3).

***Phloeonemus* Dejean, 1834: 195**

Originally included available species: none.

***Phtora* Dejean, 1834: 200**

Originally included available species: none.

***Phylethus* Dejean, 1834: 196** (as “Phylethus. *Megerle*.”)

Originally included available species: none.

***Platycrepis* Dejean, 1834: 198** (as “Platycrepis. *Eschscholtz*.”)

Originally included available species: none.

***Scaptes* Dejean, 1834: 194** (as “Scaptes. *Eschscholtz*.”)

Originally included available species: none.

***Xyloborus* Dejean, 1834: 201**

Originally included available species: none.

### Hétéromères: Ténébrionites

***Anaedus* Dejean, 1834: 206**

Originally included available species: none.

***Anthracias* Dejean, 1834: 205** (as “Anthracias. *Stéven*.”)

Originally included available species: *Uloma cornuta* Fischer von Waldheim, 1823.

Type species: *Uloma cornuta* Fischer von Waldheim, 1823 by monotypy.

Current status: junior subjective synonym of *Cryphaeus* Klug, 1833 in Tenebrionidae (*fide* Löbl et al. 2008b: 300).

***Aspisoma* Dejean, 1834: 206**

Originally included available species: none.

***Baryscelis* Dejean, 1834: 204** (as “Baryscelis. *Boisduval*.”)

Originally included available species: none.

***Bius* Dejean, 1834: 205**

Originally included available species: *Trogosita thoracica* Fabricius, 1792.

Type species: *Trogosita thoracica* Fabricius, 1792 by monotypy.

Current status: valid genus in Tenebrionidae (*fide* Löbl et al. 2008b: 299).

***Bucerus* Dejean, 1834: 203**

Originally included available species: none.

***Camptobrachys* Dejean, 1834: 206**

Originally included available species: none.

***Centronipus*** **Dejean, 1834: 205**

Originally included available species: none.

***Charinotus* Dejean, 1834: 206**

Originally included available species: none.

***Chariotheca* Dejean, 1834: 206**

Originally included available species: none.

***Cholipus* Dejean, 1834: 206**

Originally included available species: none.

***Dendronomus* Dejean, 1834: 205**

Originally included available species: none.

***Eleutheris* Dejean, 1834: 206**

Originally included available species: none.

***Euphron* Dejean, 1834: 206**

Originally included available species: *Helops coerulescens* Guérin-Méneville, 1831 (as “coerulescens. *d’Urville*.”).

Type species: *Helops coerulescens* Guérin-Méneville, 1831 by monotypy.

Current status: senior subjective synonym of *Derosphaerus* Thomson, 1858 in Tenebrionidae (**new synonymy**).

Comments. *Euphron* Dejean, 1834 has precedence over *Derosphaerus* Thomson, 1858 which is currently used as valid (e.g., Löbl et al. 2008b: 340). Reversal of Precedence (ICZN 1999: Article 23.9) or an application to the Commission is necessary to conserve usage of the name *Derosphaerus* Thomson, 1858.

The type species, *Helops coerulescens*, is described in Boisduval (1835: 269, as “*Helops caerulescens* d’Urville”) who referred to *Helops coerulescens* Guérin-Méneville (1831: pl. 5, fig. 3).

***Geoborus* Dejean, 1834: 203**

Originally included available species: none.

***Haemerophygus* Dejean, 1834: 205**

Originally included available species: none.

***Hylobates* Dejean, 1834: 204**

Originally included available species: none.

***Hylocurus* Dejean, 1834: 203**

Originally included available species: none.

***Hypocalis* Dejean, 1834: 206**

Originally included available species: *Hemicera arcuata* Laporte and Brullé, 1831.

Type species: *Hemicera arcuata* Laporte and Brullé, 1831 by monotypy.

Current status: valid genus in Tenebrionidae (*fide* Gebien 1942a: 333, as “*Hypocalis* Lac[ordaire, 1859]”).

***Ichthydion* Dejean, 1834: 202**

Originally included available species: none.

***Imatismus*** **Dejean, 1834: 202**

Originally included available species: *Helops fasciculatus* Fabricius, 1798; *Stenosis orientalis* Herbst, 1799.

Type species: *Helops fasciculatus* Fabricius, 1798 by monotypy.

Current status: valid genus in Tenebrionidae (*fide* Löbl et al. 2008b: 193).

Comments. The name *orientalis* is listed in synonymy with *fasciculatus* in Dejean’s catalogue; therefore the type species of *Imatismus* is *fasciculatus* by monotypy (ICZN 1999: Article 68.3).

***Iphicerus* Dejean, 1834: 203**

Originally included available species: none.

***Iphius* Dejean, 1834: 203**

Originally included available species: *Tenebrio serratus* Fabricius, 1775.

Type species: *Tenebrio serratus* Fabricius, 1775 by monotypy.

Current status: junior homonym of *Iphius* Schoenherr, 1823 [Curculionidae]; senior subjective synonym of *Prioscelis* Hope, 1840 in Tenebrionidae (*fide* Gemminger and Harold 1870: 1991).

***Iphthinus* Dejean, 1834: 203**

Originally included available species: *Blaps clypeatus* Germar, 1813; *Upis chrysops* Herbst, 1797; *Tenebrio gigas* Linnaeus, 1763 (as “Gigas. *Fabr*.”); *Tenebrio impressus* Fabricius, 1801; *Upis maxima* Germar, 1824; *Helops punctatus* Fabricius, 1801; *Helops sinuatus* Fabricius, 1801; *Tenebrio valgus* Wiedemann, 1823; *Tenebrio variolosus* DeGeer, 1775 (as “Variolosus. *Fabr*.”).

Type species: *Tenebrio gigas* Linnaeus, 1763 by subsequent designation (Spilman 1973: 42).

Current status: junior objective synonym of *Mylaris* Pallas, 1781 in Tenebrionidae (*fide* Spilman 1973: 42).

***Mycetoma* Dejean, 1834: 201** (as “Mycetoma. *Ziegler*.”)

Originally included available species: *Dryops suturalis* Panzer, 1797.

Type species: *Dryops suturalis* Panzer, 1797 by monotypy.

Current status: valid genus in Tetratomidae (*fide* Nikitsky 2008: 64).

***Oligorus* Dejean, 1834: 206**

Originally included available species: *Tagenia indica* Wiedemann, 1823.

Type species: *Tagenia indica* Wiedemann, 1823 by monotypy.

Current status: junior subjective synonym of *Luprops* Hope, 1833 in Tenebrionidae (*fide* Löbl et al. 2008b: 119).

***Oplomerus* Dejean, 1834: 206**

Originally included available species: none.

***Pezodontus* Dejean, 1834: 203**

Comments. This name is treated as a replacement name for *Odontopus* Silbermann, 1833 [Tenebrionidae], a junior homonym of *Odontopus* Say, 1831 [Curculionidae] and *Odontopus* Laporte, 1832 [Hemiptera]. Therefore *Pezodontus* Dejean, 1834 is a **new objective synonym** of *Odontopezus* Alluaud, 1889, also a replacement name for *Odontopus* Silbermann, 1833. *Odontopezus* Alluaud, 1889 is currently the valid name (see Mal 1985) but *Pezodontus* Dejean, 1834 is older and has priority. Reversal of Precedence (ICZN 1999: Article 23.9) or an application to the Commission is necessary to conserve usage of the name *Odontopezus* Alluaud, 1889.

***Phobelius* Dejean, 1834: 203**

Originally included available species: none.

***Phymatodes* Dejean, 1834: 203**

Originally included available species: *Lagria tuberculata* Fabricius, 1792.

Type species: *Lagria tuberculata* Fabricius, 1792 by monotypy.

Current status: name suppressed in Tenebrionidae.

Comment. *Phymatodes* Dejean, 1834 was suppressed for the purposes of the Principles of Homonymy and Priority in Opinion 1525 (ICZN 1989a).

***Plateia* Dejean, 1834: 204** (as “Plateia. *De Haan*.”)

Originally included available species: none.

***Zophobas*** **Dejean, 1834: 204**

Originally included available species: *Helops morio* Fabricius, 1777; *Tenebrio nigritus* Olivier, 1795; *Helops opacus* Sahlberg, 1823; *Tenebrio quadrimaculata* Olivier, 1795.

Type species: *Helops morio* Fabricius, 1777 (= *Tenebrio atratus* Fabricius, 1775) by subsequent designation (Motschulsky 1872: 26).

Current status: valid genus in Tenebrionidae (*fide* Aalbu et al. 2002a: 498, as “*Zophobas* Blanchard, 1845”).

Comments. We have followed the intrepation of Ferrer (2006: 235) regarding the concept of *Helops morio* Fabricius, 1777.

### Hétéromères: Hélopiens

***Adelphus* Dejean, 1834: 208**

Originally included available species: *Helops beniniensis* Palisot de Beauvois, 1811; *Helops marginatus* Fabricius, 1792.

Type species: *Helops marginatus* Fabricius, 1792 by **present designation**.

Current status: senior subjective synonym of *Praeugena* Laporte, 1840 in Tenebrionidae (*fide* Gemminger and Harold 1870: 2039).

Comments. *Adelphus* Dejean, 1834 has precedence over *Praeugena* Laporte, 1840 which is currently used as valid (e.g., De Moor 1975: 42). Reversal of Precedence (ICZN 1999: Article 23.9) or an application to the Commission is necessary to conserve usage of the name *Praeugena* Laporte, 1840.

***Agapetus* Dejean, 1834: 212**

Originally included available species: none.

***Amacarus* Dejean, 1834: 212**

Originally included available species: none.

***Anorops* Dejean, 1834: 210**

Originally included available species: *Helops obliquatus* Fabricius, 1798.

Type species: *Helops obliquatus* Fabricius, 1798 by monotypy.

Current status: senior synonym of *Penthe* Newman, 1838 in Tetratomidae (*fide* Bouchard and Pollock 2009: 66).

Comments. *Anorops* Dejean, 1834is a *nomen oblitum* and *Penthe* Newman, 1838 a *nomen protectum* following Bouchard and Pollock (2009: 66).

***Atractus* Dejean, 1834: 212** (as “Atractus. *Mac Leay*.”)

Originally included available species: none.

***Cymatothes* Dejean, 1834: 208**

Originally included available species: *Helops undatus* Fabricius 1792.

Type species: *Helops undatus* Fabricius 1792 (= *Erotylus nebulosus* Fabricius, 1781) by monotypy.

Current status: valid genus in Tenebrionidae (*fide* Aalbu et al. 2002a: 498).

***Dicyrtus* Dejean, 1834: 207**

Originally included available species: none.

***Eucamptus* Dejean, 1834: 208**

Originally included available species: none.

***Eupezus* Dejean, 1834: 211**

Originally included available species: *Helops longipes* Fabricius, 1781.

Type species: *Helops longipes* Fabricius, 1781 by monotypy.

Current status: valid genus in Tenebrionidae (*fide* Robiche, 2006: 382, as “*Eupezus* Blanchard, 1845”).

***Homocyrtus* Dejean, 1834: 211**

Comments. This name is interpreted here as a replacement name for *Cyphonotus* Guérin-Méneville, 1831 [Tenebrionidae], a junior homonym of *Cyphonotus* Fischer von Waldheim, 1823 [Scarabaeidae].

***Hybonotus* Dejean, 1834: 211**

Originally included available species: *Tetraphyllus formosus* Laporte and Brullé, 1831.

Type species: *Tetraphyllus formosus* Laporte and Brullé, 1831 by monotypy.

Current status: junior subjective synonym of *Tetraphyllus* Laporte and Brullé, 1831 in Tenebrionidae (*fide* Löbl et al. 2008b: 348).

***Nephodes* Dejean, 1834: 210**

Originally included available species: none.

***Omophlus* Dejean, 1834: 213** (as “Omophlus. *Megerle*.”)

Originally included available species: *Cistela arcuata* Gebler, 1829; *Cistela armillata* Brullé, 1832 (as “Armillatus. *Parreyss*.”); *Cistela coeruleus* Fabricius, 1787; *Cistela lepturoides* Fabricius, 1787; *Cistela nigripennis* Fabricius, 1792; *Cistela picipes* Fabricius, 1792; *Cistela pilicollis* Faldermann, 1832; *Cistela ruficollis* Fabricius, 1781.

Type species: *Cistela lepturoides* Fabricius, 1787 by subsequent designation (Solier 1835: 246).

Current status: valid genus in Tenebrionidae (*fide* Löbl et al. 2008b: 334).

***Oplocheirus* Dejean, 1834: 211**

Originally included available species: none.

***Penichrus* Dejean, 1834: 210**

Originally included available species: none.

***Phlaegmatus* Dejean, 1834: 208**

Originally included available species: none.

***Physocoelus* Dejean, 1834: 211**

Originally included available species: none.

***Poecilesthus* Dejean, 1834: 207** (as “Paecilesthus. *Dejean*.”)

Originally included available species: *Erotylus fasciatus* Fabricius, 1781; *Helops geniculatus* Germar, 1824; *Helops geometricus* Perty, 1832; *Helops suturalis* Germar, 1824.

Type species: *Erotylus fasciatus* Fabricius, 1781 by subsequent designation (Hope 1840: 133).

Current status: valid genus in Tenebrionidae (*fide* Aalbu et al. 2002b: 512).

Comments: *Poecilesthus*, an incorrect subsequent spelling first used by Dejean (1836b: 229), is in prevailing usage and so deemed to be the correct original spelling (ICZN 1999: Article 33.3.1).

***Saerangodes* Dejean, 1834: 208**

Originally included available species: none.

***Sphenosoma* Dejean, 1834: 212**

Comments. This name is treated as an unnecessary replacement name for *Acropteron* Perty, 1832 [Tenebrionidae].

***Talanus* Dejean, 1834: 211**

Originally included available species: none.

***Thecacerus*** **Dejean, 1834: 207**

Originally included available species: none.

### Hétéromères: Trachélides

***Acosmus* Dejean, 1834: 218**

Originally included available species: none.

***Eutrapela*** **Dejean, 1834: 215**

Originally included available species: *Crioceris elongata* Fabricius, 1781; *Helodes porrecta* Fabricius, 1801.

Type species: *Crioceris elongata* Fabricius, 1781 (= *Chrysomela unifasciata* DeGeer, 1778) by subsequent designation (Duponchel 1845: 533).

Current status: junior homonym of *Eutrapela* Hübner, 1809 [Lepidoptera]; valid genus in Tenebrionidae (*fide* Borchmann 1936: 202, as “*Eutrapela* Blanch[ard]”).

Comments: Borchmann (1910: 14) reported that the use of this genus name in Lepidoptera predated the use in Tenebrionidae but expressed doubts about the availability of Hübner’s *Eutrapela* and therefore did not propose a replacement name. The geometrid name *Eutrapela* Hübner, 1809 is treated as valid in recent catalogues (e.g., Parsons et al. 1999: 385). To replace *Eutrapela* Dejean, 1834, we propose the name *Neoeutrapela*, **nomen novum**.

***Isotoma* Dejean, 1834: 214**

Originally included available species: none.

***Metoecus* Dejean, 1834: 218**

Originally included available species: *Mordella paradoxa* Linnaeus, 1760 (as “Paradoxus. *Fabr*.”).

Type species: *Mordella paradoxa* Linnaeus, 1760 by monotypy.

Current status: valid genus in Ripiphoridae (*fide* Batelka 2008: 77).

***Ochthenomus*** **Dejean, 1834: 217**

Originally included available species: none.

***Ptilophorus* Dejean, 1834: 218**

Originally included available species: *Pelecotoma dufouri* Latreille, 1817.

Type species: *Pelecotoma dufouri* Latreille, 1817 by monotypy.

Current status: valid genus in Ripiphoridae (*fide* Batelka 2008: 74).

***Trigonodera* Dejean, 1834: 217**

Originally included available species: *Pelecotoma leachi* Latreille, 1817.

Type species: *Pelecotoma leachi* Latreille, 1817 by monotypy.

Current status: valid genus in Ripiphoridae (*fide* Batelka 2008: 74).

### Hétéromères: Vésicants

***Causima* Dejean, 1834: 226**

Originally included available species: *Lytta vidua* Klug, 1825.

Type species: *Lytta vidua* Klug, 1825 by monotypy.

Current status: invalid subjective synonym of *Epicauta* Dejean, 1834 in Meloidae (*fide* Bologna 2008: 372).

***Dacnodes* Dejean, 1834: 226**

Originally included available species: none.

***Eletica* Dejean, 1834: 224**

Originally included available species: *Lytta rufa* Fabricius, 1801.

Type species: *Lytta rufa* Fabricius, 1801 (= *Lytta bipustulata* Fabricius, 1801) by monotypy.

Current status: valid genus in Meloidae (*fide* Bologna 2008: 370).

***Epicauta* Dejean, 1834: 224**

Originally included available species: *Lytta atomaria* Germar, 1821; *Lytta atrata* Fabricius, 1775; *Lytta bimaculata* Klug, 1825; *Lytta cinerea* Fabricius, 1798; *Lytta concinna* Klug, 1829 (as “Concinna. *Dej*.”); *Meloe erythrocephalus* Pallas, 1771 (as “Erythrocephala. *Fabr*.”); *Lytta geniculata* Klug, 1829; *Lytta gigas* Fabricius, 1792; *Lytta lemniscata* Fabricius, 1801; *Lytta lugubris* Klug, 1829; *Lytta megalocephala* Gebler, 1817; *Lytta melanocephala* Fabricius, 1801; *Cantharis melophtalmos* Olivier, 1795; *Lytta ochropus* Klug, 1829 (as “Ochropus. *Dej*.”); *Lytta oculata* Fabricius, 1792; *Lytta punctata* Germar, 1824; *Lytta ruficeps* Illiger, 1800; *Lytta ruficollis* Fabricius, 1792; *Meloe sibirica* Pallas, 1773; *Lytta strigosa* Gyllenhal, 1817 (as “Strigosa. *Schönherr*.”); *Lytta testacea* Fabricius, 1792; *Lytta villosa* Fabricius, 1798; *Lytta vittata* Fabricius, 1775.

Type species: *Meloe erythrocephalus* Pallas, 1771 by subsequent designation (Werner 1945: 425).

Current status: valid genus in Meloidae (*fide* Bologna 2008: 372).

Comments. The first type species designation for *Epicauta* Dejean is that of Duponchel (1844: 356) who selected *Lytta gigas* Fabricius, 1792 (= *Cantharis gigas* Olivier, 1790), one of the species originally included in the genus. This species is currently included in the genus *Cyaneolytta* Péringuey, 1909 (e.g., Selander 1986: 103) and its acceptance would bring significant nomenclatural changes. We believe that a request to the Commission to reject Duponchel’s designation is the best avenue.

***Pyrota* Dejean, 1834: 224**

Originally included available species: *Lytta afzeliana* Fabricius, 1801; *Lytta dispar* Germar, 1824; *Lytta herculeana* Germar, 1824; *Cantharis sinuata* Olivier, 1795.

Type species: *Lytta dispar* Germar, 1824 by subsequent designation (Aksentjev 1988: 578).

Current status: valid genus in Meloidae (*fide* Pinto and Bologna 2002: 526).

***Spastica* Dejean, 1834: 226**

Originally included available species: none.

***Synamma* Dejean, 1834: 221**

Originally included available species: none.

### Hétéromères: Sténélytres

***Anogcodes*** **Dejean, 1834: 228**

Originally included available species: *Necydalis collaris* Panzer, 1795; *Oedemera coarctata* Germar, 1824 (as “Coarctata. *Gebler*.”); *Cantharis fulvicollis* Scopoli, 1763 (as “Fulvicollis. *Fabr*.”); *Necydalis melanocephala* Fabricius, 1794; *Necydalis melanura* Fabricius, 1787; *Necydalis ruficollis* Fabricius, 1781; *Cantharis ustulata* Scopoli, 1763 (as “Ustulata. *Fabr*.”).

Type species: *Necydalis melanura* Fabricius, 1787 by subsequent designation (Randall 1838: 33).

Current status: valid genus in Oedemeridae (*fide* Švihla 2008: 361).

***Asclera*** **Dejean, 1834: 228**

Originally included available species: *Necydalis coerulescens* Fabricius, 1775; *Oedemera erythrocephala* Germar, 1824; *Necydalis notoxoides* Fabricius, 1801; *Necydalis sanguinicollis* Fabricius, 1787; *Necydalis thalassina* Fabricius, 1792; *Necydalis thoracica* Fabricius, 1801; *Cantharis viridissima* Linnaeus, 1758 (as “Viridissima. *Fabr*.”).

Type species: *Necydalis sanguinicollis* Fabricius, 1787 by subsequent designation (Arnett 1950: 219).

Current status: junior subjective synonym of *Ischnomera* Stephens, 1832 in Oedemeridae (*fide* Švihla 2008: 357).

***Ichnodes* Dejean, 1834: 227**

Originally included available species: none.

***Microps* Dejean, 1834: 228** (as “Microps. *Megerle*.”)

Comments. This name was listed by Dejean as an invalid synonym of *Ditylus* Fischer von Waldheim, 1817. It has not been treated before 1961 as an available name and adopted as the name of a taxon or treated as a senior homonym. Therefore *Microps* Dejean, 1834 is unavailable. *Microps* was first used as a valid name by Dahl (1823: 46) but his work was suppressed in Opinion 710 (ICZN 1964) for nomenclatural purposes.

***Nacerdes* Dejean, 1834: 228** (as Nacerdes. *Stéven*.)

Originally included available species: *Cantharis lepturoides* Thunberg, 1784 (as “*Lepturoides. Gyllenhal*.”); *Lagria livida* Fabricius, 1775; *Necydalis notata* Fabricius, 1792; *Oedemera pallipes* Olivier, 1811; *Oedemera suturalis* Olivier, 1811; *Lagria vittata* Fabricius, 1775.

Type species: *Necydalis notata* Fabricius, 1792 (= *Cantharis melanura* Linnaeus, 1758) by subsequent designation (Arnett 1950: 222).

Current status: valid genus in Oedemeridae (*fide* Švihla 2008: 362).

### Tétramères: Curculionites

***Acentrus*** **Dejean, 1835: 298** (as “Acentrus. *Chevrolat*.”)

Originally included available species: none.

***Aclees* Dejean, 1835: 276** (as “Aclees. *Schönherr*.”)

Originally included available species: none.

***Amalactus* Dejean, 1835: 276** (as “Amalactus. *Schönherr*.”)

Originally included available species: none.

***Anchylorhynchus*** **Dejean, 1835: 281** (as “Anchylorhynchus. *Klug*.”)

Originally included available species: none.

***Aporhina*** **Dejean, 1834: 240** (as “Aporhina. *Boisduval*.”)

Originally included available species: none.

***Apotomoderes*** **Dejean, 1834: 253** (as “Apotomoderes. *Mannerheim*.”)

Comments. This genus is treated as a replacement name for *Apotomus* Schönherr, 1834 [Curculionidae], a junior homonym of *Apotomus* Illiger, 1807 [Carabidae].

***Arhynchus*** **Dejean, 1835: 282**

Originally included available species: none.

***Atractomerus*** **Dejean, 1835: 280**

Originally included available species: none.

***Axestus*** **Dejean, 1835: 262**

Originally included available species: none.

***Botrobatys*** **Dejean, 1835: 294** (as “Botrobatys. *Chevrolat*.”)

Originally included available species: none.

***Brachypterus*** **Dejean, 1835: 289**

Originally included available species: none.

***Brachysoma* Dejean, 1834: 246**

Comments. This name was listed by Dejean as an invalid synonym of *Gonipterus* Schönherr, 1833. To our knowledge, it has not been treated before 1961 as an available name and adopted as the name of a taxon or treated as a senior homonym and therefore *Brachysoma* Dejean, 1834 is unavailable. *Brachysoma* was first used by Dejean (1821: 96) but not made available.

***Byrsopages* Dejean, 1835: 261** (as “Byrsopages. *Faldermann*.”)

Originally included available species: none.

***Camarhinus*Dejean, 1835: 280**

Originally included available species: none.

***Camptocheirus*** **Dejean, 1835: 279**

Originally included available species: none.

***Carpodes*** **Dejean, 1835: 301**

Originally included available species: none.

***Centemerus*** **Dejean, 1835: 277** (as “Centemerus. *Chevrolat*.”)

Originally included available species: none.

***Cephalosphaerus*** **Dejean, 1835: 287**

Originally included available species: none.

***Chalcodermus*** **Dejean, 1835: 297** (as “Chalcodermus. *Chevrolat*.”)

Originally included available species: *Rhynchaenus calidus* Fabricius, 1801; *Curculio metallinus* Fabricius, 1792.

Type species: *Rhynchaenus calidus* Fabricius, 1801 by subsequent designation (Schönherr 1837: 378).

Current status: valid genus in Curculionidae (*fide* Alonso-Zarazaga and Lyal 1999: 208).

***Chloropholus*** **Dejean, 1835: 263**

Originally included available species: none.

***Coccosomus*** **Dejean, 1835: 282**

Originally included available species: none.

***Coelostethus*** **Dejean, 1835: 287**

Originally included available species: none.

***Comasinus* Dejean, 1835: 283** (as “Comasinus. *Meg. Dej. Catal*.”)

Comments. This name was listed by Dejean as an invalid synonym of *Styphlus* Schönherr, 1826. It has not been treated before 1961 as an available name and adopted as the name of a taxon or treated as a senior homonym and therefore *Comasinus* Dejean, 1835 is unavailable. *Comasinus* was first used by Dejean (1821: 85) but not made available (see Alonso-Zarazaga and Lyal 1999: 84), notwithstanding Silfverberg’s (1984b: 61) comment.

***Conotrachelus* Dejean, 1835: 296** (as “Conotrachelus. *Latreille*.”)

Originally included available species: *Rhynchaenus concentricus* Olivier, 1807; *Balaninus diaconitus* Klug, 1829 (as “Diaconitus. *Germar*.”); *Curculio irroratus* Fabricius, 1787.

Type species: *Balaninus diaconitus* Klug, 1829 by subsequent designation (Schönherr 1837: 392).

Current status: valid genus in Curculionidae (*fide* Alonso-Zarazaga and Lyal 1999: 200).

***Corysosps* Dejean, 1835: 301**

Originally included available species: none.

***Cratocnemus*** **Dejean, 1835: 277**

Originally included available species: none.

***Cratoparis*** **Dejean, 1834: 235**

Comments. This name is treated as an unnecessary replacement name for *Euparius* Schönherr, 1823 [Anthribidae].

***Cyclopus* Dejean, 1834: 247**

Comments. This name was listed by Dejean as an invalid synonym of *Syzygops* Schönherr, 1826. To our knowledge, it has not been treated before 1961 as an available name and adopted as the name of a taxon or treated as a senior homonym and therefore *Cyclopus* Dejean, 1834 is unavailable. *Cyclopus* was first used by Dejean (1821: 96) but not made available.

***Cycnorhinus*** **Dejean, 1834: 235**

Originally included available species: none.

***Cyphipterus*** **Dejean, 1835: 271**

Originally included available species: none.

***Dactylocrepis*** **Dejean, 1835: 291**

Originally included available species: none.

***Desmidophorus*** **Dejean, 1835: 296** (as “Desmidophorus. *Chevrolat*.”)

Originally included available species: *Curculio hebes* Fabricius, 1781.

Type species: *Curculio hebes* Fabricius, 1781 by monotypy.

Current status: valid genus in Brachyceridae (*fide* Alonso-Zarazaga and Lyal 1999: 63).

***Diaprosomus*** **Dejean, 1834: 256**

Originally included available species: none.

***Diurus*** **Dejean, 1834: 244**

Originally included available species: *Ceocephalus furcillatus* Gyllenhal, 1833 (as “Furcillatus. *Chevrolat*.”).

Type species: *Ceocephalus furcillatus* Gyllenhal, 1833 (= *Ceocephalus furcillatus* Guérin-Méneville, 1833) by monotypy.

Current status: junior objective synonym of *Ceocephalus* Guérin-Méneville, 1833 in Brentidae (*fide* Alonso-Zarazaga and Lyal 1999: 54).

***Doryaspis*** **Dejean, 1835: 301** (as “Doryaspis. *Chevrolat*.”)

Originally included available species: none.

***Eudocinus* Dejean, 1835: 276** (as “Eudocinus. *Schönherr*.”)

Originally included available species: none.

***Eusomatus* Dejean, 1834: 249**

Comments. This name was listed by Dejean as an invalid synonym of *Eusomus* Germar, 1824. It has not been treated before 1961 as an available name and adopted as the name of a taxon or treated as a senior homonym and therefore *Eusomatus* Dejean, 1834 is unavailable. This name is treated as different from *Eusomatus* Krynicki, 1834 (see Alonso-Zarazaga and Lyal 1999: 177). The name was first used by Dejean (1821: 94) but not made available.

***Eutyrhinus*** **Dejean, 1835: 292** (as “Eutyrhinus. *Chevrolat*.”)

Originally included available species: *Curculio meditabundus* Fabricius, 1775.

Type species: *Curculio meditabundus* Fabricius, 1775 by monotypy.

Current status: valid genus in Curculionidae (*fide* Alonso-Zarazaga and Lyal 1999: 136).

***Glyphideres*** **Dejean, 1835: 301**

Originally included available species: none.

***Hadrotomus*** **Dejean, 1834: 253**

Originally included available species: none.

***Hypsophorus*** **Dejean, 1835: 292**

Originally included available species: *Cryptorhynchus dromedarius* Boisduval, 1835 (as “Dromedarius. *Dej*.”).

Type species: *Cryptorhynchus dromedarius* Boisduval, 1835 by monotypy.

Current status: valid genus in Curculionidae (*fide* Alonso-Zarazaga and Lyal 1999: 129).

Comments. Boisduval’s species was described before the fourth livraison of Dejean’s catalogue (see “Precedence” section [5]).

***Ichnorhinus*** **Dejean, 1835: 280**

Originally included available species: none.

***Ischnocerus*** **Dejean, 1834: 234** (as “Ischnocerus. *Chevrolat*.”)

Originally included available species: none.

***Ithyporus* Dejean, 1835: 284** (as “Ithyporus. *Schönherr*.”)

Originally included available species: none.

***Lagopezus*** **Dejean, 1834: 235**

Originally included available species: *Anthribus tenuicornis* Fabricius, 1801.

Type species: *Anthribus tenuicornis* Fabricius, 1801 by monotypy.

Current status: valid genus in Anthribidae (*fide* Alonso-Zarazaga and Lyal 1999: 35).

***Leptonemus* Dejean, 1834: 234**

Originally included available species: none.

***Leptoschoinus*** **Dejean, 1835: 291** (as “Leptoschoinus. *Klug*.”)

Originally included available species: *Baris fucata* Klug, 1829 (as “*Fucatus. Dej*.”).

Type species: *Baris fucata* Klug, 1829 by monotypy.

Current status: valid genus in Curculionidae (*fide* Alonso-Zarazaga and Lyal 1999: 95, as “*Leptoschoinus* Dejean, 1836”).

Comments. This name was first proposed by Schönherr (1833: 22) but not made available.

***Leucolopus* Dejean, 1835: 261**

Comments. This name was listed by Dejean as an invalid synonym of *Lophotus* Schönherr, 1834. To our knowledge, it has not been treated before 1961 as an available name and adopted as the name of a taxon or treated as a senior homonym and therefore *Leucolopus* Dejean is unavailable.

***Lignyodes* Dejean, 1835: 278** (as “Lignyodes. *Schönherr*.”)

Originally included available species: *Curculio enucluator* Panzer, 1798.

Type species: *Curculio enucluator* Panzer, 1798 by monotypy.

Current status: valid genus in Curculionidae (*fide* Alonso-Zarazaga and Lyal 1999: 85).

Comments. This name was also proposed the same year by Schönherr (1835: 323) but Dejean’s fourth livraison preceeds Schönherr’s book (see “Precedence” section [6]).

***Madopterus* Dejean, 1835: 289** (as “Madopterus. *Schönherr*.”)

Originally included available species: none.

Comments. This name was first proposed by Schönherr (1833: 23) but not made available. It was first made available by Schönherr (1836: 734).

***Micronyx* Dejean, 1835: 281** (as “Micronyx. *Schönherr*.”)

Originally included available species: none.

***Nemotrichus* Dejean, 1834: 234**

Originally included available species: none.

***Notosomalus*** **Dejean, 1835: 292** (as “Notosomalus. *Chevrolat*.”)

Originally included available species: none.

***Onchoscelis*** **Dejean, 1835: 294** (as “Onchoscelis. *Chevrolat*.”)

Originally included available species: none.

***Ophrylophus*** **Dejean, 1835: 264**

Originally included available species: none.

***Oplocnemus*** **Dejean, 1835: 277**

Originally included available species: none.

***Otideres*** **Dejean, 1835: 260**

Originally included available species: none.

***Pachydermus*** **Dejean, 1835: 283**

Originally included available species: none.

***Perolopus* Dejean, 1835: 282** (as “Perolopus. *Schönherr*.”)

Originally included available species: none.

***Petalochilus* Dejean, 1835: 286** (as “Petalochilus. *Schönherr*.”)

Originally included available species: none.

Comments. This name was first proposed by Schönherr (1833: 22) but not made available. It was first made available by Schönherr (1836: 591).

***Phiternus* Dejean, 1835: 281** (as “Phiternus. *Schönherr*.”)

Originally included available species: none.

***Phyllonomus*** **Dejean, 1835: 279**

Originally included available species: none.

***Phytophilus* Dejean, 1835: 277** (as “Phytophilus. *Schönherr*.”)

Originally included available species: none.

Comments. The sole species listed by Dejean (1835: 277) in this genus, *cruciferus* Eschscholtz, was described later in 1835 by Gyllenhal [in Schönherr] (see “Precedence” section [6]).

***Phytotribus*** **Dejean, 1835: 277**

Originally included available species: none.

***Piesocorynus* Dejean, 1834: 235** (as “Piesocorynus. *Chevrolat*.”)

Originally included available species: *Euparius dispar* Gyllenhal, 1833 (as “Dispar *Dej. Schönherr*.”).

Type species: *Euparius dispar* Gyllenhal, 1833 by monotypy.

Current status: valid genus in Anthribidae (*fide* Alonso-Zarazaga and Lyal 1999: 32).

***Pimelocerus* Dejean, 1835: 262**

Originally included available species: none.

***Plocamus*** **Dejean, 1835: 287**

Originally included available species: none.

***Prionomerus* Dejean, 1835: 279** (as “Prionomerus. *Schönherr*.”)

Originally included available species: none.

***Psomeles* Dejean, 1835: 271** (as “Psomeles. *Guérin*.”)

Originally included available species: *Otiorhynchus luctuosus* Boisduval, 1835 (as “Luctuosus. *d’Urville*.”); *Otiorhynchus melancholicus* Boisduval, 1835 (as “Melancholicus. *d’Urville*.”); *Otiorhynchus mutilatus* Boisduval, 1835 (as “Mutilatus. *d’Urville*.”).

Type species: *Otiorhynchus luctuosus* Boisduval, 1835 by subsequent designation (Chevrolat 1847b: 602).

Current status: valid genus in Curculionidae (*fide* Alonso-Zarazaga and Lyal 1999: 176).

Comments. Boisduval’s publication (1835) was issued earlier than the fourth livraison of Dejean’s catalogue (see “Precedence” section [5]). Therefore the three species-group names listed by Dejean (1835: 271) under *Psomeles* and credited to “d'Urville” were made available prior to Dejean's publication.

***Pteracanthus*** **Dejean, 1835: 277**

Originally included available species: *Rhynchaenus smidtii* Fabricius, 1801.

Type species: *Rhynchaenus smidtii* Fabricius, 1801 by monotypy.

Current status: valid genus in Curculionidae (*fide* Alonso-Zarazaga and Lyal 1999: 93).

***Pterodontus*** **Dejean, 1835: 280**

Originally included available species: none.

***Pyssematus*** **Dejean, 1835: 297** (as “Pyssematus. *Chevrolat*.”)

Originally included available species: none.

***Raphirhynchus*** **Dejean, 1834: 243** (as “Raphirhynchus. *Chevrolat*.”)

Originally included available species: *Brentus duplicatus* Germar, 1824; *Arrhenodes nitidicollis* Gyllenhal, 1833 (as “Nitidicollis. *Schönherr*.”).

Type species: *Arrhenodes nitidicollis* Gyllenhal, 1833 (= *Brentus cylindricornis* Fabricius, 1787) by subsequent designation (Schönherr 1840: 504).

Current status: valid genus in Brentidae (*fide* Alonso-Zarazaga and Lyal 1999: 49).

***Schimatocheilus* Dejean, 1834: 236** (as “Schimatocheilus. *Chevrolat*.”)

Originally included available species: none.

***Sporus*** **Dejean, 1835: 301**

Originally included available species: none.

***Stenops*** **Dejean, 1835: 299**

Originally included available species: none.

***Systellocerus*** **Dejean, 1834: 236**

Originally included available species: none.

***Systolus*** **Dejean, 1835: 283** (as “Systolus. *Megerle*.”)

Originally included available species: none.

***Taxicerus*** **Dejean, 1835: 289**

Originally included available species: none.

***Teinocorynus*** **Dejean, 1834: 243** (as “Teinocorynus. *Chevrolat*.”)

Originally included available species: none.

***Tophoderes*** **Dejean, 1834: 236**

Originally included available species: *Anthribus frenatus* Klug, 1833.

Type species: *Anthribus frenatus* Klug, 1833 by monotypy.

Current status: valid genus in Anthribidae (*fide* Alonso-Zarazaga and Lyal 1999: 34).

***Toxophorus* Dejean, 1835: 280** (as “Toxophorus. *Schönherr*.”)

Originally included available species: *Lixus attenuatus* Fabricius, 1801.

Type species: *Lixus attenuatus* Fabricius, 1801 by monotypy.

Current status: junior objective synonym of *Erodiscus* Schönherr, 1825 in Curculionidae (*fide* Alonso-Zarazaga and Lyal 1999: 78).

Comments. This name was also proposed the same year by Schönherr (1835: 371) but Dejean’s fourth livraison preceeds Schönherr’s book (see “Precedence” section [6]).

***Toxorhinus*** **Dejean, 1835: 280**

Originally included available species: none.

***Trachelizus*** **Dejean, 1834: 243** (as “Trachelizus. *Chevrolat*.”)

Originally included available species: *Brentus bisulcatus* Lund, 1800 (as “Bisulcatus. *Fabr*.”); *Arrhenodes pygmaeus* Gyllenhal, 1833 (as “Pygmaeus. *Schönherr*.”).

Type species: *Brentus bisulcatus* Lund, 1800 by subsequent designation (Schönherr 1840: 490).

Current status: valid genus in Brentidae (*fide* Alonso-Zarazaga and Lyal 1999: 53).

Comments. According to Alonso-Zarazaga and Lyal (1999: 56), *Trachelizus* Dejean, 1834 is preoccupied by another weevil name *Trachelizus* Gyllenhal, 1833, a name first proposed as a synonym.

***Trypetes* Dejean, 1835: 286** (as “Trypetes. *Schönherr*.”)

Originally included available species: none.

***Uterosomus*** **Dejean, 1834: 236** (as “Uterosomus. *Chevrolat*.”)

Originally included available species: *Anthribus scoparius* Klug, 1833; *Macrocephalus verrucosus* Olivier, 1795.

Type species: *Macrocephalus verrucosus* Olivier, 1795 by monotypy.

Current status: valid genus in Anthribidae (*fide* Alonso-Zarazaga and Lyal 1999: 34).

Comments: The name *scoparius* is listed in synonymy with *verrucosus* in Dejean’s catalogue; therefore the type species of *Uterosomus* is *verrucosus* by monotypy (ICZN 1999: Article 68.3).

### Tétramères: Xylophages

***Adelina*** **Dejean, 1835: 315** (as “Adelina. *Chevrolat*.”)

Originally included available species: *Cucujus planus* Fabricius, 1801.

Type species: *Cucujus planus* Fabricius, 1801 by monotypy.

Current status: valid genus in Tenebrionidae (*fide* Löbl et al. 2008b: 307).

***Biophloeus*** **Dejean, 1835: 315**

Originally included available species: *Cucujus dermestoides* Fabricius, 1792.

Type species: *Cucujus dermestoides* Fabricius, 1792, by monotypy.

Current status: senior objective synonym of *Pediacus* Shuckard, 1839 in Cucujidae (*fide* Gemminger and Harold 1868: 877).

Comments. *Biophloeus* Dejean, 1835 is a *nomen oblitum* and *Pediacus* Shuckard, 1839 a *nomen protectum* following Bouchard et al. (2011: 363).

***Bothrideres*** **Dejean, 1835: 312**

Originally included available species: *Ips contracta* Olivier, 1790 (as “Contractus. *Fabr*.”).

Type species: *Ips contracta* Olivier, 1790 by monotypy.

Current status: valid genus in Bothrideridae (*fide* Ślipiński 2007: 551).

***Camptognathus*** **Dejean, 1835: 315**

Originally included available species: none.

***Damicerus* Dejean, 1835: 308** (as “Damicerus. *Spinola*.”)

Originally included available species: none.

***Dendrophtorus*** **Dejean, 1835: 309**

Originally included available species: none.

***Epilophus* Dejean, 1835: 313**

Originally included available species: none.

***Eutomus* Dejean, 1835: 306**

Originally included available species: none.

***Gymnocheilis* Dejean, 1835: 314** (as “Gymnocheilis. *Gray*.”)

Originally included available species: *Trogosita squamosa* Gray, 1832.

Type species: *Trogosita squamosa* Gray, 1832 (= *Trogosita varia* Fabricius, 1801) by monotypy.

Current status: valid genus in Trogossitidae (*fide* Leschen and Lackner 2013).

Comments. The genus *Lepidopteryx* Hope, 1840 [type species: *Trogosita squamosa* Gray, 1832 by monotypy], incorrectly treated as the valid synonym of *Leperina* Erichson, 1844 recently (e.g., Kolibáč 2009: 127), is a junior objective synonym of *Gymnocheilis* Dejean, 1835. *Gymnochila* Erichson, 1844 [type species: *Trogosita vestita* Griffith, 1832 (= *Trogosita varia* Fabricius, 1801) by monotypy] is also a junior synonym of *Gymnocheilis* Dejean, 1835.

***Laemophloeus*** **Dejean, 1835: 315**

Originally included available species: *Cucujus bimaculatus* Olivier, 1795; *Cucujus ferrugineus* Stephens, 1831 (as “Ferrugineus. *Megerle*.”); *Cucujus monilis* Fabricius, 1787; *Cucujus muticus* Fabricius, 1781; *Cucujus pusillus* Schönherr, 1817; *Cucujus testaceus* Fabricius, 1787.

Type species: *Cucujus muticus* Fabricius, 1781 by subsequent designation (Thomson 1859: 83).

Current status: valid genus in Laemophloeidae (*fide* Wegrzynowicz 2007a: 504).

***Melalgus*** **Dejean, 1835: 309**

Originally included available species: *Apate femoralis* Fabricius, 1792 (as “*Femoralis. Olivier*.”); *Apate gonagra* Fabricius, 1798.

Type species: *Apate gonagra* Fabricius, 1798 by monotypy.

Current status: valid genus in Bostrichidae (*fide* Ivie 2002a: 239).

Comments. The name *femoralis* is listed in synonymy with *gonagra* in Dejean’s catalogue; therefore the type species of *Melalgus* is *gonagra* by monotypy (ICZN 1999: Article 68.3).

***Monopis*** **Dejean, 1835: 314** (as “Monopis. *Ziegler*.”)

Originally included available species: none.

***Nemicelus*** **Dejean, 1835: 315**

Originally included available species: none.

***Ogcoderes*** **Dejean, 1835: 313**

Originally included available species: none.

***Pathoderma*** **Dejean, 1835: 312**

Originally included available species: *Peltis orientalis* Wiedemann, 1821.

Type species: *Peltis orientalis* Wiedemann, 1821 by monotypy.

Current status: unknown.

Comments. We were unable to find any information regarding the type species of this genus.

***Rhagodera*** **Dejean, 1835: 312** (as “Rhagodera. *Eschscholtz*.”)

Originally included available species: none.

***Teredus*** **Dejean, 1835: 313**

Originally included available species: *Lyctus nitidus* Fabricius, 1792; *Ips cylindrica* Olivier, 1790.

Type species: *Lyctus nitidus* Fabricius, 1792 (= *Ips cylindrica* Olivier, 1790) by monotypy.

Current status: valid genus in Bothrideridae (*fide* Ślipiński 2007: 552).

Comments. The name *cylindricus* is listed in synonymy with *nitidus* in Dejean’s catalogue; therefore the type species of *Teredus* is *nitidus* by monotypy (ICZN 1999: Article 68.3).

***Xylographus*** **Dejean, 1835: 310**

Originally included available species: none.

***Xylolaemus*** **Dejean, 1835: 313**

Originally included available species: *Lyctus fasciculosus* Gyllenhal, 1827 (as “Fasciculosus. *Schönherr*.”).

Type species: *Lyctus fasciculosus* Gyllenhal, 1827 by monotypy.

Current status: valid genus in Zopheridae (*fide* Ślipiński and Schuh 2008: 87, as “*Xylolaemus* Redtenbacher, 1857”).

***Xylophtorus*** **Dejean, 1835: 312**

Originally included available species: none.

***Xylotrupes*** **Dejean, 1835: 310**

Originally included available species: none.

### Tétramères: Longicornes

***Acharidis* Dejean, 1835: 348**

Originally included available species: none.

***Acmocera* Dejean, 1835: 345**

Originally included available species: *Lamia compressa* Fabricius, 1801.

Type species: *Lamia compressa* Fabricius, 1801 by monotypy.

Current status: valid genus in Cerambycidae (*fide* Breuning and Teocchi 1980: 367, as “*Acmocera* Thomson, 1858”).

***Aegomorphus* Dejean, 1835: 337**

Originally included available species: none.

***Aegoprosopus* Dejean, 1835: 318**

Comments. This name is treated as an unnecessary replacement name for *Closterus* Audinet-Serville, 1832 [Cerambycidae].

***Aerenaea* Dejean, 1835: 344**

Originally included available species: none.

***Aerenica* Dejean, 1835: 352**

Originally included available species: *Saperda canescens* Klug, 1825.

Type species: *Saperda canescens* Klug, 1825 by monotypy.

Current status: valid genus in Cerambycidae (*fide* Monné and Bezark 2009: 233).

***Alcidion* Dejean, 1835: 338**

Originally included available species: none.

***Alphitopola* Dejean, 1835: 348**

Originally included available species: none.

***Alphus* Dejean, 1835: 337**

Originally included available species: none.

***Amallocerus* Dejean, 1835: 321**

Originally included available species: none.

***Amblesthis* Dejean, 1835: 341**

Originally included available species: none.

***Amniscus* Dejean, 1835: 338**

Originally included available species: *Acanthocinus incrassatus* Klug, 1829 (as “Incrassatus. *Dej*.”); *Lamia inermis* Fabricius, 1801; *Lamia praemorsa* Fabricius, 1792.

Type species: *Lamia praemorsa* Fabricius, 1792 by subsequent designation (Monné and Giesbert 1992: 253).

Current status: valid genus in Cerambycidae (*fide* Monné and Bezark 2009: 191).

***Amphionycha* Dejean, 1835: 352**

Originally included available species: *Saperda cirrata* Germar, 1824; *Saperda hemispila* Germar, 1821; *Saperda marginata* Fabricius, 1798; *Saperda nigromaculata* Klug, 1829 (as “Nigromaculata *Dej*.”); *Saperda triangularis* Germar, 1824.

Type species: *Saperda hemispila* Germar, 1821 by subsequent designation (Marinoni 1977: 40).

Current status: junior synonym of *Adesmus* Lepeletier and Audinet-Serville, 1825 in Cerambycidae (*fide* Monné and Bezark 2009: 279, as “*Amphyonycha* Dejean, 1835”).

***Anaesthetis* Dejean, 1835: 348**

Originally included available species: *Saperda testacea* Fabricius, 1781.

Type species: *Saperda testacea* Fabricius, 1781 by monotypy.

Current status: valid genus in Cerambycidae (*fide* Adlbauer et al. 2010: 221).

***Anaetia* Dejean, 1835: 350**

Originally included available species: *Leptura praeusta* Linnaeus, 1758 (as “Praeusta. *Fabr*.”).

Type species: *Leptura praeusta* Linnaeus, 1758 by monotypy.

Current status: junior objective synonym of *Tetrops* Stephens, 1829 in Cerambycidae (*fide* Adlbauer et al. 2010: 332).

***Anancylus* Dejean, 1835: 341**

Originally included available species: none.

***Ancistroderus* Dejean, 1835: 341**

Originally included available species: none.

***Ancylonotus* Dejean, 1835: 335**

Originally included available species: *Lamia tribulus* Fabricius, 1775.

Type species: *Lamia tribulus* Fabricius, 1775 by monotypy.

Current status: valid genus in Cerambycidae (*fide* Aurivillius 1922: 153, as “Ancylonotus Cast[elnau]”).

***Anhammus* Dejean, 1835: 341** (as “Anhammus. *Dupont*.”)

Originally included available species: none.

***Anisarthron* Dejean, 1835: 331**

Originally included available species: *Cerambyx barbipes* Schrank, 1781 (as “Barbipes. *Dahl*.”).

Type species: *Cerambyx barbipes* Schrank, 1781 by monotypy.

Current status: valid genus in Cerambycidae (*fide* Adlbauer et al. 2010: 137).

***Anoplomerus* Dejean, 1835: 326**

Originally included available species: none.

***Anoplosthaeta* Dejean, 1835: 341**

Originally included available species: *Lamia lactator* Fabricius, 1801.

Type species: *Lamia lactator* Fabricius, 1801 by monotypy.

Current status: senior subjective synonym of *Prosopocera* Blanchard, 1845 in Cerambycidae (*fide* Adlbauer et al. 2010: 313).

Comments. *Anoplosthaeta* Dejean, 1835 was qualified as *nomen oblitum* by Sama (2009: 24) but the Reversal of Precedence (ICZN 1999, Article 23.9) cannot be used to suppress *Anoplosthaeta* in favour of *Prosopocera* since *Anoplosthaeta* was used as valid after 1899 (e.g., Aurivillius 1907: 21, as *Anoplostetha*). An application to the Commission is necessary to preserve *Prosopocera*, the type genus of the valid tribe Prosopocerini Thomson, 1864.

***Aphanasium* Dejean, 1835: 322**

Originally included available species: *Callidium australe* Boisduval, 1835 (as “Australe. *Dej*.”).

Type species: *Callidium australe* Boisduval, 1835 by monotypy.

Current status: valid genus in Cerambycidae (*fide* McKeown 1947: 51, as “*Aphanasium* Thomson, 1860”).

Comments. Boisduval’s species was described earlier than the fourth livraison of Dejean’s catalogue (see “Precedence” section [5]).

***Aphies* Dejean, 1835: 353**

Originally included available species: none.

***Aplectrus* Dejean, 1835: 330**

Originally included available species: none.

***Asemnis* Dejean, 1835: 350**

Originally included available species: none.

***Astynomus* Dejean, 1835: 337**

Comments. This name is treated as anunnecessary replacement name for *Aedilis* Audinet-Serville, 1835 [Cerambycidae]. Audinet-Serville’s publication (1835a), where the name *Aedilis* was made available, appeared before the fourth livraison of Dejean’s catalogue (see “Precedence” section [7]).

***Atelodesmis* Dejean, 1835: 348**

Originally included available species: none.

***Axinopalpis* Dejean, 1835: 332**

Originally included available species: *Obrium gracile* Krynicki, 1832 (as “Gracilis. *Ziegler*.”).

Type species: *Obrium gracile* Krynicki, 1832 by monotypy.

Current status: valid genus in Cerambycidae (*fide* Adlbauer et al. 2010: 184).

***Batocera* Dejean, 1835: 341**

Originally included available species: *Lamia octomaculata* Fabricius, 1792; *Cerambyx rubus* Linnaeus, 1758 (as “Rubus. *Fabr*.”).

Type species: *Cerambyx rubus* Linnaeus, 1758 by subsequent designation (Blanchard 1845: 175).

Current status: valid genus in Cerambycidae (*fide* Adlbauer et al. 2010: 238).

***Batrachorhina* Dejean, 1835: 345**

Originally included available species: none.

***Bebelis* Dejean, 1835: 349**

Originally included available species: none.

***Cacostola* Dejean, 1835: 349**

Originally included available species: none.

***Callimation* Dejean, 1835: 342**

Originally included available species: none.

***Carterica* Dejean, 1835: 352**

Originally included available species: none.

***Centrocerum* Dejean, 1835: 330**

Originally included available species: none.

***Cephalophis* Dejean, 1835: 318** (as “Cephalophis. *Dupont*.”)

Originally included available species: none.

***Ceropogon* Dejean, 1835: 328**

Originally included available species: *Cerasphorus hirticornis* Audinet-Serville, 1834.

Type species: *Cerasphorus hirticornis* Audinet-Serville, 1834 by monotypy.

Current status: junior synonym of *Cerasphorus* Audinet-Serville, 1834 in Cerambycidae (*fide* Gemminger and Harold 1872: 2811).

***Cerosterna* Dejean, 1835: 341**

Originally included available species: *Lamia gladiator* Fabricius, 1801; *Lamia punctator* Fabricius, 1777; *Lamia reticulator* Fabricius, 1781; *Lamia scabrator* Fabricius, 1781 (as “*Scabrator. Olivier*.”).

Type species: *Lamia gladiator* Fabricius, 1801 (= *Lamia scabrator* Fabricius, 1781) by subsequent designation (Thomson 1864: 75).

Current status: valid genus in Cerambycidae (*fide* Adlbauer et al. 2010: 279).

***Chaetosoma* Dejean, 1835: 340**

Originally included available species: none.

***Choeromorpha* Dejean, 1835: 343**

Originally included available species: none.

***Cloniocerus* Dejean, 1835: 340**

Originally included available species: *Lamia hystrix* Fabricius, 1781.

Type species: *Lamia hystrix* Fabricius, 1781 by monotypy.

Current status: valid genus in Cerambycidae (*fide* Breuning 1950: 416, as “*Cloniocerus* Cast[elnau], 1840”).

***Closteromerus* Dejean, 1835: 324**

Originally included available species: *Saperda sexpunctata* Fabricius, 1792.

Type species: *Saperda sexpunctata* Fabricius, 1792 by monotypy.

Current status: senior objective synonym of *Hylomela* Gahan, 1904 in Cerambycidae (*fide* Veiga Ferreira 1966: 721, as “*Closteromerus* Lacordaire, 1869”).

Comments. *Closteromerus* Dejean, 1835 has precedence over *Hylomela* Gahan, 1904 which is currently used as valid (e.g., Veiga Ferreira 1966: 721). If the name *Hylomela* Gahan is to be conserved as valid, an application to the Commission is necessary.

The name *Closteromerus* has often been credited to Thomson (1861: 169) in a different taxonomic sense than that of Dejean (e.g., Veiga Ferreira 1966: 678). The name *Homaloceraea* Schmidt, 1922 is available for *Closteromerus sensu* Thomson (1861: 169).

***Closteropus* Dejean, 1835: 324**

Originally included available species: none.

***Coccoderus* Dejean, 1835: 321**

Originally included available species: none.

***Corethrogaster* Dejean, 1835: 328**

Originally included available species: none.

***Cosmocerus* Dejean, 1835: 321**

Originally included available species: none.

***Cosmotoma* Dejean, 1835: 338**

Originally included available species: none.

***Criocephalum* Dejean, 1835: 328**

Originally included available species: *Cerambyx rusticus* Linnaeus, 1758 (as “Rusticum. *Fabr*.”).

Type species: *Cerambyx rusticus* Linnaeus, 1758 by monotypy.

Current status: junior objective synonym of *Arhopalus* Audinet-Serville, 1834 in Cerambycidae (*fide* Adlbauer et al. 2010: 137).

***Criomorphus* Dejean, 1835: 337**

Originally included available species: none.

***Cyclopeplus* Dejean, 1835: 335**

Originally included available species: none.

***Cyrtognathus* Dejean, 1835: 316**

Originally included available species: *Prionus paradoxus* Faldermann, 1833.

Type species: *Prionus paradoxus* Faldermann, 1833.

Current status: valid genus in Cerambycidae (*fide* Drumont and Komiya 2010: 91, as “*Cyrtognathus* Faldermann, 1835”).

Comments. This name was proposed the same year by both Dejean (1835: 316) and Faldermann (1835: 431). As indicated in the “Precedence” section [10], Dejean’s name has priority.

***Delocheilus* Dejean, 1835: 319**

Originally included available species: none.

***Deltosoma* Dejean, 1835: 321**

Originally included available species: none.

***Deroplia* Dejean, 1835: 348**

Originally included available species: *Saperda genei* Aragona, 1830 (as “Genei. *Chevrolat*.”).

Type species: *Saperda genei* Aragona, 1830 by monotypy.

Current status: valid genus in Cerambycidae (*fide* Adlbauer et al. 2010: 222).

***Diastocera* Dejean, 1835: 342**

Originally included available species: *Lamia trifasciata* Fabricius, 1775.

Type species: *Lamia trifasciata* Fabricius, 1775 by monotypy.

Current status: valid genus in Cerambycidae (*fide* Danilevsky 2011: 321).

Comments. *Diastocera* Dejean was placed on the Official List of Generic Names in Zoology in Opinion 1407 (ICZN 1986).

***Dicranoderes* Dejean, 1835: 320** (as “Dicranoderes. *Dupont*.”)

Originally included available species: none.

***Dicranops* Dejean, 1835: 322**

Originally included available species: none.

***Dorcacephalum* Dejean, 1835: 345** (as “Dorcacephalum. *Dupont*.”)

Originally included available species: none.

***Dorcaschema* Dejean, 1835: 348**

Originally included available species: none.

***Entelopes* Dejean, 1835: 347**

Originally included available species: none.

***Erioderus* Dejean, 1835: 318**

Originally included available species: none.

***Euchaetes* Dejean, 1835: 340**

Originally included available species: none.

***Eudoxilus* Dejean, 1835: 323** (as “Eudoxilus. *Dupont*.”)

Originally included available species: none.

***Eumathes* Dejean, 1835: 348**

Originally included available species: none.

***Eurycephalus* Dejean, 1835: 323**

Originally included available species: *Cerambyx lundii* Fabricius, 1792; *Cerambyx maxillosus* Olivier, 1795; *Cerambyx nigripes* Olivier, 1795.

Type species: *Cerambyx maxillosus* Olivier, 1795 (= *Cerambyx lundii* Fabricius, 1792) by monotypy.

Current status: junior homonym of *Eurycephalus* Gray, 1832 [Cerambycidae]; senior synonym of *Euryphagus* Thomson, 1864 in Cerambycidae (*fide* Adlbauer et al. 2010: 197).

Comments. The names *lundii* and *nigripes* are listed in synonymy with *maxillosus* in Dejean’s catalogue; therefore the type species of *Eurycephalus* is *maxillosus* by monotypy (ICZN 1999: Article 68.3).

***Eurypygon* Dejean, 1835: 329**

Originally included available species: none.

***Euryscelis* Dejean, 1835: 331**

Originally included available species: *Callidium suturale* Olivier, 1795.

Type species: *Callidium suturale* Olivier, 1795 by monotypy.

Current status: valid genus in Cerambycidae (*fide* Monné and Bezark 2009: 44).

***Eusebis* Dejean, 1835: 349**

Originally included available species: none.

***Euteles* Dejean, 1835: 348**

Originally included available species: none.

***Eutheia* Dejean, 1835: 353** (as “Eutheia. *Reichenbach*.”)

Originally included available species: *Hippopsis filum* Klug, 1829.

Type species: *Hippopsis filum* Klug, 1829 by monotypy.

Current status: junior homonym of *Eutheia* Stephens, 1830 [Staphylinidae]; senior subjective synonym of *Spalacopsis* Newman, 1842 in Cerambycidae (*fide* Monné and Bezark 2009: 239).

***Eutrypanus* Dejean, 1835: 337**

Originally included available species: none.

***Evethis* Dejean, 1835: 349**

Originally included available species: none.

***Exocentrus* Dejean, 1835: 339** (as “Exocentrus. *Megerle*.”)

Originally included available species: *Cerambyx balteus* Linnaeus *sensu* Dejean, 1835 (as “Balteatus. *Fabr*.”); *Cerambyx crinitus* Panzer, 1795; *Cerambyx lusitanus* Linnaeus, 1767 (as “*Lusitanicus. Olivier*.”).

Type species: *Cerambyx balteus* Linnaeus *sensu* Dejean, 1835 (= *Cerambyx lusitanus* Linnaeus, 1767) by monotypy.

Current status: valid genus in Cerambycidae (*fide* Adlbauer et al. 2010: 309).

Comments. Except for *Cerambyx balteatus* DeGeer, 1775, a junior synonym of *Knulliana cincta* (Drury, 1773) described from Virginia, USA (Linsley 1962: 110), no other new species-group taxon was described under the name *Cerambyx balteatus* or *Lamia balteata* despite that such names are recorded in several works on the European fauna and attributed to Fabricius or Gyllenhal. The first use of *Cerambyx balteatus* in Europe appeared in Fabricius (1792: 262) who referred the species to Linnaeus and Olivier. Both of these authors actually described *Cerambyx balteus* Linnaeus, 1767. Therefore it is quite evident that Fabricius used an incorrect subsequent spelling for the name *balteus* Linnaeus. Audinet-Serville (1835: 59), who had most likely access to Dejean’s collection, reported that the *balteatus* in Dejean’s catalogue was a species of *Pogonocherus*. This species is actually *Exocentrus lusitanus* (Linnaeus, 1767) in the tribe Pogonocherini Mulsant, 1839 (Adlbauer et al. 2010: 310) while *Cerambyx balteus* Linnaeus, 1767 is a valid species in the genus *Parmena* Dejean of the tribe Parmenini Mulsant, 1839 (Adlbauer et al. 2010: 290).

The names *crinitus* and *lusitanicus* are listed in synonymy with *balteatus* in Dejean’s catalogue; therefore the type species of *Exocentrus* is *balteus sensu* Dejean by monotypy (ICZN 1999: Article 68.3).

***Gnaphalocera* Dejean, 1835: 349**

Originally included available species: none.

***Grammoptera* Dejean, 1835: 356** (as “Grammoptera. *Serville*. ”)

Originally included available species: *Leptura holosericea* Fabricius, 1801 [no 68]; *Leptura laevis* Fabricius, 1792; *Leptura lurida* Fabricius, 1792; *Leptura praeusta* Fabricius, 1787; *Leptura quadriguttata* Fabricius, 1787; *Leptura ruficornis* Fabricius, 1781.

Type species: *Leptura praeusta* Fabricius, 1787 (= *Leptura ustulata* Schaller, 1783) by subsequent designation (Westwood 1838: 41).

Current status: valid genus in Cerambycidae (*fide* Adlbauer et al. 2010: 101, as “*Grammoptera* Audinet-Serville, 1835”).

Comments. The name *Grammoptera* was proposed the same year by Dejean (1835: 356) and Audinet-Serville (1835: 215). As indicated in the “Precedence” section [8], Dejean’s publication has priority.

***Hammoderus* Dejean, 1835: 341**

Originally included available species: none.

***Hastatis* Dejean, 1835: 352**

Originally included available species: none.

***Hathlia* Dejean, 1835: 347**

Originally included available species: none.

***Hebecerus* Dejean, 1835: 336**

Originally included available species: *Acanthocinus australis* Boisduval, 1835 (as “Australis. *Dej*.”); *Acanthocinus marginicollis* Boisduval, 1835 (as “Marginicollis. *Dej*.”).

Type species: *Acanthocinus marginicollis* Boisduval, 1835 by **present designation**.

Current status: senior subjective synonym of *Ancita* Thomson, 1864 in Cerambycidae (*fide* McKeown 1947: 127, as “*Hebecerus* Thomson”).

Comments. *Hebecerus* Dejean, 1835 has precedence over *Ancita* Thomson, 1864 and should be used as valid. Reversal of Precedence (ICZN 1999, Article 23.9) or an application to the Commission is necessary to conserve usage of the name *Ancita* Thomson, 1864.

Boisduval’s publication was issued by 27 March 1835 (Evenhuis 1997: 104), so the two names listed in Dejean (1835: 336), which were credited to Dejean in Boisduval’s work, were made available prior to Dejean’s publication.

***Hebestola* Dejean, 1835: 348**

Originally included available species: none.

***Hesperophanes* Dejean, 1835: 328**

Originally included available species: *Callidium bimaculatum* Fabricius, 1781; *Callidium holosericeum* Rossi, 1790; *Callidium mixtum* Fabricius, 1798; *Callidium nebulosum* Olivier, 1790.

Type species: none validy designated.

Current status: valid genus in Cerambycidae (*fide* Adlbauer et al. 2010: 185).

Comments. *Callidium sericeum* Fabricius, 1787 is usually cited as type species of *Hesperophanes* Dejean (e.g., Thomson 1864: 253; Sama 2002: 49; Adlbauer et al. 2010: 185). However this taxon is a *species inquirendum* in Dejean’s catalogue and cannot be considered as an originally included species (ICZN 1999: Article 67.2.5). All available species originally included actually belong to genera other than *Hesperophanes*. An application to the Commission is necessary to retain *Callidium sericeum* Fabricius as type species of *Hesperophanes* Dejean.

***Hesycha* Dejean, 1835: 344**

Originally included available species: none.

***Heterogaster* Dejean, 1835: 332**

Originally included available species: *Callidium pilicorne* Fabricius *sensu* Olivier, 1795 (as “*Pilicorne. Olivier*.”).

Type species: *Callidium pilicorne* Fabricius *sensu* Olivier, 1795 (= *Heterogaster pilicornis* Dejean, 1835) by monotypy.

Current status: junior homonym of *Heterogaster* Schilling, 1829 [Hemiptera]; senior synonym of *Anisogaster* Deyrolle, 1862 in Cerambycidae (*fide* Gemminger and Harold 1872: 2836).

Comments. Dejean obviously retained the name *Callidium pilicorne* in the sense of a misidentification used by Olivier (1795 [70]: 68) since the true *Callidium pilicorne* Fabricius is included by Dejean (1835: 332) in his previous genus, *Onchomerus* Dejean. According to ICZN (1999: Article 11.10), an author, who employs a specific name for the type species of a new nominal genus-group taxon deliberately in the sense of a previous misidentification of it, is deemed to have denoted a new nominal species, with its own author and date as though it were newly proposed in combination with the new genus-group name. Therefore Dejean (1835: 332) indirectly proposed the name *Heterogaster pilicornis*, which is a senior synonym of *Anisogaster flavicans* Deyrolle, 1862 (**new synonymy**).

***Hetoemis* Dejean, 1835: 348**

Originally included available species: none.

***Imantocera* Dejean, 1835: 345**

Originally included available species: *Lamia plumosa* Olivier, 1792.

Type species: *Lamia plumosa* Olivier, 1792 by monotypy.

Current status: valid genus in Cerambycidae (*fide* Breuning 1962: 395, as “Imantocera Thoms[on] 1864”).

***Isarthron* Dejean, 1835: 329**

Originally included available species: *Callidium aulicum* Fabricius, 1775; *Cerambyx castaneus* Linnaeus, 1758 (as “*Castaneum. Paykull*.”); *Callidium femorale* Ménétriés, 1832; *Callidium fulcratum* Fabricius, 1792; *Callidium fuscum* Fabricius, 1787; *Cerambyx luridus* Linnaeus, 1767 (as “Luridum. *Fabr*.”).

Type species: *Callidium aulicum* Fabricius, 1775 (= *Cerambyx castaneus* Linnaeus, 1758) by subsequent designation (Linsley 1962: 85).

Current status: name suppressed in Cerambycidae.

Comments. The name *Isarthron* was suppressed for the purposes of the Principle of Priority in Opinion 1473 (ICZN 1988).

***Lagocheirus* Dejean, 1835: 336**

Originally included available species: *Cerambyx araneiformis* Linnaeus, 1767 (as “Areneiformis. *Fabr*.”).

Type species: *Cerambyx araneiformis* Linnaeus, 1767 by monotypy.

Current status: valid genus in Cerambycidae (*fide* Monné and Bezark 2009: 199).

***Lasiodactylus* Dejean, 1835: 335**

Originally included available species: none.

***Leprodera* Dejean, 1835: 342**

Originally included available species: none.

***Leprosoma* Dejean, 1835: 345**

Originally included available species: none.

***Leptocnemus* Dejean, 1835: 323**

Originally included available species: none.

***Leptoplia* Dejean, 1835: 339**

Comments. This name is treated as an unnecessary replacement name for *Microplia* Audinet-Serville, 1835 [Cerambycidae]. Audinet-Serville’s *Microplia* (1835: 21) was made available prior to Dejean’s publication (see “Precedence” section [7]).

***Leptoscelis* Dejean, 1835: 338**

Comments. This name is treated as a replacement name for *Anisopus* Audinet-Serville, 1835 [Cerambycidae], a junior homonym of *Anisopus* Meigen, 1803 [Diptera]. Audinet-Serville’s *Anisopus* (1835: 30) was made available prior to Dejean’s publication (see “Precedence” section [7]). *Leptoscelis* Dejean, 1835 is a junior homonym of *Leptoscelis* Laporte, 1832 [Hemiptera] and a senior synonym of *Anisopodus* White, 1855.

***Lypsymena* Dejean, 1835: 348**

Originally included available species: none.

***Macronemus* Dejean, 1835: 337**

Originally included available species: *Lamia antennator* Fabricius, 1801.

Type species: *Lamia antennator* Fabricius, 1801 by monotypy.

Current status: valid genus in Cerambycidae (*fide* Monné and Bezark 2009: 224).

***Maschalodonta* Dejean, 1835: 349**

Originally included available species: none.

***Mastigocera* Dejean, 1835: 345**

Originally included available species: *Lamia barbicornis* Fabricius, 1798.

Type species: *Lamia barbicornis* Fabricius, 1798 by monotypy.

Current status: senior objective synonym of *Mallonia* Thomson, 1857 in Cerambycidae (*fide* Gemminger and Harold 1873: 3057).

Comments. This name has precedence over *Mallonia* Thomson, 1857. Reversal of Precedence (ICZN 1999: Article 23.9) or an application to the Commission is necessary to conserve usage of the name *Mallonia* Thomson.

*Mastigocera* Dejean, 1835 is not a junior homonym of *Mastigocera* Berthold, 1827 [Hymenoptera] since Berthold’s name is unavailable (Taeger and Blank 1996: 255).

***Milothris* Dejean, 1835: 347**

Originally included available species: *Saperda irrorata* Fabricius, 1801; *Lamia lynx* Dalman, 1817; *Saperda marmorea* Schönherr, 1817.

Type species: *Saperda marmorea* Schönherr, 1817 by monotypy.

Current status: valid genus in Cerambycidae (*fide* Breuning 1961: 235, as “Milothris Cast[elnau] 1840”).

Comments. The names *irrorata* and *lynx* are listed in synonymy with *marmorea* in Dejean’s catalogue; therefore the type species of *Milothris* is *marmorea* by monotypy (ICZN 1999: Article 68.3).

***Monohammus* Dejean, 1835: 340** (as “Monohammus. *Megerle*.”)

Comments. This name is treated as an unjustified emendationof *Monochamus* Dejean, 1821. The name *Monohammus* was first proposed by Dahl (1823: 67) but his work was suppressed in Opinion 710 by the Commission (ICZN 1964) for nomenclatorial purposes.

***Myoxinus* Dejean, 1835: 336**

Originally included available species: none.

***Myzomorphus* Dejean, 1835: 319**

Originally included available species: none.

***Nosophloeus* Dejean, 1835: 320** (as “Nosophloeus. *Dupont*.”)

Comments. This name is treated as an unnecessary replacement name for *Cryptobias* Audinet-Serville, 1834 [Cerambycidae].

***Nyphona* Dejean, 1835: 344** (as “Nyphona. *Ziegler*.”)

Originally included available species: *Lamia obscurator* Fabricius, 1801.

Type species: *Lamia obscurator* Fabricius, 1801 by monotypy.

Current status: senior subjective synonym of *Hecyra* Thomson, 1857 in Cerambycidae (*fide* Marinoni 1977: 47).

Comments. *Nyphona* Dejean, 1835is a *nomen oblitum* and *Hecyra* Thomson, 1857 a *nomen protectum* following Sudre and Téocchi (2002).

***Oberea* Dejean, 1835: 350** (as “Oberea. *Megerle*.”)

Originally included available species: *Saperda atricornis* Fabricius, 1792; *Saperda depressa* Gebler, 1824; *Cerambyx erythrocephalus* Schrank, 1776 (as “Erythrocephala. *Fabr*.”); *Saperda gracilis* Fabricius, 1801; *Cerambyx linearis* Linnaeus, 1761 (as “Linearis. *Fabr*.”); *Saperda luteicollis* Gebler, 1833; *Cerambyx oculatus* Linnaeus, 1758 (as “Oculata. *Fabr*.”); *Saperda pupillata* Gyllenhal, 1817 (as “Pupillata. *Schönherr*. ”); *Saperda ruficollis* Fabricius, 1792; *Saperda tripunctata* Fabricius, 1792.

Type species: *Cerambyx linearis* Linnaeus, 1761 by subsequent designation (Thomson 1859: 153).

Current status: valid genus in Cerambycidae (*fide* Adlbauer et al. 2010: 296).

***Oedecnema* Dejean, 1835: 355**

Originally included available species: *Leptura dubia* Fabricius, 1781.

Type species: *Leptura dubia* Fabricius, 1781 (= *Oedecnema gebleri* Ganglbauer, 1889) by monotypy.

Current status: valid genus in Cerambycidae (*fide* Adlbauer et al. 2010: 107).

***Onchomerus* Dejean, 1835: 332**

Originally included available species: *Callidium pilicorne* Fabricius, 1792.

Type species: *Callidium pilicorne* Fabricius, 1792 (= *Callidium flavum* Fabricius, 1775) by monotypy.

Current status: senior synonym of *Curtomerus* Stephens, 1839 in Cerambycidae (*fide* Adlbauer et al. 2010: 157).

Comments. *Onchomerus* Dejean, 1835 is a *nomen oblitum* and *Curtomerus* Stephens, 1839 a *nomen protectum* following Sama (2010: 50).

***Onocephala* Dejean, 1835: 350**

Originally included available species: none.

***Ophistomis* Dejean, 1835: 355**

Originally included available species: none.

***Opsimus* Dejean, 1835: 329**

Originally included available species: none.

***Orion* Dejean, 1835: 327**

Originally included available species: none.

***Ornistomus* Dejean, 1835: 321**

Originally included available species: none.

***Pachystola* Dejean, 1835: 342**

Originally included available species: *Cerambyx textor* Linnaeus, 1758 (as “Textor. *Fabr*.”).

Type species: *Cerambyx textor* Linnaeus, 1758 by monotypy.

Current status: junior objective synonym of *Lamia* Fabricius, 1775 in Cerambycidae (*fide* Adlbauer et al. 2010: 267).

***Penthea* Dejean, 1835: 343**

Originally included available species: *Lamia vermicularisa* Donovan, 1805.

Type species: *Lamia vermicularisa* Donovan, 1805 by monotypy.

Current status: valid genus in Cerambycidae (*fide* McKeown 1947: 161, as “Penthea Castelneau, 1840”).

***Phacellocera* Dejean, 1835: 345**

Originally included available species: none.

***Phacellus* Dejean, 1835: 335**

Originally included available species: *Acanthocinus boryi* Gory, 1832.

Type species: *Acanthocinus boryi* Gory, 1832 by monotypy.

Current status: valid genus in Cerambycidae (*fide* Monné and Bezark 2009: 316).

***Phaula* Dejean, 1835: 348**

Originally included available species: none.

***Phidola* Dejean, 1835: 348**

Originally included available species: none.

***Phrissoma* Dejean, 1835: 345**

Originally included available species: *Lamia crispa* Fabricius, 1777; *Lamia rugosula* Guérin-Méneville, 1831.

Type species: *Lamia crispa* Fabricius, 1777 by subsequent designation (Desmarest 1860: 324).

Current status: valid genus in Cerambycidae (*fide* Aurivillius 1922: 64, as “Phrissoma Cast[elnau]”).

***Phryneta* Dejean, 1835: 341**

Originally included available species: *Lamia marmorea* Olivier, 1792; *Lamia spinator* Fabricius, 1792.

Type species: *Lamia marmorea* Olivier, 1792 by subsequent designation (Thomson 1864: 71).

Current status: valid genus in Cerambycidae (*fide* Adlbauer et al. 2010: 291).

***Phymasterna* Dejean, 1835: 342**

Originally included available species: *Lamia pictor* Klug, 1829; *Lamia sparsa* Klug, 1833.

Type species: none found.

Current status: unknown in Cerambycidae.

Comments. The name *Phymasterna* is usually credited to Laporte (1840: 473) (e.g., Aurivillius 1922: 176; Breuning 1959: 74) with *Phymasterna lacteoguttata* Laporte, 1840 as its type species by monotypy. *Lamia pictor* Klug, 1829 is currently included in the genus *Solymus* Lacordaire, 1872 (e.g., Breuning 1959: 78) and the second species, *Lamia sparsa* Klug, 1833, in the genus *Frea* Thomson, 1858 (e.g., Breuning 1962: 432). In order to preserve stability, we believe the best solution would be to apply to the Commission to suppress the name *Phymasterna* Dejean, 1835 for the Principles of Homonymy and Priority.

***Phymatoderus* Dejean, 1835: 340**

Originally included available species: none.

***Physobrachys* Dejean, 1835: 340**

Originally included available species: none.

***Phytoecia* Dejean, 1835: 350**

Originally included available species: *Saperda argus* Frölich, 1793 (as “Argus. *Fabr*.”); *Saperda azurea* Steven, 1817; *Cerambyx cylindricus* Linnaeus, 1758 (as “Cylindrica. *Fabr*.”); *Saperda ephippium* Fabricius, 1792; *Cerambyx ferreus* Schrank, 1776 (as “Ferrea. *Fabr*.”); *Saperda flavimana* Creutzer, 1796 (as “*Flavimana. Panzer*.”); *Saperda gilvimana* Ménétriés, 1832 (as “*Gilvimana. Stéven*.”); *Saperda hirsutula* Fabricius, 1801; *Saperda lineola* Fabricius, 1781; *Saperda nigricornis* Fabricius, 1781; *Saperda praetextata* Stéven, 1817; *Saperda punctum* Ménétriés, 1832 (as “Punctum. *Ziegler*.”); *Saperda rufimana* Schrank, 1789 (as “Rufimana. *Fabr*.”); *Saperda scutellata* Fabricius, 1792; *Saperda sibirica* Gebler, 1833; *Saperda virescens* Fabricius, 1781.

Type species: *Cerambyx cylindricus* Linnaeus, 1758 by subsequent designation (Thomson 1859: 153).

Current status: valid genus in Cerambycidae (*fide* Adlbauer et al. 2010: 302).

***Platyarthron* Dejean, 1835: 322**

Originally included available species: none.

***Platysternus* Dejean, 1835: 336**

Originally included available species: *Cerambyx hebraeus* Fabricius, 1781.

Type species: *Cerambyx hebraeus* Fabricius, 1781 by monotypy.

Current status: valid genus in Cerambycidae (*fide* Monné and Bezark 2009: 243).

***Plectrocerum* Dejean, 1835: 330**

Originally included available species: *Callidium spinicorne* Olivier, 1795.

Type species: *Callidium spinicorne* Olivier, 1795 by monotypy.

Current status: valid genus in Cerambycidae (*fide* Monné and Bezark 2009: 91).

***Plectrodera* Dejean, 1835: 341**

Originally included available species: *Lamia scalator* Fabricius, 1792.

Type species: *Lamia scalator* Fabricius, 1792 by monotypy.

Current status: valid genus in Cerambycidae (*fide* Monné and Bezark 2009: 296, as “Plectrodera Dejean 1837”).

***Plectromerus* Dejean, 1835: 332**

Originally included available species: none.

***Plectrura* Dejean, 1835: 346**

Originally included available species: none.

***Plocaederus* Dejean, 1835: 321**

Originally included available species: *Hamaticherus bellator* Audinet-Serville, 1834 (as “Bellator. *Dej*.”); *Cerambyx plicatus* Olivier, 1790.

Type species: *Hamaticherus bellator* Audinet-Serville, 1834 by subsequent designation (Gianfranco 1991: 123).

Current status: valid genus in Cerambycidae (*fide* Monné 2012: 11).

***Podius* Dejean, 1835: 333** (as “Podius. *Megerle*.”)

Comments. This name was listed by Dejean as an invalid synonym of *Deilus* Audinet-Serville, 1834. The name has not been treated before 1961 as an available name and adopted as the name of a taxon or treated as a senior homonym and therefore *Podius* Dejean is not available.

***Poeciloderma* Dejean, 1835: 330**

Originally included available species: none.

***Poecilopeplus* Dejean, 1835: 319**

Originally included available species: *Prionus corallifer* Sturm, 1826.

Type species: *Prionus corallifer* Sturm, 1826 by monotypy.

Current status: valid genus in Cerambycidae (*fide* Monné and Bezark 2009: 162).

***Polyzonus* Dejean, 1835: 324**

Originally included available species: *Cerambyx bicinctus* Olivier, 1795; *Saperda clavicornis* Fabricius, 1775; *Saperda fasciata* Fabricius, 1781; *Cerambyx sibiricus* Gmelin, 1789 (as “*Sibiricus. Pallas*.”).

Type species: *Saperda fasciata* Fabricius, 1781 by subsequent designation (Thomson 1864: 177).

Current status: valid genus in Cerambycidae (*fide* Adlbauer et al. 2010: 149).

***Praonetha* Dejean, 1835: 344**

Originally included available species: *Lamia alternans* Wiedemann, 1823; *Lamia crassipes* Wiedemann, 1823.

Type species: *Lamia crassipes* Wiedemann, 1823 by subsequent designation (Sama 2002: 95).

Current status: senior subjective synonym of *Pterolophia* Newman, 1842 in Cerambycidae (*fide* Adlbauer et al. 2010: 319).

Comments. Sama (2002: 95) listed *Praonetha* Dejean as *nomen oblitum* and *Pterolophia* Newman as *nomen protectum* although he did not provide the necessary references to justify his action (see ICZN 1999: Article 23.9.2).

***Probatius* Dejean, 1835: 337**

Originally included available species: none.

***Prosopocera* Dejean, 1835: 343**

Originally included available species: none.

***Psectrocera* Dejean, 1835: 345**

Originally included available species: none.

***Pteroplatus* Dejean, 1835: 321**

Originally included available species: none.

***Pterostenus* Dejean, 1835: 353** (as “Pterostenus. *Mac Leay*.”)

Originally included available species: *Cerambyx abbreviatus* Fabricius, 1801; *Leptura ceramboides* Kirby, 1818.

Type species: *Cerambyx abbreviatus* Fabricius, 1801 (= *Stenocorus suturalis* Olivier, 1795) by **present designation**.

Current status: junior objective synonym of *Stenoderus* Dejean, 1821 in Cerambycidae (*fide* Dejean 1835: 353, as “Pterostenus *Mac Leay*”).

Comment. *Pterostenus* was first proposed by Dejean as an invalid synonym of *Stenoderus* Dejean, 1821. It is available because it was treated before 1961 as an available name and adopted as the name of a taxon (e.g., Lacordaire 1868: 412).

McKeown (1947: 72) used *Stenocentrus* McKeown, 1845 as the valid name for the genus including *Stenocorus suturalis* Olivier, 1795. However, the valid name should be *Stenoderus* Dejean, 1821.

***Pyrobolus* Dejean, 1835: 352** (as “Pyrobolus.”)

Comments. This name was listed by Dejean as an invalid synonym of *Amphionycha* Dejean, 1835. The name has not been treated before 1961 as an available name and adopted as the name of a taxon or treated as a senior homonym and therefore *Pyrobolus* Dejean is not available.

***Schoeniocera* Dejean, 1835: 345**

Originally included available species: none.

***Sclerocerus* Dejean, 1835: 328**

Originally included available species: none.

***Scleronotus* Dejean, 1835: 336**

Originally included available species: none.

***Sericogaster* Dejean, 1835: 324**

Originally included available species: none.

***Smodicum* Dejean, 1835: 332**

Originally included available species: none.

***Sophronica* Dejean, 1835: 347**

Originally included available species: none.

***Sphenothecus* Dejean, 1835: 321**

Originally included available species: none.

***Sphenura* Dejean, 1835: 350**

Originally included available species: *Saperda bidentata* Fabricius, 1792; *Saperda fricator* Dalman, 1817; *Saperda morbillosa* Fabricius, 1798; *Saperda novemguttata* Guérin-Méneville, 1831 (as “Novemguttata. *Dej*.”); *Saperda venusta* Guérin-Méneville, 1831; *Saperda viridicincta* Boisduval, 1835.

Type species: *Saperda fricator* Dalman, 1817 by subsequent designation (Desmarest 1860: 326).

Current status: junior homonym of *Sphenura* Lichtenstein, 1820 [Aves]; senior objective synonym of *Nupserha* Chevrolat, 1858 in Cerambycidae (Chevrolat 1858: 358).

Comments. *Nupserha* Chevrolat (1858: 358) was proposed as a replacement name for *Sphenura* Dejean.

***Stellognatha* Dejean, 1835: 342**

Originally included available species: *Cerambyx maculatus* Olivier, 1795.

Type species: *Cerambyx maculatus* Olivier, 1795 by monotypy.

Current status: valid genus in Cerambycidae (*fide* Aurivillius 1922: 164, as “Stellognatha Cast[elnau]”).

***Stenopeplus* Dejean, 1835: 327**

Originally included available species: none.

***Stenosphenus* Dejean, 1835: 330**

Originally included available species: none.

***Stenostola* Dejean, 1835: 350**

Originally included available species: *Saperda dubia* Laicharting, 1784 (as “*Dubia. Megerle*.”); *Cerambyx ferreus* Schrank, 1776 (as “*Ferrea. Sturm*.”); *Saperda nigripes* Fabricius, 1792.

Type species: *Saperda nigripes* Fabricius, 1792 (= *Cerambyx ferreus* Schrank, 1776) by monotypy.

Current status: valid genus in Cerambycidae (*fide* Sama 2002: 112).

Comments. The names *dubia* and *ferrea* are listed in synonymy with *nigripes* in Dejean’s catalogue; therefore the type species of *Stenostola* is *nigripes* by monotypy (ICZN 1999: Article 68.3).

***Stenura* Dejean, 1835: 355**

Originally included available species: *Leptura atra* Fabricius, 1775; *Leptura aurulenta* Fabricius, 1792; *Leptura bifasciata* Schrank, 1781; *Leptura cruciata* Olivier, 1795; *Leptura distigma* Charpentier, 1825 (as “Distigma. *Hoffmansegg*.”); *Leptura duodecimguttata* Fabricius, 1801; *Leptura emarginata* Fabricius, 1787; *Leptura fugax* Fabricius, 1798; *Leptura holosericea* Fabricius, 1801 [no 22]; *Leptura jaegeri* Hummel, 1824; *Leptura lunata* Fabricius, 1801; *Leptura melanura* Linnaeus, 1758 (as “Melanura. *Fabr*. ”); *Leptura nigra* Linnaeus, 1758 (as “Nigra. *Fabr*. ”); *Leptura nigripes* DeGeer, 1775 (as “Nigripes. *Paykull*. ”); *Leptura pubescens* Fabricius, 1787; *Leptura quadrifasciata* Linnaeus, 1758 (as “Quadrifasciata. *Fabr*.”); *Leptura septempunctata* Fabricius, 1792; *Leptura thoracica* Creutzer, 1799 (as “Thoracica. *Fabr*.”); *Leptura velutina* Olivier, 1795; *Leptura villica* Fabricius, 1775; *Leptura zebra* Olivier, 1795; *Leptura zebrata* Fabricius, 1801.

Type species: *Leptura emarginata* Fabricius, 1787 by subsequent designation (Chemsak 1964: 235).

Current status: junior homonym of *Stenura* Cuvier, 1829 [Aves]; senior objective synonym of *Stenelytrana* Gistel, 1848 in Cerambycidae (*fide* Monné and Bezark 2009: 182).

Comments. *Stenelytrana* Gistel, 1848 was proposed as a replacement name for *Stenura* Dejean.

***Sternodonta* Dejean, 1835: 342**

Originally included available species: *Lamia imperialis* Fabricius, 1801; *Cerambyx ornatus* Olivier, 1795; *Lamia regalis* Fabricius, 1781.

Type species: *Cerambyx ornatus* Olivier, 1795 (= *Cerambyx pulcher* Drury, 1773) by subsequent designation (Marinoni 1977: 49).

Current status: senior synonym of *Sternotomis* Percheron, 1836 in Cerambycidae (*fide* Aurivillius 1922: 166, as “*Sternodonta* Cast[elnau]”).

Comments. *Sternodonta* Dejean, 1835 is a *nomen oblitum* and *Sternotomis* Percheron, 1836 a *nomen protectum* following Sama (2009: 24).

***Sthenias* Dejean, 1835: 344** (as “Sthenias. *Dupont*.”)

Originally included available species: *Lamia cylindrator* Fabricius, 1801; *Lamia grisator* Fabricius, 1787.

Type species: *Lamia grisator* Fabricius, 1787 by monotypy.

Current status: valid genus in Cerambycidae (*fide* Breuning 1961: 233, as “Sthenias Cast[elnau] 1840”).

Comments. The name *cylindrator* is listed as a variety of *grisator* in Dejean’s catalogue; therefore the type species of *Sthenias* is *grisator* by monotypy (ICZN 1999: Article 68.3).

***Strangalia* Dejean, 1835: 355** (as “Strangalia. *Serville*.”)

Originally included available species: *Leptura annularis* Fabricius, 1801; *Leptura arcuata* Panzer, 1793; *Leptura attenuata* Linnaeus, 1758 (as “Attenuata. *Fabr*.”); *Leptura bicolor* Swederus, 1787; *Leptura calcarata* Fabricius, 1792; *Leptura luteicornis* Fabricius, 1775; *Leptura subspinosa* Fabricius, 1792.

Type species: *Leptura luteicornis* Fabricius, 1775 by subsequent designation (Thomson 1864: 141).

Current status: valid genus in Cerambycidae (*fide* Adlbauer et al. 2010: 116, as “*Strangalia* Audinet-Serville, 1835”).

Comments. The name *Strangalia* was proposed the same year by Dejean (1835: 355) and Audinet-Serville (1835: 220). As indicated in the “Precedence” section [8], Dejean’s name has priority.

***Talaepora* Dejean, 1835: 347**

Originally included available species: none.

***Tetraophthalmus* Dejean, 1835: 347** (as “Tetraophthalmus. *De Haan*.”)

Originally included available species: *Lamia daldorfii* Illiger, 1800; *Cerambyx splendidus* Fabricius, 1792.

Type species: *Cerambyx splendidus* Fabricius, 1792 by monotypy.

Current status: valid genus in Cerambycidae (*fide* Adlbauer et al. 2010: 237).

Comments. The name *daldorfii* is listed as a variety of *splendidus* in Dejean’s catalogue; therefore the type species of *Tetraophthalmus* is *splendidus* by monotypy (ICZN 1999: Article 68.3).

***Trachystola* Dejean, 1835: 343**

Originally included available species: none.

***Tragocephala* Dejean, 1835: 342** (as “Tragocephala. *Dupont*.”)

Originally included available species: *Lamia formosa* Olivier, 1792; *Lamia nobilis* Fabricius, 1787; *Cerambyx virescens* Olivier, 1795.

Type species: *Lamia formosa* Olivier, 1792 by subsequent designation (Thomson 1864: 70).

Current status: valid genus in Cerambycidae (*fide* Breuning 1959: 81).

***Tragomorphus* Dejean, 1835: 335**

Comments. This name is treated as an unnecessary replacement name for *Anisocerus* Lacordaire, 1830 [Cerambycidae].

***Trichoscelis* Dejean, 1835: 330**

Originally included available species: none.

***Trigonopeplus* Dejean, 1835: 335**

Originally included available species: none.

***Trigonotarsis* Dejean, 1835: 356**

Originally included available species: none.

***Trypanidius* Dejean, 1835: 337**

Originally included available species: none.

***Xylorhiza* Dejean, 1835: 344**

Originally included available species: *Lamia adusta* Wiedemann, 1819.

Type species: *Lamia adusta* Wiedemann, 1819 by monotypy.

Current status: valid genus in Cerambycidae (*fide* Adlbauer et al. 2010: 334).

***Zographus* Dejean, 1835: 342**

Originally included available species: *Lamia oculator* Fabricius, 1775.

Type species: *Lamia oculator* Fabricius, 1775 by monotypy.

Current status: valid genus in Cerambycidae (*fide* Breuning 1959: 88, as “Zographus Cast[elnau] 1840”).

***Zygocera* Dejean, 1835: 344**

Originally included available species: *Acanthocinus pruinosus* Boisduval, 1835 (as “Pruinosa. *Mac Leay*.”).

Type species: *Acanthocinus pruinosus* Boisduval, 1835 by monotypy.

Current status: senior objective synonym of *Disternopsis* Breuning, 1939 in Cerambycidae (**new synonymy**).

Comments. *Zygocera* is currently credited to Erichson (1842: 224) in a sense different than that of Dejean (1835) (e.g., Breuning 1970: 85) and *Disternopsis* Breuning, 1939 is the current valid generic name for the type species of *Zygocera* Dejean, 1835. In order to promote stability, we believe the best avenue would be to apply to the Commission to suppress *Zygocera* Dejean, 1835 for both the Principles of Homonymy and Priority.

### Tétramères: Chrysomélines

***Acentroptera* Chevrolat, 1836: 364**

Originally included available species: none.

***Acidalia* Chevrolat, 1836: 417**

Originally included available species: *Clythra varians* Sahlberg, 1823 (as “Varians. *Sturm*.”).

Type species: *Clythra varians* Sahlberg, 1823 by monotypy.

Current status: junior homonym of *Acidalia* Hübner, 1819 [Lepidoptera]; senior objective synonym of *Helioscopa* Gistel, 1837 in Chrysomelidae (*fide* Monrós and Bechyné 1956: 1122).

***Acis* Chevrolat, 1836: 411**

Comments. This name is treated as an unnecessary replacement name for *Colasposoma* Laporte, 1833 [Chrysomelidae]. *Acis* Chevrolat, 1836 is a junior homonym of *Acis* Billberg, 1820 [Tenebrionidae].

***Acromis* Chevrolat, 1836: 370**

Originally included available species: *Cassida perforata* Pallas, 1772 (as “Var. *Perforata. Fabr*.”); *Cassida spinifex* Linnaeus, 1763 (as “Spinifex. *Fabr*.”).

Type species: *Cassida spinifex* Linnaeus, 1763 by monotypy.

Current status: valid genus in Chrysomelidae (*fide* Borowiec 1999: 83).

Comments. The name *perforata* is listed as a variety of *spinifex* in Dejean’s catalogue; therefore the type species of *Acromis* is *spinifex* by monotypy (ICZN 1999: Article 68.3).

***Agelastica* Chevrolat, 1836: 381**

Originally included available species: *Chrysomela alni* Linnaeus, 1758 (as “Alni. *Fabr*.”).

Type species: *Chrysomela alni* Linnaeus, 1758 by monotypy.

Current status: valid genus in Chrysomelidae (*fide* Beenen 2010: 455).

***Amasia* Dejean, 1836a: 411**

Originally included available species: none.

***Amblyopus* Chevrolat, 1836: 429**

Originally included available species: *Triplax vittata* Olivier, 1807.

Type species: *Triplax vittata* Olivier, 1807 by monotypy.

Current status: valid genus in Erotylidae (*fide* Wegrzynowicz 2007b: 541).

***Amphicyrta* Dejean, 1836a: 405** (as “Amphicyrta. *Eschscholtz*.”)

Originally included available species: none.

***Amphilocus* Dejean, 1836a: 426**

Originally included available species: none.

***Anisodera* Chevrolat, 1836: 363**

Originally included available species: *Alurnus ferrugineus* Fabricius, 1801.

Type species: *Alurnus ferrugineus* Fabricius, 1801 by monotypy.

Current status: valid genus in Chrysomelidae (*fide* Borowiec and Sekerka 2010: 368).

***Anomoia* Chevrolat, 1836: 419**

Originally included available species: *Clytra ephippium* Germar, 1824; *Cryptocephalus obsitus* Fabricius, 1775.

Type species: *Cryptocephalus obsitus* Fabricius, 1775 by monotypy.

Current status: junior homonym of *Anomoia* Walker, 1835 [Diptera]; senior objective synonym of *Anomoea* Agassiz, 1846 in Chrysomelidae (*fide* Riley et al. 2003: 175).

Comments. The name *ephippium* is listed in synonymy with *obsitus* in Dejean’s catalogue; therefore the type species of *Anomoia* is *obsitus* by monotypy (ICZN 1999: Article 68.3).

***Aphthona* Chevrolat, 1836: 391**

Originally included available species: *Galeruca coerulea* Paykull, 1799; *Haltica cyparissiae* Koch, 1803 (as “Cyparissiae. *Ent. Hefte*.”); *Chrysomela euphorbiae* Schrank (as “Euphorbiae. *Fabr*.”); *Haltica lutescens* Gyllenhal, 1813; *Galeruca rubi* Paykull, 1799 (as “Rubi. *Fabr*.”); *Galeruca salicariae* Paykull, 1800.

Type species: *Haltica cyparissiae* Knoch, 1803 by subsequent designation (Maulik 1926: 366).

Current status: valid genus in Chrysomelidae (*fide* Döberl 2010: 496).

***Aplosonyx* Chevrolat, 1836: 375**

Originally included available species: *Galleruca albicornis* Wiedemann, 1821; *Galleruca javana* Wiedemann, 1819; *Galleruca semiflava* Wiedemann, 1819.

Type species: *Galleruca albicornis* Wiedemann, 1821 by subsequent designation (Duponchel and Chevrolat 1841: 17).

Current status: valid genus in Chrysomelidae (*fide* Beenen 2010: 456).

***Apophylia* Chevrolat, 1836: 382**

Originally included available species: none.

***Apteropeda* Chevrolat, 1836: 393**

Originally included available species: *Altica ciliata* Olivier, 1808; *Haltica hederae* Illiger, 1807.

Type species: *Altica ciliata* Olivier, 1808 (= *Chrysomela orbiculata* Marsham, 1802) by monotypy.

Current status: valid genus in Chrysomelidae (*fide* Döberl 2010: 503).

Comments. The name *hederae* is listed in synonymy with *ciliata* in Dejean’s catalogue; therefore the type species of *Apteropeda* is *ciliata* by monotypy (ICZN 1999: Article 68.3).

***Asphaera* Chevrolat, 1836: 387**

Originally included available species: none.

***Aspicela* Dejean, 1836a: 387**

Originally included available species: *Altica albomarginata* Latreille, 1809; *Altica cretacea* Latreille, 1813; *Altica scutata* Latreille, 1813; *Altica unipunctata* Latreille, 1813.

Type species: *Altica unipunctata* Latreille, 1813 by subsequent designation (Monrós and Bechyné 1956: 1134).

Current status: valid genus in Chrysomelidae (*fide* Seeno and Wilcox 1982: 140).

***Asteriza* Chevrolat, 1836: 372**

Originally included available species: *Cassida flavicornis* Olivier, 1790.

Type species: *Cassida flavicornis* Olivier, 1790 by monotypy.

Current status: valid genus in Chrysomelidae (*fide* Borowiec 1999: 169).

***Astolisma* Dejean, 1836a: 387**

Originally included available species: none.

***Atechna* Chevrolat, 1836: 403**

Originally included available species: *Chrysomela alternans* Fabricius, 1794; *Chrysomela guttata* Fabricius, 1792; *Chrysomela linea* Fabricius, 1796; *Chrysomela quatuordecimguttata* Fabricius, 1798; *Chrysomela striata* Fabricius, 1781; *Chrysomela trilineata* Boisduval, 1835 (as “Trilineata. *d’Urville*.”); *Chrysomela vigintiguttata* Olivier, 1807; *Chrysomela vulpina* Fabricius, 1781.

Type species: *Chrysomela quatuordecimguttata* Fabricius, 1798 by subsequent designation (Chevrolat 1843: 656).

Current status: valid subgenus of *Chrysolina* Motschulsky, 1860 in Chrysomelidae (*fide* Seeno and Wilcox 1982: 81).

Comments. In Opinion 1279, the ICZN (1984) ruled that “*Atechna* Chevrolat, 1837” is not to be given priority over *Chrysolina* Motschulsky, 1860 when the two names are regarded as synonyms.

***Atrachya* Dejean, 1836a: 377**

Originally included available species: *Galleruca menetriesii* Faldermann, 1835.

Type species: *Galleruca menetriesii* Faldermann, 1835 by monotypy.

Current status: valid genus in Chrysomelidae (*fide* Beenen 2010: 469, as “*Atrachya* Chevrolat, 1836”).

***Aulacocheilus* Chevrolat, 1836: 429**

Originally included available species: *Erotylus quadripustulatus* Fabricius, 1801.

Type species: *Erotylus quadripustulatus* Fabricius, 1801 by monotypy.

Current status: valid genus in Erotylidae (*fide* Delkeskamp 1981: 56).

***Aulacophora* Chevrolat, 1836: 378**

Originally included available species: *Galleruca analis* Weber, 1801 (as “Analis. *Fabr*.”); *Galeruca bipunctata* Olivier, 1808; *Galleruca cyanoptera* Boisduval, 1835 (as “Cyanoptera. *d’Urville*.”); *Galleruca dorsalis* Boisduval, 1835; *Galeruca hilaris* Boisduval, 1835 (as “*Hilaris. Mac Leay*.”); *Galeruca quadraria* Olivier, 1808; *Galleruca rosea* Fabricius, 1801; *Galleruca vicina* Boisduval, 1835 (as “Vicina. *d’Urville*.”).

Type species: *Galleruca quadraria* Olivier, 1808 by subsequent designation (Duponchel and Chevrolat 1842a: 337).

Current status: valid genus in Chrysomelidae (*fide* Beenen 2010: 465).

***Aulacoscelis* Chevrolat, 1836: 395**

Originally included available species: none.

***Australica* Chevrolat, 1836: 402**

Originally included available species: *Chrysomela curtisii* Kirby, 1819 (as “*curtissii. Kirby*.”); *Chrysomela maculicollis* Boisduval, 1835 (as “Maculicollis. *d’Urville*.”); *Chrysomela macleayi* Boisduval, 1835 (as “Mac Leayi. *Dej*.”); *Chrysomela ruficeps* Boisduval, 1835 (as “Ruficeps. *Mac Leay*.”).

Type species: *Chrysomela curtisii* Kirby, 1819 by subsequent designation (Chevrolat 1843: 656).

Current status: senior objective synonym of *Calomela* Hope, 1840 in Chrysomelidae (*fide* Seeno and Wilcox 1982: 86).

Comments. *Australica* Chevrolat, 1836 has precedence over *Calomela* Hope, 1840 which is currently used as valid (e.g., Reid 2006: 53). Reversal of Precedence (ICZN 1999, Article 23.9) or an application to the Commission is necessary to conserve usage of the name *Calomela* Hope, 1840.

***Axiotheata* Chevrolat, 1836: 387**

Originally included available species: none.

***Babia* Chevrolat, 1836: 417**

Originally included available species: *Chlamys cruciata* Klug, 1824; *Clythra pusilla* Klug, 1829 (as “Pusilla. *Dej*.”); *Clytra quadriguttata* Olivier, 1791.

Type species: *Clytra quadriguttata* Olivier, 1791 by subsequent designation (Monrós 1953: 46).

Current status: valid genus in Chrysomelidae (*fide* Riley et al. 2003: 180).

***Bacis* Dejean, 1836a: 427**

Originally included available species: *Erotylus tripunctatus* Duponchel, 1825 (as “Tripunctatus. *Dej*.”).

Type species: *Erotylus tripunctatus* Duponchel, 1825 by monotypy.

Current status: valid genus in Erotylidae (*fide* Blackwelder 1945: 463, as “*Bacis* Hope [18]41”).

***Balanomorpha* Chevrolat, 1836: 393**

Originally included available species: *Haltica chrysanthemi* Koch, 1803 (as “Chrysanthemi. *Ent. Hefte*.”); *Haltica obtusata* Gyllenhal, 1813; *Chrysomela rustica* Linnaeus, 1766 (as “*Rustica. Illiger*.”); *Galleruca semiaenea* Fabricius, 1792.

Type species: *Haltica chrysanthemi* Koch, 1803 by subsequent designation (Monrós and Bechyné 1956: 1133).

Current status: junior objective synonym of *Mantura* Stephens, 1831 in Chrysomelidae (*fide* Döberl 2010: 536).

Comments. The type species mentioned by Riley et al. (2003: 114) and Döberl (2010: 536), *Galleruca semiaenea* Fabricius, 1792 (= *Chrysomela rustica* Linnaeus, 1767), is incorrect (Manfred Döberl, pers. comm.).

***Barytopus* Chevrolat, 1836: 425**

Originally included available species: *Erotylus adustus* Duponchel, 1825 (as “Adustus. *Dej*.”); *Erotylus alternans* Olivier, 1791 (as “Alternans. *Fabr*.”); *Erotylus decemmaculatus* Duponchel, 1825 (as “Decemmaculatus. *Dej*.”); *Erotylus distinctus* Duponchel, 1825 (as “Distinctus. *Dej*.”); *Erotylus fasciatus* Olivier, 1791 (as “Fasciatus. *Fabr*.”); *Erotylus flavofasciatus* Duponchel, 1825 (as “Flavofasciatus. *Dej*.”); *Erotylus militaris* Germar, 1824; *Erotylus notatus* Olivier, 1791 (as “Notatus. *Fabr*.”); *Erotylus ramosus* Olivier, 1807; *Erotylus tricinctus* Duponchel, 1825 (as “Tricinctus. *Dej*.”); *Erotylus trifasciatus* Olivier, 1807; *Erotylus zebra* Fabricius, 1787.

Type species: *Erotylus alternans* Olivier, 1791 (= *Chrysomela gronovii* Herbst, 1783) by subsequent designation (Duponchel and Chevrolat 1842a: 483).

Current status: valid genus in Erotylidae (*fide* Alvarenga 1965: 82).

Comments. As mentioned by Alvarenga (1965: 82), *Barytopus* Chevrolat, 1836 is a senior synonym of *Micrerotylus* Crotch, 1876.

***Basiprionota* Chevrolat, 1836: 367**

Originally included available species: *Cassida octopunctata* Fabricius, 1787.

Type species: *Cassida octopunctata* Fabricius, 1787 by monotypy.

Current status: valid genus in Chrysomelidae (*fide* Borowiec and Sekerka 2010: 369).

***Basipta* Chevrolat, 1836: 374**

Originally included available species: none.

***Bathis* Dejean, 1836a: 409**

Originally included available species: none.

***Bathseba* Dejean, 1836a: 411**

Originally included available species: none.

***Blepharida* Chevrolat, 1836: 394**

Originally included available species: *Haltica marmorea* Wiedemann, 1819; *Chrysomela meticulosa* Olivier, 1808.

Type species: *Chrysomela meticulosa* Olivier, 1808 (= *Blepharida rhois* Forster, 1771) by subsequent designation (Chevrolat 1842: 606).

Current status: valid genus in Chrysomelidae (*fide* Döberl 2010: 505).

***Botanochara* Dejean, 1836a: 369**

Originally included available species: *Cassida angulata* Germar, 1824; *Cassida nervosa* Fabricius, 1801; *Cassida octopustulata* Klug, 1829 (as “Octopustulata. *Dej*.”).

Type species: *Cassida nervosa* Fabricius, 1801 (= *Cassida impressa* Panzer, 1798) by subsequent designation (Hincks 1952: 335).

Current status: valid genus in Chrysomelidae (*fide* Borowiec 1999: 87).

***Botryonopa* Chevrolat, 1836: 363**

Originally included available species: none

***Brachycoryna* Dejean, 1836a: 366**

Originally included available species: none.

***Brachymerus* Chevrolat, 1836: 427**

Originally included available species: *Erotylus ephippium* Duponchel, 1825 (as “Ephippium. *Dej*.”); *Erotylus flavosignatus* Duponchel, 1825 (as “Flavosignatus. *Dej*.”); *Erotylus fuscipes* Duponchel, 1825 (as “Fuscipes. *Dej*.”); *Erotylus fuscomaculatus* Duponchel, 1825 (as “Fuscomaculatus. *Dej*.”); *Erotylus lineellus* Duponchel, 1825 (as “Lineellus. *Dej*.”); *Erotylus nitidulus* Olivier, 1807; *Erotylus oculatus* Duponchel, 1825 (as “Oculatus. *Dej*.”); *Erotylus quadrimaculatus* Duponchel, 1825 (as “Quadrimaculatus. *Dej*.”); *Erotylus signatus* Duponchel, 1825 (as “Signatus. *Dej*.”); *Erotylus tibialis* Duponchel, 1825 (as “Tibialis. *Dej*.”).

Type species: *Erotylus tibialis* Duponchel, 1825 by subsequent designation (Hope 1841: 113).

Current status: valid subgenus of *Iphiclus* Chevrolat, 1836 in Erotylidae (*fide* Alvarenga 1965: 82).

***Bromius* Chevrolat, 1836: 412**

Originally included available species: *Eumolpus hirtus* Fabricius, 1801; *Chrysomela obscura* Linnaeus, 1758 (as “Obscurus. *Fabr*.”); *Cryptocephalus vitis* Fabricius, 1775.

Type species: *Chrysomela obscura* Linnaeus, 1758 by subsequent designation (Monrós and Bechyné 1956: 1127).

Current status: valid genus in Chrysomelidae (*fide* Moseyko and Sprecher-Uebersax 2010: 621).

Comments. This name was recently placed on the Official List of Generic Names in Zoology in Opinion 2298 (ICZN 2012: 147).

***Cacoscelis*Chevrolat, 1836: 389**

Originally included available species: *Altica binotata* Illiger, 1807; *Chrysomela famelica* Fabricius, 1787; *Chrysomela fervida* Fabricius, 1775 (as “*Fervida. Olivier*.”); *Galleruca melanoptera* Germar, 1821; *Altica quinquelineata* Latreille, 1809.

Type species: *Chrysomela famelica* Fabricius, 1787 by subsequent designation (Monrós and Bechyné 1956: 1133).

Current status: valid genus in Chrysomelidae (*fide* Seeno and Wilcox 1982: 133).

***Cadmus* Chevrolat, 1836: 420**

Originally included available species: *Cryptocephalus rubiginosus* Boisduval, 1835 (as “Rubiginosus. *Mac Leay*.”).

Type species: *Cryptocephalus rubiginosus* Boisduval, 1835 (= *Cryptocephalus gigas* Olivier, 1807) by monotypy.

Current status: valid genus in Chrysomelidae (*fide* Seeno and Wilcox 1982: 39, as “*Cadmus* Erichson 1842”).

***Caeporis* Dejean, 1836a: 387**

Originally included available species: *Galeruca stigmula* Germar, 1824.

Type species: *Galeruca stigmula* Germar, 1824 by monotypy.

Current status: valid genus in Chrysomelidae (*fide* Seeno and Wilcox 1982: 134).

***Calenus* Dejean, 1836a: 427**

Originally included available species: *Erotylus signaticollis* Duponchel, 1825.

Type species: *Erotylus signaticollis* Duponchel, 1825 by monotypy.

Current status: valid genus in Erotylidae (*fide* Alvarenga 1965: 82).

Comments. As mentioned by Alvarenga (1965: 82), *Calenus* Dejean, 1836 is a senior synonym of *Glabrototelus* Mader, 1942.

***Calliaspis* Dejean, 1836a: 367**

Originally included available species: *Cassida rubra* Olivier, 1808.

Type species: *Cassida rubra* Olivier, 1808 by monotypy.

Current status: valid genus in Chrysomelidae (*fide* Seeno and Wilcox 1982: 172).

***Calligrapha* Chevrolat, 1836: 398**

Originally included available species: *Chrysomela decipiens* Weber, 1801; *Chrysomela exclamationis* Fabricius, 1798; *Chrysomela philadelphica* Linnaeus, 1758 (as “Philadelphica. *Fabr*.”); *Chrysomela polyspila* Germar, 1821; *Chrysomela punctipennis* Germar, 1824; *Chrysomela vigintimaculata* Chevrolat, 1833.

Type species: *Chrysomela polyspila* Germar, 1821 by subsequent designation (Chevrolat 1843: 656).

Current status: valid genus in Chrysomelidae (*fide* Riley et al. 2003: 51).

***Callipepla* Dejean, 1836a: 375**

Originally included available species: *Adorium posticum* Boisduval, 1835 (as “Postica. *d’Urville*.”); *Galleruca sexsignata* Boisduval, 1835 (as “Sexsignata. *d’Urville*.”).

Type species: *Adorium posticum* Boisduval, 1835 by subsequent designation (Wilcox 1971: 2).

Current status: junior homonym of *Callipepla* Wagler, 1832 [Aves]; junior subjective synonym of *Oides* Weber, 1801 in Chrysomelidae (*fide* Beenen 2010: 490).

***Callistola* Dejean, 1836a: 363**

Originally included available species: *Hispa speciosa* Boisduval, 1835 (as “Speciosa. *d’Urville*.”).

Type species: *Hispa speciosa* Boisduval, 1835 by monotypy.

Current status: valid genus in Chrysomelidae (*fide* Seeno and Wilcox 1982: 163).

***Callopistria* Chevrolat, 1836: 378**

Originally included available species: *Galleruca fulminans* Faldermann, 1835.

Type species: *Galleruca fulminans* Faldermann, 1835 by monotypy.

Current status: junior homonym of *Callopistria* Hübner, 1821 [Lepidoptera]; senior objective synonym of *Clitenella* Laboissière, 1927 in Chrysomelidae (*fide* Beenen 2010: 445).

***Calyptocephala* Chevrolat, 1836: 367**

Originally included available species: *Cassida nigricornis* Germar, 1824 (as “Nigricornis. *Dej. Germar*.”).

Type species: *Cassida nigricornis* Germar, 1824 by monotypy.

Current status: valid genus in Chrysomelidae (*fide* Borowiec 1999: 34).

***Camptolenes* Chevrolat, 1836: 419**

Originally included available species: *Clythra psilothorax* Wiedemann, 1823 (as “Spilothorax. *Wiedemann*.”); *Clythra rugosa* Fabricius, 1798.

Type species: *Clythra rugosa* Fabricius, 1798 by subsequent designation (Monrós 1953: 47).

Current status: junior synonym of *Clytra* Laicharting, 1781 in Chrysomelidae (*fide* Seeno and Wilcox 1982: 32).

***Centroscelis* Chevrolat, 1836: 403**

Originally included available species: *Chrysomela notata* Fabricius, 1781.

Type species: *Chrysomela notata* Fabricius, 1781 by monotypy.

Current status: valid genus in Chrysomelidae (*fide* Seeno and Wilcox 1982: 85).

***Cephalodonta* Chevrolat, 1836: 364**

Originally included available species: none.

***Cephaloleia* Chevrolat, 1836: 366**

Originally included available species: *Hispa metallica* Fabricius, 1801; *Hispa nigricornis* Fabricius, 1792.

Type species: *Hispa nigricornis* Fabricius, 1792 by subsequent designation (Staines 1991: 247).

Current status: valid genus in Chrysomelidae (*fide* Seeno and Wilcox 1982: 159).

***Cerophysa* Chevrolat, 1836: 379**

Originally included available species: *Galleruca nodicornis* Wiedemann, 1823.

Type species: *Galleruca nodicornis* Wiedemann, 1823 by monotypy.

Current status: valid genus in Chrysomelidae (*fide* Beenen 2010: 472).

***Cerotoma* Chevrolat, 1836: 379**

Originally included available species: *Crioceris arcuata* Olivier, 1791; *Crioceris caminea* Fabricius, 1801; *Crioceris cincta* Fabricius, 1775; *Galleruca denticornis* Fabricius, 1792; *Galeruca furcata* Olivier, 1808; *Crioceris laeta* Fabricius, 1801; *Galeruca melanura* Olivier, 1808; *Chrysomela palliata* Schaller, 1783 (as “Palliata. *Fabr*.”); *Altica quinquefasciata* Latreille, 1813; *Crioceris variegata* Fabricius, 1792.

Type species: *Crioceris caminea* Fabricius, 1801 (= *Crioceris ruficornis* Olivier, 1791) by subsequent designation (Chapuis 1875: 230).

Current status: valid genus in Chrysomelidae (*fide* Riley et al. 2003: 80).

***Chalcophana* Chevrolat, 1836: 407**

Originally included available species: *Colaspis aurata* Olivier, 1808; *Colaspis glabrata* Fabricius, 1801 (as “*Glabrata. Schüppel. Fabr?*”); *Colaspis hilaris* Germar, 1824; *Colaspis picipes* Olivier, 1808; *Colaspis proxima* Klug, 1829 (as “Proxima. *Dej*.”); *Colaspis ruficrus* Germar, 1824.

Type species: *Colaspis hilaris* Germar, 1824 by subsequent designation (Monrós and Bechyné 1956: 1125).

Current status: valid genus in Chrysomelidae (*fide* Seeno and Wilcox 1982: 57, as “*Chalcophana* Chevrolat, 1843”).

***Chalcoplacis* Chevrolat, 1836: 409**

Originally included available species: *Colaspis fulgurans* Klug, 1829.

Type species: *Colaspis fulgurans* Klug, 1829 by monotypy.

Current status: valid genus in Chrysomelidae (*fide* Seeno and Wilcox 1982: 55).

***Charitonia* Dejean, 1836a: 411**

Originally included available species: none.

***Cheilotoma* Chevrolat, 1836: 420**

Originally included available species: *Chrysomela bucephala* Schaller, 1783 (as “Bucephala. *Fabr*.”).

Type species: *Chrysomela bucephala* Schaller, 1783 (= *Chrysomela musciformis* Goeze, 1777) by monotypy.

Current status: valid genus in Chrysomelidae (*fide* Regalin and Medvedev 2010: 565).

***Chelymorpha* Chevrolat, 1836: 369**

Originally included available species: *Cassida brunnea* Fabricius, 1798; *Cassida cribraria* Fabricius, 1775; *Cassida flavicollis* Klug, 1829 (as “Flavicollis. *Dej*.”); *Cassida gibba* Fabricius, 1798; *Cassida insignis* Klug, 1829; *Cassida multipunctata* Olivier, 1791; *Cassida punctulata* Klug, 1829; *Cassida sexlunata* Klug, 1829; *Cassida variolosa* Olivier, 1791.

Type species: *Cassida multipunctata* Olivier, 1791 (= *Cassida cribraria* Fabricius, 1775) by subsequent designation (Duponchel and Chevrolat 1842b: 211).

Current status: valid genus in Chrysomelidae (*fide* Borowiec 1999: 95).

***Chrysochus* Chevrolat, 1836: 413**

Originally included available species: *Chrysomela asiatica* Pallas, 1771 (as “Asiaticus. *Fabr*.”); *Chrysomela aurata* Fabricius, 1775; *Chrysomela praetiosa* Fabricius, 1792 (as “Pretiosus. *Fabr*.”).

Type species: *Chrysomela praetiosa* Fabricius, 1792 (= *Chrysomela asclepiadea* Pallas, 1773) by subsequent designation (Marseul 1864: xli).

Current status: valid genus in Chrysomelidae (*fide* Moseyko and Sprecher-Uebersax 2010: 631).

Comments. This name was recently placed on the Official List of Generic Names in Zoology in Opinion 2298 (ICZN 2012: 147).

***Chrysopeplis* Dejean, 1836a: 409**

Originally included available species: none.

***Cladophila* Chevrolat, 1836: 430**

Originally included available species: none.

***Cladophora* Dejean, 1836a: 366**

Originally included available species: none.

***Clamophora* Chevrolat, 1836: 388**

Originally included available species: none.

***Coelomera* Chevrolat, 1836: 375**

Originally included available species: *Galeruca bajula* Olivier, 1808; *Chrysomela cayennensis* Fabricius, 1787; *Galleruca coryli* Say, 1824; *Galleruca grossa* Hope, 1831; *Galleruca lanio* Dalman, 1823; *Galleruca nigripennis* Fabricius, 1792; *Galeruca ruficollis* Olivier, 1791.

Type species: *Chrysomela cayennensis* Fabricius, 1787 by subsequent designation (Weise 1924: 51).

Current status: valid genus in Chrysomelidae (*fide* Seeno and Wilcox 1982: 98).

***Colaphus* Dejean, 1836a: 411** (as “Colaphus. *Megerle*.”)

Comments. This name is treated as an unnecessary replacement name for *Colaspidema* Laporte, 1833 [Chrysomelidae].

***Colpodes* Chevrolat, 1836: 394**

Originally included available species: *Altica rotundata* Olivier, 1808.

Type species: *Altica rotundata* Olivier, 1808 by monotypy.

Current status: junior homonym of *Colpodes* Macleay, 1825 [Carabidae]; senior subjective synonym of *Acrocrypta* Baly, 1862 in Chrysomelidae (*fide* Döberl 2010: 491).

***Colposcelis* Dejean, 1836a: 408**

Originally included available species: *Colaspis striatopunctata* Boisduval, 1835 (as “Striatopunctata. *d’Urville*.”); *Colaspis viridiaenea* Gyllenhal, 1808.

Type species: *Colaspis viridiaenea* Gyllenhal, 1808 by subsequent designation (Monrós and Bechyné 1956: 1126).

Current status: junior homonym of *Colposcelis* Dejean, 1834 [Tenebrionidae]; senior subjective synonym of *Pagria* Lefèvre, 1884 in Chrysomelidae (*fide* Moseyko and Sprecher-Uebersax 2010: 642).

***Coptocephala* Chevrolat, 1836: 419**

Originally included available species: *Clytra chalybaea* Germar, 1824 (as “*Chabybea. Germar*.”); *Clytra floralis* Olivier, 1791; *Clytra melanocephala* Olivier, 1808; *Chrysomela quadrimaculata* Linnaeus, 1767 (as “Quadrimaculata. *Fabr*.”); *Chrysomela scopolina* Linnaeus, 1767 (as “Scopolina. *Fabr*.”); *Clythra sexnotata* Fabricius, 1801.

Type species: *Chrysomela scopolina* Linnaeus, 1767 by subsequent designation (Desmarest 1860: 345).

Current status: valid genus in Chrysomelidae (*fide* Regalin and Medvedev 2010: 568).

Comments. The type species designation of *Clytra melanocephala* Olivier, 1808 (= *Cryptocephalus plagiocephalus* Fabricius, 1792) by Jacoby (1908: 174), cited by Regalin and Medvedev (2010: 568), is invalid because of the prior valid typification by Desmarest (1860: 345). Both species are currently included in the genus *Coptocephala* Chevrolat (*fide* Regalin and Medvedev 2010: 568-569).

***Coptocycla* Chevrolat, 1836: 372**

Originally included available species: *Cassida adamantina* Germar, 1824; *Cassida aequinoctialis* Olivier, 1808; *Cassida annulus* Fabricius, 1781 (as “*Annulus. Olivier*.”); *Cassida aurichalcea* Fabricius, 1801; *Cassida bicolon* Germar, 1824 (as “*Bicolor. Germar*.”); *Cassida circularis* Olivier, 1808; *Cassida circumdata* Herbst, 1799; *Cassida diomma* Boisduval, 1835; *Cassida dorso-punctata* Klug, 1829 (as “Dorsopunctata. *Dej*.”); *Cassida flavescens* Latreille, 1813; *Cassida flavolineata* Latreille, 1813; *Cassida graphica* Germar, 1824; *Cassida hebraea* Fabricius, 1781; *Cassida immaculata* Olivier, 1790; *Cassida judaica* Fabricius, 1781; *Cassida polita* Klug, 1829; *Cassida pygmaea* Klug, 1829; *Cassida quadrata* DeGeer, 1775 (as “Quadrata. *Fabr*.”); *Cassida scalaris* Weber, 1801 (as “Scalaris. *Fabr*.”); *Cassida sexguttata* Boisduval, 1835 (as “Sexguttata. *d’Urville*.”); *Cassida sexnotata* Fabricius, 1798; *Cassida sexpunctata* Fabricius, 1781; *Cassida stigma* Germar, 1824; *Cassida tenella* Klug, 1829; *Cassida tristriata* Fabricius, 1792; *Cassida undecimpunctata* Fabricius, 1781; *Cassida zona* Fabricius, 1801.

Type species: *Cassida undecimpunctata* Fabricius, 1781 by subsequent designation (Duponchel and Chevrolat 1842b: 211).

Current status: valid genus in Chrysomelidae (*fide* Borowiec 1999: 358).

***Corynopalpa* Dejean, 1836a: 375**

Originally included available species: *Adorium fasciatum* Olivier, 1807.

Type species: *Adorium fasciatum* Olivier, 1807 by monotypy.

Current status: invalid synonym of *Diacantha* Chevrolat, 1836 in Chrysomelidae (*fide* Beenen 2010: 466).

***Craspedonta* Chevrolat, 1836: 367**

Originally included available species: *Imatidium leayanum* Latreille, 1807 (as “Leyana. *Latreille*.”).

Type species: *Imatidium leayanum* Latreille, 1807 by monotypy.

Current status: valid genus in Chrysomelidae (*fide* Borowiec and Sekerka 2010: 370).

***Crepidodera* Chevrolat, 1836: 391**

Originally included available species: *Altica chrysis* Olivier, 1808; *Crioceris copalina* Fabricius, 1801; *Altica exoleta* Fabricius, 1775; *Haltica femorata* Gyllenhal, 1813; *Galleruca fulvicornis* Fabricius, 1792; *Chrysomela helxines* Linnaeus, 1758 (as “Helxines. *Fabr*.”); *Chrysomela lineata* Rossi, 1790; *Chrysomela modeeri* Linnaeus, 1761 (as “Modeeri. *Fabr*.”); *Haltica nigritula* Gyllenhal, 1813; *Chrysomela nitidula* Linnaeus, 1758 (as “Nitidula. *Fabr*.”); *Haltica pubescens* Knoch, 1803 (as “Pubescens. *Ent. Hefte*.”); *Galleruca ruficornis* Fabricius, 1792; *Chrysomela rufipes* Linnaeus, 1758; *Chrysomela transversa* Marsham, 1802.

Type species: *Chrysomela nitidula* Linnaeus, 1758 by subsequent designation (Maulik 1926: 234).

Current status: valid genus in Chrysomelidae (*fide* Döberl 2010: 510).

***Cyaniris* Chevrolat, 1836: 420**

Originally included available species: *Clytra affinis* Illiger, 1794; *Chrysomela aurita* Linnaeus, 1767 (as “Aurita. *Fabr*.”); *Cryptocephalus collaris* Fabricius, 1781; *Cryptocephalus cyaneus* Fabricius, 1775; *Clytra xanthaspis* Germar, 1824.

Type species: *Cryptocephalus collaris* Fabricius, 1781 by subsequent designation (Monrós 1953: 47).

Current status: junior homonym of *Cyaniris* Dalman, 1816 [Lepidoptera]; invalid subjective synonym of *Smaragdina* Chevrolat, 1836 in Chrysomelidae (*fide* Regalin and Medvedev 2010: 575).

***Cyclodera* Dejean, 1836a: 408**

Originally included available species: none.

***Cyrtocephalus* Dejean, 1836a: 431** (as Cyrtocephalus. *Audouin*.”)

Originally included available species: none.

***Cyrtomorphus* Chevrolat, 1836: 429**

Originally included available species: none.

***Cyrtonota* Chevrolat, 1836: 368**

Originally included available species: *Cassida aenea* Olivier, 1791; *Cassida bipustulata* Linnaeus, 1763 (as “Bipustulata. *Fabr*.”); *Cassida chalybaea* Germar, 1824 (as “*Chalybea. Germar*.”); *Cassida conspersa* Germar, 1824; *Cassida discoides* Linnaeus, 1758 (as “Var. *Discoidea. Fabr*.”); *Cassida discors* Fabricius, 1801; *Cassida festiva* Klug, 1829 (as “Festiva. *Dej*.”); *Cassida gibbosa* Fabricius, 1781; *Cassida illustris* Chevrolat, 1835; *Cassida inaequalis* Linnaeus, 1758 (as “Inaequalis. *Fabr*.”); *Cassida lateralis* Linnaeus, 1758 (as “Lateralis. *Fabr*.”); *Cassida obsoleta* Olivier, 1808; *Cassida reticularis* Linnaeus, 1758 (as “Reticularis. *Fabr*.”); *Cassida sexpustulata* Fabricius, 1881.

Type species: *Cassida lateralis* Linnaeus, 1758 by subsequent designation (Duponchel and Chevrolat 1842b: 211).

Current status: valid genus in Chrysomelidae (*fide* Borowiec 1999: 105).

***Damia* Dejean, 1836a: 419**

Originally included available species: none.

***Dasymallus* Chevrolat, 1836: 384**

Originally included available species: none.

***Delocrania* Dejean, 1836a: 367**

Originally included available species: none.

***Deloyala* Chevrolat, 1836: 371**

Originally included available species: *Cassida adhaerens* Weber, 1801 (as “Adhaerens. *Fabr*.”); *Cassida clavata* Fabricius, 1798; *Cassida cruciata* Linnaeus, 1758 (as “*Cruciata. Olivier*. ”); *Cassida crux* Fabricius, 1781; *Cassida diaphana* Sahlberg, 1823; *Cassida divisa* Boisduval, 1835 (as “Divisa. *d’Urville*.”); *Cassida dorsata* Fabricius, 1787; *Cassida elatior* Klug, 1829; *Cassida elevata* Fabricius, 1801; *Cassida fuliginosa* Olivier, 1808; *Cassida micans* Fabricius, 1801; *Cassida miliaris* Fabricius, 1775; *Cassida punctum* Fabricius, 1801; *Cassida quinquefasciata* Fabricius, 1801; *Cassida signifera* Herbst, 1799; *Cassida tredecimpunctata* Fabricius, 1801; *Cassida tuberculata* Fabricius, 1775.

Type species: *Cassida crux* Fabricius, 1781 (= *Cassida cruciata* Linnaeus, 1758) by subsequent designation (Duponchel and Chevrolat 1842b: 211).

Current status: valid genus in Chrysomelidae (*fide* Borowiec 1999: 370).

***Delphus* Dejean, 1836a: 427**

Originally included available species: none.

***Deuterocampta* Chevrolat, 1836: 397**

Originally included available species: *Chrysomela dissecta* Germar, 1824; *Chrysomela semistriata* Fabricius, 1775; *Chrysomela stauroptera* Wiedemann, 1821 (as “Stauroptera. *Germar*.”); *Chrysomela vinculata* Germar, 1824.

Type species: *Chrysomela stauroptera* Wiedemann, 1821 by subsequent designation (Chevrolat 1843: 656).

Current status: valid genus in Chrysomelidae (*fide* Seeno and Wilcox 1982: 79).

***Dia* Dejean, 1836a: 411**

Comments. This name is treated as an unnecessary replacement name for *Colaspidea* Laporte, 1833 [Chrysomelidae].

***Diabrotica* Chevrolat, 1836: 380**

Originally included available species: *Crioceris abrupta* Fabricius, 1801; *Crioceris bivittata* Fabricius, 1801; *Crioceris capitata* Fabricius, 1801; *Crioceris cyanipennis* Fabricius, 1801; *Altica decempunctata* Latreille, 1813; *Chrysomela duodecimpunctata* Fabricius, 1775; *Crioceris elata* Fabricius, 1801; *Crioceris fucata* Fabricius, 1787; *Cistela innuba* Fabricius, 1775; *Crioceris liciens* Fabricius, 1801; *Crioceris ochreata* Fabricius, 1792 (as “Ocreata. *Fabr*.”); *Galeruca pallipes* Olivier, 1791; *Galeruca quadrilineata* Latreille, 1813; *Galeruca quadrivittata* Latreille, 1813; *Galeruca quinquelineata* Latreille, 1813; *Crioceris quinquemaculata* Fabricius, 1801; *Crioceris ruficollis* Fabricius, 1801; *Galeruca scripta* Olivier, 1808; *Altica sinuata* Olivier, 1789; *Galeruca speciosa* Germar, 1824; *Crioceris thoracica* Fabricius, 1801; *Crioceris tripunctata* Fabricius, 1801; *Crioceris vittata* Fabricius, 1775.

Type species: *Crioceris fucata* Fabricius, 1787 by subsequent designation (Barber 1947b: 151).

Current status: valid genus in Chrysomelidae (*fide* Riley et al. 2003: 78).

***Diacantha* Chevrolat, 1836: 378**

Originally included available species: *Chrysomela picea* Fabricius, 1781; *Galeruca unifasciata* Olivier, 1808.

Type species: *Galleruca unifasciata* Olivier, 1808 by subsequent designation (Hincks 1949: 613).

Current status: valid genus in Chrysomelidae (*fide* Beenen 2010: 466).

***Diphaulaca* Chevrolat, 1836: 388**

Originally included available species: *Altica aulica* Olivier, 1808; *Altica janthinipennis* Latreille, 1813; *Haltica striata* Klug, 1829.

Type species: *Altica aulica* Olivier, 1808 by subsequent designation (Chevrolat 1844: 46).

Current status: valid genus in Chrysomelidae (*fide* Seeno and Wilcox 1982: 138).

***Discomorpha* Chevrolat, 1836: 368**

Originally included available species: *Cassida palliata* Fabricius, 1787; *Cassida variegata* Linnaeus, 1758 (as “Variegata. *Fabr*.”).

Type species: *Cassida variegata* Linnaeus, 1758 by subsequent designation (Duponchel and Chevrolat 1842b: 211).

Current status: valid genus in Chrysomelidae (*fide* Borowiec 1999: 44).

***Disonycha* Chevrolat, 1836: 390**

Originally included available species: *Haltica alternata* Illiger sensu Latreille, 1813; *Crioceris caroliniana* Fabricius, 1775; *Galleruca collaris* Fabricius, 1798; *Crioceris collata* Fabricius, 1801; *Haltica conjuncta* Germar, 1824; *Crioceris glabrata* Fabricius, 1781; *Haltica quadrivittata* Illiger, 1807; *Chrysomela tricolor* Fabricius, 1781.

Type species: *Crioceris collata* Fabricius, 1801 by subsequent designation (Blake 1933: 1).

Current status: valid genus in Chrysomelidae (*fide* Riley et al. 2003: 120).

***Disopus* Chevrolat, 1836: 425**

Originally included available species: *Chrysomela pini* Linnaeus, 1758 (as “Pini. *Fabr*.”).

Type species: *Chrysomela pini* Linnaeus, 1758 by monotypy.

Current status: valid subgenus of *Cryptocephalus* Geoffroy, 1762 in Chrysomelidae (*fide* Lopatin et al. 2010: 604).

***Ditropidus* Chevrolat, 1836: 425**

Originally included available species: none.

***Dorylas* Dejean,** 1836a**: 409**

Originally included available species: none.

***Dorynota* Chevrolat, 1836: 370**

Originally included available species: *Cassida bidens* Fabricius, 1781; *Cassida pugionata* Germar, 1824 (as “*Pugionata. Hoffmansegg*.”); *Cassida truncata* Fabricius, 1781.

Type species: *Cassida bidens* Fabricius, 1781 by subsequent designation (Duponchel and Chevrolat 1842b: 211).

Current status: valid genus in Chrysomelidae (*fide* Borowiec 1999: 161).

***Echoma* Chevrolat, 1836: 370**

Originally included available species: *Cassida basalis* Germar, 1824; *Cassida dichroa* Germar, 1824; *Cassida irrorata* Fabricius, 1801; *Cassida marginata* Linnaeus, 1767 [no 23] (as “Marginata. *Fabr*.”); *Cassida normalis* Germar, 1824; *Cassida suturalis* Fabricius, 1777.

Type species: *Cassida marginata* Linnaeus, 1767 (= *Cassida clypeata* Panzer, 1798) by subsequent designation (Duponchel and Chevrolat 1842b: 211).

Current status: valid genus in Chrysomelidae (*fide* Borowiec 1999: 112).

Comments. Linnaeus (1758: 363) described a *Cassida marginata* from “America.” In 1767, Linnaeus described two *Cassida marginata*, one (no 14 on p. 576) from “America,” identical to his species of 1758, and one (no 23 on p. 578) from “India occidentali [= West Indies].” According to Borowiec (1999: 101, 113), *Cassida marginata* (no 14) is *Chelymorpha marginata* (Linnaeus, 1758) and the second *Chelymorpha marginata* (no 23) is a synonym of *Echoma clypeata* (Panzer, 1798). It is impossible to know which species Chevrolat (1836: 370) had in mind when he listed “*marginata* Fabr.” However, to promote stability we accept *Chelymorpha marginata* Linnaeus, 1767 as the species included in Dejean’s catalogue.

***Ecthrophyta* Dejean, 1836a: 379**

Originally included available species: none.

***Edusa* Chevrolat, 1836: 408**

Originally included available species: *Colaspis varipes* Boisduval, 1835 (as “Varipes. *Latreille*.”).

Type species: *Colaspis varipes* Boisduval, 1835 by monotypy.

Current status: senior synonym of *Edusella* Chapuis, 1874 in Chrysomelidae (*fide* Seeno and Wilcox 1982: 59).

Comments. *Edusa* Chevrolat, 1836 has precedence over *Edusella* Chapuis, 1874 which is currently used as valid (e.g., Seeno and Wilcox 1982: 59). Reversal of Precedence (ICZN 1999: Article 23.9) cannot be used because *Edusa* Chevrolat was used as valid after 1899 (e.g., Weise 1923: 53). Therefore an application to the Commission is necesssary to conserve usage of the name *Edusella* Chapuis.

***Ellipticus* Chevrolat, 1836: 426**

Originally included available species: *Erotylus immaculatus* Olivier, 1807; *Erotylus lineaticollis* Duponchel, 1825 (as “*Lineatocollis. Dej*.”); *Erotylus pallidus* Olivier, 1791; *Erotylus testaceus* Fabricius, 1775.

Type species: *Erotylus testaceus* Fabricius, 1775 by subsequent designation (Alvarenga 1965: 83).

Current status: valid genus in Erotylidae (*fide* Alvarenga 1965: 83).

Comments. As mentioned by Alvarenga (1965: 83), *Ellipticus* Chevrolat, 1836 is a senior synonym of *Omoiotelus* Hope, 1841.

***Elytrogona* Chevrolat, 1836: 370**

Originally included available species: *Cassida ampulla* Olivier, 1808; *Cassida quatuordecimmaculata* Latreille, 1802.

Type species: *Cassida ampulla* Olivier, 1808 (= *Cassida quatuordecimmaculata* Latreille, 1802) by monotypy.

Current status: valid genus in Chrysomelidae (*fide* Borowiec 1999: 114).

Comments. The name *quatuordecimmaculata* is listed in synonymy with *ampulla* in Dejean’s catalogue; therefore the type species *Elytrogona* is *ampulla* by monotypy (ICZN 1999: Article 68.3).

***Elytrosphaera* Chevrolat, 1836: 397**

Originally included available species: none.

***Endocephalus* Chevrolat, 1836: 412**

Originally included available species: *Eumolpus bigatus* Germar, 1824; *Eumolpus maculatus* Germar, 1824.

Type species: *Eumolpus bigatus* Germar, 1824 (= *Cryptocephalus lineatus* Fabricius, 1775) by subsequent designation (Monrós and Bechyné 1956: 1127).

Current status: valid genus in Chrysomelidae (*fide* Seeno and Wilcox 1982: 60).

Comments. The type species of *Endocephalus* listed by Bechyné (1953: 274), *Eumolpus octopunctatus* Germar, 1824, is a *species inquirandum* in Dejean’s catalogue and therefore not an originally included species (ICZN 1999: Article 67.2.5).

***Entomoscelis* Chevrolat, 1836: 402**

Originally included available species: *Chrysomela adonidis* Pallas, 1771 (as “Adonidis. *Fabr*.”); *Chrysomela cincta* Olivier, 1790; *Chrysomela dorsalis* Fabricius, 1777; *Chrysomela senegalensis* Fabricius, 1792.

Type species: *Chrysomela adonidis* Pallas, 1771 by subsequent designation (Chevrolat 1843: 654).

Current status: valid genus in Chrysomelidae (*fide* Kippenberg 2010: 428).

***Epytus* Dejean, 1836a: 428**

Originally included available species: *Erotylus cyaneus* Duponchel, 1825; *Erotylus violaceus* Sturm, 1826.

Type species: *Erotylus violaceus* Sturm, 1826 (= *Erotylus cyaneus* Duponchel, 1825) by subsequent designation (Crotch 1876: 433).

Current status: valid genus in Erotylidae (*fide* Alvarenga 1965: 84).

Comments. As mentioned by Alvarenga (1965: 84), *Epytus* Dejean, 1836 is a senior synonym of *Oocyanus* Hope, 1841.

***Eubrachis* Dejean, 1836a: 414**

Comments. This name is treated as an unnecessary replacement name for *Pseudocolaspis* Laporte, 1833 [Chrysomelidae].

***Euclada* Dejean, 1836a: 382**

Originally included available species: none.

***Eugenysa* Chevrolat, 1836: 368**

Originally included available species: *Cassida grossa* Linnaeus, 1758 (as “Grossa. *Fabr*.”).

Type species: *Cassida grossa* Linnaeus, 1758 by monotypy.

Current status: valid genus in Chrysomelidae (*fide* Borowiec 1999: 79).

***Eugonycha* Chevrolat, 1836: 404**

Originally included available species: none.

***Euparocha* Dejean, 1836a: 399**

Originally included available species: none.

***Euprionota* Chevrolat, 1836: 365**

Originally included available species: none.

***Eva* Dejean, 1836a: 411**

Originally included available species: none.

***Exora* Chevrolat, 1836: 379**

Originally included available species: *Crioceris obsoleta* Fabricius, 1801; *Crioceris olivacea* Fabricius, 1801.

Type species: *Crioceris olivacea* Fabricius, 1801 by subsequent designation (Hincks 1949: 615).

Current status: valid genus in Chrysomelidae (*fide* Seeno and Wilcox 1982: 103).

***Fatua* Dejean, 1836a: 430**

Originally included available species: *Languria longicornis* Wiedemann, 1823.

Type species: *Languria longicornis* Wiedemann, 1823 by monotypy.

Current status: valid genus in Erotylidae (*fide* Fowler 1908: 16).

***Fidia* Dejean, 1836a: 412**

Originally included available species: none.

***Gamelia* Dejean, 1836a: 430**

Originally included available species: none.

***Gastrophysa* Chevrolat, 1836: 405**

Originally included available species: *Chrysomela polygoni* Linnaeus, 1758 (as “Polygoni. *Fabr*.”); *Chrysomela raphani* Herbst, 1783 (as “Raphani. *Fabr*.”); *Chrysomela viridula* DeGeer, 1775 (as “*Viridula. Olivier*.”).

Type species: *Chrysomela polygoni* Linnaeus, 1758 by subsequent designation (Chevrolat 1845: 34).

Current status: valid genus in Chrysomelidae (*fide* Kippenberg 2010: 393).

***Glyptoscelis* Chevrolat, 1836: 414**

Originally included available species: *Cryptocephalus aeneus* Wiedemann, 1821; *Eumolpus hirtus* Olivier, 1808.

Type species: *Eumolpus hirtus* Olivier, 1808 by subsequent designation (Monrós and Bechyné 1956: 1127).

Current status: valid genus in Chrysomelidae (*fide* Riley et al. 2003: 145).

***Goniocephala* Chevrolat, 1836: 430**

Originally included available species: none.

***Gonioctena* Chevrolat, 1836: 403**

Originally included available species: *Chrysomela affinis* Gyllenhal, 1808 (as “Affinis. *Schönherr*.”); *Chrysomela decempunctata* Linnaeus, 1758 (as “Decempunctata. *Fabr*.”); *Chrysomela dispar* Paykull, 1799; *Chrysomela haemorrhoidalis* Linnaeus, 1758 (as “Var. *Haemorrhoidalis. Fabr*.”); *Chrysomela pallida* Linnaeus, 1758 (as “Pallida. *Fabr*.”); *Chrysomela rufipes* DeGeer, 1775 (as “*Rufipes. Paykull*.”); *Chrysomela viminalis* Linnaeus, 1758 (as “Viminalis. *Fabr*.”).

Type species: *Chrysomela viminalis* Linnaeus, 1758 by subsequent designation (Thomson 1859: 158).

Current status: valid genus in Chrysomelidae (*fide* Kippenberg 2010: 432).

***Gonophora* Chevrolat, 1836: 366**

Originally included available species: *Hispa haemorrhoidalis* Weber, 1801 (as “Haemorrhoidalis. *Fabr*.”).

Type species: *Hispa haemorrhoidalis* Weber, 1801 by monotypy.

Current status: valid genus in Chrysomelidae (*fide* Seeno and Wilcox 1982: 164).

***Graptodera* Chevrolat, 1836: 388**

Originally included available species: *Altica aenea* Olivier, 1808; *Galeruca caerulea* Olivier, 1791; *Haltica carinata* Germar, 1824; *Altica chrysoptera* Latreille, 1813; *Altica cyanea* Weber, 1801; *Galleruca erucae* Fabricius, 1792; *Haltica indigacea* Illiger, 1807; *Haltica janthina* Illiger, 1807; *Galleruca mercurialis* Fabricius, 1792; *Chrysomela oleracea* Linnaeus, 1758 (as “Oleracea. *Fabr*.”); *Galeruca plebeja* Olivier, 1808.

Type species: *Chrysomela oleracea* Linnaeus, 1758 by subsequent designation (Chevrolat 1845: 307).

Current status: junior objective synonym of *Altica* Geoffroy, 1762 in Chrysomelidae (*fide* Döberl 2010: 492).

***Guyanica* Chevrolat, 1836: 409**

Originally included available species: *Colaspis octoguttata* Olivier, 1808; *Colaspis pallida* Olivier, 1808; *Colaspis unipunctata* Olivier, 1808.

Type species: *Colaspis octoguttata* Olivier, 1808 by subsequent designation (Monrós and Bechyné 1956: 1125).

Current status: valid genus in Chrysomelidae (*fide* Seeno and Wilcox 1982: 57).

***Hadrocera* Dejean, 1836a: 375**

Originally included available species: none.

***Hemipyxis* Dejean, 1836a: 387**

Originally included available species: *Altica troglodytes* Olivier, 1808.

Type species: *Altica troglodytes* Olivier, 1808 (= *Haltica fulvipennis* Illiger, 1807) by monotypy.

Current status: valid genus in Chrysomelidae (*fide* Döberl 2010: 515, as “*Hemipyxis* Chevrolat, 1836”).

***Hemisphaerota* Chevrolat, 1836: 367**

Originally included available species: *Cassida erythrocera* Germar, 1824.

Type species: *Cassida erythrocera* Germar, 1824 (= *Imatidium cyaneum* Say, 1824) by monotypy.

Current status: valid genus in Chrysomelidae (*fide* Borowiec 1999: 28).

***Hersilia* Dejean, 1836a: 412**

Originally included available species: *Brevicolaspis pilosa* Laporte, 1833.

Type species: *Brevicolaspis pilosa* Laporte, 1833 by monotypy.

Current status: junior objective synonym of *Brevicolaspis* Laporte, 1833 in Chrysomelidae (*fide* Seeno and Wilcox 1982: 62).

***Heteraspis* Chevrolat, 1836: 413**

Originally included available species: *Eumolpus vittatus* Olivier, 1808.

Type species: *Eumolpus vittatus* Olivier, 1808 by monotypy.

Current status: valid genus in Chrysomelidae (*fide* Moseyko and Sprecher-Uebersax 2010: 623).

***Homalopus* Chevrolat, 1836: 422**

Originally included available species: none.

***Hybosa* Chevrolat, 1836: 372**

Originally included available species: none.

***Hygrotophila* Chevrolat, 1836: 431**

Originally included available species: *Tritoma piliferum* Müller, 1821 (as “Piligera. *Müller*.”).

Type species: *Tritoma piliferum* Müller, 1821 by monotypy.

Current status: junior subjective synonym of *Sphaerosoma* Stephens, 1832 in Alexiidae (*fide* Tomaszewska 2007: 555, as “*Hygrotophila* Champion, 1887”).

***Hylax* Dejean, 1836a: 409**

Originally included available species: none.

***Hypsomorpha* Dejean, 1836a: 375**

Originally included available species: none.

***Iphiclus* Chevrolat, 1836: 426**

Originally included available species: *Erotylus apiatus* Chevrolat, 1835; *Erotylus conspersus* Duponchel, 1825; *Erotylus decemnotatus* Duponchel, 1825; *Erotylus flavovittatus* Duponchel, 1825 (as “Flavovittatus. *Dej*.”); *Erotylus guttatus* Duponchel, 1825 (as “Guttatus. *Dej*.”); *Erotylus praeustus* Duponchel, 1825 (as “Praeustus. *Dej*.”); *Erotylus quinquepunctatus* Fabricius, 1775; *Erotylus rubidus* Duponchel, 1825 (as “Rubidus. *Dej*.”); *Erotylus sexdecimguttatus* Olivier, 1791; *Erotylus sexpunctatus* Duponchel, 1825 (as “Sexpunctatus. *Dej*.”); *Erotylus vigintiguttatus* Duponchel, 1825 (as “Vigintiguttatus. *Dej*.”).

Type species: *Erotylus flavovittatus* Duponchel, 1825 by subsequent designation (Alvarenga 1965: 85).

Current status: valid genus in Erotylidae (*fide* Alvarenga 1965: 85).

Comments. As mentioned by Alvarenga (1965: 85), *Iphiclus* Chevrolat, 1836 is a senior synonym of *Brachysphaenus* Lacordaire, 1842.

***Iscadida* Dejean, 1836a: 399**

Originally included available species: none.

***Ischiopachys* Chevrolat, 1836: 416**

Originally included available species: *Clytra bicolor* Olivier, 1791.

Type species: *Clytra bicolor* Olivier, 1791 by monotypy.

Current status: valid genus in Chrysomelidae (*fide* Seeno and Wilcox 1982: 35).

***Ischyrosonyx* Chevrolat, 1836: 370**

Originally included available species: none.

***Ischyrus* Chevrolat, 1836: 428**

Originally included available species: *Erotylus semipunctatus* Germar, 1824; *Erotylus undatus* Olivier, 1791.

Type species: *Erotylus undatus* Olivier, 1791 by subsequent designation (Alvarenga 1965: 85).

Current status: name suppressed in Erotylidae.

Comments. *Ischyrus* Chevrolat, 1836 was suppressed for the purposes of the Principles of Priority and Homonymy in Opinion 1824 (ICZN 1996a).

***Janessa* Chevrolat, 1836: 430**

Originally included available species: *Languria thoracica* Olivier, 1807.

Type species: *Languria thoracica* Olivier, 1807 (= *Trogosita bicolor* Fabricius, 1798) by monotypy.

Current status: junior subjective synonym of *Languria* Latreille, 1802 in Erotylidae (*fide* Leschen and Skelley 2002: 345).

***Labidognatha* Dejean, 1836a: 419**

Originally included available species: *Cryptocephalus coerulans* Fabricius, 1781.

Type species: *Cryptocephalus coerulans* Fabricius, 1781 by monotypy.

Current status: valid subgenus of *Coptocephala* Chevrolat, 1836 in Chrysomelidae (*fide* Seeno and Wilcox 1982: 33).

***Labidomera* Chevrolat, 1836: 397**

Originally included available species: *Chrysomela trimaculata* Linnaeus *sensu* Fabricius, 1775 (as “Trimaculata. *Fabr*.”).

Type species: *Chrysomela trimaculata* Linnaeus *sensu* Fabricius, 1775 (= *Labidomera trimaculata* Chevrolat, 1836) by monotypy.

Current status: valid genus in Chrysomelidae (*fide* Seeno and Wilcox 1982: 79).

Comments. *Chrysomela trimaculata* Fabricius (1775: 95) is usually listed as a valid species but is not an available species since Fabricius referred to Linnaeus (1767: 592) when describing the species (as “*Linn. Syst. Nat*. 11. 592. 45”). The true *Chrysomela trimaculata* Linnaeus is the coccinellid *Hyperaspidius trimaculatus* (Linnaeus, 1767). It is obvious that Chevrolat (1836: 397) used the name *trimaculata* in the sense of Fabricius (1775: 95). According to ICZN (1999: Article 11.10), an author, who employs a specific name for the type species of a new nominal genus-group taxon deliberately in the sense of a previous misidentification of it, is deemed to have denoted a new nominal species, with its own author and date as though it were newly proposed in combination with the new genus-group name. Therefore Chevrolat (1836: 397) indirectly proposed the name *Labidomera trimaculata*, which is a senior synonym of *Chrysomela clivicollis* Kirby, 1837 (**new synonymy**). An application to the Commission is necessary to conserve the name *Chrysomela clivicollis* Kirby, 1837 as a valid name.

***Labidostomis* Chevrolat, 1836: 418**

Originally included available species: *Clytra cyanicornis* Germar, 1817 (as “Cyanicornis. *Dahl*.”); *Cryptocephalus hordei* Fabricius, 1787; *Clytra humeralis* Schneider, 1792 (as “Humeralis. *Panzer*.”); *Chrysomela longimana* Linnaeus, 1760 (as “Longimana. *Fabr*.”); *Clythra notata* Gebler, 1830; *Clythra pallidipennis* Gebler, 1830; *Cryptocephalus taxicornis* Fabricius, 1792; *Chrysomela tridentata* Linnaeus, 1758.

Type species: *Chrysomela longimana* Linnaeus, 1760 by subsequent designation (Thomson 1859: 158).

Current status: valid genus in Chrysomelidae (*fide* Regalin and Medvedev 2010: 570).

Comments. The type species designation of *Cryptocephalus taxicornis* Fabricius, 1792 by Jacoby (1908: 96), cited by Regalin and Medvedev (2010: 570), is invalid because of the prior valid typification by Thomson (1859: 158). Both species are currently included in the nominotypical subgenus of *Labidostomis* Chevrolat (*fide* Regalin and Medvedev 2010: 571–572).

***Lachnaia* Chevrolat, 1836: 418**

Originally included available species: *Clytra cerealis* Olivier, 1808; *Cryptocephalus lentisci* Fabricius, 1792; *Cryptocephalus longipes* Fabricius, 1775; *Clytra paradoxa* Olivier, 1808; *Cryptocephalus tripunctatus* Fabricius, 1792; *Chrysomela variolosa* Linnaeus, 1767.

Type species: *Chrysomela variolosa* Linnaeus, 1767 by subsequent designation (Monrós 1953: 46).

Current status: valid genus in Chrysomelidae (*fide* Regalin and Medvedev 2010: 573).

***Lacpatica* Chevrolat, 1836: 389**

Originally included available species: none.

***Laertes* Dejean, 1836a: 413**

Originally included available species: none.

***Lamprotheca* Dejean, 1836a: 409**

Originally included available species: none.

***Leioplacis* Dejean, 1836a: 404**

Originally included available species: none.

***Leiopomis* Dejean, 1836a: 387**

Originally included available species: none.

***Lepronota* Chevrolat, 1836: 408**

Originally included available species: none.

***Lepropterus* Dejean, 1836a: 414**

Originally included available species: none.

***Leptinotarsa* Chevrolat, 1836: 397**

Originally included available species: none.

Comments. This name was conserved in Opinion 1290 by the International Commission on Zoological Nomenclature and placed on the Official List of Generic Names in Zoology (ICZN 1985a) as “*Leptinotarsa* Chevrolat, 1837.” Its type species is *Leptinotarsa heydenii* Stål, 1858.

***Leptomorpha* Chevrolat, 1836: 366**

Originally included available species: none.

***Leucocera* Chevrolat, 1836: 404**

Originally included available species: *Chrysomela decempustulata* Fabricius, 1792.

Type species: *Chrysomela decempustulata* Fabricius, 1792 by monotypy.

Current status: valid genus in Chrysomelidae (*fide* Seeno and Wilcox 1982: 79).

***Lisias* Dejean, 1836a: 410**

Originally included available species: none.

***Lithonoma* Chevrolat, 1836: 384**

Originally included available species: *Galleruca marginella* Fabricius, 1801.

Type species: *Galleruca marginella* Fabricius, 1801 (= *Chrysomela cincta* Fabricius, 1781) by monotypy.

Current status: junior objective synonym of *Oedionychis* Latreille, 1829 in Chrysomelidae (*fide* Döberl 2010: 541).

***Litosonycha* Chevrolat, 1836: 387**

Originally included available species: *Haltica decipiens* Klug, 1829 (as “Decipiens. *Dej*.”).

Type species: *Haltica decipiens* Klug, 1829 by monotypy.

Current status: senior synonym of *Asphaera* Duponchel and Chevrolat, 1842 (*fide* Riley et al. 2003: 125, as “*Litosonycha* H. Clark, 1865”).

Comments. This genus is attributed to Clark (1865: 377) in the literature (e.g., Seeno and Wilcox 1982: 140; Riley et al. 2003: 125). *Litosonycha* Chevrolat, 1836 has precedence over *Asphaera* Duponchel and Chevrolat, 1842. Reversal of Precedence (ICZN 1999: Article 23.9) or an application to the Commission is necessary to conserve usage of the name *Asphaera* Duponchel and Chevrolat, 1842.

***Lybas* Chevrolat, 1836: 429**

Originally included available species: *Erotylus lesueuri* Chevrolat, 1835 (as “*Lesueurii*. *Chevrolat*.”); *Erotylus melanophtalmus* Duponchel, 1825 (as “Melanophthalmus. *Dej*.”); *Erotylus sanguineus* Duponchel, 1825 (as “Sanguineus. *Dej*.”).

Type species: *Erotylus lesueuri* Chevrolat, 1835 by subsequent designation (Alvarenga 1965: 85).

Current status: name suppressed in Erotylidae.

Comments. This name was suppressed for the purposes of the Principles of Priority and Homonymy in Opinion 1824 (ICZN 1996a).

***Macrolenes* Chevrolat, 1836: 419**

Originally included available species: *Clytra biguttata* Olivier, 1791; *Cryptocephalus bimaculatus* Fabricius, 1781 (as “*Bimaculata. Rossi*.”); *Clytra dentipes* Olivier, 1808; *Clytra macropus* Illiger, 1800; *Cryptocephalus maxillosus* Fabricius, 1781; *Clytra novempunctata* Dufour, 1820; *Cryptocephalus octopunctatus* Fabricius, 1787; *Clytra ruficollis* Olivier, 1791; *Cryptocephalus ruficollis* Fabricius, 1792; *Cryptocephalus sexmaculatus* Fabricius, 1781; *Clytra sexpunctata* Olivier, 1808.

Type species: *Cryptocephalus ruficollis* Fabricius, 1792 (= *Clytra dentipes* Olivier, 1808) by subsequent designation (Monrós 1953: 45).

Current status: valid genus in Chrysomelidae (*fide* Regalin and Medvedev 2010: 574).

***Malacosoma* Chevrolat, 1836: 379**

Originally included available species: *Crioceris abdominalis* Schönherr, 1808; *Chrysomela lusitanica* Linnaeus, 1767 (as “Lusitanica. *Olivier*.”); *Galeruca nigripes* Olivier, 1791 (as “*Nigripes. Encyclopédie*.”); *Cistela testacea* Fabricius, 1775.

Type species: *Chrysomela lusitanica* Linnaeus, 1767 by monotypy.

Current status: junior homonym of *Malacosoma* Hübner, 1820 [Lepidoptera]; senior objective synonym of *Exosoma* Jacoby, 1903 in Chrysomelidae (*fide* Beenen 2010: 475).

Comments. The names *abdominalis*, *nigripes*, and *testacea* are listed in synonymy with *lusitanica* in Dejean’s catalogue; therefore the type species of *Malacosoma* is *lusitanica* by monotypy (ICZN 1999: Article 68.3).

***Megalostomis* Chevrolat, 1836: 416**

Originally included available species: *Clytra auricapilla* Germar, 1824; *Clytra bicincta* Germar, 1824; *Clytra boopis* Germar, 1824 (as “Boopis. *Hoffmansegg*.”); *Clythra cingulata* Latreille, 1809; *Clythra dominicana* Fabricius, 1801; *Clytra tetrastigma* Germar, 1824.

Type species: *Clytra boopis* Germar, 1824 by subsequent designation (Monrós 1953: 46).

Current status: valid genus in Chrysomelidae (*fide* Riley et al. 2003: 179).

***Melina* Chevrolat, 1836: 409**

Originally included available species: none.

***Melitonoma* Chevrolat, 1836: 419**

Originally included available species: *Cryptocephalus pallens* Fabricius, 1787.

Type species: *Cryptocephalus pallens* Fabricius, 1787 by monotypy.

Current status: valid genus in Chrysomelidae (*fide* Regalin and Medvedev 2010: 574).

Comments. Monrós (1953: 46) designated *Clytra decempunctata* Olivier, 1808 as type species of *Melitonoma* Chevrolat and this species is the type species listed for this genus by Regalin and Medvedev (2010: 574). However, *Clytra decempunctata* Olivier is listed as a *species inquirendum* in Dejean’s catalogue. The sole available species listed by Chevrolat (1836: 419) is *Cryptocephalus pallens* Fabricius and this species is currently included in the genus *Diapromorpha* Lacordaire, 1848 (Regalin and Medvedev 2010: 569). A request to the Commission is necessary to retain *Clytra decempunctata* Olivier as type species of *Melitonoma* Chevrolat.

***Menalcas* Dejean, 1836a: 413**

Originally included available species: none.

***Metachroma* Chevrolat, 1836: 412**

Originally included available species: *Eumolpus aterrimus* Olivier, 1808; *Cryptocephalus canellus* Fabricius, 1801; *Colaspis quadrinotata* Say, 1824; *Colaspis quercata* Fabricius, 1801.

Type species: *Colaspis quercata* Fabricius, 1801 by subsequent designation (Crotch 1873a: 41).

Current status: valid genus in Chrysomelidae (*fide* Riley et al. 2003: 135).

***Metaxyonycha* Chevrolat, 1836: 406** (as “Metazyonycha. *Chevrolat*.”)

Originally included available species: *Colaspis chloroptera* Germar, 1824; *Colaspis granulata* Germar, 1821; *Colaspis quadrimaculata* Olivier, 1808; *Colaspis testacea* Fabricius, 1801.

Type species: *Colaspis testacea* Fabricius, 1801 by subsequent designation (Monrós and Bechyné 1956: 1125).

Current status: valid genus in Chrysomelidae (*fide* Seeno and Wilcox 1982: 58).

Comments. The spelling *Metaxyonycha* is an incorrect subsequent spelling of *Metazyonycha* in prevailing usage and attributed to the publication of the original spelling; therefore *Metaxyonycha* is deemed to be the correct original spelling (ICZN 1999: Article 33.3.1).

***Metazycera* Chevrolat, 1836: 364**

Originally included available species: *Hispa trimaculata* Olivier, 1808.

Type species: *Hispa trimaculata* Olivier, 1808 by monotypy.

Current status: valid genus in Chrysomelidae (*fide* Staines 2010: 2).

***Microdonta*Chevrolat 1836: 364**

Originally included available species: *Hispa serraticornis* Fabricius, 1792.

Type species: *Hispa serraticornis* Fabricius, 1792 by monotypy.

Current status: invalid synonym of *Sceloenopla* Chevrolat, 1836 in Chrysomelidae (*fide* Seeno and Wilcox 1982: 159).

***Microrhopala* Chevrolat, 1836: 365**

Originally included available species: *Hispa excavata* Olivier, 1808; *Hispa vittata* Fabricius, 1798.

Type species: *Hispa vittata* Fabricius, 1798 by subsequent designation (Monrós and Bechyné 1956: 1135).

Current status: valid genus in Chrysomelidae (*fide* Seeno and Wilcox 1982: 161).

***Microtheca* Dejean, 1836a: 395**

Originally included available species: none.

***Monachus* Chevrolat, 1836: 425**

Originally included available species: *Cryptocephalus saponatus* Fabricius, 1801.

Type species: *Cryptocephalus saponatus* Fabricius, 1801 by monotypy.

Current status: junior homonym of *Monachus* Fleming, 1822 [Mammalia]; senior objective synonym of *Lexiphanes* Gistel, 1848 in Chrysomelidae (*fide* Seeno and Wilcox 1982: 38).

***Monolepta* Chevrolat, 1836: 383**

Originally included available species: *Crioceris apicalis* Sahlberg, 1823; *Crioceris bioculata* Fabricius, 1781; *Crioceris humeralis* Fabricius, 1801; *Altica limbata* Olivier, 1808; *Galleruca luteicollis* Boisduval, 1835 (as “Luteicollis. *d’Urville*.”); *Crioceris neglecta* Sahlberg, 1823; *Crioceris quadrinotata* Fabricius, 1801; *Crioceris rubra* Gyllenhal, 1808 (as “Rubra. *Schönherr*.”); *Crioceris semicincta* Sahlberg, 1823; *Galleruca subsulcata* Boisduval, 1835 (as “Subsulcata. *d’Urville*.”).

Type species: *Crioceris bioculata* Fabricius, 1781 by subsequent designation (Chevrolat 1845: 5).

Current status: valid genus in Chrysomelidae (*fide* Beenen 2010: 482).

***Monomacra* Chevrolat, 1836: 389**

Originally included available species: *Haltica capitata* Illiger, 1807; *Haltica inermis* Klug, 1829; *Altica tibialis* Olivier, 1808.

Type species: *Haltica inermis* Klug, 1829 by subsequent designation (Monrós and Bechyné 1956: 1133).

Current status: valid genus in Chrysomelidae (*fide* Riley et al. 2003: 124).

Comments. The first valid type species designation for *Monomacra* Chevrolat is that of Chevrolat (1845: 6) who selected *Altica tibialis* Olivier, 1808. This species is currently included in the genus *Parchicola* Bechyné and Springlovà de Bechyné (Riley et al. 2003: 124). An application to the Commission is needed to conserve *Haltica inermis* Klug as type species of this genus.

***Monoplatus* Chevrolat, 1836: 383**

Originally included available species: none.

***Mycotretus* Chevrolat, 1836: 428**

Originally included available species: *Erotylus affinis* Duponchel, 1825 (as “*Affinis. Dej*.”); *Erotylus decoratus* Duponchel, 1825; *Erotylus duodecimguttatus* Duponchel, 1825 (as “Duodecimguttatus. *Dej*.”); *Erotylus hieroglyphicus* Duponchel, 1825 (as “Hieroglyphicus. *Dej*.”); *Erotylus interruptus* Duponchel, 1825 (as “Interruptus. *Dej*.”); *Erotylus maculosus* Duponchel, 1825 (as “Maculosus. *Dej*.”); *Erotylus minutus* Duponchel, 1825 (as “Minutus. *Dej*.”); *Erotylus modestus* Olivier, 1807; *Erotylus ornatus* Duponchel, 1825 (as “Ornatus. *Dej*.”); *Erotylus puncticollis* Duponchel, 1825 (as “Puncticollis. *Dej*.”); *Erotylus quadripunctatus* Olivier, 1791; *Erotylus scriptus* Olivier, 1807; *Erotylus tigrinus* Olivier, 1791; *Erotylus variabilis* Duponchel, 1825 (as “Variabilis. *Dej*.”).

Type species: *Erotylus ornatus* Duponchel, 1825 by subsequent designation (Alvarenga 1965: 87).

Current status: name suppressed in Erotylidae.

Comments. This name was suppressed for the purposes of the Principles of Priority and Homonymy in Opinion 1824 (ICZN 1996a).

***Myocera* Dejean, 1836a: 382**

Originally included available species: none.

***Myochrous* Chevrolat, 1836: 414**

Originally included available species: none.

***Myocoryna* Dejean, 1836a: 404**

Originally included available species: none.

***Nerissus* Dejean, 1836a: 414**

Originally included available species: none.

***Noda* Chevrolat, 1836: 410**

Originally included available species: *Colaspis humeralis* Latreille, 1813; *Chrysomela luteicornis* Fabricius, 1792 (as “Luteicornis. *Sch. Fabr?*”); *Colaspis tristis* Olivier, 1808.

Type species: *Colaspis tristis* Olivier, 1808 by subsequent designation (Monrós and Bechyné 1956: 1124).

Current status: junior homonym of *Noda* Schellenberg, 1803 [Diptera]; senior objective synonym of *Brachypnoea* Gistel, 1850 in Chrysomelidae (*fide* Riley et al. 2003: 141).

***Notosacantha* Chevrolat, 1836: 367**

Originally included available species: *Cassida echinata* Fabricius, 1801.

Type species: *Cassida echinata* Fabricius, 1801 by monotypy.

Current status: valid genus in Chrysomelidae (*fide* Borowiec and Sekerka 2010: 389).

***Notozona* Chevrolat, 1836: 394**

Originally included available species: *Altica bifasciata* Olivier, 1789.

Type species: *Altica bifasciata* Olivier, 1789 by monotypy.

Current status: valid genus in Chrysomelidae (*fide* Seeno and Wilcox 1982: 128).

***Ochralea* Chevrolat, 1836: 375**

Originally included available species: *Adorium flavum* Olivier, 1807.

Type species: *Adorium flavum* Olivier, 1807 by monotypy.

Current status: junior subjective synonym of *Oides* Weber, 1801 in Chrysomelidae (*fide* Beenen 2010: 491).

***Octotoma* Dejean, 1836a: 366**

Originally included available species: *Hispa plicatula* Fabricius, 1801.

Type species: *Hispa plicatula* Fabricius, 1801 by monotypy.

Current status: valid genus in Chrysomelidae (*fide* Riley et al. 2003: 28).

***Odontionopa* Chevrolat, 1836: 408**

Originally included available species: *Colaspis dentipes* Wiedemann, 1821; *Colaspis sericea* Gyllenhal, 1808.

Type species: *Colaspis sericea* Gyllenhal, 1808 by monotypy.

Current status: valid genus in Chrysomelidae (*fide* Seeno and Wilcox 1982: 54).

Comments. The name d*entipes* is listed in synonymy with *sericea* in Dejean’s catalogue; therefore the type species of *Odontionopa* is *sericea* by monotypy (ICZN 1999: Article 68.3).

***Odontoderes* Chevrolat, 1836: 420**

Originally included available species: none.

***Odontota* Chevrolat, 1836: 364**

Originally included available species: *Hispa bicolor* Olivier, 1792; *Hispa dentata* Fabricius, 1787; *Hispa humeralis* Fabricius, 1801; *Hispa nigrita* Olivier, 1808; *Hispa notata* Olivier, 1808; *Hispa ruficollis* Fabricius, 1801; *Hispa sanguinicollis* Linnaeus, 1771 (as “Sanguinicollis. *Fabr*.”); *Hispa scapularis* Olivier, 1808; *Hispa scutellaris* Olivier, 1808.

Type species: *Hispa humeralis* Fabricius, 1801 by subsequent designation (Monrós and Bechyné 1956: 1135).

Current status: valid genus in Chrysomelidae (*fide* Seeno and Wilcox 1982: 160).

***Oedipodes* Dejean, 1836a: 384** (as “Oedipodes. *Illiger*.”)

Originally included available species: none.

***Oligocera* Chevrolat, 1836: 382**

Originally included available species: none.

***Oligocorynus* Chevrolat, 1836: 426**

Originally included available species: *Erotylus discoideus* Olivier, 1807.

Type species: *Erotylus discoideus* Olivier, 1807 (= *Erotylus cinctus* Herbst, 1799) by monotypy.

Current status: valid genus in Erotylidae (*fide* Alvarenga 1965: 87).

Comments. As mentioned by Alvarenga (1965: 87), *Oligocorynus* Chevrolat, 1836 is a senior synonym of *Alloiotelus* Hope, 1841.

***Omaspides* Chevrolat, 1836: 371**

Originally included available species: *Cassida clatrata* Linnaeus, 1758 (as “*Clathrata. Olivier*.”); *Cassida transversa* Fabricius, 1798; *Cassida trifasciata* Fabricius, 1787.

Type species: *Cassida transversa* Fabricius, 1798 (= *Cassida clatrata* Linnaeus, 1758) by subsequent designation (Hope 1840: 158).

Current status: valid genus in Chrysomelidae (*fide* Borowiec 1999: 120).

***Omophoita* Chevrolat, 1836: 386**

Originally included available species: *Galleruca abbreviata* Fabricius, 1798; *Chrysomela aequinoctialis* Linnaeus, 1758 (as “Æquinoctialis. *Fabr*.”); *Chrysomela albicollis* Fabricius, 1787; *Galleruca cyanipennis* Fabricius, 1798; *Haltica episcopalis* Illiger, 1807; *Altica fulgida* Olivier, 1808; *Haltica octoguttata* Gröndal, 1808 (as “*Octoguttata. Schönherr*.”); *Haltica personata* Illiger, 1807; *Galleruca quadrinotata* Fabricius, 1798; *Haltica sesquilunata* Klug, 1829; *Haltica sexguttata* Illiger, 1807.

Type species: *Chrysomela aequinoctialis* Linnaeus, 1758 by subsequent designation (Chevrolat 1845: 6).

Current status: valid genus in Chrysomelidae (*fide* Seeno and Wilcox 1982: 140).

Comments. The type species of *Omophoita* designated by Monrós and Bechyné (1956: 1134), *Chrysomela equestris* Fabricius, 1787, is a *species inquirendum* in Dejean’s catalogue and so is not an originally included species.

***Omoteina* Chevrolat, 1836: 374**

Originally included available species: *Cassida humeralis* Olivier, 1808.

Type species: *Cassida humeralis* Olivier, 1808 by monotypy.

Current status: valid genus in Chrysomelidae (*fide* Borowiec 1999: 166).

***Omototus* Chevrolat, 1836: 383**

Originally included available species: none.

***Onchocephala* Chevrolat, 1836: 366**

Originally included available species: none.

***Ootheca* Dejean, 1836a: 378**

Originally included available species: *Crioceris mutabilis* Sahlberg, 1823 (as “Mutabilis. *Schönherr*.”).

Type species: *Crioceris mutabilis* Sahlberg, 1823 by monotypy.

Current status: valid genus in Chrysomelidae (*fide* Seeno and Wilcox 1982: 111).

***Oreina* Chevrolat, 1836: 402**

Originally included available species: *Chrysomela basilea* Gebler, 1823; *Chrysomela cacaliae* Schrank, 1785; *Chrysomela gloriosa* Fabricius, 1781; *Chrysomela sapphirus* Fabricius, 1801; *Chrysomela speciosa* Linnaeus, 1767 (as “Speciosa. *Fabr*.”); *Chrysomela sulcata* Gebler, 1823; *Chrysomela tristis* Fabricius, 1792.

Type species: *Chrysomela speciosa* Linnaeus, 1767 by subsequent designation (Chevrolat 1843: 656).

Current status: valid genus in Chrysomelidae (*fide* Kippenberg 2010: 422).

***Oxygona* Chevrolat, 1836: 389**

Originally included available species: none.

***Ozomena* Chevrolat, 1836: 379**

Originally included available species: none.

***Pachnephorus* Chevrolat, 1836: 414**

Originally included available species: *Cryptocephalus arenarius* Panzer, 1797 (as “Arenarius. *Fabr*.”); *Eumolpus sabulosus* Gebler, 1830; *Eumolpus villosus* Duftschmid, 1825 (as “Villosus. *Megerle*.”).

Type species: *Cryptocephalus arenarius* Panzer, 1797 (= *Cryptocephalus pilosus* Rossi, 1790) by subsequent designation (Monrós and Bechyné 1956: 1127).

Current status: valid genus in Chrysomelidae (*fide* Moseyko and Sprecher-Uebersax 2010: 627).

***Pachybrachis* Chevrolat, 1836: 420**

Originally included available species: *Cryptocephalus equestris* Olivier, 1808; *Cryptocephalus femoratus* Olivier, 1808; *Cryptocephalus glycyrhizae* Olivier, 1808; *Cryptocephalus hieroglyphicus* Laicharting, 1781 (as “Var. *Hieroglyphicus. Fabr*.”); *Cryptocephalus histrio* Fabricius, 1781; *Cryptocephalus luridus* Fabricius, 1798; *Cryptocephalus perlatus* Olivier, 1808; *Cryptocephalus pubescens* Fabricius, 1777; *Cryptocephalus quindecimguttatus* Fabricius, 1775; *Cryptocephalus rubi* Ménétriés, 1832; *Cryptocephalus tristis* Laicharting, 1781; *Cryptocephalus viduatus* Fabricius, 1801.

Type species: *Cryptocephalus hieroglyphicus* Laicharting, 1781 by subsequent designation (Jacoby 1908: 265).

Current status: valid genus in Chrysomelidae (*fide* Schöller et al. 2010: 611).

***Pachyonychus* Chevrolat, 1836: 384**

Originally included available species: none.

***Pales* Chevrolat, 1836: 408**

Originally included available species: *Colaspis ulema* Germar, 1813 (as “Ulema. *Megerle*.”).

Type species: *Colaspis ulema* Germar, 1813 by monotypy.

Current status: junior homonym of *Pales* Meigen, 1800 [Diptera]; senior objective synonym of *Eupales* Lefèvre, 1885 in Chrysomelidae (*fide* Moseyko and Sprecher-Uebersax 2010: 643, as “*Pales* Chevrolat, 1837”) for which the name *Floricola* Gistel, 1848 should be used as valid (see Löbl and Smetana 2011: 59).

***Pandona* Dejean, 1836a: 404**

Originally included available species: none.

***Pedema* Dejean, 1836a: 384**

Comments. This name was listed by Dejean as an invalid synonym of *Oedionychis* Latreille, 1829. The name has not been treated before 1961 as an available name and adopted as the name of a taxon or treated as a senior homonym and therefore *Pedema* Dejean is not available. *Pedema* was first used by Klug (1829: 9) but not made available.

***Periscapta* Chevrolat, 1836: 405**

Originally included available species: none.

***Phaedra* Dejean, 1836a: 414**

Originally included available species: none.

***Philocalis* Dejean, 1836a: 387**

Originally included available species: *Galleruca pulchra* Boisduval, 1835 (as “Pulchra. *d’Urville*.”)

Type species: *Galleruca pulchra* Boisduval, 1835 by monotypy.

Current status: valid genus in Chrysomelidae (*fide* Seeno and Wilcox 1982: 134, as “*Philocalis* Boisduval, 1835”).

***Phratora* Chevrolat, 1836: 405**

Originally included available species: *Chrysomela vitellinae* Linnaeus, 1758 (as “Vitellinae. *Fabr*.”); *Chrysomela vulgatissima* Linnaeus, 1758 (as “*Vulgatissima. Duftschmid*.”).

Type species: *Chrysomela vitellinae* Linnaeus, 1758 by monotypy.

Current status: valid genus in Chrysomelidae (*fide* Kippenberg 2010: 394).

Comments. The name *vulgatissima* is listed as an invalid synonym of *vitellinae* in Dejean’s catalogue; therefore the type species of *Phratora* is *vitellinae* by monotypy (ICZN 1999: Article 68.3). This species was also listed as type species of *Phratora* by Thomson (1859: 157) and several recent authors (e.g., Kimoto 1986: 126; Riley et al. 2003: 62) use that species as type species. However, some authors (e.g., Lopatin 1977: 167; Kippenberg 2010: 394) use *Chrysomela vulgatissima* Linnaeus, 1758 as type species of *Phratora* following Motschulsky (1860: 219). Both species are currently included in different subgenera (*fide* Kippenberg 2010: 394–395).

***Phygasia* Dejean, 1836a: 387**

Originally included available species: *Haltica helveola* Dalman, 1823 (as “Helvola. *Dalman*.”); *Altica unicolor* Olivier, 1808.

Type species: *Altica unicolor* Olivier, 1808 (= *Haltica silacea* Illiger, 1807) by subsequent designation (Chevrolat 1845: 6).

Current status: valid genus in Chrysomelidae (*fide* Döberl 2010: 544).

***Phyllecthris* Dejean, 1836a: 382**

Originally included available species: *Galeruca dorsalis* Olivier, 1808.

Type species: *Galeruca dorsalis* Olivier, 1808 by monotypy.

Current status: valid genus in Chrysomelidae (*fide* Riley et al. 2003: 81).

***Phyllobrotica* Chevrolat, 1836: 381**

Originally included available species: *Crioceris adusta* Creutzer, 1799 (as “Adusta. *Fabr*.”); *Galleruca discoidea* Fabricius, 1801; *Chrysomela quadrimaculata* Linnaeus, 1758 (as “Quadrimaculata. *Fabr*.”).

Type species: *Chrysomela quadrimaculata* Linnaeus, 1758 by subsequent designation (Thomson 1859: 156).

Current status: valid genus in Chrysomelidae (*fide* Beenen 2010: 486).

***Phyllotreta* Chevrolat, 1836: 391**

Originally included available species: *Haltica antennata* Koch, 1803 (as “Antennata. *Ent. Hefte*.”); *Haltica armoraciae* Koch, 1803 (as “Armoraciae. *Ent. Hefte*.”); *Altica atra* Fabricius, 1775 (as “Atra. *Ent. Hefte*.”); *Crioceris bipustulata* Fabricius, 1801; *Chrysomela brassicae* Fabricius, 1787; *Altica flexuosa* Illiger, 1794 (as “Flexuosa. *Ent. Hefte*.)”; *Haltica lepidii* Koch, 1803 (as “Lepidii. *Ent. Hefte*.”); *Chrysomela nemorum* Linnaeus, 1758 (as “Nemorum. *Fabr*.”); *Haltica obscurella* Illiger, 1807.

Type species: *Chrysomela nemorum* Linnaeus, 1758 by subsequent designation (Desmarest 1860: 351).

Current status: valid genus in Chrysomelidae (*fide* Döberl 2010: 545).

Comments. The type species usually listed for *Phyllotreta* Chevrolat is *Chrysomela brassicae* Fabricius, 1787 (= *Chrysomela exclamationis* Thunberg, 1784) (e.g., Döberl 2010: 545) apparently from Chevrolat (1845: 6). However, Chevrolat’s designation of *Chrysomela brassicae* Fabricius is made for “*Crepidodera*, *Phyllotreta* Ch. (*Orchestris* Kirby),” a group of genera. It cannot be considered a valid typification for *Phyllotreta*. *Chrysomela nemorum* Linnaeus is currently included in the genus *Phyllotreta* Chevrolat, so the change in type species has no taxonomic impact.

***Physicerus* Chevrolat, 1836: 420**

Originally included available species: *Cryptocephalus speciosus* Boisduval, 1835 (as “Speciosus. *d’Urville*.”).

Type species: *Cryptocephalus speciosus* Boisduval, 1835 by monotypy.

Current status: junior subjective synonym of *Cryptocephalus* Geoffroy, 1762 in Chrysomelidae (*fide* Seeno and Wilcox 1982: 39).

***Physimerus* Chevrolat, 1836: 383**

Originally included available species: none.

***Physocoryna* Chevrolat, 1836: 365**

Originally included available species: none.

***Physonota* Chevrolat, 1836: 374**

Originally included available species: *Cassida fuscata* Klug, 1829 (as “Fuscata. *Dej*.”).

Type species: *Cassida fuscata* Klug, 1829 by monotypy.

Current status: senior objective of *Anacassis* Spaeth, 1913 in Chrysomelidae (**new synonymy**).

Comments. The name *Physonota* is attributed to Boheman (1854: 190) in the literature (e.g., Borowiec 1999: 172; Riley et al. 2002: 646) with *Physonota alutacea* Boheman, 1854 as type species. To conserve the current concept of the genera *Anacassis* and *Physonota*, we believe the best avenue is to apply to the Commission to reject the name *Physonota* Chevrolat, 1836 for the Principles of Priority and Homonymy.

***Physonychis* Dejean, 1836a: 384**

Originally included available species: none.

***Physopalpa* Dejean, 1836a: 375**

Originally included available species: none.

***Plagiodera* Chevrolat, 1836: 404**

Originally included available species: *Chrysomela armoraciae* Linnaeus, 1758 (as “Armoriciae. *Fabr*.”); *Chrysomela circumcincta* Sahlberg, 1823; *Chrysomela encausta* Klug, 1829 (as “Encausta. *Dej*.”); *Chrysomela jucunda* Klug, 1829 (as “Jucunda. *Dej*.”); *Chrysomela nigriventris* Germar, 1824; *Chrysomela pallidiventris* Germar, 1824; *Chrysomela rufescens* Gyllenhal, 1808 (as “Rufescens. *Gröndal*.”); *Chrysomela thoracica* Fabricius, 1801; *Chrysomela transversa* Olivier, 1807.

Type species: see comments.

Current status: valid genus in Chrysomelidae (*fide* Kippenberg 2010: 392).

Comments. The type species of *Plagiodera* currently recognized is *Chrysomela armoraciae* Fabricius, 1775 (= *Chrysomela versicolora* Laicharting, 1781) (*fide* Kippenberg 2010: 392). However, Fabricius (1775: 103) did not describe a new species under this name since he referred to the species described by Linnaeus in 1758 under the name *Chrysomela armoraciae*. We have not found any indication that Fabricius misidentified Linnaeus’ species. In fact Fabricius’ description of the species is very short and similar to that of Linnaeus. We also found no indication that Dejean misidentified *Chrysomela armoraciae*. Therefore the type species of *Plagiodera* is *Chrysomela armoraciae* Linnaeus, 1758 by subsequent designation (Thomson 1859: 158). This species is currently included in the genus *Phaedon* Latreille, 1829. To promote stability, we believe that an application to the Commission to designate *Chrysomela versicolora* Laicharting, 1781 as the type species of *Plagiodera* is the best avenue.

***Planagetes* Chevrolat, 1836: 404**

Originally included available species: none.

***Platycorynus* Chevrolat, 1836: 413**

Originally included available species: *Eumolpus bifasciatus* Olivier, 1808; *Eumolpus chrysis* Olivier, 1808; *Eumolpus compressicornis* Fabricius, 1801; *Chrysomela cyanea* Fabricius, 1792; *Eumolpus groendalii* Swartz, 1808; *Eumolpus senegalensis* Olivier, 1808.

Type species: *Eumolpus compressicornis* Fabricius, 1801 by subsequent designation (Chapuis 1874: 339).

Current status: valid genus in Chrysomelidae (*fide* Moseyko and Sprecher-Uebersax 2010: 633).

***Plectroscelis* Chevrolat, 1836: 393**

Originally included available species: *Galeruca aridella* Paykull, 1799; *Haltica aridula* Gyllenhal, 1827; *Haltica dentipes* Koch, 1803 (as “Dentipes. *Ent. Hefte*.”); *Haltica mannerheimii* Gyllenhal, 1827; *Haltica sahlbergii* Gyllenhal, 1827.

Type species: *Haltica aridula* Gyllenhal, 1827 by subsequent designation (Monrós and Bechyné 1956: 1134).

Current status: junior subjective synonym of *Chaetocnema* Stephens, 1831 in Chrysomelidae (*fide* Döberl 2010: 505).

Comments. Konstantinov et al. (2011: 17) listed *Haltica dentipes* sensu Olivier, 1808 (= *Altica chlorophana* Duftschmid, 1825) as type species of *Plectroscelis* Chevrolat by subsequent designation in Chevrolat (1845: 6). However, Chevrolat (1845: 6) listed the type species as “*Alt. dentipes* Ol.” and it is evident that he considered *Altica dentipes* Olivier and *Altica dentipes* Koch as two different species (see Chevrolat 1847b: 267). Therefore his type species designation of *Altica dentipes* sensu Olivier is invalid since Olivier’s taxon is not originally included in Dejean’s catalogue.

***Pleuraulaca* Chevrolat, 1836: 409**

Originally included available species: *Eumolpus bicolor* Olivier, 1808; *Colaspis dives* Germar, 1824; *Chrysomela glabrata* Fabricius, 1792; *Colaspis limbata* Olivier, 1808.

Type species: *Colaspis dives* Germar, 1824 by subsequent designation (Monrós and Bechyné 1956: 1125).

Current status: senior subjective synonym of *Iphimeis* Baly, 1864 in Chrysomelidae (*fide* Seeno and Wilcox 1982: 57).

Comments. Monrós and Bechyné (1956) designated two type species for this genus under two distinct entries, one (*Colaspis dives* Germar) under *Paraulaca* Chevrolat (p. 1125), evidently an error for *Pleuraulaca*, and the other one (*Colaspis limbata* Olivier) under *Pleuraulaca* Chevrolat (p. 1128). The first type species designation is accepted here as the valid one because *Colaspis dives* Germar is currently included in the genus *Iphimeis* Baly while the second type species (*Colaspis limbata* Olivier) is included in the genus *Eriphylina* Lefèvre, 1891 (e.g., Bechyné 1953: 168, 176).

*Pleuraulaca* Chevrolat, 1836 has precedence over *Iphimeis* Baly, 1864 which is currently used as valid (e.g., Bechyné 1953: 168). Reversal of Precedence (ICZN 1999: Article 23.9) or an application to the Commission is necessary to conserve usage of the name *Iphimeis* Baly, 1864.

***Pleurophora* Chevrolat, 1836: 361**

Originally included available species: none

***Plusiopeplis* Dejean, 1836a: 414**

Originally included available species: none.

***Podagrica* Chevrolat, 1836: 394**

Originally included available species: *Altica aeneipennis* Latreille, 1813; *Haltica dilecta* Dalman, 1823; *Crioceris fulvipes* Fabricius, 1801; *Chrysomela fuscicornis* Linnaeus, 1767; *Altica fuscipes* Fabricius, 1775; *Haltica malvae* Illiger, 1807.

Type species: *Altica fuscipes* Fabricius, 1775 by subsequent designation (Maulik 1926: 273).

Current status: valid genus in Chrysomelidae (*fide* Döberl 2010: 550).

Comments. The designation of *Haltica malvae* Illiger, 1807 as type-species by Monrós and Bechyné (1956: 1133) is invalid because of the prior valid typification by Maulik (1926: 273).

***Polychalca* Chevrolat, 1836: 368**

Originally included available species: *Cassida antiqua* Klug, 1829 (as “Antiqua. *Dej*.”); *Cassida metallica* Klug, 1829 (as “Metallica. *Dej*.”); *Cassida platynota* Germar, 1824; *Cassida variolosa* Weber, 1801 (as “Variolosa. *Fabr*.”).

Type species: *Cassida variolosa* Weber, 1801 (= *Cassida punctatissima* Wolf, 1818) by subsequent designation (Duponchel and Chevrolat 1842b: 211).

Current status: valid genus in Chrysomelidae (*fide* Borowiec 1999: 57).

***Polyclada* Chevrolat, 1836: 375**

Originally included available species: *Clytra pectinicornis* Olivier, 1791.

Type species: *Clytra pectinicornis* Olivier, 1791 by monotypy.

Current status: valid genus in Chrysomelidae (*fide* Döberl 2010: 551).

***Polygramma* Chevrolat, 1836: 397**

Originally included available species: *Chrysomela juncta* Germar, 1824.

Type species: *Chrysomela juncta* Germar, 1824 by monotypy.

Current status: name suppressed in Chrysomelidae.

Comments. This name was suppressed in Opinion 1290 by the International Commission on Zoological Nomenclature for the purposes of the Principle of Priority and placed on the Official Index of Rejected and Invalid Generic Names in Zoology as “*Polygramma* Chevrolat, 1837” (ICZN 1985a).

***Prionocheilus* Chevrolat, 1836: 427**

Originally included available species: none.

***Prionodera* Chevrolat, 1836: 407**

Originally included available species: *Colaspis bicolor* Olivier, 1808.

Type species: *Colaspis bicolor* Olivier, 1808 by monotypy.

Current status: valid genus in Chrysomelidae (*fide* Seeno and Wilcox 1982: 58).

***Promecosoma* Chevrolat, 1836: 409**

Originally included available species: none.

***Promecotheca* Dejean, 1836a: 363**

Originally included available species: none.

***Proseicela* Chevrolat, 1836: 398**

Originally included available species: *Chrysomela vittata* Fabricius, 1781.

Type species: *Chrysomela vittata* Fabricius, 1781 by monotypy.

Current status: valid genus in Chrysomelidae (*fide* Seeno and Wilcox 1982: 79).

***Protophysus* Chevrolat, 1836: 422**

Originally included available species: *Chryptocephalus haemorrhoidalis* Olivier, 1791 (as “♀. *Haemorrhoidalis. Fabr*.”); *Cryptocephalus lobatus* Fabricius, 1792.

Type species: *Cryptocephalus lobatus* Fabricius, 1792 (= *Cryptocephalus schaefferi* Schrank, 1789) by monotypy.

Current status: valid subgenus of *Cryptocephalus* Geoffroy, 1762 in Chrysomelidae (*fide* Lopatin et al. 2010: 606).

Comments. The name *haemorrhoidalis* is listed in synonymy with *lobatus* in Dejean’s catalogue; therefore the type species of *Protophysus* is *lobatus* by monotypy (ICZN 1999: Article 68.3).

***Prototrigona* Chevrolat, 1836: 387**

Originally included available species: none.

***Ptena* Chevrolat, 1836: 386**

Originally included available species: *Altica cruciata* Olivier, 1808; *Chrysomela nobilitata* Fabricius, 1787; *Haltica ornata* Illiger, 1807; *Chrysomela quadrifasciata* Fabricius, 1787.

Type species: *Chrysomela nobilitata* Fabricius, 1787 by subsequent designation (Chevrolat 1845: 6).

Current status: invalid synonym of *Omophoita* Chevrolat, 1836 in Chrysomelidae (*fide* Seeno and Wilcox 1982: 140).

***Pyxis* Dejean, 1836a: 404**

Originally included available species: none.

***Raphidopalpa* Chevrolat, 1836: 378**

Originally included available species: *Crioceris abdominalis* Fabricius, 1781; *Chrysomela coffeae* Hornstedt, 1788 (as “*Coffeae. Herbst*.”); *Gallerica ioptera* Wiedemann, 1823 (as “*Eoptera. Wiedemann*.”); *Galleruca oblonga* Gyllenhal, 1808 (as “Oblonga. *Schönherr*.”); *Galeruca similis* Olivier, 1808.

Type species: *Crioceris abdominalis* Fabricius, 1781 by subsequent designation (Chevrolat 1845: 6).

Current status: invalid synonym of *Aulacophora* Chevrolat, 1836 in Chrysomelidae (*fide* Beenen 2010: 465).

***Rhinotmetus* Chevrolat, 1836: 383**

Originally included available species: none.

***Rhombopalpa* Chevrolat, 1836: 375**

Originally included available species: *Adorium decempunctatum* Billberg, 1808 (as “Decempunctata. *Schönherr*.”).

Type species: *Adorium decempunctatum* Billberg, 1808 by monotypy.

Current status: junior subjective synonym of *Oides* Weber, 1801 in Chrysomelidae (*fide* Beenen 2010: 491).

***Romalocera* Dejean, 1836a: 389**

Originally included available species: none.

***Rumina* Dejean, 1836a: 414**

Originally included available species: none.

***Saccomorphus* Chevrolat, 1836: 426**

Originally included available species: *Erotylus abdominalis* Fabricius, 1792; *Erotylus bimaculatus* Duponchel, 1825 (as “Bimaculatus. *Dej*.”); *Erotylus clavicornis* Olivier, 1791; *Erotylus limbatus* Olivier, 1791 (as “Limbatus. *Fabr*.”); *Erotylus quadrisignatus* Duponchel, 1825.

Type species: *Erotylus limbatus* Olivier, 1791 by subsequent designation (Alvarenga 1965: 89).

Current status: valid genus in Erotylidae (*fide* Alvarenga 1965: 89).

Comments. As mentioned by Alvarenga (1965: 89), *Saccomorphus* Chevrolat, 1836 is a senior synonym of *Morphoides* Hope, 1841.

***Sceloenopla* Chevrolat, 1836: 364**

Originally included available species: *Hispa spinipes* Fabricius, 1794.

Type species: *Hispa spinipes* Fabricius, 1794 (= *Hispa maculata* Olivier, 1792) by monotypy.

Current status: valid genus in Chrysomelidae (*fide* Seeno and Wilcox 1982: 159).

***Schematiza* Chevrolat, 1836: 377**

Originally included available species: *Galleruca flavofasciata* Klug, 1829 (as “Flavofasciata. *Dej*.”); *Lycus laevigatus* Fabricius, 1801.

Type species: *Lycus laevigatus* Fabricius, 1801 by subsequent designation (Barber 1947a: 242).

Current status: valid genus in Chrysomelidae (*fide* Seeno and Wilcox 1982: 101).

***Smaragdina* Chevrolat, 1836: 420**

Originally included available species: *Cryptocephalus concolor* Fabricius, 1792; *Clytra dorsalis* Olivier, 1808; *Clythra menetriesii* Faldermann, 1832.

Type species: *Clythra menetriesii* Faldermann, 1832 (= *Clytra unipunctata* Olivier, 1808) by subsequent designation (Monrós 1953: 46).

Current status: valid genus in Chrysomelidae (*fide* Regalin and Medvedev 2010: 575).

***Spartophila* Chevrolat, 1836: 403**

Originally included available species: *Chrysomela aegrota* Fabricius, 1798; *Chrysomela caraganae* Gebler, 1823; *Chrysomela flavicans* Fabricius, 1787 (as “Var. *Flavicans. Olivier*.”); *Chrysomela litura* Fabricius, 1775; *Chrysomela sexnotata* Fabricius, 1798; *Chrysomela sexpunctata* Fabricius, 1787; *Chrysomela spartii* Olivier, 1807; *Chrysomela variabilis* Olivier, 1790.

Type species: *Chrysomela litura* Fabricius, 1775 (= *Chrysomela olivacea* Forster, 1771) by subsequent designation (Chevrolat 1843: 656).

Current status: valid subgenus of *Gonioctena* Chevrolat, 1836 in Chrysomelidae (*fide* Kippenberg 2010: 436, as “*Spartophila* Stephens, 1834”).

Comments. The name *Spartophila* is usually credited to Stephens (1834: 340) from the *Illustrations of British Entomology* (e.g., Weise 1916: 182; Seeno and Wilcox 1982: 85; Kippenberg 2010: 436). However, we were unable to find this name in Stephen’s *Illustrations of British Entomology*. The first typification for *Spartophila* is that of Hope (1840: 163) who selected *Chrysomela spartii* Olivier, 1807 (= *Chrysomela variabilis* Olivier, 1790). This species is currently included in the subgenus *Spartoxena* Motschulsky, 1860 (*fide* Kippenberg 2010: 437) and its acceptance as type species of *Spartophila* will bring nomenclatural changes. A request to the Commission is needed to suppress the typification of Hope (1840).

***Sphaerometopa* Chevrolat, 1836: 387**

Originally included available species: *Haltica acroleuca* Wiedemann, 1819.

Type species: *Haltica acroleuca* Wiedemann, 1819 by monotypy.

Current status: valid genus in Chrysomelidae (*fide* Seeno and Wilcox 1982: 141).

***Sphaeronychus* Dejean, 1836a: 383** (as “Sphraeronychus. *Dejean*.”)

Originally included available species: *Altica melanura* Olivier, 1808.

Type species: *Altica melanura* Olivier, 1808 by monotypy.

Current status: valid genus in Chrysomelidae (*fide* Seeno and Wilcox 1982: 141).

Comments. The spelling *Sphaeronychus* is an incorrect subsequent spelling of *Sphraeronychus* in prevailing usage and attributed to the publication of the original spelling; therefore *Sphaeronychus* is deemed to be the correct original spelling (ICZN 1999: Article 33.3.1).

***Sphaeropalpus* Chevrolat, 1836: 367**

Originally included available species: none.

***Sphaeropis* Chevrolat, 1836: 410**

Originally included available species: none.

***Sphaeroplacis* Chevrolat, 1836: 409**

Originally included available species: none.

***Sphaeropomis* Dejean, 1836a: 393**

Originally included available species: none.

***Spintherophyta* Dejean, 1836a: 410**

Originally included available species: *Colaspis nana* Klug, 1829 (as “Nana. *Dej*.”); *Colaspis semiaurata* Klug, 1829.

Type species: *Colaspis semiaurata* Klug, 1829 by subsequent designation (Monrós and Bechyné 1956: 1124).

Current status: valid genus in Chrysomelidae (*fide* Riley et al. 2003: 148).

***Stenodiloba* Chevrolat, 1836: 407**

Originally included available species: none.

***Stilodes* Chevrolat, 1836: 403**

Originally included available species: *Chrysomela humeralis* Gory, 1833.

Type species: *Chrysomela humeralis* Gory, 1833 by monotypy.

Current status: valid genus in Chrysomelidae (*fide* Seeno and Wilcox 1982: 79).

***Strabala* Chevrolat, 1836: 389**

Originally included available species: none.

Comments. This name is considered valid with *Altica ferruginea* Olivier, 1808 as type species (Riley et al. 2003: 123). However this species and *Altica scutellaris* Olivier, 1808, the second available species listed by Chevrolat (1836: 389) under the genus *Strabala*, have question marks after the species names and so are considered *species inquiranda* (see “Methods” section). These species are deemed not to be originally included (ICZN 1999: Article 67.2.5). The name *Strabala* was subsequently used by Chevrolat (1848: 52) who listed this time the two Olivier’s species without question marks. Therefore, this name should be credited to Chevrolat (1848) as indicated by Blake (1953: 121–122).

***Strichosa* Chevrolat, 1836: 397**

Originally included available species: none.

***Strigophorus* Chevrolat, 1836: 422**

Originally included available species: none.

***Strongylosomus* Chevrolat, 1836: 427**

Originally included available species: *Erotylus brevicornis* Duponchel, 1825 (as “Brevicornis. *Dej*.”); *Erotylus coccinelloides* Duponchel, 1825 (as “Coccinelloides. *Dej*.”); *Erotylus nigripes* Duponchel, 1825 (as “Nigripes. *Dej*.”); *Erotylus unicolor* Olivier, 1807 (as “Unicolor. *Latreille. Olivier?*”).

Type species: *Erotylus unicolor* Olivier, 1807 by subsequent designation (Crotch 1876: 487).

Current status: valid genus in Erotylidae (*fide* Alvarenga 1965: 90).

Comments. As mentioned by Alvarenga (1965: 90), *Strongylosomus* Chevrolat, 1836 is a senior synonym of *Coccimorphus* Hope, 1841.

***Strongylotarsa* Chevrolat, 1836: 410**

Originally included available species: none.

***Syneta* Dejean, 1835: 359 and 1836a: 361** (as “Syneta. Eschscholtz”)

Originally included available species: *Crioceris betulae* Fabricius, 1792.

Type species: *Crioceris betulae* Fabricius, 1792 by monotypy.

Current status: valid genus in Chrysomelidae (*fide* Silfverberg 2010: 643, as “*Syneta* Chevrolat, 1837”).

***Systena* Chevrolat, 1836: 390**

Originally included available species: *Galleruca elongata* Fabricius, 1798 (as “*Elongata. Olivier*.”); *Galleruca frontalis* Fabricius, 1801; *Haltica striolata* Schönherr, 1808.

Type species: *Galleruca frontalis* Fabricius, 1801 by subsequent designation (Monrós and Bechyné 1956: 1133).

Current status: valid genus in Chrysomelidae (*fide* Riley et al. 2003: 102).

***Tachypetes* Chevrolat, 1836: 419**

Originally included available species: none.

***Teinodactyla* Chevrolat, 1836: 392**

Comments. This name is treated as an unnecessary replacement name for *Longitarsus* Latreille, 1829 [Chrysomelidae].

***Tetraphala* Chevrolat, 1836: 430**

Originally included available species: none.

***Thalassia* Chevrolat, 1836: 430**

Originally included available species: none.

***Thyra* Dejean, 1836a: 410**

Originally included available species: none.

***Thyreomorpha* Dejean, 1836a: 367**

Originally included available species: none.

***Thysbe* Dejean, 1836a: 411**

Originally included available species: none.

***Trichostola* Chevrolat, 1836: 411**

Originally included available species: none.

***Typocephalus* Chevrolat, 1836: 427**

Originally included available species: none.

Comments. The type species cited by Alvarenga (1965: 90) for *Typocephalus* Chevrolat, *Erotylus dimidiatus* Olivier, 1792, is a *species inquirandum* in Dejean’s catalogue and therefore is not an originally included species (ICZN 1999: Article 67.2.5). The name *Typocephalus* should be credited to Hope (1841: 113) who first made the name available by describing the genus and selecting *Erotylus dimidiatus* Olivier, 1792 as type species.

***Typophorus* Chevrolat, 1836: 412**

Originally included available species: *Eumolpus cyanellus* Boisduval, 1835 (as “Cyanellus. *d’Urville*.”); *Eumolpus nigritus* Fabricius, 1801; *Eumolpus spinipes* Latreille, 1813.

Type species: *Eumolpus nigritus* Fabricius, 1801 by subsequent designation (Monrós and Bechyné 1956: 1127).

Current status: valid genus in Chrysomelidae (*fide* Riley et al. 2003: 141).

***Uroplata* Chevrolat, 1836: 365**

Originally included available species: *Hispa hastata* Fabricius, 1801; *Hispa inaequalis* Weber, 1801; *Hispa mucronata* Olivier, 1808; *Hispa quadrata* Fabricius, 1801; *Hispa truncata* Fabricius, 1801 (as “Truncata. *Olivier*”).

Type species: *Hispa mucronata* Olivier, 1808 by subsequent designation (Monrós and Bechyné 1956: 1135).

Current status: valid genus in Chrysomelidae (*fide* Seeno and Wilcox 1982: 161).

Comments. *Uroplata* Chevrolat was placed on the official List of Generic Names in Zoology in Opinion 1359 (ICZN 1985b) with *Hispa mucronata* Olivier as the type species designated by White (1981: 714).

***Zygogramma* Chevrolat, 1836: 398**

Originally included available species: *Chrysomela decora* Klug, 1829 (as “Decora. *Dej*.”); *Chrysomela deleta* Klug, 1829 (as “Deleta. *Dej*.”); *Chrysomela elegans* Olivier, 1808; *Chrysomela pulcra* Fabricius, 1792 (as “Pulchra. *Fabr*.”); *Chrysomela quadrivittata* Latreille, 1813; *Chrysomela tetragramma* Klug, 1829 (as “Tetragramma. *Dej*.”).

Type species: *Chrysomela pulcra* Fabricius, 1792 (= *Chrysomela suturalis* Fabricius, 1775) by subsequent designation (Motschulsky 1860: 181).

Current status: valid genus in Chrysomelidae (*fide* Kippenberg 2010: 426).

### Trimères

***Ancylopus* Chevrolat, 1836: 439** (as “Agcylopus. *Chevrolat*.”)

Originally included available species: *Endomychus melanocephalus* Olivier, 1808.

Type species: *Endomychus melanocephalus* Olivier, 1808 by monotypy.

Current status: valid genus in Endomychidae (*fide* Shockley et al. 2009: 31).

Comments. The spelling *Ancylopus* is an incorrect subsequent spelling of *Agcylopus* in prevailing usage, attributed to the publication of the original spelling, and so deemed to be the correct original spelling (ICZN 1999: Article 33.3.1).

***Anisosticta* Chevrolat, 1836: 432**

Originally included available species: *Coccinella bissexpunctata* Latreille, 1813; *Coccinella decemmaculata* Fabricius, 1781; *Coccinella m-nigrum* Fabricius, 1792; *Coccinella novemdecimpunctata* Linnaeus, 1758 (as “Novemdecimpunctata. *Fabr*.”); *Coccinella 18.pustulata* Klug, 1829 (as “Octodecimpustulata. *Dej*.”); *Coccinella quadrifasciata* Thunberg, 1808.

Type species: *Coccinella novemdecimpunctata* Linnaeus, 1758 by subsequent designation (Crotch 1874: 93).

Current status: valid genus in Coccinellidae (*fide* Kovář 2007: 599).

***Aploscelis* Chevrolat, 1836: 439**

Originally included available species: *Eumorphus atratus* Klug, 1833.

Type species: *Eumorphus atratus* Klug, 1833 by monotypy.

Current status: valid genus in Endomychidae (*fide* Shockley et al. 2009: 51, as “*Haploscelis* Blanchard, 1845”).

Comments. This generic name is usually credited to Blanchard (1845: 312) under the spelling *Haploscelis* (e.g., Strohecker 1953: 81; Tomaszewska 2005: 43; Shockley et al. 2009: 51). This spelling is in prevailing usage but not attributed to the original author (see ICZN 1999: Article 33.3.1). Therefore the original spelling used by Chevrolat must be retained.

***Brachiacantha* Chevrolat, 1836: 432**

Originally included available species: *Coccinella bisquinquepustulata* Fabricius, 1801; *Coccinella bistripustulata* Fabricius, 1801; *Coccinella dentipes* Fabricius, 1801; *Coccinella ursina* Fabricius, 1787.

Type species: *Coccinella dentipes* Fabricius, 1801 by subsequent designation (Crotch 1873b: 377).

Current status: valid genus in Coccinellidae (*fide* Gordon 1985: 556).

***Cheilomenes* Chevrolat, 1836: 435**

Originally included available species: *Coccinella interrupta* Fabricius, 1792; *Coccinella lunata* Fabricius, 1775; *Coccinella quadriplagiata* Swartz, 1808; *Coccinella sexmaculata* Fabricius, 1781; *Coccinella sulphurea* Olivier, 1791; *Coccinella vulpina* Fabricius, 1798.

Type species: *Coccinella lunata* Fabricius, 1775 by subsequent designation (Crotch 1874: 179).

Current status: valid genus in Coccinellidae (*fide* Kovář 2007: 610).

Comments. This genus-group name is sometimes reported under the spelling *Chilomenes*. However, *Cheilomenes* seems in prevailing usage as seen by a search through the Zoological Records for the last 20 years.

***Chnoodes* Chevrolat, 1836: 437**

Originally included available species: none.

***Chnootriba* Chevrolat, 1836: 436**

Originally included available species: *Coccinella erythromela* Wiedemann, 1821; *Coccinella similis* Thunberg, 1781 (as “Similis. *Herbst*.”).

Type species: *Coccinella similis* Thunberg, 1781 by monotypy.

Current status: valid genus in Coccinellidae (*fide* Kovář 2007: 626).

Comments. The name *erythromela* is listed in synonymy with *similis* in Dejean’s catalogue; therefore the type species of *Chnootriba* is *similis* by monotypy (ICZN 1999: Article 68.3).

***Corynomalus* Chevrolat, 1836: 439**

Originally included available species: *Aegithus cinctus* Fabricius, 1801 (as “Cinctus. *Olivier*.”); *Eumorphus cruciger* Latreille, 1809; *Eumorphus limbatus* Olivier, 1808.

Type species: *Eumorphus limbatus* Olivier, 1808 (= *Erotylus marginatus* Fabricius, 1798) by **present designation**.

Current status: valid genus in Endomychidae (*fide* Shockley et al. 2009: 38).

Comments. The typification of Shockley et al. (2009: 38) for *Corynomalus* Chevrolat, *Corynomalus tarsatus* Erichson, 1847, is invalid since the species is not an originally included available species.

***Cynegetis* Chevrolat, 1836: 437**

Originally included available species: *Coccinella aptera* Paykull, 1798; *Coccinella globosa* Illiger, 1798; *Coccinella impunctata* Linnaeus, 1767; *Coccinella vigintiquatuorpunctata* Linnaeus, 1758 (as “*Vigintiquatuorpunctata. Fabr*.”).

Type species: *Coccinella impunctata* Linnaeus, 1767 by subsequent designation (Thomson 1859: 160).

Current status: valid genus in Coccinellidae (*fide* Kovář 2007: 625).

***Ephebus* Chevrolat, 1836: 439**

Originally included available species: none.

***Epilachna* Chevrolat, 1836: 436**

Originally included available species: *Coccinella argulata* Fabricius, 1798 (as “*Argulata. Olivier*.”); *Coccinella bifasciata* Fabricius, 1781; *Coccinella borealis* Fabricius, 1775; *Coccinella canina* Fabricius, 1781; *Coccinella capensis* Thunberg, 1781; *Coccinella chrysomelina* Fabricius, 1775; *Coccinella dispar* Fabricius, 1801; *Coccinella duodecimverrucata* Fabricius, 1801; *Coccinella elaterii* Rossi, 1794; *Coccinella flavicollis* Thunberg, 1781 (as “Flavicollis. *Olivier*.”); *Coccinella haemorrhoa* Boisduval, 1835 (as “Haemorrhoa. *d’Urville*.”); *Coccinella humeralis* Latreille, 1809; *Coccinella immaculicollis* Chevrolat, 1835; *Coccinella marginella* Fabricius, 1787; *Coccinella obscurocincta* Klug, 1829 (as “Obscurocincta. *Dej*.”); *Coccinella obsoleta* Olivier, 1808; *Coccinella paenulata* Germar, 1824; *Coccinella palliata* Schönherr, 1808; *Coccinella pavonia* Olivier, 1808; *Coccinella quadriplagiata* Latreille, 1809; *Coccinella signatipennis* Boisduval, 1835 (as “Signatipennis. *d’Urville*.”); *Coccinella tredecimnotata* Latreille, 1813; *Coccinella undecimmaculata* Fabricius, 1787; *Coccinella velutina* Olivier, 1808; *Coccinella vigintioctopunctata* Fabricius, 1775.

Type species: *Coccinella borealis* Fabricius, 1775 by subsequent designation (Hope 1840: 157).

Current status: valid genus in Coccinellidae (*fide* Kovář 2007: 626).

***Epipocus* Chevrolat, 1836: 439**

Originally included available species: *Lycoperdina lata* Guérin-Méneville, 1834 (as “*Latus. Chevrolat*.”); *Endomychus rufitarsis* Chevrolat, 1835; *Endomychus tibialis* Guérin-Méneville, 1834 (as “*Tibialis. Chevrolat*.”).

Type species: *Endomychus tibialis* Guérin-Méneville, 1834 by subsequent designation (Strohecker 1953: 66).

Current status: valid genus in Endomychidae (*fide* Shockley et al. 2009: 24).

***Epopterus* Chevrolat, 1836: 439**

Originally included available species: *Erotylus ocellatus* Olivier, 1791.

Type species: *Erotylus ocellatus* Olivier, 1791 by monotypy.

Current status: valid genus in Endomychidae (*fide* Shockley et al. 2009: 25).

***Exoplectra* Chevrolat, 1836: 437**

Originally included available species: *Coccinella coccinea* Fabricius, 1801; *Coccinella miniata* Germar, 1824.

Type species: *Coccinella coccinea* Fabricius, 1801 by subsequent designation (Korschefsky 1932: 227).

Current status: valid genus in Coccinellidae (*fide* Gordon 1985: 670).

***Hippodamia* Chevrolat, 1836: 432**

Originally included available species: *Coccinella abbreviata* Fabricius, 1787; *Coccinella amoena* Faldermann, 1835; *Coccinella arctica* Fabricius, 1794; *Coccinella connexa* Germar, 1824; *Coccinella glacialis* Fabricius, 1775; *Coccinella mutabilis* Scriba, 1791 (as “Mutabilis. *Illiger*.”); *Coccinella novempunctata* Linnaeus *sensu* Scopoli, 1763 (as “*Novempunctata. Schrank*.”); *Coccinella quinquemaculata* Fabricius, 1787; *Coccinella septemmaculata* Fabricius, 1777; *Coccinella septemnotata* Fabricius, 1792; *Coccinella sexdecimpustulata* Latreille, 1813; *Coccinella tredecimpunctata* Linnaeus, 1758 (as “Tredecimpunctata. *Fabr*.”); *Coccinella undecimpunctata* Schrank, 1781.

Type species: *Coccinella mutabilis* Scriba, 1791 (= *Coccinella variegata* Goeze, 1777) by subsequent designation (Chevrolat 1845: 623).

Current status: valid genus in Coccinellidae (*fide* Kovář 2007: 616).

***Hylaia* Chevrolat, 1836: 440**

Originally included available species: none.

Comments. This taxon is incorrectly credited to Chevrolat (1836: 440) by Tomaszewska (2007: 564) and Shockley et al. (2009: 52) with *Lycoperdina rubricollis* Germar, 1844 as type species. *Hylaia* was first made available by Germar (1844: pl. 18) who proposed the name in synonymy with *Lycoperdina* Latreille, 1807. The name is available from Germar (1844) because it was used as a valid name before 1961 (e.g., Guérin-Méneville 1857: 273) (ICZN 1999: Article 11.6.1).

***Hyperaspis* Chevrolat, 1836: 435**

Originally included available species: *Coccinella connectens* Thunberg, 1808; *Coccinella lateralis* Panzer, 1794 (as “Lateralis. *Fabr*.”); *Coccinella marginella* Fabricius, 1801; *Coccinella reppensis* Herbst, 1783 (as “*Reppensis. Paykull*.”) *Coccinella trilineata* Fabricius, 1787; *Coccinella stigma* Olivier, 1808.

Type species: *Coccinella reppensis* Herbst, 1783 by subsequent designation (Thomson 1859: 161).

Current status: valid genus in Coccinellidae (*fide* Kovář 2007: 577).

***Leiestes* Chevrolat, 1836: 440**

Originally included available species: *Cryptophagus seminiger* Gyllenhal, 1808.

Type species: *Cryptophagus seminiger* Gyllenhal, 1808 by monotypy.

Current status: valid genus in Endomychidae (*fide* Shockley et al. 2009: 88).

***Macaria* Dejean, 1836a: 434**

Originally included available species: none.

***Menoscelis* Dejean, 1836a: 435**

Originally included available species: none.

***Micraspis* Chevrolat, 1836: 435**

Originally included available species: *Coccinella cincta* Fabricius, 1798; *Coccinella duodecimpunctata* Linnaeus, 1767 (as “Duodecimpunctata. *Fabr*.”); *Coccinella limbata* Fabricius, 1801; *Coccinella striata* Fabricius, 1792; *Coccinella vittata* Olivier, 1808 (as “Vittata. *Fabr*.”).

Type species: *Coccinella striata* Fabricius, 1792 (= *Coccinella lineata* Thunberg, 1781) by subsequent designation (Hope 1840: 157).

Current status: valid genus in Coccinellidae (*fide* Kovář 2007: 619).

***Nundina* Dejean, 1836a: 438**

Comments. This name is treated as an unnecessary replacement name for *Rhyzobius* Stephens, 1829 [Coccinellidae].

***Olenus* Chevrolat, 1836: 439**

Originally included available species: none.

***Orestia* Chevrolat, 1836: 440**

Originally included available species: none.

***Pelinus* Dejean, 1836a: 439**

Originally included available species: none.

***Psyllobora* Chevrolat, 1836: 434**

Originally included available species: *Coccinella confluens* Fabricius, 1801; *Coccinella lineola* Fabricius, 1792.

Type species: *Coccinella lineola* Fabricius, 1792 (= *Psyllobora fabricii* Crotch, 1871) by subsequent designation (Timberlake 1943: 41).

Current status: valid genus in Coccinellidae (*fide* Kovář 2007: 599).

***Quirinus* Chevrolat, 1836: 439**

Originally included available species: none.

***Rhanis* Dejean, 1836a: 440**

Originally included available species: none.

***Synonycha* Chevrolat, 1836: 436**

Originally included available species: *Coccinella versicolor* Fabricius, 1792.

Type species: *Coccinella versicolor* Fabricius, 1792 (= *Coccinella grandis* Thunberg, 1781) by monotypy.

Current status: valid genus in Coccinellidae (*fide* Kovář 2007: 625).

## Figures and Tables

**Table:** 

**Names in Dejean’s catalogue [year and page in Dejean]**	**Current names with author and date^1^**
**Pentamères: Carabiques**	
*Abacetus* Dejean [1833: 32]	*Abacetus* Dejean, 1828
*Abaris* Dejean [1833: 37]	*Abaris* Dejean, 1831
*Abax* Bonelli [1833: 36]	*Abax* Bonelli, 1810
*Acanthoscelis* Latreille [1833: 15]	*Acanthoscelis* Latreille, 1810
*Acinopus* Ziegler [1833: 40]	*Acinopus* Dejean, 1821
*Acupalpus* Latreille [1833: 47]	*Acupalpus* Latreille, 1829
*Agonoderus* Dejean [1833: 41]	*Agonoderus* Dejean, 1829
*Agonum* Bonelli [1833: 30]	*Agonum* Bonelli, 1810
*Agra* Fabricius [1833: 7]	*Agra* Fabricius, 1801
*Amara* Bonelli [1833: 38]	*Amara* Bonelli, 1810
*Amblygnathus* Dejean [1833: 41]	*Amblygnathus* Dejean, 1829
*Anchomenus* Bonelli [1833: 29]	*Anchomenus* Bonelli, 1810
*Anisodactylus* Dejean [1833: 42]	*Anisodactylus* Dejean, 1829
*Antarctia* Dejean [1833: 40]	*Antarctia* Dejean, 1828
*Anthia* Weber [1833: 13]	*Anthia* Weber, 1801
*Apotomus* Hoffmansegg [1833: 16]	*Apotomus* Illiger, 1807
*Aptinus* Bonelli [1833: 12]	*Aptinus* Bonelli, 1810
*Argutor* Megerle [1833: 33]	*Argutor* Dejean, 1821
*Aspasia* Dejean [1833: 9]	*Aspasia* Dejean, 1831
*Axinotoma* Dejean [1833: 40]	*Axinotoma* Dejean, 1829
*Badister* Clairville [1833: 27]	*Badister* Clairville, 1806
*Baripus* Dejean [1833: 27]	*Baripus* Dejean, 1828
*Barysomus* Dejean [1833: 41]	*Barysomus* Dejean, 1829
*Bembidium* Latreille [1833: 49]	*Bembidion* Latreille, 1802
*Blemus* Ziegler [1833: 49]	*Blemus* Dejean, 1821
*Blethisa* Bonelli [1833: 22]	*Blethisa* Bonelli, 1810
*Brachinus* Weber [1833: 11]	*Brachinus* Weber, 1801
*Bradybaenus* Dejean [1833: 43]	*Bradybaenus* Dejean, 1829
*Calathus* Bonelli [1833: 28]	*Calathus* Bonelli, 1810
*Calleida* Dejean [1833: 8]	*Calleida* Latreille, 1824
*Callistus* Bonelli [1833: 23]	*Callistus* Bonelli, 1810
*Calosoma* Weber [1833: 21]	*Calosoma* Weber, 1801
*Camptodontus* Dejean [1833: 15]	*Camptodontus* Dejean, 1826
*Camptoscelis* Dejean [1833: 37]	*Camptoscelis* Dejean, 1828
*Carabus* Linné [1833: 17]	*Carabus* Linnaeus, 1758
*Cardiaderus* Dejean [1833: 27]	*Cardiaderus* Dejean, 1828
*Carterus* Dejean [1833: 16]	*Carterus* Dejean, 1830
*Casnonia* Latreille [1833: 6]	*Cosnania* Dejean, 1821
*Catadromus* Mac Leay [1833: 31]	*Catadromus* Macleay, 1825
*Catascopus* Kirby [1833: 13]	*Catascopus* Kirby, 1825
*Cephalotes* Bonelli [1833: 37]	*Cephalotes* Bonelli, 1810
*Chlaenius* Bonelli [1833: 23]	*Chlaenius* Bonelli, 1810
*Cicindela* Linné [1833: 2]	*Cicindela* Linnaeus, 1758
*Cillenum* Leach [1833: 49]	*Cillenus* Leach, 1819
*Clivina* Latreille [1833: 15]	*Clivina* Latreille, 1802
*Colliuris* Latreille [1833: 5]	*Collyris* Fabricius, 1801
*Cophosus* Ziegler [1833: 35]	*Cophosus* Dejean, 1821
*Coptodera* Dejean [1833: 10]	*Coptodera* Dejean, 1825
*Cordistes* Latreille [1833: 6]	*Cordistes* Dejean, 1821
*Corsyra* Stéven [1833: 12]	*Corsyra* Dejean, 1825
*Coscinia* Dejean [1833: 14]	*Coscinia* Dejean, 1831
*Cratacanthus* Dejean [1833: 41]	*Cratacanthus* Dejean, 1829
*Cratocerus* Dejean [1833: 40]	*Cratocerus* Dejean, 1829
*Cratognathus* Dejean [1833: 41]	*Cratognathus* Dejean, 1829
*Ctenodactyla* Dejean [1833: 6]	*Ctenodactyla* Dejean, 1825
*Ctenostoma* Klug [1833: 5]	*Ctenostoma* Klug, 1821
*Cychrus* Fabricius [1833: 16]	*Cychrus* Fabricius, 1794
*Cyclosomus* Latreille [1833: 40]	*Cyclosomus* Latreille, 1829
*Cymindis* Latreille [1833: 7]	*Cymindis* Latreille, 1806
*Daptus* Fischer [1833: 40]	*Daptus* Fischer von Waldheim, 1824
*Demetrias* Bonelli [1833: 8]	*Demetrias* Bonelli, 1810
*Diaphorus* Dejean [1833: 7]	*Diaphorus* Dejean, 1831
*Dicaelus* Bonelli [1833: 26]	*Dicaelus* Bonelli, 1813
*Dinodes* Bonelli [1833: 26]	*Dinodes* Bonelli, 1810
*Distrigus* Dejean [1833: 32]	*Distrigus* Dejean, 1828
*Ditomus* Bonelli [1833: 16]	*Ditomus* Bonelli, 1810
*Dolichus* Bonelli [1833: 28]	*Dolichus* Bonelli, 1810
*Drepanus* Illiger [1833: 12]	*Drepanus* Dejean, 1831
*Drimostoma* Dejean [1833: 32]	*Drimostoma* Dejean, 1831
*Dromica* Dejean [1833: 5]	*Dromica* Dejean, 1826
*Dromius* Bonelli [1833: 8]	*Dromius* Bonelli, 1810
*Drypta* Fabricius [1833: 6]	*Drypta* Latreille, 1797
*Dyscolus* Dejean [1833: 12]	*Dyscolus* Dejean, 1831
*Elaphrus* Fabricius [1833: 23]	*Elaphrus* Fabricius, 1775
*Enceladus* Bonelli [1833: 13]	*Enceladus* Bonelli, 1813
*Epomis* Bonelli [1833: 25]	*Epomis* Bonelli, 1810
*Eripus* Höpfner [1833: 40]	*Eripus* Dejean, 1829
*Eucheila* Dejean [1833: 13]	*Eucheila* Dejean, 1831
*Euprosopus* Latreille [1833: 5]	*Euprosopus* Dejean, 1825
*Eurysoma* Oberleitner [1833: 23]	*Eurysoma* Dejean, 1830
*Feronia* Latreille [1833: 32]	*Feronia* Latreille, 1817
*Galerita* Fabricius [1833: 6]	*Galerita* Fabricius, 1801
*Geobaenus* Dejean [1833: 47]	*Geobaenus* Dejean, 1829
*Geobius* Dejean [1833: 23]	*Geobius* Dejean, 1831
*Geodromus* Dejean [1833: 43]	*Geodromus* Dejean, 1829
*Graphipterus* Latreille [1833: 13]	*Graphipterus* Latreille, 1802
*Gynandromorphus* Dejean [1833: 43]	*Gynandromorphus* Dejean, 1829
*Gynandropus* Dejean [1833: 41]	*Gynandropus* Dejean, 1831
*Harpalus* Bonelli [1833: 43]	*Harpalus* Bonelli, 1810
*Helluo* Bonelli [1833: 10]	*Helluo* Bonelli, 1813
*Hypolithus* Dejean [1833: 43]	*Hypolithus* Dejean, 1829
*Iresia* Dejean [1833: 1]	*Iresia* Dejean, 1831
*Lachnophorus* Dejean [1833: 49]	*Lachnophorus* Dejean, 1831
*Lasiocera* Dejean [1833: 6]	*Lasiocera* Dejean, 1831
*Lebia* Latreille [1833: 9]	*Lebia* Latreille, 1802
*Leistus* Froehlich [1833: 21]	*Leistus* Frölich, 1799
*Leja* Megerle [1833: 51]	*Leja* Dejean, 1821
*Leptotrachelus* Latreille [1833: 6]	*Leptotrachelus* Latreille, 1829
*Lesticus* Dejean [1833: 31]	*Lesticus* Dejean, 1828
*Licinus* Latreille [1833: 26]	*Licinus* Latreille, 1802
*Lopha* Megerle [1833: 52]	*Lopha* Dejean, 1821
*Lophidius* Dejean [1833: 40]	*Lophidius* Dejean, 1831
*Loricera* Latreille [1833: 23]	*Loricera* Latreille, 1802
*Manticora* Fabricius [1833: 1]	*Manticora* Fabricius, 1781
*Masoreus* Ziegler [1833: 40]	*Masoreus* Dejean, 1821
*Megacephala* Latreille [1833: 1]	*Megacephala* Latreille, 1802
*Melaenus* Dejean [1833: 14]	*Melaenus* Dejean, 1831
*Melanotus* Dejean [1833: 27]	*Melanotus* Dejean, 1831
*Metrius* Eschscholtz [1833: 23]	*Metrius* Eschscholtz, 1829
*Microcephalus* Latreille [1833: 32]	*Microcephalus* Dejean, 1828
*Molops* Bonelli [1833: 37]	*Molops* Bonelli, 1810
*Morio* Latreille [1833: 16]	*Morion* Latreille, 1810
*Mormolyce* Hagenbach [1833: 29]	*Mormolyce* Hagenbach, 1825
*Myas* Ziegler [1833: 37]	*Myas* Sturm, 1826
*Nebria* Latreille [1833: 21]	*Nebria* Latreille, 1802
*Notaphus* Megerle [1833: 50]	*Notaphus* Dejean, 1821
*Notiophilus* Duméril [1833: 22]	*Notiophilus* Duméril, 1805
*Odacantha* Fabricius [1833: 6]	*Odacantha* Paykull, 1798
*Olisthopus* Dejean [1833: 31]	*Olisthopus* Dejean, 1828
*Omaseus* Ziegler [1833: 33]	*Omaseus* Dejean, 1821
*Omophron* Latreille [1833: 22]	*Omophron* Latreille, 1802
*Omphreus* Parreys [1833: 27]	*Omphreus* Dejean, 1828
*Onypterygia* Chevrolat [1833: 8]	*Onypterygia* Dejean, 1831
*Oodes* Bonelli [1833: 26]	*Oodes* Bonelli, 1810
*Ophonus* Ziegler [1833: 43]	*Ophonus* Dejean, 1821
*Orthogonius* Dejean [1833: 10]	*Orthogonius* Macleay, 1825
*Oxycheila* Dejean [1833: 1]	*Oxycheila* Dejean, 1825
*Oxygnathus* Dejean [1833: 15]	*Oxygnathus* Dejean, 1826
*Oxystomus* Latreille [1833: 15]	*Oxystomus* Dejean, 1825
*Ozaena* Olivier [1833: 16]	*Ozaena* Olivier, 1812
*Pamborus* Latreille [1833: 17]	*Pamborus* Latreille, 1812
*Panagaeus* Latreille [1833: 23]	*Panagaeus* Latreille, 1802
*Paramecus* Dejean [1833: 41]	*Paramecus* Dejean, 1829
*Pasimachus* Bonelli [1833: 15]	*Pasimachus* Bonelli, 1813
*Patrobus* Megerle [1833: 27]	*Patrobus* Dejean, 1821
*Pelecium* Kirby [1833: 40]	*Pelecium* Kirby, 1817
*Pelophila* Dejean [1833: 22]	*Pelophila* Dejean, 1821
*Pelor* Bonelli [1833: 37]	*Pelor* Bonelli, 1810
*Percus* Bonelli [1833: 36]	*Percus* Bonelli, 1810
*Peryphus* Megerle [1833: 51]	*Peryphus* Dejean, 1821
*Platychile* Mac Leay [1833: 1]	*Platychile* Macleay, 1825
*Platymetopus* Dejean [1833: 41]	*Platymetopus* Dejean, 1829
*Platynus* Bonelli [1833: 29]	*Platynus* Bonelli, 1810
*Platysma* Sturm [1833: 34]	*Platysma* Bonelli, 1810
*Plochionus* Dejean [1833: 9]	*Plochionus* Dejean, 1821
*Poecilus* Bonelli [1833: 32]	*Poecilus* Bonelli, 1810
*Pogonus* Ziegler [1833: 27]	*Pogonus* Dejean, 1821
*Polistichus* Bonelli [1833: 7]	*Polistichus* Bonelli, 1810
*Pristodactyla* Dejean [1833: 29]	*Pristodactyla* Dejean, 1828
*Pristonychus* Dejean [1833: 28]	*Pristonychus* Dejean, 1828
*Procerus* Megerle [1833: 17]	*Procerus* Dejean, 1821
*Procrustes* Bonelli [1833: 17]	*Procrustes* Bonelli, 1810
*Promecoderus* Dejean [1833: 40]	*Promecoderus* Dejean, 1829
*Promecoptera* Dejean [1833: 12]	*Promecoptera* Dejean, 1831
*Pteroloma* Schönherr [1833: 21]	*Pteroloma* Gyllenhal, 1827
*Pterostichus* Bonelli [1833: 35]	*Pterostichus* Bonelli, 1810
*Rathymus* Dejean [1833: 37]	*Rathymus* Dejean, 1831
*Rembus* Latreille [1833: 26]	*Rembus* Dejean, 1826
*Scaphinotus* Latreille [1833: 17]	*Scaphinotus* Dejean, 1826
*Scapterus* Dejean [1833: 15]	*Scapterus* Dejean, 1826
*Scarites* Fabricius [1833: 14]	*Scarites* Fabricius, 1775
*Selenophorus* Dejean [1833: 41]	*Selenophorus* Dejean, 1829
*Siagona* Latreille [1833: 13]	*Siagona* Latreille, 1804
*Somoplatus* Dejean [1833: 40]	*Somoplatus* Dejean, 1829
*Sphaeroderus* Dejean [1833: 17]	*Sphaeroderus* Dejean, 1826
*Sphodrus* Clairville [1833: 29]	*Sphodrus* Clairville, 1806
*Stenolophus* Megerle [1833: 47]	*Stenolophus* Dejean, 1821
*Stenomorphus* Dejean [1833: 27]	*Stenomorphus* Dejean, 1831
*Steropus* Megerle [1833: 34]	*Steropus* Dejean, 1821
*Stomis* Clairville [1833: 37]	*Stomis* Clairville, 1806
*Tachypus* Megerle [1833: 52]	*Tachypus* Dejean, 1821
*Tachys* Megerle [1833: 49]	*Tachys* Dejean, 1821
*Taphria* Bonelli [1833: 29]	*Taphria* Dejean, 1821
*Tefflus* Leach [1833: 17]	*Tefflus* Leach, 1819
*Tetragonoderus* Dejean [1833: 48]	*Tetragonoderus* Dejean, 1829
*Therates* Latreille [1833: 5]	*Therates* Latreille, 1817
*Thyreopterus* Dejean [1833: 12]	*Thyreopterus* Dejean, 1831
*Trechus* Clairville [1833: 48]	*Trechus* Clairville, 1806
*Trichognathus* Latreille [1833: 6]	*Trichognathus* Latreille, 1829
*Tricondyla* Latreille [1833: 5]	*Tricondyla* Latreille, 1822
*Trigonodactyla* Dejean [1833: 6]	*Trigonodactyla* Dejean, 1831
*Trigonotoma* Dejean [1833: 31]	*Trigonotoma* Dejean, 1828
*Vertagus* Dejean [1833: 23]	*Vertagus* Dejean, 1831
*Zabrus* Clairville [1833: 37]	*Zabrus* Clairville, 1806
*Zuphium* Latreille [1833: 7]	*Zuphium* Latreille, 1806
**Pentamères: Hydrocanthares**	
*Acilius* Leach [1833: 53]	*Acilius* Leach, 1817
*Agabus* Leach [1833: 54]	*Agabus* Leach, 1817
*Colymbetes* Clairville [1833: 55]	*Colymbetes* Clairville, 1806
*Dytiscus* Linné [1833: 53]	*Dytiscus* Linnaeus, 1758
*Gyrinus* Linné [1833: 58]	*Gyrinus* Geoffroy, 1762
*Haliplus* Latreille [1833: 56]	*Haliplus* Latreille, 1802
*Hydaticus* Leach [1833: 54]	*Hydaticus* Leach, 1817
*Hydroporus* Latreille [1833: 56]	*Hydroporus* Clairville, 1806
*Hygrobia* Latreille [1833: 56]	*Hygrobia* Latreille, 1804
*Hyphidrus* Latreille [1833: 58]	*Hyphydrus* Illiger, 1802
*Laccophilus* Leach [1833: 56]	*Laccophilus* Leach, 1815
*Noterus* Latreille [1833: 56]	*Noterus* Clairville, 1806
**Pentamères: Brachélytres**	
*Achelium* Leach [1833: 64]	*Achenium* Leach, 1819
*Acidota* Kirby [1833: 69]	*Acidota* Stephens, 1829
*Aleochara* Gravenhorst [1833: 71]	*Aleochara* Gravenhorst, 1802
*Anthobium* Leach [1833: 68]	*Anthobium* Leach, 1819
*Anthophagus* Gravenhorst [1833: 68]	*Anthophagus* Gravenhorst, 1802
*Astrapaeus* Gravenhorst [1833: 61]	*Astrapaeus* Gravenhorst, 1802
*Autalia* Leach [1833: 74]	*Autalia* Leach, 1819
*Bledius* Leach [1833: 67]	*Bledius* Leach, 1819
*Bolitobius* Leach [1833: 69]	*Bolitobius* Leach, 1819
*Bolitochara* Mannerheim [1833: 72]	*Bolitochara* Mannerheim, 1830
*Cafius* Leach [1833: 63]	*Cafius* Stephens, 1829
*Calodera* Mannerheim [1833: 74]	*Calodera* Mannerheim, 1830
*Cryptobium* Mannerheim [1833: 65]	*Cryptobium* Mannerheim, 1830
*Dianous* Leach [1833: 66]	*Dianous* Leach, 1819
*Dinarda* Leach [1833: 71]	*Dinarda* Leach, 1819
*Drusilla* Leach [1833: 74]	*Drusilla* Leach, 1819
*Emus* Leach [1833: 59]	*Emus* Leach, 1819
*Eulissus* Mannerheim [1833: 63]	*Eulissus* Mannerheim, 1830
*Evaesthetus* Gravenhorst [1833: 66]	*Euaesthetus* Gravenhorst, 1806
*Falagria* Leach [1833: 74]	*Falagria* Leach, 1819
*Girophaena* Mannerheim [1833: 72]	*Gyrophaena* Mannerheim, 1830
*Gymnusa* Karsten [1833: 72]	*Gymnusa* Gravenhorst, 1806
*Homalota* Mannerheim [1833: 74]	*Homalota* Mannerheim, 1830
*Hypocyphtus* Schüppel [1833: 71]	*Hypocyphtus* Gyllenhal, 1827
*Lathrobium* Gravenhorst [1833: 64]	*Lathrobium* Gravenhorst, 1802
*Lomechusa* Gravenhorst [1833: 71]	*Lomechusa* Gravenhorst, 1806
*Mycetoporus* Mannerheim [1833: 70]	*Mycetoporus* Mannerheim, 1830
*Omalium* Gravenhorst [1833: 69]	*Omalium* Gravenhorst, 1802
*Osorius* Leach [1833: 67]	*Osorius* Guérin-Méneville, 1829
*Oxypoda* Mannerheim [1833: 72]	*Oxypoda* Mannerheim, 1830
*Oxyporus* Fabricius [1833: 66]	*Oxyporus* Fabricius, 1775
*Oxytelus* Gravenhorst [1833: 67]	*Oxytelus* Gravenhorst, 1802
*Paederus* Fabricius [1833: 65]	*Paederus* Fabricius, 1775
*Phloeocharis* Mannerheim [1833: 68]	*Phloeocharis* Mannerheim, 1830
*Platyprosopus* Mannerheim [1833: 64]	*Platyprosopus* Mannerheim, 1830
*Platystethus* Mannerheim [1833: 67]	*Platystethus* Mannerheim, 1830
*Prognathus* Latreille [1833: 67]	*Prognathus* Blondel, 1827
*Proteinus* Latreille [1833: 69]	*Proteinus* Latreille, 1797
*Rugilus* Leach [1833: 65]	*Rugilus* Leach, 1819
*Sphenoma* Mannerheim [1833: 72]	*Sphenoma* Mannerheim, 1830
*Staphylinus* Linné [1833: 61]	*Staphylinus* Linnaeus, 1758
*Stenus* Fabricius [1833: 66]	*Stenus* Latreille, 1797
*Stilicus* Leach [1833: 64]	*Stilicus* Berthold, 1827
*Tachinus* Gravenhorst [1833: 70]	*Tachinus* Gravenhorst, 1802
*Tachyporus* Gravenhorst [1833: 70]	*Tachyporus* Gravenhorst, 1802
*Taenosoma* Mannerheim [1833: 68]	*Taenosoma* Mannerheim, 1830
*Trogophloeus* Mannerheim [1833: 68]	*Trogophloeus* Mannerheim, 1830
*Trychophya* Mannerheim [1833: 72]	*Trichophya* Mannerheim, 1830
*Velleius* Leach [1833: 59]	*Velleius* Leach, 1819
*Xantholinus* Dahl [1833: 63]	*Xantholinus* Dejean, 1821
*Zirophorus* Dalman [1833: 67]	*Zirophorus* Dalman, 1821
**Pentamères: Sternoxes**	
*Acmaeodera* Eschscholtz [1833: 75]	*Acmaeodera* Eschscholtz, 1829
*Adrastus* Megerle [1833: 96]	*Adrastus* Eschscholtz, 1829
*Aeolus* Eschscholtz [1833: 91]	*Aeolus* Eschscholtz, 1829
*Agrilus* Megerle [1833: 81]	*Agrilus* Curtis, 1825
*Agriotes* Eschscholtz [1833: 95]	*Agriotes* Eschscholtz, 1829
*Agrypnus* Eschscholtz [1833: 88]	*Agrypnus* Eschscholtz, 1829
*Alaus* Eschscholtz [1833: 89]	*Alaus* Eschscholtz, 1829
*Ancylocheira* Eschscholtz [1833: 77]	*Ancylocheira* Eschscholtz, 1829
*Anthaxia* Eschscholtz [1833: 80]	*Anthaxia* Eschscholtz, 1829
*Aphanisticus* Latreille [1833: 84]	*Aphanisticus* Latreille, 1810
*Aphanobius* Eschscholtz [1833: 92]	*Aphanobius* Eschscholtz, 1829
*Aptopus* Eschscholtz [1833: 87]	*Aptopus* Eschscholtz, 1829
*Athous* Eschscholtz [1833: 89]	*Athous* Eschscholtz, 1829
*Belionota* Eschscholtz [1833: 80]	*Belionota* Eschscholtz, 1829
*Callirhipis* Latreille [1833: 84]	*Callirhipis* Latreille, 1829
*Campylus* Fischer [1833: 90]	*Campylus* Fischer von Waldheim, 1821
*Capnodis* Eschscholtz [1833: 77]	*Capnodis* Eschscholtz, 1829
*Cardiophorus* Eschscholtz [1833: 91]	*Cardiophorus* Eschscholtz, 1829
*Cardiorhinus* Eschscholtz [1833: 95]	*Cardiorhinus* Eschscholtz, 1829
*Cerophytum* Latreille [1833: 84]	*Cerophytum* Latreille, 1809
*Chalcolepidius* Eschscholtz [1833: 89]	*Chalcolepidius* Eschscholtz, 1829
*Chrysobothris* Eschscholtz [1833: 79]	*Chrysobotris* Eschscholtz, 1829
*Conoderus* Eschscholtz [1833: 86]	*Conoderus* Eschscholtz, 1829
*Conognatha* Eschscholtz [1833: 76]	*Conognatha* Eschscholtz, 1829
*Cosmesus* Eschscholtz [1833: 95]	*Cosmesus* Eschscholtz, 1829
*Cryptohypnus* Eschscholtz [1833: 93]	*Cryptohypnus* Eschscholtz, 1830
*Cryptostoma* Dejean [1833: 84]	*Cryptostoma* Dejean, 1821
*Dicerea* Eschscholtz [1833: 77]	*Dicerca* Eschscholtz, 1829
*Dicrepidius* Eschscholtz [1833: 85]	*Dicrepidius* Eschscholtz, 1829
*Dicronychus* Eschscholtz [1833: 88]	*Dicronychus* Brullé, 1832
*Dima* Ziegler [1833: 87]	*Dima* Charpentier, 1825
*Dolopius* Megerle [1833: 96]	*Dalopius* Eschscholtz, 1829
*Drapetes* Megerle [1833: 84]	*Drapetes* Dejean, 1821
*Drasterius* Eschscholtz [1833: 93]	*Drasterius* Eschscholtz, 1829
*Ectinus* Eschscholtz [1833: 96]	*Ectinus* Eschscholtz, 1829
*Elater* Eschscholtz [1833: 92]	*Elater* Linnaeus, 1758
*Eucnemis* Ahrens [1833: 84]	*Eucnemis* Ahrens, 1812
*Galba* Latreille [1833: 84]	*Galba* Latreille, 1829
*Hemirhipus* Latreille [1833: 89]	*Hemirhipus* Dillwyn, 1829
*Hypolithus* Eschscholtz [1833: 93]	*Hypolithus* Eschscholtz, 1829
*Julodis* Eschscholtz [1833: 74]	*Julodis* Eschscholtz, 1829
*Limonius* Eschscholtz [1833: 90]	*Limonius* Eschscholtz, 1829
*Lissomus* Dalman [1833: 84]	*Lissomus* Dalman, 1824
*Ludius* Latreille [1833: 94]	*Ludius* Berthold, 1827
*Melanotus* Eschscholtz [1833: 87]	*Melanotus* Eschscholtz, 1829
*Melasis* Fabricius [1833: 84]	*Melasis* Olivier, 1790
*Monocrepidius* Eschscholtz [1833: 86]	*Monocrepidius* Eschscholtz, 1829
*Nematodes* Latreille [1833: 85]	*Nematodes* Berthold, 1827
*Pericalus* Encyclopédie [1833: 85]	*Pericallus* Lepeletier & Audinet-Serville, 1828
*Phyllocerus* Dejean [1833: 84]	*Phyllocerus* Lepeletier & Audinet-Serville, 1825
*Poecilonota* Eschscholtz [1833: 76]	*Poecilonota* Eschscholtz, 1829
*Pomachilius* Eschscholtz [1833: 86]	*Pomachilius* Eschscholtz, 1829
*Pyrophorus* Illiger [1833: 88]	*Pyrophorus* Billberg, 1820
*Sericus* Eschscholtz [1833: 96]	*Sericus* Eschscholtz, 1829
*Sternocera* Eschscholtz [1833: 74]	*Sternocera* Eschscholtz, 1829
*Synaptus* Eschscholtz [1833: 87]	*Synaptus* Eschscholtz, 1829
*Tetralobus* Encyclopédie [1833: 85]	*Tetralobus* Lepeletier & Audinet-Serville, 1828
*Trachys* Fabricius [1833: 83]	*Trachys* Fabricius, 1801
*Xylophilus* Mannerheim [1833: 85]	*Xylophilus* Mannerheim, 1823
**Pentamères: Malacodermes**	
*Amydetes* Hoffmansegg [1833: 100]	*Amydetes* Illiger, 1807
*Atopa* Fabricius [1833: 97]	*Atopa* Paykull, 1799
*Cantharis* Linné [1833: 105]	*Cantharis* Linnaeus, 1758
*Cebrio* Fabricius [1833: 96]	*Cebrio* Olivier, 1790
*Cyphon* Fabricius [1833: 97]	*Cyphon* Paykull, 1799
*Dasytes* Fabricius [1833: 110]	*Dasytes* Paykull, 1799
*Drilus* Olivier [1833: 104]	*Drilus* Olivier, 1790
*Dyctyopterus* Latreille [1833: 100]	*Dictyoptera* Latreille, 1829
*Eubria* Ziegler [1833: 98]	*Eubria* Germar, 1818
*Lampyris* Linné [1833: 103]	*Lampyris* Geoffroy, 1762
*Lycus* Fabricius [1833: 98]	*Lycus* Fabricius, 1787
*Malachius* Fabricius [1833: 108]	*Malachius* Fabricius, 1775
*Malthinus* Latreille [1833: 108]	*Malthinus* Latreille, 1806
*Melyris* Fabricius [1833: 111]	*Melyris* Fabricius, 1775
*Nycteus* Latreille [1833: 98]	*Nycteus* Latreille, 1829
*Omalisus* Geoffroy [1833: 100]	*Omalisus* Geoffroy, 1762
*Pelecophora* Dejean [1833: 111]	*Pelecophora* Dejean, 1821
*Phengodes* Hoffmansegg [1833: 100]	*Phengodes* Illiger, 1807
*Ptilodactyla* Illiger [1833: 97]	*Ptilodactyla* Illiger, 1807
*Rhipicera* Latreille [1833: 96]	*Rhipicera* Latreille, 1817
*Sandalus* Knoch [1833: 97]	*Sandalus* Knoch, 1801
*Scyrtes* Latreille [1833: 98]	*Scirtes* Illiger, 1807
*Silis* Megerle [1833: 107]	*Silis* Charpentier, 1825
*Zygia* Fabricius [1833: 111]	*Zygia* Fabricius, 1775
**Pentamères: Terediles**	
*Anobium* Fabricius [1833: 116]	*Anobium* Fabricius, 1775
*Atractocerus* Palisot Beauvois [1833: 114]	*Atractocerus* Palisot de Beauvois, 1802
*Clerus* Fabricius [1833: 113]	*Clerus* Geoffroy, 1762
*Corynetes* Fabricius [1833: 114]	*Korynetes* Herbst, 1792
*Cupes* Fabricius [1833: 114]	*Cupes* Fabricius, 1801
*Cylidrus* Latreille [1833: 112]	*Cylidrus* Latreille, 1829
*Dorcatoma* Fabricius [1833: 115]	*Dorcatoma* Herbst, 1792
*Enoplium* Latreille [1833: 114]	*Enoplium* Latreille, 1802
*Eurypus* Kirby [1833: 112]	*Eurypus* Kirby, 1819
*Gibbium* Scopoli [1833: 117]	*Gibbium* Scopoli, 1777
*Hedobia* Ziegler [1833: 116]	*Hedobia* Dejean, 1821
*Hylecoetus* Latreille [1833: 114]	*Hylecoetus* Latreille, 1806
*Lymexylon* Fabricius [1833: 114]	*Lymexylon* Fabricius, 1775
*Mastigus* Hoffmansegg [1833: 117]	*Mastigus* Latreille, 1802
*Notoxus* Fabricius [1833: 112]	*Notoxus* Fabricius, 1775
*Ochina* Ziegler [1833: 115]	*Ochina* Dejean, 1821
*Ptilinus* Geoffroy [1833: 115]	*Ptilinus* Geoffroy, 1762
*Ptinus* Linné [1833: 116]	*Ptinus* Linnaeus, 1767
*Rhysodes* Latreille [1833: 114]	*Rhysodes* Dalman, 1823
*Scydmaenus* Latreille [1833: 117]	*Scydmaenus* Latreille, 1802
*Tillus* Fabricius [1833: 112]	*Tillus* Olivier, 1790
*Trichodes* Fabricius [1833: 112]	*Trichodes* Herbst, 1792
*Xyletinus* Latreille [1833: 115]	*Xyletinus* Latreille, 1810
**Pentamères: Clavicornes**	
*Abraeus* Leach [1833: 128]	*Abraeus* Leach, 1817
*Agyrtes* Froehlich [1833: 119]	*Agyrtes* Frölich, 1799
*Antherophagus* Knoch [1833: 123]	*Antherophagus* Dejean, 1821
*Anthrenus* Fabricius [1833: 125]	*Anthrenus* Geoffroy, 1762
*Aspidiphorus* Ziegler [1833: 125]	*Aspidiphorus* Dejean, 1821
*Attagenus* Latreille [1833: 124]	*Attagenus* Latreille, 1802
*Byrrhus* Fabricius [1833: 130]	*Byrrhus* Linnaeus, 1767
*Byturus* Latreille [1833: 122]	*Byturus* Latreille, 1797
*Catops* Fabricius [1833: 119]	*Catops* Paykull, 1798
*Cercus* Latreille [1833: 122]	*Cercus* Latreille, 1797
*Ceuthocerus* Schüppel [1833: 129]	*Ceutocerus* Germar, 1824
*Chelonarium* Fabricius [1833: 130]	*Chelonarium* Fabricius, 1801
*Colobicus* Latreille [1833: 120]	*Colobicus* Latreille, 1807
*Cryptophagus* Herbst [1833: 123]	*Cryptophagus* Herbst, 1792
*Dendrophilus* Leach [1833: 128]	*Dendrophilus* Leach, 1817
*Dermestes* Linné [1833: 124]	*Dermestes* Linnaeus, 1758
*Elmis* Latreille [1833: 131]	*Elmis* Latreille, 1802
*Engis* Fabricius [1833: 123]	*Engis* Paykull, 1800
*Georissus* Latreille [1833: 131]	*Georissus* Latreille, 1809
*Helota* Mac Leay [1833: 120]	*Helota* Macleay, 1825
*Heterocerus* Fabricius [1833: 131]	*Heterocerus* Fabricius, 1792
*Hister* Linné [1833: 125]	*Hister* Linnaeus, 1758
*Hololepta* Paykull [1833: 129]	*Hololepta* Paykull, 1811
*Ips* Fabricius [1833: 120]	*Ips* Fabricius, 1777
*Limnichus* Ziegler [1833: 130]	*Limnichus* Dejean, 1821
*Macronychus* Müller [1833: 131]	*Macronychus* Müller, 1806
*Megatoma* Latreille [1833: 125]	*Megatoma* Herbst, 1792
*Micropeplus* Latreille [1833: 122]	*Micropeplus* Latreille, 1809
*Necrodes* Wilkin [1833: 118]	*Necrodes* Leach, 1815
*Necrophilus* Latreille [1833: 118]	*Necrophilus* Latreille, 1829
*Necrophorus* Fabricius [1833: 117]	*Nicrophorus* Fabricius, 1775
*Nitidula* Fabricius [1833: 121]	*Nitidula* Fabricius, 1775
*Nosodendron* Latreille [1833: 130]	*Nosodendron* Latreille, 1804
*Onthophilus* Leach [1833: 128]	*Onthophilus* Leach, 1817
*Parnus* Fabricius [1833: 131]	*Parnus* Fabricius, 1792
*Peltis* Fabricius [1833: 119]	*Peltis* Geoffroy, 1762
*Platysoma* Leach [1833: 128]	*Platysoma* Leach, 1817
*Potamophilus* Germar [1833: 131]	*Potamophilus* Germar, 1811
*Ptilium* Schüppel [1833: 124]	*Ptilium* Gyllenhal, 1827
*Scaphidium* Fabricius [1833: 119]	*Scaphidium* Olivier, 1790
*Silpha* Linné [1833: 118]	*Silpha* Linnaeus, 1758
*Sphaerites* Duftschmid [1833: 119]	*Sphaerites* Duftschmid, 1805
*Strongylus* Herbst [1833: 120]	*Strongylus* Herbst, 1792
*Throscus* Latreille [1833: 130]	*Throscus* Latreille, 1797
*Thymalus* Latreille [1833: 119]	*Thymalus* Latreille, 1802
*Trinodes* Megerle [1833: 125]	*Trinodes* Dejean, 1821
*Trogoderma* Latreille [1833: 125]	*Trogoderma* Dejean, 1821
*Trypaneus* Godet [1833: 129]	*Trypanaeus* Eschscholtz, 1829
**Pentamères: Palpicornes**	
*Berosus* Leach [1833: 132]	*Berosus* Leach, 1817
*Cercyon* Leach [1833: 134]	*Cercyon* Leach, 1817
*Elophorus* Fabricius [1833: 132]	*Helophorus* Fabricius, 1775
*Hydraena* Kugellann [1833: 132]	*Hydraena* Kugelann, 1794
*Hydrobius* Leach [1833: 133]	*Hydrobius* Leach, 1815
*Hydrochus* Germar [1833: 132]	*Hydrochus* Leach, 1817
*Hydrophilus* Fabricius [1833: 133]	*Hydrophilus* Geoffroy, 1762
*Ochthebius* Leach [1833: 132]	*Ochthebius* Leach, 1815
*Spercheus* Fabricius [1833: 132]	*Spercheus* Kugelann, 1798
*Sphaeridium* Fabricius [1833: 134]	*Sphaeridium* Fabricius, 1775
**Pentamères: Lamellicornes**	
*Acanthocerus* Mac Leay [1833: 147]	*Acanthocerus* Macleay, 1819
*Acanthurus* Kirby [1833: 168]	*Acanthurus* Kirby, 1827
*Aegialia* Latreille [1833: 147]	*Aegialia* Latreille, 1807
*Aeschrotes* Mac Leay [1833: 144]	*Aeschrotes* Lepeletier & Audinet-Serville, 1828
*Aesalus* Fabricius [1833: 175]	*Aesalus* Fabricius, 1801
*Agacephala* Mannerheim [1833: 153]	*Agaocephala* Audinet-Serville, 1828
*Agenius* Encyclopédie [1833: 168]	*Agenius* Lepeletier & Audinet-Serville, 1828
*Amblyterus* Mac Leay [1833: 155]	*Amblyterus* Macleay, 1819
*Amphicoma* Latreille [1833: 167]	*Amphicoma* Latreille, 1807
*Amphistoros* Gory [1833: 170]	*Amphistoros* Gory & Percheron, 1833 [**3**]
*Anisonyx* Latreille [1833: 167]	*Anisonyx* Latreille, 1807
*Anisoplia* Megerle [1833: 158]	*Anisoplia* Fischer von Waldheim, 1824
*Anomala* Megerle [1833: 155]	*Anomala* Samouelle, 1819
*Anoplognathus* Leach [1833: 154]	*Anoplognathus* Leach, 1815
*Anthipna* Eschscholtz [1833: 167]	*Anthypna* Eschscholtz, 1818
*Aphodius* Fabricius [1833: 144]	*Aphodius* Hellwig, 1798
*Apogonia* Kirby [1833: 162]	*Apogonia* Kirby, 1819
*Areoda* Mac Leay [1833: 155]	*Areoda* Macleay, 1819
*Ateuchus* Fabricius [1833: 135]	*Ateuchus* Weber, 1801
*Athyreus* Mac Leay [1833: 149]	*Athyreus* Macleay, 1819
*Aulacodus* Eschscholtz [1833: 157]	*Aulacodus* Eschscholtz, 1822
*Bolboceras* Kirby [1833: 149]	*Bolboceras* Kirby, 1819
*Campulipus* Kirby [1833: 168]	*Campulipus* Kirby, 1827
*Ceraspis* Encyclopédie [1833: 163]	*Ceraspis* Lepeletier & Audinet-Serville, 1828
*Cetonia* Fabricius [1833: 171]	*Cetonia* Fabricius, 1775
*Chalcimon* Dalman [1833: 173]	*Chalcimon* Dalman, 1824
*Chalepus* Mac Leay [1833: 152]	*Chalepus* Macleay, 1819
*Chasme* Encyclopédie [1833: 165]	*Chasme* Lepeletier & Audiet-Serville, 1828
*Chasmatopterus* Dejean [1833: 165]	*Chasmatopterus* Dejean, 1821
*Chasmodia* Mac Leay [1833: 154]	*Chasmodia* Macleay, 1819
*Chiron* Mac Leay [1833: 147]	*Chiron* Macleay, 1819
*Choeridium* Encyclopédie [1833: 137]	*Choeridium* Lepeletier & Audinet-Serville, 1828
*Chrysophora* Dejean [1833: 153]	*Chrysophora* Dejean, 1821
*Circellium* Latreille [1833: 136]	*Circellium* Latreille, 1829
*Cnemida* Kirby [1833: 154]	*Cnemida* Kirby, 1827
*Copris* Fabricius [1833: 138]	*Copris* Geoffroy, 1762
*Coprobius* Latreille [1833: 136]	*Coprobius* Latreille, 1829
*Cremastocheilus* Knoch [1833: 169]	*Cremastocheilus* Knoch, 1801
*Cyclocephala* Latreille [1833: 152]	*Cyclocephala* Dejean, 1821
*Dasygnathus* Mac Leay [1833: 152]	*Dasygnathus* Macleay, 1819
*Dasyus* Encyclopédie [1833: 164]	*Dasyus* Lepeletier & Audinet-Serville, 1828
*Diasomus* Dalman [1833: 147]	*Diasomus* Dalman, 1824
*Dichelus* Encyclopédie [1833: 166]	*Dichelus* Lepeletier & Audinet-Serville, 1828
*Dicheros* Gory [1833: 169]	*Dicheros* Gory & Percheron, 1833 [**3**]
*Dicrania* Encyclopédie [1833: 162]	*Dicrania* Lepeletier & Audinet-Serville, 1828
*Diphucephala* Dejean [1833: 162]	*Diphucephala* Dejean, 1821
*Diplognatha* Gory [1833: 171]	*Diplognatha* Gory & Percheron, 1833 [**3**]
*Dorcus* Megerle [1833: 174]	*Dorcus* Macleay, 1819
*Euchlora* Mac Leay [1833: 155]	*Euchlora* Macleay, 1819
*Euparia* Encyclopédie [1833: 147]	*Euparia* Lepeletier & Audinet-Serville, 1828
*Eurysternus* Dalman [1833: 144]	*Eurysternus* Dalman, 1824
*Figulus* Mac Leay [1833: 175]	*Figulus* Macleay, 1819
*Geniates* Kirby [1833: 157]	*Geniates* Kirby, 1819
*Genucus* Mac Leay [1833: 169]	*Genuchus* Macleay, 1825
*Geotrupes* Latreille [1833: 148]	*Geotrupes* Latreille, 1797
*Glaphyrus* Latreille [1833: 167]	*Glaphyrus* Latreille, 1802
*Gnathocera* Kirby [1833: 170]	*Gnathocera* Kirby, 1825
*Gnorimus* Encyclopédie [1833: 168]	*Gnorimus* Lepeletier & Audinet-Serville, 1828
*Goliathus* de Lamarck [1833: 169]	*Goliathus* Lamarck, 1801
*Gymnetis* Mac Leay [1833: 169]	*Gymnetis* Macleay, 1819
*Gymnopleurus* Illiger [1833: 135]	*Gymnopleurus* Illiger, 1803
*Hoplia* Illiger [1833: 166]	*Hoplia* Illiger, 1803
*Hyboma* Encyclopédie [1833: 136]	*Hyboma* Lepeletier & Audinet-Serville, 1828
*Hybosorus* Mac Leay [1833: 149]	*Hybosorus* Macleay, 1819
*Hymenontia* Eschscholtz [1833: 165]	*Hymenoplia* Eschscholtz, 1830^2^
*Inca* Encyclopédie [1833: 168]	*Inca* Lepeletier & Audinet-Serville, 1828
*Isonichus* Mannerheim [1833: 164]	*Isonychus* Mannerheim, 1829
*Lamprima* Latreille [1833: 173]	*Lamprima* Latreille, 1804
*Lepisia* Encyclopédie [1833: 166]	*Lepisia* Lepeletier & Audinet-Serville, 1828
*Lepitrix* Encyclopédie [1833: 167]	*Lepitrix* Lepeletier & Audinet-Serville, 1828
*Lethrus* Fabricius [1833: 148]	*Lethrus* Scopoli, 1777
*Leucothyreus* Mac Leay [1833: 157]	*Leucothyreus* Macleay, 1819
*Liparetrus* Mac Leay [1833: 164]	*Liparetus* Guérin-Méneville, 1831
*Lonchophorus* Germar [1833: 139]	*Lonchophorus* Germar, 1824
*Lucanus* Linné [1833: 174]	*Lucanus* Scopoli, 1763
*Macraspis* Mac Leay [1833: 154]	*Macraspis* Macleay, 1819
*Macrodactylus* Latreille [1833: 163]	*Macrodactylus* Dejean, 1821
*Macroma* Kirby [1833: 171]	*Macroma* Gory & Percheron, 1833 [**3**]
*Macronota* Wiedemann [1833: 170]	*Macronota* Hoffmannsegg, 1817
*Melolontha* Fabricius [1833: 158]	*Melolontha* Fabricius, 1775
*Monochelus* Illiger [1833: 166]	*Monochelus* Lepeletier & Audinet-Serville, 1828
*Ochodaeus* Megerle [1833: 149]	*Ochodaeus* Dejean, 1821
*Odontaeus* Meg. [1833: 149]	*Odontaeus* Dejean, 1821
*Omaloplia* Megerle [1833: 165]	*Omaloplia* Schönherr, 1817
*Ometis* Latreille [1833: 154]	*Ometis* Latreille, 1829
*Oniticellus* Ziegler [1833: 143]	*Oniticellus* Dejean, 1821
*Onitis* Fabricius [1833: 143]	*Onitis* Fabricius, 1798
*Onthophagus* Latreille [1833: 140]	*Onthophagus* Latreille, 1802
*Orphnus* Mac Leay [1833: 149]	*Orphnus* Macleay, 1819
*Oryctes* Illiger [1833: 150]	*Oryctes* Hellwig, 1798
*Osmoderma* Encyclopédie [1833: 168]	*Osmoderma* Lepeletier & Audinet-Serville, 1828
*Pachycnema* Encyclopédie [1833: 167]	*Pachycnema* Lepeletier & Audinet-Serville, 1828
*Pachypus* Dejean [1833: 152, 159]	*Pachypus* Dejean, 1821
*Pachysoma* Kirby [1833: 135]	*Pachysoma* Macleay, 1821
*Passalus* Fabricius [1833: 175]	*Passalus* Fabricius, 1792
*Pelidnota* Mac Leay [1833: 153]	*Pelidnota* Macleay, 1819
*Phanaeus* Mac Leay [1833: 139]	*Phanaeus* Macleay, 1819
*Phileurus* Latreille [1833: 150]	*Phileurus* Latreille, 1807
*Pholidotus* Mac Leay [1833: 173]	*Pholidotus* Macleay, 1819
*Platycerus* Latreille [1833: 175]	*Platycerus* Geoffroy, 1762
*Platygenia* Mac Leay [1833: 168]	*Platygenia* Macleay, 1819
*Plectris* Encyclopédie [1833: 162]	*Plectris* Lepeletier & Audinet-Serville, 1828
*Popilia* Leach [1833: 158]	*Popillia* Dejean, 1821
*Psammodius* Gyllenhal [1833: 147]	*Psammodius* Fallén, 1807
*Repsimus* Leach [1833: 155]	*Repsimus* Macleay, 1819
*Rhisotrogus* Latreille [1833: 160]	*Rhizotrogus* Latreille, 1825
*Rutela* Latreille [1833: 153]	*Rutela* Latreille, 1802
*Ryssonotus* Mac Leay [1833: 173]	*Ryssonotus* Macleay, 1819
*Scarabaeus* Latreille [1833: 150]	*Scarabaeus* Linnaeus, 1758
*Schizognathus* Kirby [1833: 155]	*Schizognathus* Fischer von Waldheim, 1823
*Schyzorhina* Kirby [1833: 170]	*Schizorhina* Kirby, 1825
*Serica* Mac Leay [1833: 165]	*Serica* Macleay, 1819
*Sinodendron* Fabricius [1833: 175]	*Sinodendron* Hellwig, 1791
*Sisyphus* Latreille [1833: 136]	*Sisyphus* Latreille, 1807
*Stripsifer* Gory [1833: 168]	*Stripsipher* Gory & Percheron, 1833 [**3**]
*Tanyproctus* Faldermann [1833: 159]	*Tanyproctus* Faldermann, 1832
*Tetragona* Gory [1833: 170]	*Tetragonus* Gory & Percheron, 1833 [**3**]
*Trichius* Fabricius [1833: 168]	*Trichius* Fabricius, 1775
*Trox* Fabricius [1833: 147]	*Trox* Fabricius, 1775
*Valgus* Scriba [1833: 168]	*Valgus* Scriba, 1790
**Hétéromères:** **Mélasomes**	
*Acanthomera* Latreille [1834: 189]	*Acanthomera* Latreille, 1829
*Acanthopus* Megerle [1834: 189]	*Accanthopus* Dejean, 1821
*Adelostoma* Duponchel [1834:183]	*Adelostoma* Duponchel, 1827
*Adesmia* Fischer [1834: 179]	*Adesmia* Fischer von Waldheim, 1822
*Akis* Fabricius [1834: 182]	*Akis* Herbst, 1799
*Anomalipes* Guérin [1834: 187]	*Anomalipes* Guérin-Méneville, 1831
*Asida* Latreille [1834: 187]	*Asida* Latreille, 1802
*Blaps* Fabricius [1834: 188]	*Blaps* Fabricius, 1775
*Blapstinus* Dejean [1834: 192]	*Blapstinus* Dejean, 1821
*Coniontis* Eschscholtz [1834: 190]	*Coniontis* Eschscholtz, 1829
*Crypticus* Latreille [1834: 194]	*Crypticus* Latreille, 1817
*Cryptochyle* Latreille [1834: 180]	*Cryptochyle* Latreille, 1829
*Dendarus* Dejean [1834: 191]	*Dendarus* Dejean, 1821
*Diesia* Fischer [1834: 180]	*Diesia* Fischer von Waldheim, 1820
*Elenophorus* Megerle [1834: 182]	*Elenophorus* Dejean, 1821
*Eleodes* Eschscholtz [1834: 189]	*Eleodes* Eschscholtz, 1829
*Erodius* Fabricius [1834: 177]	*Erodius* Fabricius, 1775
*Eulabis* Eschscholtz [1834: 183]	*Eulabis* Eschscholtz, 1829
*Eurychora* Fabricius [1834: 181]	*Eurychora* Thunberg, 1791
*Eurynotus* Kirby [1834: 190]	*Eurynotus* Kirby, 1819
*Gnaptor* Megerle [1834: 188]	*Gnaptor* Brullé, 1833
*Gnathosia* Fischer [1834: 185]	*Gnathosia* Fischer von Waldheim, 1821
*Gonopus* Latreille [1834: 188]	*Gonopus* Latreille, 1829
*Hegeter* Latreille [1834: 186]	*Hegeter* Latreille, 1802
*Heliophilus* Dejean [1834: 191]	*Heliophilus* Dejean, 1821
*Heteroscelis* Latreille [1834: 187]	*Heteroscelis* Latreille, 1829
*Isocerus* Megerle [1834: 190]	*Isocerus* Dejean, 1821
*Laena* Megerle [1834: 184]	*Laena* Dejean, 1821
*Leptonychus* Chevrolat [1834: 177]	*Leptonychus* Chevrolat, 1833
*Machla* Herbst [1834: 187]	*Machla* Herbst, 1799
*Misolampus* Latreille [1834: 189]	*Misolampus* Latreille, 1807
*Moluris* Latreille [1834: 180]	*Moluris* Latreille, 1802
*Nyctelia* Latreille [1834: 186]	*Nyctelia* Latreille, 1825
*Nycterinus* Eschscholtz [1834: 189]	*Nycterinus* Eschscholtz, 1829
*Nyctoporis* Eschscholtz [1834: 183]	*Nyctoporis* Eschscholtz, 1831
*Nyctozoilus* Guérin [1834: 187]	*Nyctozoilus* Guérin-Méneville, 1831
*Opatrinus* Dejean [1834: 191]	*Opatrinus* Dejean, 1821
*Opatrum* Fabricius [1834: 193]	*Opatrum* Fabricius, 1775
*Oxura* Kirby [1834: 181]	*Oxura* Kirby, 1819
*Pedinus* Latreille [1834: 190]	*Pedinus* Latreille, 1797
*Philax* Megerle [1834: 192]	*Phylan* Dejean, 1821
*Physogaster* Latreille [1834: 180]	*Pimelia* Fabricius, 1775
*Pimelia* Fabricius [1834: 178]	*Physogaster* Lacordaire, 1830
*Platynotus* Fabricius [1834: 190]	*Platynotus* Fabricius, 1801
*Platyope* Fischer [1834: 180]	*Platyope* Fischer von Waldheim, 1820
*Platyscelis* Latreille [1834: 189]	*Platyscelis* Latreille, 1818
*Polypleurus* Eschscholtz [1834: 183]	*Polypleurus* Eschscholtz, 1831
*Praocis* Eschscholtz [1834: 180]	*Praocis* Eschscholtz, 1829
*Psammetichus* Latreille [1834: 183]	*Psammetichus* Latreille, 1829
*Scaurus* Fabricius [1834: 183]	*Scaurus* Fabricius, 1775
*Scotinus* Kirby [1834: 186]	*Scotinus* Kirby, 1819
*Scotobius* Germar [1834: 182]	*Scotobius* Germar, 1824
*Sepidium* Fabricius [1834: 181]	*Sepidium* Fabricius, 1775
*Tagenia* Latreille [1834: 183]	*Tagenia* Latreille, 1802
*Tagona* Fischer [1834: 188]	*Tagona* Fischer von Waldheim, 1820
*Tentyria* Latreille [1834: 184]	*Tentyria* Latreille, 1802
*Trachyderma* Latreille [1834: 179]	*Trachyderma* Latreille, 1829
*Trachynotus* Latreille [1834: 181]	*Trachynotus* Latreille, 1829
*Zophosis* Latreille [1834: 177]	*Zophosis* Latreille, 1802
**Hétéromères:** **Taxicornes**	
*Anisotoma* Fabricius [1834: 195]	*Anisotoma* Panzer, 1797
*Bolitophagus* Fabricius [1834: 195]	*Bolitophagus* Illiger, 1798
*Ceropria* De Laporte [1834: 198]	*Ceropria* Laporte & Brullé, 1831
*Cnodalon* Latreille [1834: 198]	*Cnodalon* Latreille, 1797
*Coelus* Eschscholtz [1834: 194]	*Coelus* Eschscholtz, 1829
*Corticus* Dejean [1834: 200]	*Corticus* Latreille, 1829
*Cossyphus* Fabricius [1834: 198]	*Cossyphus* Olivier, 1791
*Coxelus* Ziegler [1834: 201]	*Coxelus* Dejean, 1821
*Diaperis* Fabricius [1834: 197]	*Diaperis* Geoffroy, 1762
*Diodesma* Megerle [1834: 201]	*Diodesma* Latreille, 1829
*Eledona* Latreille [1834: 195]	*Eledona* Latreille, 1797
*Helaeus* Kirby [1834: 198]	*Heleus* Latreille, 1817
*Hemicera* De Laporte [1834: 197]	*Hemicera* Laporte & Brullé, 1831
*Hypophloeus* Fabricius [1834: 200]	*Hypophlaeus* Fabricius, 1790
*Leiodes* Latreille [1834: 195]	*Leiodes* Latreille, 1797
*Lithophilus* Megerle [1834: 195]	*Lithophilus* Frölich, 1799
*Neomida* Ziegler [1834: 197]	*Neomida* Latreille, 1829
*Nilio* Latreille [1834: 198]	*Nilio* Latreille, 1802
*Oplocephala* De Laporte [1834: 197]	*Hoplocephala* Laporte & Brullé, 1831
*Oopiestus* Chevrolat [1834: 199]	*Opiestus* Chevrolat, 1833
*Orthocerus* Latreille [1834: 200]	*Orthocerus* Latreille, 1797
*Pentaphyllus* Megerle [1834: 196]	*Pentaphyllus* Dejean, 1821
*Phaleria* Latreille [1834: 194]	*Phaleria* Latreille, 1802
*Platydema* De Laporte [1834: 196]	*Platydema* Laporte & Brullé, 1831
*Sarrotrium* Fabricius [1834: 200]	*Sarrotrium* Illiger, 1798
*Tetratoma* Fabricius [1834: 196]	*Tetratoma* Fabricius, 1790
*Trachyscelis* Latreille [1834: 194]	*Trachyscelis* Latreille, 1809
*Uloma* Megerle [1834: 199]	*Uloma* Dejean, 1821
**Hétéromères:** **Ténébrionites**	
*Blapida* Perty [1834: 207]	*Blapida* Perty, 1830
*Boros* Herbst [1834: 205]	*Boros* Herbst, 1797
*Calcar* Dejean [1834: 205]	*Calcar* Dejean, 1821
*Camaria* Encyclopédie [1834: 206]	*Camaria* Lepeletier & Audinet-Serville, 1828
*Campsia* Encyclopédie [1834: 207]	*Campsia* Lepeletier & Audinet-Serville, 1828
*Chiroscelis* De Lamarck [1834: 203]	*Chiroscelis* Lamarck, 1804
*Conopalpus* Gyllenhal [1834: 202]	*Conopalpus* Gyllenhal, 1810
*Dircaea* Fabricius [1834: 201]	*Dircaea* Fabricius, 1798
*Epitragus* Latreille [1834: 202]	*Epitragus* Latreille, 1802
*Eustrophus* Illiger [1834: 201]	*Eustrophus* Illiger, 1802
*Goniadera* Perty [1834: 206]	*Goniadera* Perty, 1832
*Hallomenus* Paykull [1834: 201]	*Hallomenus* Panzer, 1793
*Heterotarsus* Latreille [1834: 205]	*Heterotarsus* Latreille, 1829
*Hypulus* Paykull [1834: 202]	*Hypulus* Paykull, 1798
*Melandrya* Fabricius [1834: 202]	*Melandrya* Fabricius, 1801
*Mycetophagus* Fabricius [1834: 201]	*Mycetophagus* Fabricius, 1792
*Odontopus* Silbermann [1834: 203]	*Odontopus* Silbermann, 1833
*Orchesia* Latreille [1834: 201]	*Orchesia* Latreille, 1807
*Pytho* Fabricius [1834: 202]	*Pytho* Latreille, 1797
*Scotodes* Eschscholtz [1834: 202]	*Scotodes* Eschscholtz, 1818
*Serropalpus* Paykull [1834: 202]	*Serropalpus* Hellenius, 1786
*Tenebrio* Fabricius [1834: 205]	*Tenebrio* Linnaeus, 1758
*Toxicum* Latreille [1834: 205]	*Toxicum* Latreille, 1802
*Trogosita* Fabricius [1834: 205]	*Trogossita* Olivier, 1790
*Upis* Fabricius [1834: 204]	*Upis* Fabricius, 1792
**Hétéromères:** **Hélopiens**	
*Acropteron* Perty [1834: 212]	*Acropteron* Perty, 1832
*Adelium* Kirby [1834: 211]	*Adelium* Kirby, 1819
*Allecula* Fabricius [1834: 212]	*Allecula* Fabricius, 1801
*Amarygmus* Dalman [1834: 211]	*Amarygmus* Dalman, 1823
*Amphydora* Eschscholtz [1834: 210]	*Amphidora* Eschscholtz, 1829
*Cistela* Fabricius [1834: 213]	*Cistela* Fabricius, 1775
*Cyphonotus* Guérin [1834: 211]	*Cyphonotus* Guérin-Méneville, 1831
*Helops* Fabricius [1834: 208]	*Helops* Fabricius, 1775
*Lystronychus* Latreille [1834: 212]	*Lystronychus* Latreille, 1829
*Mycetochares* Latreille [1834: 213]	*Mycetochara* Berthold, 1827
*Mycetophila* Gyl. [1834: 213]	*Mycetophila* Gyllenhal, 1810
*Phymatisoma* De Laporte [1834: 207]	*Phymatisoma* Laporte & Brullé, 1831
*Prostenus* Latreille [1834: 212]	*Prostenus* Klug, 1829
*Sphaerotus* Kirby [1834: 211]	*Sphaerotus* Kirby, 1819
*Spheniscus* Kirby [1834: 207]	*Spheniscus* Kirby, 1819
*Stenochia* Kirby [1834: 210]	*Stenochia* Kirby, 1819
*Stenotrachelus* Latreille [1834: 211]	*Stenotrachelus* Berthold, 1827
*Strongylium* Kirby [1834: 208]	*Strongylium* Kirby, 1819
**Hétéromères:** **Trachélides**	
*Agnathus* Germar [1834: 216]	*Agnathus* Germar, 1818
*Anaspis* Geoffroy [1834: 220]	*Anaspis* Geoffroy, 1762
*Anthicus* Fabricius [1834: 216]	*Anthicus* Paykull, 1798
*Cissites* Latreille [1834: 220]	*Cissites* Latreille, 1804
*Dendroides* Latreille [1834: 216]	*Dendroides* Latreille, 1810
*Helodes* Fabricius [1834: 215]	*Helodes* Paykull, 1799
*Horia* Fabricius [1834: 220]	*Horia* Fabricius, 1787
*Lagria* Fabricius [1834: 215]	*Lagria* Fabricius, 1775
*Monocerus* Megerle [1834: 216]	*Monocerus* Villa & Villa, 1833
*Mordella* Fabricius [1834: 218]	*Mordella* Linnaeus, 1758
*Myodes* Latreille [1834: 218]	*Myodes* Latreille, 1818
*Pedilus* Fischer [1834: 215]	*Pedilus* Fischer von Waldheim, 1820
*Pelecotoma* Fischer [1834: 218]	*Pelecotoma* Fischer von Waldheim, 1808
*Pogonocerus* Fischer [1834: 216]	*Pogonocerus* Fischer von Waldheim, 1812
*Pyrochroa* Fabricius [1834: 215]	*Pyrochroa* Geoffroy, 1762
*Ripiphorus* Fabricius [1834: 218]	*Ripiphorus* Bosc, 1791
*Scraptia* Latreille [1834: 217]	*Scraptia* Latreille, 1807
*Statyra* Latreille [1834: 214]	*Statira* Berthold, 1827
*Steropes* Stéven [1834: 216]	*Steropes* Steven, 1806
*Symbius* Sundewall [1834: 217]	*Symbius* Sundevall, 1831
*Xylophilus* Bonelli [1834: 217]	*Xylophilus* Latreille, 1829
**Hétéromères:** **Vésicants**	
*Apalus* Fabricius [1834: 227]	*Apalus* Fabricius, 1775
*Cerocoma* Fabricius [1834: 221]	*Cerocoma* Geoffroy, 1762
*Decatoma* Dejean [1834: 221]	*Decatoma* Dejean, 1821
*Dices* Latreille [1834: 221]	*Dices* Dejean, 1821
*Hycleus* Latreille [1834: 221]	*Hycleus* Latreille, 1817
*Lydus* Megerle [1834: 223]	*Lydus* Dejean, 1821
*Lytta* Fabricius [1834: 224]	*Lytta* Fabricius, 1775
*Meloe* Fabricius [1834: 220]	*Meloe* Linnaeus, 1758
*Mylabris* Fabricius [1834: 222]	*Mylabris* Fabricius, 1775
*Nemognatha* Latreille [1834: 227]	*Nemognatha* Illiger, 1807
*Oenas* Latreille [1834: 224]	*Oenas* Latreille, 1802
*Sitaris* Latreille [1834: 227]	*Sitaris* Latreille, 1802
*Tetraonyx* Latreille [1834: 226]	*Tetraonyx* Latreille, 1809
*Zonitis* Fabricius [1834: 226]	*Zonitis* Fabricius, 1775
**Hétéromères:** **Sténélytres**	
*Calopus* Fabricius [1834: 227]	*Calopus* Fabricius, 1775
*Ditylus* Fischer [1834: 228]	*Ditylus* Fischer von Waldheim, 1817
*Dryops* Fabricius [1834: 229]	*Dryops* Fabricius, 1791
*Mycterus* Olivier [1834: 229]	*Mycterus* Clairville, 1798
*Nothus* Ziegler [1834: 227]	*Nothus* Olivier, 1811
*Oedemera* Olivier [1834: 229]	*Oedemera* Olivier, 1789
*Osphya* Illiger [1834: 227]	*Osphya* Illiger, 1807
*Rhinosimus* Latreille [1834: 230]	*Rhinosimus* Latreille, 1802
*Salpingus* Gyllenhal [1834: 229]	*Salpingus* Illiger, 1802
*Sparedrus* Megerle [1834: 227]	*Sparedrus* Dejean, 1821
*Stenostoma* Latreille [1834: 229]	*Stenostoma* Latreille, 1810
**Tétramères: Curculionites**	
*Acalles* Schönherr [1835: 296]	*Acalles* Schönherr, 1825
*Acallopistus* Schönherr [1835: 282]	*Acallopistus* Schönherr, 1826
*Acalyptus* Schönherr [1835: 281]	Acalyptus Schönherr, 1833
*Acantholophus* Mac Leay [1835: 265]	*Acantholophus* Boisduval, 1835 [**5**]
*Acorynus* Schönherr [1834: 234]	*Acorynus* Schönherr, 1833
*Agraphus* Schönherr [1835: 271]	*Agraphus* Say, 1831
*Alcides* Dalman [1835: 286]	*Alcides* Wiedemann, 1823
*Alophus* Schönherr [1835: 260]	*Alophus* Schönherr, 1826
*Amalus* Schönherr [1835: 280]	*Amalus* Schönherr, 1825
*Ambates* Schönherr [1835: 277]	*Embates* Chevrolat, 1833
*Amerhinus* Schönherr [1835: 286]	*Amerhinus* Schönherr, 1825
*Ameris* Germar [1835: 286]	*Ameris* Dejean, 1821
*Amblyrhinus* Schönherr [1835: 265]	*Amblyrhinus* Schönherr, 1826
*Amycterus* Dalman [1835: 266]	*Amycterus* Schönherr, 1823
*Anaemerus* Schönherr [1834: 254]	*Anaemerus* Schönherr, 1823
*Analcis* Schönherr [1835: 295]	*Analcis* Say, 1831
*Anchonus* Schönherr [1835: 283]	*Anchonus* Schönherr, 1825
*Anoplus* Schüppel [1835: 282]	*Anoplus* Germar, 1820
*Anthonomus* Germar [1835: 279]	*Anthonomus* Germar, 1817
*Anthribus* Fabricius [1834: 234]	*Anthribus* Geoffroy, 1762
*Apion* Herbst [1834: 240]	*Apion* Herbst, 1797
*Apoderus* Olivier [1834: 237]	*Apoderus* Olivier, 1807
*Apotomus* Schönherr [1834: 253]	*Apotomus* Schönherr, 1834 [**4**]
*Apsis* Germar [1835: 284]	*Apsis* Germar, 1820
*Araecerus* Schönherr [1834: 237]	*Araecerus* Schönherr, 1823
*Archarias* Dej. [1835: 285]	*Archarias* Dejean, 1821
*Arrhenodes* Stéven [1834: 242]	*Arrenodes* Schönherr, 1823
*Arthrostenus* Schönherr [1835: 284]	*Arthrostenus* Schönherr, 1826
*Artipus* Schönherr [1834: 254]	*Artipus* Sahlberg, 1823
*Astygus* Schönherr [1834: 254]	*Astycus* Schönherr, 1823
*Aterpus* Schönherr [1835: 259]	*Aterpus* Schönherr, 1826
*Attelabus* Linné [1834: 237]	*Attelabus* Linnaeus, 1758
*Auletes* Schönherr [1834: 239]	*Auletes* Schönherr, 1826
*Bagous* Germar [1835: 284]	*Bagous* Germar, 1817
*Balaninus* Germar [1835: 280]	*Balaninus* Germar, 1817
*Baridius* Schönherr [1835: 287]	*Baridius* Schönherr, 1825
*Baris* Germar [1835: 287]	*Baris* Germar, 1817
*Barynotus* Germar [1835: 261]	*Barynotus* Germar, 1817
*Belopherus* Schönherr [1834: 243]	*Belopherus* Schönherr, 1833
*Belorhynchus* Latreille [1834: 243]	*Belorhynchus* Berthold, 1827
*Belus* Schönherr [1834: 240]	*Belus* Schönherr, 1823
*Blosyrus* Schönherr [1834: 248]	*Blosyrus* Schönherr, 1823
*Bothynoderes* Schönherr [1835: 257]	*Bothynoderes* Schönherr, 1823
*Brachonyx* Schönherr [1835: 278]	*Brachonyx* Schönherr, 1825
*Brachycerus* Fabricius [1834: 245]	*Brachycerus* Olivier, 1789
*Brachyderes* Schönherr [1834: 249]	*Brachyderes* Schönherr, 1823
*Brachytarsus* Schönherr [1834: 237]	*Brachytarsus* Schönherr, 1823
*Bradybatus* Germar [1835: 278]	*Bradybatus* Germar, 1824
*Brentus* Fabricius [1834: 243]	*Brentus* Fabricius, 1787
*Bronchus* Germar [1834: 246]	*Bronchus* Germar, 1817
*Bruchela* Meg. [1834: 233]	*Bruchela* Dejean, 1821
*Bruchus* Fabricius [1834: 231]	*Bruchus* Linnaeus, 1767
*Bulbifer* Megerle [1835: 305]	*Bulbifer* Dejean, 1821
*Byrsops* Schönherr [1835: 264]	*Byrsops* Germar, 1829
*Calandra* Fabricius [1835: 303]	*Calandra* Clairville, 1798
*Callinotus* Schönherr [1835: 285]	*Callinotus* Schönherr, 1826
*Camarotus* Germar [1834: 237]	*Camarotus* Germar, 1833
*Camptorhinus* Schönherr [1835: 294]	*Camptorhinus* Schönherr, 1825
*Campylirhynchus* Megerle [1835: 299]	*Campylirhynchus* Dejean, 1821
*Carpophagus* Mac Leay [1834: 231]	*Carpophagus* Macleay, 1827
*Caryoborus* Schönherr [1834: 233]	*Caryoborus* Schönherr, 1833
*Cenchroma* Germar [1834: 254]	*Cenchroma* Dejean, 1821
*Centrinus* Schönherr [1835: 289]	*Centrinus* Schönherr, 1825
*Ceocephalus* Schönherr [1834: 244]	*Ceocephalus* Schönherr, 1833
*Cepurus* Schönherr [1835: 262]	*Cepurus* Schönherr, 1834
*Ceutorhynchus* Schüppel [1835: 298]	*Ceutorhynchus* Germar, 1824
*Cherrus* Dalman [1834: 247]	*Cherrus* Schönherr, 1826
*Chlorophanus* Dalman [1834: 253]	*Chlorophanus* Sahlberg, 1823
*Cholus* Germar [1835: 285]	*Cholus* Germar, 1824
*Chrysolopus* Germar [1835: 259]	*Chrysolopus* Germar, 1817
*Cionus* Clairville [1835: 301]	*Cionus* Clairville, 1798
*Claeoderes* Schönherr [1834: 244]	*Claeoderes* Schönherr, 1833
*Cleogonus* Schönherr [1835: 301]	*Cleogonus* Schönherr, 1825
*Cleonis* Megerle [1835: 257]	*Cleonis* Dejean, 1821
*Cleonus* Schönherr [1835: 257]	*Cleonus* Schönherr, 1826
*Cleopus* Megerle [1835: 302]	*Cleopus* Dejean, 1821
*Cneorhinus* Schönherr [1834: 248]	*Cneorhinus* Schönherr, 1823
*Coelosternus* Schönherr [1835: 294]	*Coelosternus* Schönherr, 1825
*Coniatus* Germar [1835: 264]	*Coniatus* Germar, 1817
*Coptorus* Schönherr [1835: 300]	*Copturus* Schönherr, 1825
*Corrhecerus* Schönherr [1834: 234]	*Corrhecerus* Schönherr, 1826
*Corryssomerus* Schönherr [1835: 280]	*Coryssomerus* Schönherr, 1825
*Cosmorhinus* Schönherr [1835: 267]	*Cosmorhinus* Schönherr, 1826
*Cossonus* Fabricius [1835: 305]	*Cossonus* Clairville, 1798
*Cratosomus* Schönherr [1835: 292]	*Cratosomus* Schönherr, 1825
*Cratopus* Dalman [1834: 253]	*Cratopus* Schönherr, 1823
*Cryptorhynchus* Illiger [1835: 292]	*Cryptorhynchus* Illiger, 1807
*Cyclomus* Schönherr [1835: 267]	*Cyclomus* Schönherr, 1823
*Cylas* Latreille [1834: 244]	*Cylas* Latreille, 1802
*Cylindrocerus* Schönherr [1835: 291]	*Cylindrocerus* Schönherr, 1826
*Cyphicerus* Schönherr [1835: 265]	*Cyphicerus* Schönherr, 1823
*Cyphus* Schönherr [1834: 250]	*Cyphus* Germar, 1824
*Cyrtomon* Schönherr [1835: 292]	*Cyrtomon* Schönherr, 1823
*Derelomus* Schönherr [1835: 287]	*Derelomus* Schönherr, 1825
*Diaprepes* Schönherr [1834: 252]	*Diaprepes* Schönherr, 1823
*Diodyrhynchus* Germar [1834: 239]	*Doydirhynchus* Dejean, 1821
*Dionychus* Germar [1835: 285]	*Dionychus* Germar, 1824
*Diorymerus* Schönherr [1835: 291]	*Diorymerus* Schönherr, 1825
*Dorytomus* Germar [1835: 277]	*Dorytomus* Germar, 1817
*Dryophthorus* Schüppel [1835: 305]	*Dryophthorus* Germar, 1824
*Eccoptus* Dej. [1835: 299]	*Eccoptus* Dejean, 1821
*Ellescus* Megerle [1835: 278]	*Ellescus* Dejean, 1821
*Elytrodon* Schönherr [1835: 271]	*Elytrodon* Schönherr, 1826
*Elytrurus* Boisduval [1835: 271]	*Elytrurus* Boisduval, 1835 [**5**]
*Endaeus* Schönherr [1835: 282]	*Endaeus* Schönherr, 1826
*Entimus* Germar [1834: 246]	*Entimus* Germar, 1817
*Entyus* Schönherr [1834: 256]	*Entyus* Schönherr, 1826
*Epicaerus* Schönherr [1835: 262]	*Epicaerus* Schönherr, 1834
*Episomus* Schönherr [1835: 266]	*Episomus* Schönherr, 1823
*Episus* Billberg [1834: 245]	*Episus* Bilberg, 1820
*Eremnus* Schönherr [1835: 267]	*Eremnus* Schönherr, 1823
*Erirhinus* Schönherr [1835: 277]	*Erirhinus* Schönherr, 1825
*Eucorynus* Schönherr [1834: 237]	*Eucorynus* Schönherr, 1823
*Euderes* Schönherr [1835: 284]	*Euderes* Schönherr, 1825
*Eudius* Schönherr [1834: 256]	*Eudius* Schönherr, 1834 [**4**]
*Eugnathus* Schönherr [1834: 255]	*Eugnathus* Schönherr, 1834 [**4**]
*Euparius* Schönherr [1834: 235]	*Euparius* Schönherr, 1823
*Eupholus* Boisduval [1835: 260]	*Eupholus* Boisduval, 1835 [**5**]
*Eurhinus* Illiger [1835: 291]	*Eurhinus* Illiger, 1807
*Eurhinus* Kirby [1834: 240]	*Eurhinus* Kirby, 1819
*Eurhynchus* Schönherr [1834: 240]	*Eurhynchus* Schönherr, 1833
*Eurylobus* Schönherr [1835: 260]	*Eurylobus* Schönherr, 1826
*Euscelus* Germar [1834: 238]	*Euscelus* Schönherr, 1833
*Eusomus* Germar [1834: 249]	*Eusomus* Germar, 1824
*Eustales* Germar [1834: 251]	*Eustalis* Germar, 1824
*Eutrachelus* Latreille [1834: 243]	*Eutrachelus* Berthold, 1827
*Exophthalmus* Schönherr [1834: 252]	*Exophthalmus* Schönherr, 1823
*Falciger* Meg. [1835: 298]	*Falciger* Dejean, 1821
*Gasterocercus* De Laporte [1835: 295]	*Gasterocercus* Laporte & Brullé, 1828
*Gastrodus* Megerle [1835: 261]	*Gastrodus* Dejean, 1821
*Geonemus* Schönherr [1835: 260]	*Geonemus* Schönherr, 1833
*Gonipterus* Schönherr [1834: 246]	*Gonipterus* Schönherr, 1833
*Graphorhinus* Schönherr [1834: 247]	*Graphorhinus* Say, 1831
*Gronops* Schönherr [1835: 259]	*Gronops* Schönherr, 1823
*Grypidius* Schönherr [1835: 278]	*Grypidius* Schönherr, 1826
*Gymnaetron* Schönherr [1835: 302]	*Gymnetron* Schönherr, 1825
*Gymnognathus* Schönherr [1834: 236]	*Gymnognathus* Schönherr, 1826
*Heilipus* Germar [1835: 274]	*Heilipus* Germar, 1824
*Heomus* Schönherr [1835: 273]	*Ileomus* Schönherr, 1823
*Herpysticus* Germar [1834: 248]	*Herpisticus* Germar, 1824
*Hipporhinus* Schönherr [1834: 246]	*Hipporhinus* Schönherr, 1823
*Homalonotus* Schönherr [1835: 285]	*Homalinotus* Sahlberg, 1825
*Hydronomus* Schönherr [1835: 278]	*Hydronomus* Schönherr, 1825
*Hylobius* Germar [1835: 262]	*Hylobius* Germar, 1817
*Hypera* Germar [1835: 263]	*Hypera* Germar, 1817
*Hyphantus* Germar [1835: 271]	*Hyphantus* Germar, 1824
*Hypomeces* Schönherr [1834: 254]	*Hypomeces* Schönherr, 1823
*Hypsonotus* Germar [1835: 259]	*Hypsonotus* Germar, 1824
*Ithycerus* Dalman [1834: 240]	*Ithycerus* Schönherr, 1823
*Laemosaccus* Schönherr [1835: 286]	*Laemosaccus* Schönherr, 1823
*Lagostomus* Schönherr [1834: 250]	*Lagostomus* Schönherr, 1833
*Larinus* Schüppel [1835: 273]	*Larinus* Dejean, 1821
*Leptops* Schönherr [1835: 261]	*Leptops* Schönherr, 1834
*Leptosomus* Schönherr [1835: 257]	*Leptosomus* Schönherr, 1823
*Lepyrus* Germar [1835: 262]	*Lepyrus* Germar, 1817
*Liophloeus* Germar [1835: 261]	*Liophloeus* Germar, 1817
*Liparus* Oliv. [1835: 262]	*Liparus* Olivier, 1807
*Lissorhinus* Schönherr [1834: 254]	*Lissorhinus* Schönherr, 1826
*Listroderes* Schönherr [1835: 260]	*Listroderes* Schönherr, 1826
*Lithinus* Klug [1834: 248]	*Lithinus* Klug, 1833
*Litocerus* Schönherr [1834: 234]	*Litocerus* Schönherr, 1833
*Litomerus* Schönherr [1835: 285]	*Litomerus* Schönherr, 1833
*Lixus* Fabricius [1835: 271]	*Lixus* Fabricius, 1801
*Loncophorus* Chevrolat [1835: 280]	*Loncophorus* Chevrolat, 1832
*Lophotus* Schönherr [1835: 261]	*Lophotus* Schönherr, 1834
*Lordops* Schönherr [1835: 259]	*Lordops* Schönherr, 1823
*Lyprus* Schönherr [1835: 284]	*Lyprus* Schönherr, 1826
*Macrocorynus* Schönherr [1835: 265]	*Macrocorynus* Schönherr, 1823
*Macromerus* Schönherr [1835: 294]	*Macromerus* Schönherr, 1825
*Madarus* Schönherr [1835: 287]	*Madarus* Schönherr, 1825
*Mecinus* Germar [1835: 302]	*Mecinus* Germar, 1821
*Mecocerus* Schönherr [1834: 234]	*Mecocerus* Schönherr, 1833
*Mecocorynus* Schönherr [1835: 296]	*Mecocorynus* Schönherr, 1825
*Mecopus* Dalman [1835: 284]	*Mecopus* Schönherr, 1825
*Meleus* Meg. [1835: 263]	*Meleus* Dejean, 1821
*Metallites* Schönherr [1834: 256]	*Metallites* Germar, 1824
*Microcerus* Gyllenhal [1834: 246]	*Microcerus* Schönherr, 1833
*Minyops* Schönherr [1835: 261]	*Minyops* Schönherr, 1823
*Molytes* Schönherr [1835: 262]	*Molytes* Schönherr, 1823
*Mononychus* Schüppel [1835: 299]	*Mononychus* Germar, 1824
*Myllocerus* Schönherr [1835: 264]	*Myllocerus* Schönherr, 1823
*Myorhinus* Schönherr [1835: 284]	*Myorhinus* Schönherr, 1826
*Myrmacicelus* Chevrolat [1834: 244]	*Myrmacicelus* Chevrolat, 1833
*Nanodes* Schönherr [1835: 302]	*Nanodes* Schönherr, 1825
*Naupactus* Megerle [1834: 249]	*Naupactus* Dejean, 1821
*Nemocephalus* Latreille [1834: 244]	*Nemocephalus* Guérin-Méneville, 1827
*Nerthops* Schönherr [1835: 274]	*Nerthops* Schönherr, 1826
*Nettarhinus* Schönherr [1835: 286]	*Nettarhinus* Schönherr, 1826
*Ocladius* Schönherr [1835: 301]	*Ocladius* Schönherr, 1825
*Omias* Germar [1835: 266]	*Omias* Germar, 1817
*Orchestes* Illiger [1835: 283]	*Orchestes* Illiger, 1798
*Orobites* Schönherr [1835: 301]	*Orobites* Schönherr, 1833
*Orobitis* Germar [1835: 301]	*Orobitis* Germar, 1817
*Orthorhinus* Schönherr [1835: 276]	*Orthorhinus* Schönherr, 1825
*Orthorhynchus* Mac Leay [1834: 240]	*Orthorhynchus* Kirby, 1819
*Otidocephalus* Chevrolat [1835: 279]	*Otidocephalus* Chevrolat, 1832
*Otiorhynchus* Germar [1835: 267]	*Otiorhynchus* Germar, 1822
*Oxyderces* Schönherr [1834: 251]	*Oxyderces* Schönherr, 1823
*Oxyrhynchus* Schönherr [1834: 245]	*Oxyrhynchus* Schönherr, 1823
*Pachnaeus* Schönherr [1834: 253]	*Pachnaeus* Schönherr, 1826
*Pachycerus* Gyllenhal [1835: 258]	*Pachycerus* Schönherr, 1823
*Pachygaster* Germar [1835: 267]	*Pachygaster* Germar, 1817
*Pachymerus* Latreille [1834: 233]	*Pachymerus* Thunberg, 1805
*Pachyrhynchus* Germar [1834: 247]	*Pachyrhynchus* Germar, 1824
*Pandeleteius* Schönherr [1834: 255]	*Pandeleteius* Schönherr, 1834 [**4**]
*Paramecops* Schönherr [1835: 276]	*Paramecops* Schönherr, 1825
*Penestes* Schönherr [1835: 278]	*Penestes* Schönherr, 1825
*Peritelus* Germar [1835: 267]	*Peritelus* Germar, 1824
*Phaedropus* Schönherr [1834: 251]	*Phaedropus* Schönherr, 1823
*Phaenithon* Schönherr [1834: 236]	*Phaenithon* Schönherr, 1826
*Phalidura* Mac Leay [1835: 266]	*Phalidura* Fischer von Waldheim, 1823
*Phloeotragus* Schönherr [1834: 234]	*Phloeotragus* Schönherr, 1823
*Phlyctinus* Schönherr [1835: 267]	*Phlyctinus* Schönherr, 1826
*Pholicodes* Schönherr [1835: 267]	*Pholicodes* Schönherr, 1826
*Phyllobius* Schönherr [1835: 265]	*Phyllobius* Germar, 1824
*Phytobius* Schmidt [1835: 282]^3^	*Phytobius* Schönherr, 1833
*Phytonomus* Schönherr [1835: 263]	*Phytonomus* Schönherr, 1823
*Phytoscaphus* Schönherr [1835: 271]	*Phytoscaphus* Schönherr, 1826
*Piazorus* Schönherr [1835: 300]	*Piazorus* Schönherr, 1826
*Pinarus* Schönherr [1835: 301]	*Pinarus* Schönherr, 1826
*Pissodes* Germar [1835: 276]	*Pissodes* Germar, 1817
*Platyomus* Schönherr [1834: 251]	*Platyomus* Sahlberg, 1823
*Platyonyx* Schönherr [1835: 291]	*Platyonyx* Schönherr, 1826
*Platyrhinus* Clairville [1834: 236]	*Platyrhinus* Clairville, 1798
*Plinthus* Germar [1835: 263]	*Plinthus* Germar, 1817
*Polycomus* Schönherr [1834: 252]	*Polycomus* Schönherr, 1833
*Polydacrys* Schönherr [1834: 255]	*Polydacrys* Schönherr, 1834 [**4**]
*Polyderces* Schönherr [1835: 285]	*Polyderces* Schönherr, 1833
*Polydrosus* Schönherr [1834: 255]	*Polydrosus* Schönherr, 1823
*Polydrusus* Germar [1834: 255]	*Polydrusus* Germar, 1817
*Prepodes* Schönherr [1834: 252]	*Prepodes* Schönherr, 1823
*Prionopus* Dalman [1835: 302]	*Prionopus* Dalman, 1826
*Promecops* Schönherr [1835: 257]	*Promecops* Sahlberg, 1823
*Prostomus* Dalman [1834: 247]	*Prostomus* Schönherr, 1823
*Protenomus* Schönherr [1834: 254]	*Protenomus* Schönherr, 1826
*Prypnus* Schönherr [1834: 247]	*Prypnus* Schönherr, 1823
*Psalidium* Illiger [1834: 247]	*Psallidium* Herbst, 1795
*Pterocolus* Schönherr [1834: 239]	*Pterocolus* Say, 1831
*Ptilopus* Schönherr [1834: 252]	*Ptilopus* Schönherr, 1823
*Ptochus* Schönherr [1835: 266]	*Ptochus* Schönherr, 1826
*Ptychoderes* Schönherr [1834: 234]	*Ptychoderes* Schönherr, 1823
*Ramphus* Clairville [1834: 242]	*Ramphus* Clairville, 1798
*Rembus* Germar [1834: 256]	*Rembus* Germar, 1824
*Rhigus* Dalman [1834: 246]	*Rhigus* Schönherr, 1823
*Rhina* Latreille [1835: 302]	*Rhina* Latreille, 1802
*Rhinaria* Kirby [1835: 282]	*Rhinaria* Kirby, 1819
*Rhinastus* Schönherr [1835: 284]	*Rhinastus* Schönherr, 1825
*Rhinobatus* Germar [1835: 273]	*Rhinobatus* Germar, 1817
*Rhinocyllus* Germar [1835: 274]	*Rhinocyllus* Germar, 1817
*Rhinodes* Schönherr [1835: 276]	*Rhinodes* Dejean, 1821
*Rhinomacer* Fabricius [1834: 239]	*Rhinomacer* Olivier, 1807
*Rhinotia* Kirby [1834: 240]	*Rhinotia* Kirby, 1819
*Rhynchaenus* Dej. [1835: 277]	*Rhynchaenus* Clairville, 1798
*Rhynchites* Herbst [1834: 238]	*Rhynchites* Schneider, 1791
*Rhynchophorus* Herbst [1835: 303]	*Rhynchophorus* Herbst, 1795
*Rhyncolus* Creutzer [1835: 305]	*Rhyncolus* Germar, 1817
*Rhytirhinus* Schönherr [1835: 264]	*Rhythirrhinus* Schönherr, 1823
*Sciaphilus* Schönherr [1834: 248]	*Sciaphilus* Schönherr, 1823
*Sciobius* Schönherr [1835: 267]	*Sciobius* Schönherr, 1823
*Scytropus* Schönherr [1834: 256]	*Scytropus* Schönherr, 1826
*Sibinia* Germar [1835: 281]	*Sibinia* Germar, 1817
*Sibynes* Schönherr [1835: 281]	*Sibynes* Schönherr, 1825
*Siderodactylus* Schönherr [1834: 255]	*Siderodactylus* Schönherr, 1834 [**4**]
*Sipalus* Schönherr [1835: 303]	*Sipalus* Schönherr, 1825
*Sitona* Germar [1834: 255]	*Sitona* Germar, 1817
*Solenopus* Schönherr [1835: 286]	*Solenopus* Schönherr, 1825
*Spermophagus* Stéven [1834: 233]	*Spermophagus* Schönherr, 1833
*Sphaerogaster* Dejean [1834: 247]	*Sphaerogaster* Germar, 1833^4^
*Sphaeromus* Schönherr [1835: 267]	*Sphaeromus* Schönherr, 1834
*Stenocerus* Schönherr [1834: 236]	*Stenocerus* Schönherr, 1826
*Stenocorynus* Schönherr [1835: 261]	*Stenocorynus* Schönherr, 1823
*Sternechus* Schönherr [1835: 282]	*Sternechus* Schönherr, 1826
*Styphlus* Schönherr [1835: 283]	*Styphlus* Schönherr, 1826
*Syzygops* Schönherr [1834: 247]	*Syzygops* Schönherr, 1826
*Tachigonus* Schönherr [1834: 242]	*Tachygonus* Schönherr, 1833
*Tanymecus* Germar [1834: 254]	*Tanymecus* Germar, 1817
*Tanyrhynchus* Schönherr [1835: 284]	*Tanyrhynchus* Schönherr, 1826
*Tanysphyrus* Germar [1835: 262]	*Tanysphyrus* Germar, 1817
*Taphroderes* Schönherr [1834: 244]	*Taphroderes* Schönherr, 1823
*Tapinotus* Schönherr [1835: 298]	*Tapeinotus* Schönherr, 1826
*Thamnophilus* Schönherr [1835: 276]	*Thamnophilus* Schönherr, 1823
*Thylacites* Germar [1834: 247]	*Thylacites* Germar, 1817
*Trachodes* Schüppel [1835: 283]	*Trachodes* Germar, 1824
*Trachyphloeus* Germar [1835: 266]	*Trachyphloeus* Germar, 1817
*Tropideres* Schönherr [1834: 235]	*Tropideres* Schönherr, 1823
*Tubicenus* Dejean [1834: 239]	*Tubicenus* Latreille, 1828
*Tychius* Germar [1835: 280]	*Tychius* Germar, 1817
*Tyloderes* Schönherr [1835: 270]	*Tyloderes* Schönherr, 1826
*Tylodes* Schönherr [1835: 295]	*Tylodes* Sahlberg, 1823
*Tylomus* Schönherr [1835: 282]	*Tylomus* Schönherr, 1826
*Ulocerus* Dalman [1834: 245]	*Ulocerus* Schönherr, 1823
*Urodon* Schönherr [1834: 233]	*Urodon* Schönherr, 1823
*Uropterus* Latreille [1834: 244]	*Uroptera* Latreille, 1828
*Xylinades* Latreille [1834: 237]	*Xylinades* Latreille, 1828
*Zygops* Schönherr [1835: 299]	*Zygops* Schönherr, 1825
**Tétramères: Xylophages**	
*Apate* Fabricius [1835: 308]	*Apate* Fabricius, 1775
*Biphyllus* Dejean [1835: 311]	*Biphyllus* Dejean, 1821
*Bitoma* Herbst [1835: 312]	*Bitoma* Herbst, 1793
*Bostrichus* Fabricius [1835: 307]	*Bostrichus* Geoffroy, 1762
*Brontes* Fabricius [1835: 315]	*Brontes* Fabricius, 1801
*Camptocerus* Dejean [1835: 307]	*Camptocerus* Dejean, 1821
*Cerylon* Latreille [1835: 311]	*Cerylon* Latreille, 1802
*Cis* Latreille [1835: 310]	*Cis* Latreille, 1797
*Colydium* Fabricius [1835: 313]	*Colydium* Fabricius, 1792
*Cucujus* Fabricius [1835: 315]	*Cucujus* Fabricius, 1775
*Dendrophagus* Gyllenhal [1835: 315]	*Dendrophagus* Schönherr, 1809
*Hylesinus* Fabricius [1835: 306]	*Hylesinus* Fabricius, 1801
*Hylurgus* Latreille [1835: 306]	*Hylurgus* Latreille, 1806
*Latridius* Herbst [1835: 310]	*Latridius* Herbst, 1793
*Lyctus* Fabricius [1835: 313]	*Lyctus* Fabricius, 1792
*Megagnathus* Megerle [1835: 314]	*Megagnathus* Dejean, 1821
*Meryx* Latreille [1835: 313]	*Meryx* Latreille, 1802
*Monotoma* Herbst [1835: 312]	*Monotoma* Herbst, 1793
*Mycetophagus* Fabricius [1835: 311]	*Mycetophagus* Fabricius, 1792
*Myrmechixenus* Chevrolat [1835: 312]	*Myrmechixenus* Chevrolat, 1835 [**9**]
*Nemosoma* Desmarest [1835: 313]	*Nemozoma* Latreille, 1804
*Passandra* Dalman [1835: 314]	*Passandra* Dalman, 1817
*Paussus* Linné [1835: 308]	*Paussus* Linnaeus, 1775
*Phloiotribus* Latreille [1835: 306]	*Phloeotribus* Latreille, 1797
*Platypus* Herbst [1835: 308]	*Platypus* Herbst, 1793
*Prostomis* Latreille [1835: 314]	*Prostomis* Latreille, 1819
*Psammaechus* Boudier [1835: 311]	*Psammoechus* Latreille, 1829
*Psoa* Fabricius [1835: 309]	*Psoa* Herbst, 1797
*Rhyzophagus* Herbst [1835: 312]	*Rhizophagus* Herbst, 1793
*Scolytus* Geoffroy [1835: 307]	*Scolytus* Geoffroy, 1762
*Silvanus* Latreille [1835: 313]	*Silvanus* Latreille, 1804
*Sphindus* Megerle [1835: 310]	*Sphindus* Dejean, 1821
*Synchita* Hellwig [1835: 311]	*Synchita* Hellwig, 1792
*Triphyllus* Megerle [1835: 311]	*Triphyllus* Dejean, 1821
*Trogosita* Fabricius [1835: 314]	*Trogossita* Olivier, 1790
**Tétramères: Longicornes**	
*Acanthocinus* Megerle [1835: 336]	*Acanthocinus* Dejean, 1821
*Acanthoderus* Serville [1835: 336]	*Acanthoderes* Audinet-Serville, 1835 [**7**]
*Acanthophorus* Serville [1835: 316]	*Acanthophorus* Audinet-Serville, 1832
*Achryson* Serville [1835: 328]	*Achryson* Audinet-Serville, 1834
*Acrocinus* Illiger [1835: 335]	*Acrocinus* Illiger, 1806
*Aedilis* Serville [1835: 337]	*Aedilis* Audinet-Serville, 1835 [**7**]
*Aedopeza* Serville [1835: 338]	*Oedopeza* Audinet-Serville, 1835 [**7**]
*Aegosoma* Serville [1835: 318]	*Aegosoma* Audinet-Serville, 1832
*Agapanthia* Serville [1835: 353]	*Agapanthia* Audinet-Serville, 1835 [**7**]
*Allocerus* Serville [1835: 319]	*Allocerus* Lacordaire, 1830
*Amphidesmus* Eschscholtz [1835: 321]	*Amphidesmus* Audinet-Serville, 1834
*Anacanthus* Serville [1835: 317]	*Anacanthus* Audinet-Serville, 1832
*Anacolus* Latreille [1835: 319]	*Anacolus* Berthold, 1827
*Ancistrotus* Serville [1835: 316]	*Ancistrotus* Audinet-Serville, 1832
*Ancylocera* Serville [1835: 333]	*Ancylocera* Audinet-Serville, 1834
*Anisocerus* Serville [1835: 335]	*Anisocerus* Lacordaire, 1830
*Anisopus* Serville [1835: 338]	*Anisopus* Audinet-Serville, 1835 [**7**]
*Anoplistes* Serville [1835: 323]	*Anoplistes* Audinet-Serville, 1834
*Apomecyna* Dejean [1835: 347]	*Apomecyna* Dejean, 1821
*Aromia* Serville [1835: 323]	*Aromia* Audinet-Serville, 1834
*Asemum* Eschscholtz [1835: 329]	*Asemum* Eschscholtz, 1830
*Aulacopus* Serville [1835: 318]	*Aulacopus* Audinet-Serville, 1832
*Callia* Serville [1835: 347]	*Callia* Audinet-Serville, 1835 [**7**]
*Callichroma* Latreille [1835: 323]	*Callichroma* Latreille, 1816
*Callidium* Fabricius [1835: 329]	*Callidium* Fabricius, 1775
*Callipogon* Serville [1835: 317]	*Callipogon* Audinet-Serville, 1832
*Calocomus* Serville [1835: 319]	*Calocomus* Audinet-Serville, 1832
*Cartallum* Megerle [1835: 332]	*Certallum* Dejean, 1821
*Ceragenia* Serville [1835: 321]	*Ceragenia* Audinet-Serville, 1834
*Cerasphorus* Serville [1835: 326]	*Cerasphorus* Audinet-Serville, 1834
*Ceratites* Serville [1835: 343]	*Ceratites* Audinet-Serville, 1835 [**7**]
*Ceroctenus* Dejean [1835: 319]	*Ceroctenus* Audinet-Serville, 1832
*Ceroplesis* Serville [1835: 342]	*Ceroplesis* Audinet-Serville, 1835 [**7**]
*Chlorida* Serville [1835: 321]	*Chlorida* Audinet-Serville, 1834
*Chrysoprasis* Serville [1835: 325]	*Chrysoprasis* Audinet-Serville, 1834
*Closterus* Serville [1835: 318]	*Closterus* Audinet-Serville, 1832
*Clytus* Fabricius [1835: 330]	*Clytus* Laicharting, 1784
*Colobothea* Dejean [1835: 349]	*Colobothea* Lepeletier & Audinet-Serville, 1825
*Colobus* Serville [1835: 324]	*Colobus* Audinet-Serville, 1834
*Colpoderus* Serville [1835: 317]	*Colpoderus* Audinet-Serville, 1832
*Cometes* Encyclopédie [1835: 353]	*Cometes* Lepeletier & Audinet-Serville, 1828
*Compsocerus* Serville [1835: 324]	*Compsocerus* Lacordaire, 1830
*Compsosoma* Serville [1835: 343]	*Compsosoma* Lacordaire, 1830
*Coptops* Serville [1835: 344]	*Coptops* Audinet-Serville, 1835 [**7**]
*Cordylomera* Serville [1835: 327]	*Cordylomera* Audinet-Serville, 1834
*Coremia* Serville [1835: 325]	*Coremia* Audinet-Serville, 1834
*Cosmisoma* Serville [1835: 325]	*Cosmisoma* Audinet-Serville, 1834
*Criodion* Serville [1835: 322]	*Criodion* Audinet-Serville, 1834
*Crossotus* Serville [1835: 344]	*Crossotus* Audinet-Serville, 1835 [**7**]
*Cryptobias* Dupont [1835: 320]	*Cryptobias* Audinet-Serville, 1834
*Cryptocranium* Serville [1835: 347]	*Cryptocranium* Lacordaire, 1830
*Ctenoscelis* Serville [1835: 316]	*Ctenoscelis* Audinet-Serville, 1832
*Cycnoderus* Serville [1835: 333]	*Cycnoderus* Audinet-Serville, 1834
*Dadoychus* Chevrolat [1835: 352]	*Dadoychus* Chevrolat, 1833
*Deilus* Serville [1835: 333]	*Deilus* Audinet-Serville, 1834
*Deltaspis* Serville [1835: 323]	*Deltaspis* Audinet-Serville, 1834
*Dendrobias* Dupont [1835: 320]	*Dendrobias* Audinet-Serville, 1834
*Derobrachus* Dejean [1835: 318]	*Derobrachus* Audinet-Serville, 1832
*Desmiphora* Serville [1835: 340]	*Desmiphora* Audinet-Serville, 1835 [**7**]
*Desmocerus* Dejean [1835: 353]	*Desmocerus* Dejean, 1821
*Desmoderus* Dejean [1835: 320]	*Desmoderus* Audinet-Serville, 1834
*Disaulax* Serville [1835: 325]	*Disaulax* Audinet-Serville, 1834
*Distenia* Encyclopédie [1835: 353]	*Distenia* Lepeletier & Audinet-Serville, 1828
*Distichocera* Kirby [1835: 319]	*Distichocera* Kirby, 1819
*Dorcacerus* Dejean [1835: 320]	*Dorcacerus* Dejean, 1821
*Dorcadion* Dalman [1835: 346]	*Dorcadion* Dalman, 1817
*Dorcasomus* Dejean [1835: 319]	*Dorcasomus* Audinet-Serville, 1834
*Dryoctenes* Serville [1835: 335]	*Dryoctenes* Audinet-Serville, 1835 [**7**]
*Eburia* Serville [1835: 326]	*Eburia* Lacordaire, 1830
*Elaphidion* Serville [1835: 327]	*Elaphidion* Audinet-Serville, 1834
*Enoplocerus* Serville [1835: 316]	*Enoplocerus* Audinet-Serville, 1832
*Ergates* Serville [1835: 316]	*Ergates* Audinet-Serville, 1832
*Eriphus* Serville [1835: 325]	*Eriphus* Audinet-Serville, 1834
*Eudesmus* Serville [1835: 345]	*Eudesmus* Audinet-Serville, 1835 [**7**]
*Euporus* Serville [1835: 333]	*Euporus* Audinet-Serville, 1834
*Eurycephalus* Gray [1835: 340]	*Eurycephalus* Gray, 1832
*Eurymerus* Serville [1835: 326]	*Eurymerus* Audinet-Serville, 1834
*Euryptera* Encyclopédie [1835: 354]	*Euryptera* Lepeletier & Audinet-Serville, 1828
*Gerania* Serville [1835: 348]	*Gerania* Audinet-Serville, 1835 [**7**]
*Gnoma* Fabricius [1835: 345]	*Gnoma* Fabricius, 1801
*Gracilia* Serville [1835: 332]	*Gracilia* Audinet-Serville, 1834
*Hammaticherus* Megerle [1835: 322]	*Hamaticherus* Dejean, 1821
*Hemilophus* Serville [1835: 352]	*Hemilophus* Audinet-Serville, 1835 [**7**]
*Hippopsis* Serville [1835: 349]	*Hippopsis* Lepeletier & Audinet-Serville, 1825
*Hoplideres* Serville [1835: 316]	*Hoplideres* Audinet-Serville, 1834
*Hylotrupes* Serville [1835: 329]	*Hylotrupes* Audinet-Serville, 1834
*Hypsioma* Serville [1835: 343]	*Hypsioma* Audinet-Serville, 1835 [**7**]
*Ibidion* Serville [1835: 332]	*Ibidion* Audinet-Serville, 1834
*Icthyosomus* Dejean [1835: 327]	*Icthyosoma* Boisduval, 1835^5^ [**5**]
*Ionthodes* Serville [1835: 323]	*Jonthodes* Audinet-Serville, 1834
*Leiopus* Serville [1835: 339]	*Leiopus* Audinet-Serville, 1835 [**7**]
*Leptocera* Dejean [1835: 335]	*Leptocera* Dejean, 1821
*Leptura* Fabricius [1835: 355]	*Leptura* Linnaeus, 1758
*Lissonotus* Dalman [1835: 319]	*Lissonotus* Dalman, 1817
*Listroptera* Serville [1835: 333]	*Listroptera* Audinet-Serville, 1834
*Litopus* Serville [1835: 324]	*Litopus* Audinet-Serville, 1834
*Lophonocerus* Latreille [1835: 321]	*Lophonocerus* Latreille, 1829
*Macrodontia* Serville [1835: 316]	*Macrodontia* Lacordaire, 1830
*Macrotoma* Dejean [1835: 317]	*Macrotoma* Audinet-Serville, 1832
*Malacopterus* Serville [1835: 326]	*Malacopterus* Audinet-Serville, 1834
*Mallaspis* Serville [1835: 318]	*Mallaspis* Audinet-Serville, 1832
*Mallocera* Serville [1835: 326]	*Mallocera* Audinet-Serville, 1834
*Mallodon* Serville [1835: 317]	*Mallodon* Lacordaire, 1830
*Mallosoma* Serville [1835: 330]	*Mallosoma* Audinet-Serville, 1834
*Megabasis* Serville [1835: 335]	*Megabasis* Audinet-Serville, 1835 [**7**]
*Megacera* Serville [1835: 349]	*Megacera* Audinet-Serville, 1835 [**7**]
*Megaderus* Dejean [1835: 319]	*Megaderus* Dejean, 1821
*Megopis* Dejean [1835: 318]	*Megopis* Audinet-Serville, 1832
*Meroscelisus* Serville [1835: 318]	*Meroscelisus* Audinet-Serville, 1832
*Mesosa* Megerle [1835: 345]	*Mesosa* Latreille, 1829
*Metopocoelus* Dejean [1835: 319]	*Metopocoilus* Audinet-Serville, 1832
*Microplia* Serville [1835: 339]	*Microplia* Audinet-Serville, 1835 [**7**]
*Molorchus* Fabricius [1835: 334]	*Molorchus* Fabricius, 1792
*Monochamus* Dejean [1835: 340]	*Monochamus* Dejean, 1821
*Monodesmus* Dejean [1835: 318]	*Monodesmus* Audinet-Serville, 1832
*Morimus* Serville [1835: 342]	*Morimus* Brullé, 1832
*Obrium* Megerle [1835: 332]	*Obrium* Dejean, 1821
*Odontocera* Serville [1835: 334]	*Odontocera* Audinet-Serville, 1834
*Omacantha* Serville [1835: 341]	*Omacantha* Audinet-Serville, 1835 [**7**]
*Oncideres* Serville [1835: 343]	*Oncideres* Lacordaire, 1830
*Onychocerus* Serville [1835: 335]	*Onychocerus* Lacordaire, 1830
*Oregostoma* Serville [1835: 334]	*Oregostoma* Audinet-Serville, 1834
*Oreodera* Serville [1835: 335]	*Oreodera* Audinet-Serville, 1835 [**7**]
*Orthomegas* Serville [1835: 317]	*Orthomegas* Audinet-Serville, 1832
*Orthosoma* Dejean [1835: 317]	*Orthosoma* Audinet-Serville, 1832
*Orthostoma* Serville [1835: 324]	*Orthostoma* Lacordaire, 1830
*Oxymerus* Solier [1835: 320]	*Oxymerus* Audinet-Serville, 1834
*Ozodes* Serville [1835: 333]	*Ozodes* Audinet-Serville, 1834
*Pachypeza* Serville [1835: 349]	*Pachypeza* Audinet-Serville, 1835 [**7**]
*Pachyta* Megerle [1835: 354]	*Pachyta* Dejean, 1821
*Pachyteria* Serville [1835: 323]	*Pachyteria* Audinet-Serville, 1834
*Parandra* Latreille [1835: 315]	*Parandra* Latreille, 1802
*Parmena* Megerle [1835: 347]	*Parmena* Dejean, 1821
*Phaenicocerus* Latreille [1835: 321]	*Phaenicocerus* Latreille, 1829
*Phoebe* Serville [1835: 352]	*Phoebe* Audinet-Serville, 1835 [**7**]
*Piezocera* Serville [1835: 332]	*Piezocera* Audinet-Serville, 1834
*Platygnathus* Dejean [1835: 317]	*Platygnathus* Audinet-Serville, 1832
*Poecilosoma* Serville [1835: 319]	*Poekilosoma* Audinet-Serville, 1832
*Pogonocherus* Megerle [1835: 339]	*Pogonocherus* Dejean, 1821
*Polyarthron* Serville [1835: 318]	*Polyarthron* Audinet-Serville, 1832
*Polyoza* Serville [1835: 318]	*Polyoza* Audinet-Serville, 1832
*Polyrhaphis* Serville [1835: 335]	*Polyrhaphis* Audinet-Serville, 1835 [**7**]
*Polyschisis* Serville [1835: 323]	*Polyschisis* Audinet-Serville, 1834
*Prionus* Geoffroy [1835: 318]	*Prionus* Geoffroy, 1762
*Prodontia* Serville [1835: 321]	*Prodontia* Audinet-Serville, 1834
*Promeces* Serville [1835: 324]	*Promeces* Audinet-Serville, 1834
*Ptericoptus* Serville [1835: 348]	*Ptericoptus* Lacordaire, 1830
*Pteroplius* Serville [1835: 341]	*Pteroplius* Lacordaire, 1830
*Ptychodes* Chevrolat [1835: 345]	*Ptychodes* Audinet-Serville, 1835 [**7**]
*Purpuricenus* Ziegler [1835: 323]	*Purpuricenus* Dejean, 1821
*Pyrodes* Serville [1835: 318]	*Pyrodes* Audinet-Serville, 1832
*Rachidion* Serville [1835: 320]	*Rachidion* Audinet-Serville, 1834
*Rhagium* Fabricius [1835: 353]	*Rhagium* Fabricius, 1775
*Rhamnusium* Megerle [1835: 354]	*Rhamnusium* Latreille, 1829
*Rhinotragus* Germar [1835: 334]	*Rhinotragus* Germar, 1824
*Rhopalophora* Serville [1835: 333]	*Rhopalophora* Audinet-Serville, 1834
*Rhytiphora* Serville [1835: 343]	*Rhytiphora* Audinet-Serville, 1835 [**7**]
*Rosalia* Serville [1835: 323]	*Rosalia* Audinet-Serville, 1834
*Saperda* Fabricius [1835: 350]	*Saperda* Fabricius, 1775
*Saphanus* Megerle [1835: 329]	*Saphanus* Audinet-Serville, 1834
*Solenoptera* Serville [1835: 318]	*Solenoptera* Audinet-Serville, 1832
*Spathoptera* Serville [1835: 352]	*Spathoptera* Audinet-Serville, 1835 [**7**]
*Sphaerion* Serville [1835: 327]	*Sphaerion* Audinet-Serville, 1834
*Spondylis* Fabricius [1835: 316]	*Spondylis* Fabricius, 1775
*Steirastoma* Serville [1835: 336]	*Steirastoma* Lacordaire, 1830
*Stenoderus* Dejean [1835: 353]	*Stenoderus* Dejean, 1821
*Stenodontes* Serville [1835: 316]	*Stenodontes* Audinet-Serville, 1832
*Sternacanthus* Serville [1835: 319]	*Sternacanthus* Audinet-Serville, 1832
*Stenaspis* Serville [1835: 321]	*Stenaspis* Audinet-Serville, 1834
*Stenopterus* Illiger [1835: 334]	*Stenopterus* Illiger, 1804
*Stenygra* Serville [1835: 332]	*Stenygra* Audinet-Serville, 1834
*Stictosomus* Serville [1835: 317]	*Stictosomus* Audinet-Serville, 1832
*Stromatium* Serville [1835: 328]	*Stromatium* Audinet-Serville, 1834
*Taeniotes* Serville [1835: 340]	*Taeniotes* Audinet-Serville, 1835 [**7**]
*Tapeina* Encyclopédie [1835: 340]	*Tapeina* Lepeletier & Audinet-Serville, 1828
*Temnopis* Serville [1835: 328]	*Temnopis* Audinet-Serville, 1834
*Tetraopes* Dalman [1835: 347]	*Tetraopes* Schönherr, 1817
*Titanus* Serville [1835: 316]	*Titanus* Audinet-Serville, 1832
*Tmesisternus* Latreille [1835: 327]	*Tmesisternus* Latreille, 1829
*Toxotus* Megerle [1835: 354]	*Toxotus* Dejean, 1821
*Trachelia* Serville [1835: 322]	*Trachelia* Audinet-Serville, 1834
*Trachyderes* Dalman [1835: 320]	*Trachyderes* Dalman, 1817
*Trachysomus* Serville [1835: 343]	*Trachysomus* Audinet-Serville, 1835 [**7**]
*Tragidion* Serville [1835: 328]	*Tragidion* Audinet-Serville, 1834
*Tragocerus* Dejean [1835: 319]	*Tragocerus* Latreille, 1829
*Tragosoma* Dejean [1835: 318]	*Tragosoma* Audinet-Serville, 1832
*Trichophorus* Serville [1835: 326]	*Trichophorus* Audinet-Serville, 1834
*Trictenotoma* Gray [1835: 316]	*Trictenotoma* Gray, 1832
*Uracanthus* Hope [1835: 327]	*Uracantha* Hope, 1833
*Vesperus* Dejean [1835: 353]	*Vesperus* Dejean, 1821
*Xestia* Serville [1835: 322]	*Xestia* Audinet-Serville, 1834
*Xylotribus* Serville [1835: 345]	*Xylotribus* Audinet-Serville, 1835 [**7**]
*Xystrocera* Serville [1835: 328]	*Xystrocera* Audinet-Serville, 1834
**Tétramères: Chrysomélines**	
*Adimonia* Laicharting [1836: 376]	*Adimonia* Laicharting, 1781
*Adorium* Fabricius [1836: 375]	*Adorium* Fabricius, 1801
*Aegithus* Fabricius [1836: 427]	*Aegithus* Fabricius, 1801
*Agathidium* Illiger [1836: 431]	*Agathidium* Panzer, 1797
*Alurnus* Fabricius [1835: 360; 1836: 363]	*Alurnus* Fabricius, 1775
*Anisotoma* Fabricius. Gyllenhal [1836: 431]	*Anisotoma* Panzer, 1797
*Argopus* Fischer [1836: 394]	*Argopus* Fischer von Waldheim, 1824
*Auchenia* Megerle [1835: 359; 1836: 361]	*Auchenia* Thunberg, 1789
*Cassida* Linné [1836: 374]	*Cassida* Linnaeus, 1758
*Chelobasis* Chevrolat [1836: 366]	*Chelobasis* Gray, 1832
*Chlamys* Knoch [1836: 415]	*Chlamys* Knoch, 1801
*Chrysomela* Linné [1836: 400]	*Chrysomela* Linnaeus, 1758
*Clypeaster* Andersch [1836: 431]	*Clypeaster* Dejean, 1821
*Clythra* Laicharting [1836: 417]	*Clythra* Fabricius, 1798
*Colaspidea* De Laporte [1836: 411]	*Colaspidea* Laporte, 1833
*Colaspidema* De Laporte [1836: 411]	*Colaspidema* Laporte, 1833
*Colaspis* Fabricius [1836: 406]	*Colaspis* Fabricius, 1801
*Colasposoma* De Laporte [1836: 411]	*Colasposoma* Laporte, 1833
*Cryptocephalus* Geoffroy [1836: 422]	*Cryptocephalus* Geoffroy, 1762
*Cyrtonus* Dalman [1836: 399]	*Cyrtonus* Latreille, 1829
*Dibolia* Latreille [1836: 392]	*Dibolia* Latreille, 1829
*Donacia* Fabricius [1835: 356]	*Donacia* Fabricius, 1775
*Doryphora* Illiger [1836: 395]	*Doryphora* Illiger, 1807
*Erotylus* Fabricius [1836: 425]	*Erotylus* Fabricius, 1775
*Eumolpus* Kugelan [1836: 413]	*Eumolpus* Weber, 1801
*Euryope* Dalman [1836: 413]	*Euryope* Dalman, 1824
*Galleruca* Geoffroy [1836: 377]	*Galeruca* Geoffroy, 1762
*Haemonia* Megerle [1835: 357]	*Haemonia* Dejean, 1821
*Helodes* Fabricius [1836: 405]	*Helodes* Paykull, 1799
*Hispa* Linné [1836: 366]	*Hispa* Linnaeus, 1767
*Imatidium* Fabricius [1836: 367]	*Imatidium* Fabricius, 1801
*Lamprosoma* Kirby [1836: 415]	*Lamprosoma* Kirby, 1819
*Languria* Latreille [1836: 430]	*Languria* Latreille, 1802
*Lema* Fabricius [1835: 359; 1836: 362]	*Lema* Fabricius, 1798
*Lina* Megerle [1836: 402]	*Lina* Latreille, 1829
*Longitarsus* Latreille [1836: 392]	*Longitarsus* Latreille, 1829
*Luperus* Geoffroy [1836: 382]	*Luperus* Geoffroy, 1762
*Megascelis* Dejean [1835: 358; 1836: 361]	*Megascelis* Sturm, 1826
*Megalopus* Fabricius [1835: 358]	*Megalopus* Fabricius, 1801
*Notoclea* Marsham [1836: 395]	*Notoclea* Marsham, 1808
*Octogonotes* Drapiez [1836: 383]	*Octogonotes* Drapiez, 1820
*Oedionychis* Latreille [1836: 384]	*Oedionychis* Latreille, 1829
*Oides* Weber [1836: 375]	*Oides* Weber, 1801
*Omocera* Chevrolat [1836: 367]	*Omocerus* Chevrolat, 1835
*Orsodacna* Latreille [1835: 359; 1836: 361]	*Orsodacne* Latreille, 1802
*Paropsis* Olivier [1836: 395]	*Paropsis* Olivier, 1807
*Phaedon* Megerle [1836: 405]	*Phaedon* Latreille, 1829
*Phalacrus* Paykull [1836: 430]	*Phalacrus* Paykull, 1800
*Phyllocharis* Dalman [1836: 395]	*Phyllocharis* Dalman, 1824
*Podontia* Dalman [1836: 395]	*Podontia* Dalman, 1824
*Promechus* Dejean [1836: 395]	*Promechus* Boisduval, 1835
*Pseudocolaspis* De Laporte [1836: 414]	*Pseudocolaspis* Laporte, 1833
*Psylliodes* Latreille [1836: 393]	*Psylliodes* Latreille, 1829
*Rhaebus* Fischer [1835: 357]	*Rhaebus* Fischer von Waldheim, 1824
*Sagra* Fabricius [1835: 358]	*Sagra* Fabricius, 1792
*Timarcha* Megerle [1836: 399]	*Timarcha* Samouelle, 1819
*Triplax* Fabricius [1836: 429]	*Triplax* Herbst, 1793
*Trochalonota* Westwood [1836: 404]	*Trochalonota* Westwood, 1834
**Trimères**	
*Chilocorus* Leach [1836: 435]	*Chilocorus* Leach, 1815
*Coccidula* Megerle [1836: 438]	*Coccidula* Kugelann, 1798
*Coccinella* Linné [1836: 432]	*Coccinella* Linnaeus, 1758
*Dapsa* Ziegler [1836: 440]	*Dapsa* Latreille, 1829
*Dasycerus* Brongniart [1836: 440]	*Dasycerus* Brongniart, 1800
*Endomychus* Weber [1836: 439]	*Endomychus* Panzer, 1795
*Eumorphus* Weber [1836: 438]	*Eumorphus* Weber, 1801
*Lycoperdina* Latreille [1836: 440]	*Lycoperdina* Latreille, 1807
*Rhyzobius* Stephens [1836: 438]	*Rhyzobius* Stephens, 1829
*Scymnus* Herbst [1836: 438]	*Scymnus* Kugelann, 1794
**Dimères**	
*Batrisus* Aubé [1836: 442]	*Batrisus* Aubé, 1833
*Bryaxis* Knoch [1836: 441]	*Bryaxis* Kugelann, 1794
*Bythinus* Leach [1836: 441]	*Bythinus* Leach, 1817
*Chennium* Latreille [1836: 441]	*Chennium* Latreille, 1807
*Claviger* Müller [1836: 442]	*Claviger* Preyssler, 1790
*Ctenistes* Reichenbach [1836: 441]	*Ctenistes* Reichenbach, 1816
*Euplectus* Kirby [1836: 442]	*Euplectus* Leach, 1817
*Metopias* Gory [1836: 441]	*Metopias* Gory, 1832
*Pselaphus* Herbst [1836: 441]	*Pselaphus* Herbst, 1792
*Trimium* Aubé [1836: 442]	*Trimium* Aubé, 1833
*Tychus* Leach [1836: 442]	*Tychus* Leach, 1817
*Tyrus* Aubé [1836: 441]	*Tyrus* Aubé, 1833

^1^ A number in square brackets following the year of publication refers to the publication treated in the “Precedence” section where priority between the publication and Dejean’s catalogue is established. ^2^ We agree with Branco (2007: 14) that “*Hymenontia* Eschscholtz” listed by Dejean (1833: 165) is an incorrect subsequent spelling of *Hymenoplia* Eschscholtz. ^3^ *Phytobius* Dejean, 1835 was placed on the Official List of Generic Names in Zoology in Opinion 1529 (ICZN 1989b) but removed in Opinion 1968 (ICZN 2001). We believe that Dejean’s name is simply a subsequent usage of the name *Phytobius* Schönherr, 1833 since both authors proposed the name as “*Phytobius* Schmidt.” ^4^ *Sphaerogaster* was listed in synonymy with *Pachyrhynchus* Germar, 1824 by Germar (1833: 513). It was treated before 1961 as an available name and treated as a senior homonym of *Sphaerogaster* Zetterstedt, 1842 in various publications on Diptera (e.g., Bezzi 1902: 191; Kertész 1909: 96). Therefore the name *Sphaerogaster* Germar, 1833 is available (ICZN 1999: Article 11.6.1) and is a junior synonym of *Pachyrhynchus* Germar, 1824. ^5^ *Icthyosoma* was first proposed by Boisduval (1835: 468) as a junior synonym of *Tmesisternus* Latreille, 1829. It is available because it was treated before 1961 as an available name and adopted as the name of a taxon (e.g., Montrouzier 1856: 58). We consider that *Icthyosomus* used by Dejean (1835: 327) is an incorrect subsequent spelling of *Icthyosoma* Boisduval, 1835.

## Appendix

List of genera in Dejean’s catalogue (1833–1836) available prior to the publication of the catalogue.
